# A library of vinyl phosphonate anions dimerize with cyanostars, form supramolecular polymers and undergo statistical sorting†

**DOI:** 10.1039/d3sc03685e

**Published:** 2023-11-29

**Authors:** Yusheng Chen, Anastasia Kuvayskaya, Maren Pink, Alan Sellinger, Amar H. Flood

**Affiliations:** a Department of Chemistry, Indiana University 800 E. Kirkwood Avenue Bloomington Indiana 47405 USA aflood@indiana.edu; b Department of Chemistry, Colorado School of Mines 1012 14th Street Golden Colorado 80401 USA; c National Renewable Energy Laboratory (NREL) 15013 Denver West Parkway Golden Colorado 80401 USA

## Abstract

Supramolecular dimers are elementary units allowing the build-up of multi-molecule architectures. New among these are cyanostar-stabilized dimers of phosphate and phosphonate anions. While the anion dimerization at the heart of these assemblies is reliable, the covalent synthesis leading to this class of designer anions serves as a bottleneck in the pathway to supramolecular assemblies. Herein, we demonstrate the reliable synthesis of 14 diverse anionic monomers by Heck coupling between vinyl phosphonic acid and aryl bromide compounds. When this synthesis is combined with reliable anion dimerization, we show formation of supramolecular dimers and polymers by co-assembly with cyanostar macrocycles. The removal of the covalent bottleneck opened up a seamless synthetic route to iterate through three monomers affording the solubility needed to characterize the mechanism of supramolecular polymerization. We also test the idea that the small size of these vinyl phosphonates provide identical dimer stabilities across the library by showing how mixtures of anions undergo statistical (social) self-sorting. We exploit this property by preparing soluble copolymers from the mixing of different monomers. This multi-anion assembly shows the utility of a library for programming properties.

## Abstract 2

Stacking the deck in our favor. We make a single suit of anions with different faces but all assembling the same way inside cyanostars. With this strong hand, we play two cards together to form a supramolecular copolymer and beat solubility.

## Graphical abstract

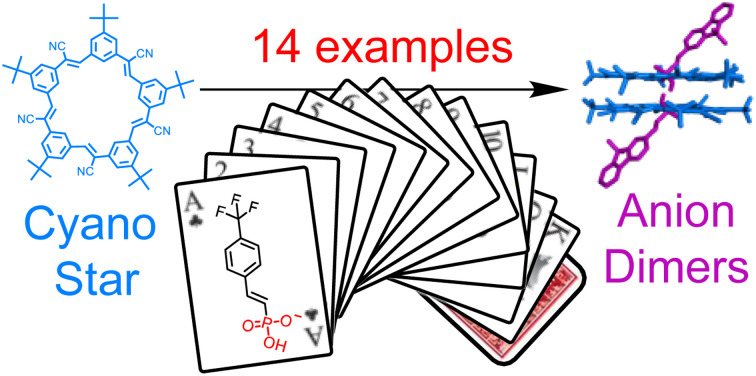

## Introduction

**Fig. 1:**
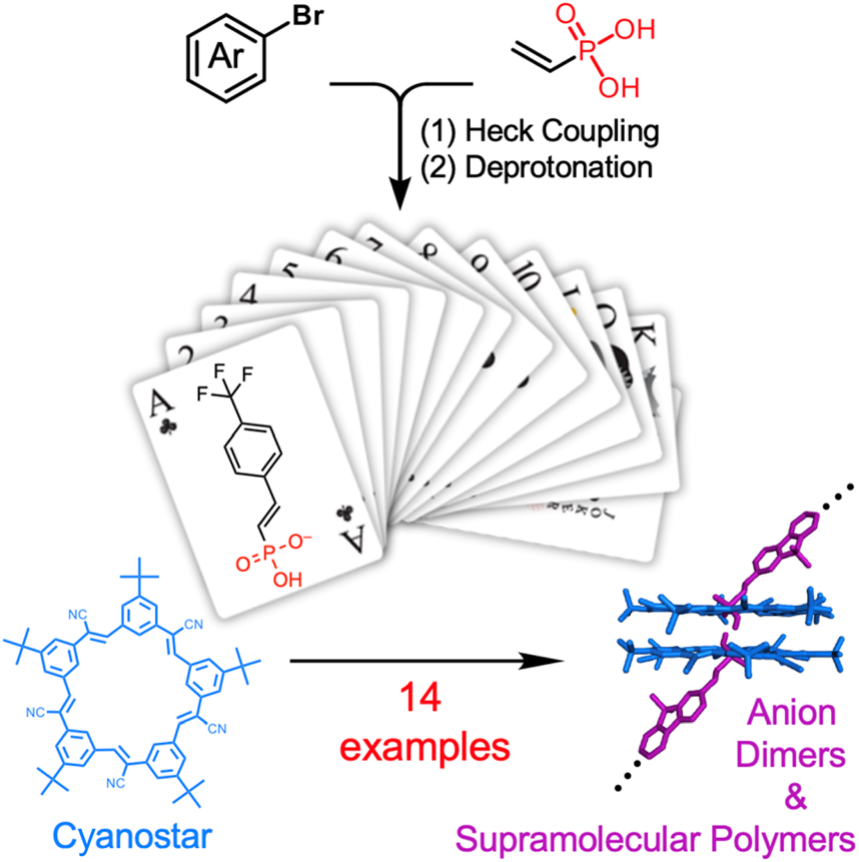
Modular synthesis of vinyl phosphonates to form a library of anions provide reliable and interchangeable assembly with cyanostar macrocycles into supramolecular dimers and polymers.

Supramolecular dimers consist of two molecules linked by non-covalent interactions.^1,2^ Well-established dimers are characterized by reliable non-covalent linkages that offer high stability and predictable stoichiometries.^3–13^ Examples are π systems linked by hydrogen bonding arrays,^14–16^ terpyridine ligands driven by metal-coordination^17,18^ and π-stacked dimers encapsulated by cucurbit[*n*]uril hosts.^19,20^ The discovery of reliable linkage chemistries led to the use of dimers in various applications^21–33^ and to construct sophisticated assemblies.^14,20,34–40^ The versatility of these dimers can be amplified when they are covalently modified to create a variety of molecular building blocks.^3,5,20,41^ For example,^35^ a variety of architectures were assembled from a series of guest molecules prepared using the same synthetic route. In principle, each building block will have the same dimerization propensity, which allows properties to be defined by the substituent. Despite this promise and the possibility of generating a library of building blocks by covalent modification, there are very few^4,42^ collections with more than 10 members. The recent discovery of cyanostar-stabilized anion dimers that form with 2 : 2 stoichiometries (Fig. 1)^43–47^ is a case in point where the synthesis of anion-based building blocks is a bottleneck. To remove this bottleneck, we leverage a vinyl-substituted phosphonic acid that can be readily substituted with aryl groups using Heck coupling for the creation of a 14-member library, with each forming supramolecular links as either dimers or polymers. We use the expedient synthesis and statistical self-sorting of the anions as complementary methods to tune polymer solubility and to showcase the utility of the library.

Receptor-stabilized anion–anion dimers are connected by *anti*-electrostatic hydrogen bonds (AEHB)^46–50^ that form inside receptors, *e.g.*, cyanostar macrocycles,^43,44^ Caballero's halogen-bonding receptors^51^ and others.^49,52–56^ These dimers have been used to construct supramolecular polymers^45,51^ leading to tunable, thermally-reversible adhesives.^57^ A key challenge, however, is preparation of the molecular building blocks. The synthesis and purification of organic hydroxyanions bearing hydrophilic headgroups (R-HPO_4_^−^, R-HPO_3_^−^) is sometimes unpredictable.^57–60^ As an example, only two organophosphates^57^ were synthesized for exploring their dimerization with eight of the other anions^43–45,61^ coming from commercial sources. These modest numbers suggest that these hydroxyanions fall short of the mark needed for conferring versatile access to subunit variation. Recently, Baran developed a phosphorylation method to access phosphoric acids.^60^ However, the reagent has limited substrate scope (aliphatic alcohols) and a cost ($500 per g) that inhibits prototyping of self-assembled materials. Ideally, these building blocks should be accessible in one or two efficient synthetic steps from a diversity of commercial building blocks.

Another opportunity that stems from generation of a library in which each member shares the same binding site is statistical self-sorting between members.^62–64^ This behavior is usually explored by mixing equimolar amounts of different building blocks with the host and evaluating the distribution of homodimers and inter-mixed heterodimers. The monomers can come together exclusively (integrative), segregate exclusively (narcissistic), show purely statistical outcomes (social) or variations thereof. Sorting has been investigated with dimers^65–67^ and used to generate alternating copolymers^68–70^ and diblock copolymers.^67,71^ The extent of sorting with anion–anion dimers, however, has not been reported to our knowledge.

Herein, we report the synthesis of 14 aromatic-based vinyl phosphonic acids using a mild Heck coupling approach (Fig. 1) and their subsequent quantitative deprotonation and dimerization with cyanostar, which exceeds the handful examined in the prior six publications.^43–45,57,61^ They were each shown to dimerize as high-fidelity complexes stabilized by a pair of cyanostars with 2 : 2 linkage stoichiometry at the equivalence point as observed using X-ray structures, ^1^H NMR spectroscopy and mass spectrometry. As a demonstration of the facile modularity, we iterated on a series of ditopic monomers using covalent synthesis to overcome solubility limits in a supramolecular homopolymer. We also tested the idea that the small size of the vinyl phosphonate would allow anion–anion linkages to have identical intermolecular affinities. We note, however, that small anions often lead to mixtures of macrocycles^43,53,61^ with a deterioration in the reliability of the 2 : 2 stoichiometry. Our results show, fortuitously, the ideal outcome of identical affinities. Thus, we leverage this property to convert an insoluble homopolymer into soluble forms of random copolymers using the statistical self-sorting between ditopic members in the library.

## Results and discussions

### Synthesis of vinyl phosphonate library

**Scheme 1:**
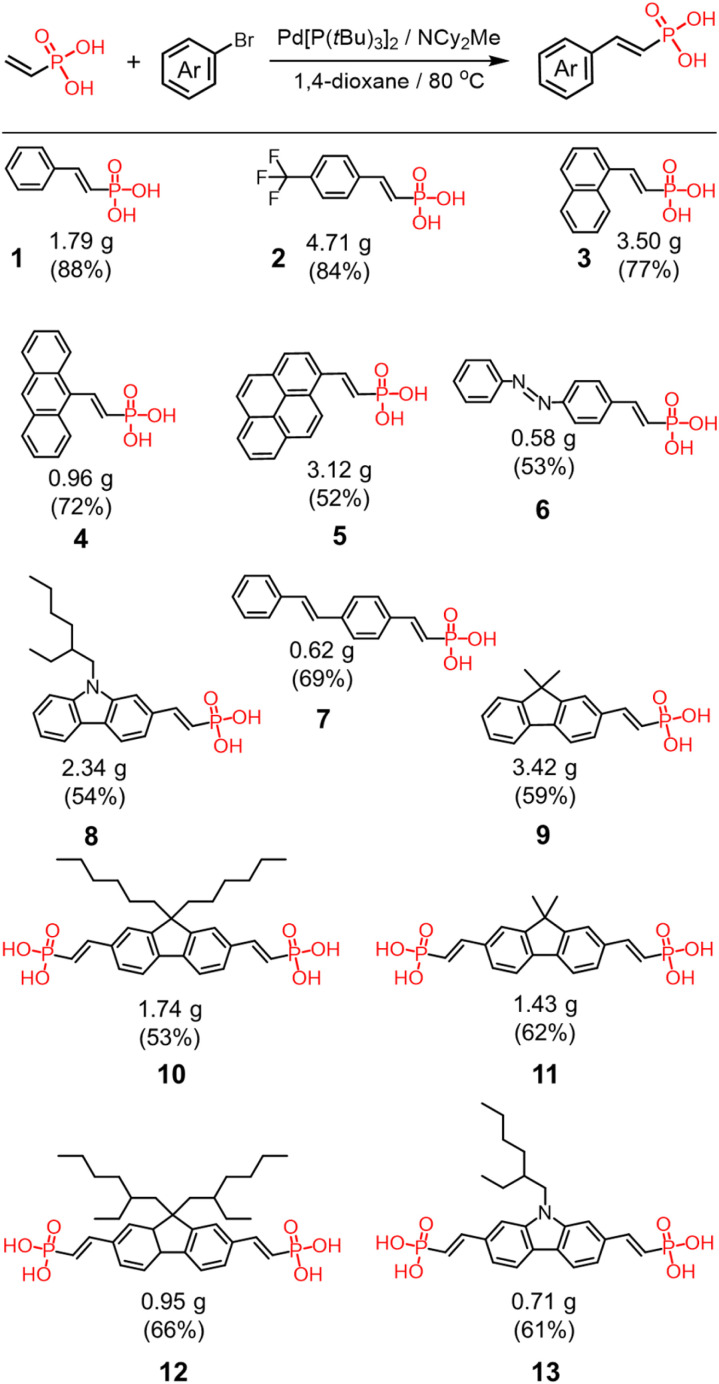
Summary of vinyl phosphonic acid synthesis using Heck coupling.

All the anions were prepared from the corresponding vinyl phosphonic acids. The acids were synthesized by Heck coupling between vinyl phosphonic acid and various aryl bromides (Scheme 1).^72,73^ This modular synthetic approach produces target compounds in good to excellent yields and on gram scales from a variety of aromatic halides, including halogenated chromophores and photoswitches (Scheme 1). All the synthesized compounds were isolated predominantly as (*Z*)-isomers, a trace amount of the (*E*)-isomer (≈34 : 1 *E*/*Z* ratio) was detectable in the crude mixture of 2. The acids were converted to the phosphonates by addition of stoichiometric amounts of tetrabutylammonium hydroxide (TBAOH) to yield the tetrabutylammonium (TBA^+^) salts.^45,57^ The salts were dried under vacuum before complexation with cyanostar macrocycle.

### Dimerization of fluorene vinyl phosphonate with cyanostar macrocycles

**Fig. 2:**
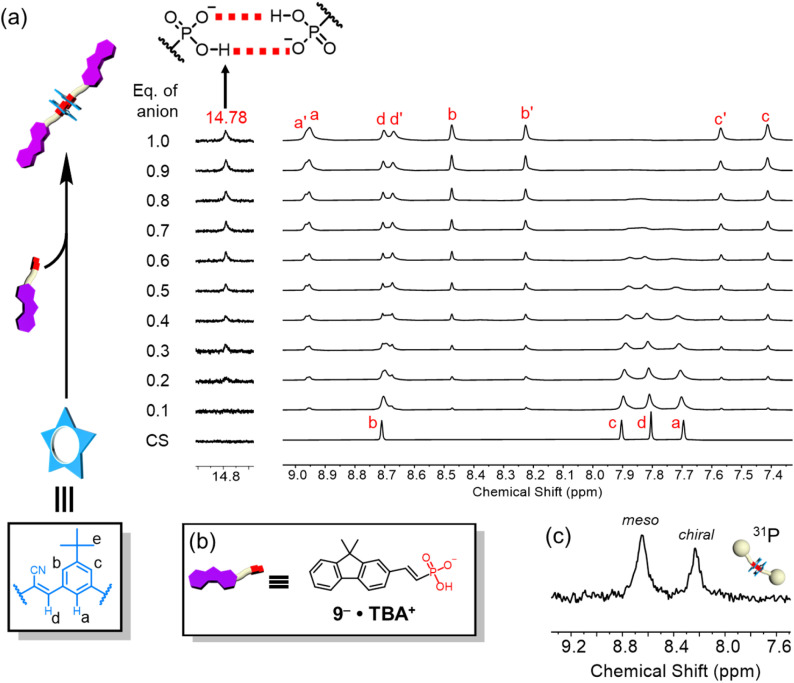
(a) Titration of dimethyl fluorene vinyl phosphonate 9 into cyanostar (1 mM, CD_2_Cl_2_, 298 K, 600 MHz). (b) Structure of tetrabutylammonium salt of dimethyl fluorene vinyl phosphonate. (c) ^31^P NMR spectrum at equivalence point (1 mM, CD_2_Cl_2_, 298 K, 202 MHz).

The formation of high-fidelity receptor : anion complexes based on *anti*-electrostatic hydrogen bonding was examined using dimethylfluorene-vinyl phosphonate 9 (Fig. 2b). Addition of this vinyl phosphonate to a cyanostar solution (1 mM, dichloromethane) confirmed formation of the 2 : 2 cyanostar : phosphonate complex at the equivalence point (1.0 equiv.). The appearance of the 14.78 ppm signal confirms^43,45^ formation of self-complementary hydrogen bonds between phosphonate head groups inside the cyanostars (Fig. 2a). We also observe the typical 8-line NMR pattern of the cyanostar dimer in 2 : 2 complexes.^43^ The cyanostar macrocycle has a bowl shape and forms a racemic mixture of *M* and *P* chiral forms. Dimerization of the macrocycle in anion complexes slows the exchange between *M* and *P* chiral forms with both *meso* (*MP*) and *chiral* (*MM*/*PP*) combinations observed as diastereomeric proton signals in a 57 : 43% ratio (*e.g.*, H_c_ : H_c′_)^74^ while retaining the racemic mixture. We also observe these two diastereomers reflected in the ^31^P-NMR signals in a 55 : 45% ratio (Fig. 2c). The similar relative peak intensities support the encapsulation of the two phosphonates inside the pair of cyanostars.

**Fig. 3:**
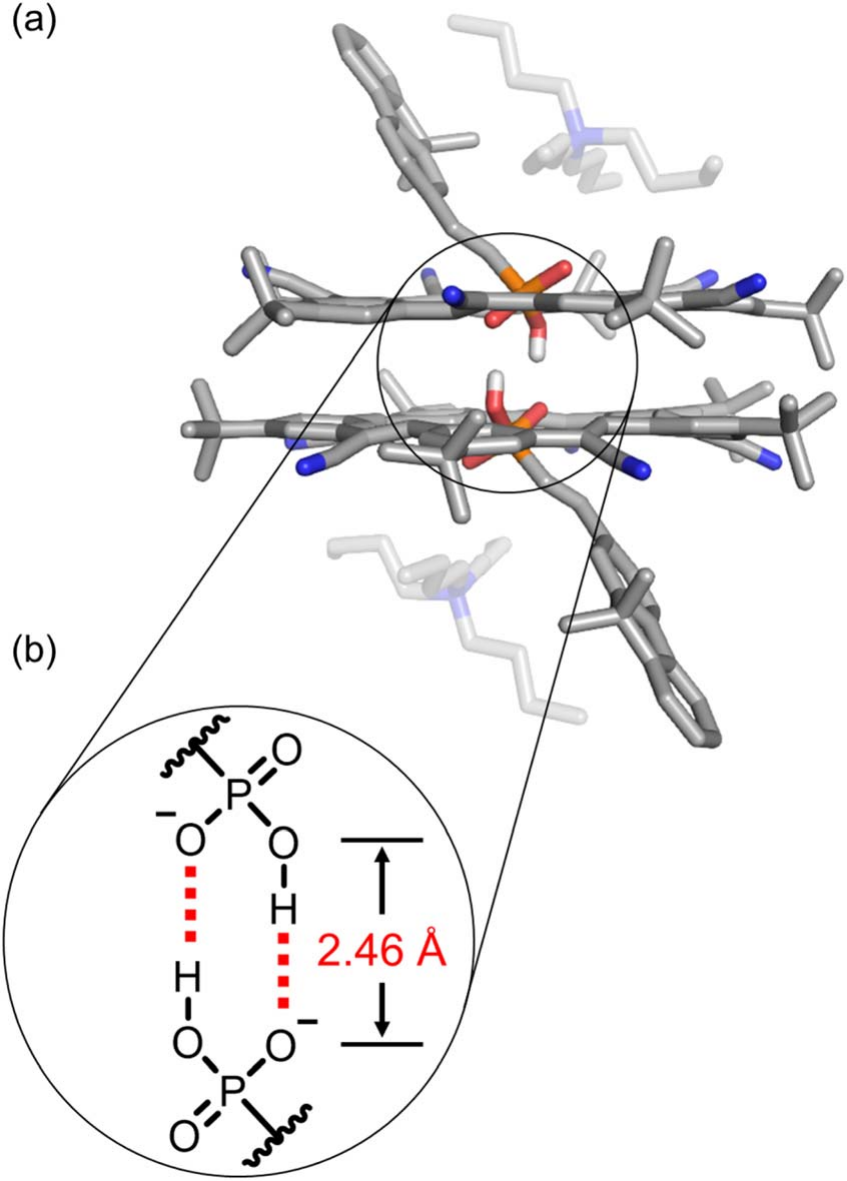
(a) Crystal structure of 2 : 2 cyanostar : phosphonate complex with 9 with the presence of two tetrabutylammonium cations. (b) The pair of self-complementary hydrogen bonds are observed at a O⋯O distance of 2.46 Å. Data collected at 150 K. Non-polar hydrogen atoms and disorder of cyanostar and phosphonate omitted for clarity (CCDC 2263985).

The 2 : 2 cyanostar : phosphonate complex was unambiguously confirmed using single-crystal structure determination (Fig. 3). The single crystal was obtained by vapor diffusion of *n*-hexane in a dichloromethane solution. The anion dimer is located inside the pair of π-stacked cyanostars with two tetrabutylammonium cations situated close to the anionic center. The two α-protons of TBA^+^ are hydrogen bonded (3.16 and 3.36 Å) with the external oxygen atom (Fig. 3a). The geometry of the phosphonate dimer shows a pair of short OH⋯O hydrogen bonds. The distance between donor and acceptor oxygens (2.46 Å), is consistent with the classification of very strong hydrogen bonding^75^ and in the range that is typical of hydrogen bonds between anionic dimers (Fig. 3b).^48,76^ The long and short bond lengths between phosphorous and oxygen atoms can be used to assign single and double bonds (Fig. 3b). Thus, the negatively charged oxygen atom is the hydrogen bond acceptor despite the greater Coulomb repulsion produced from two negatively charged centers so close together. We note that the cyanostar displays the whole-molecular disorder typical of their crystal packing preferences.^74^ We observed a 60 : 40 ratio between *M* and *P* isomers, which is consistent with the ratio of diastereomers seen in ^1^H NMR, *i.e.*, *meso* : *chiral* = 57 : 43%. We also observe two positions of the fluorene moiety with a 75 : 25% occupancy possibility, differing modestly by 11° in their tilt angle.

### Testing the sterics limits of assembly with cyanostar using vinyl phosphonate

**Fig. 4:**
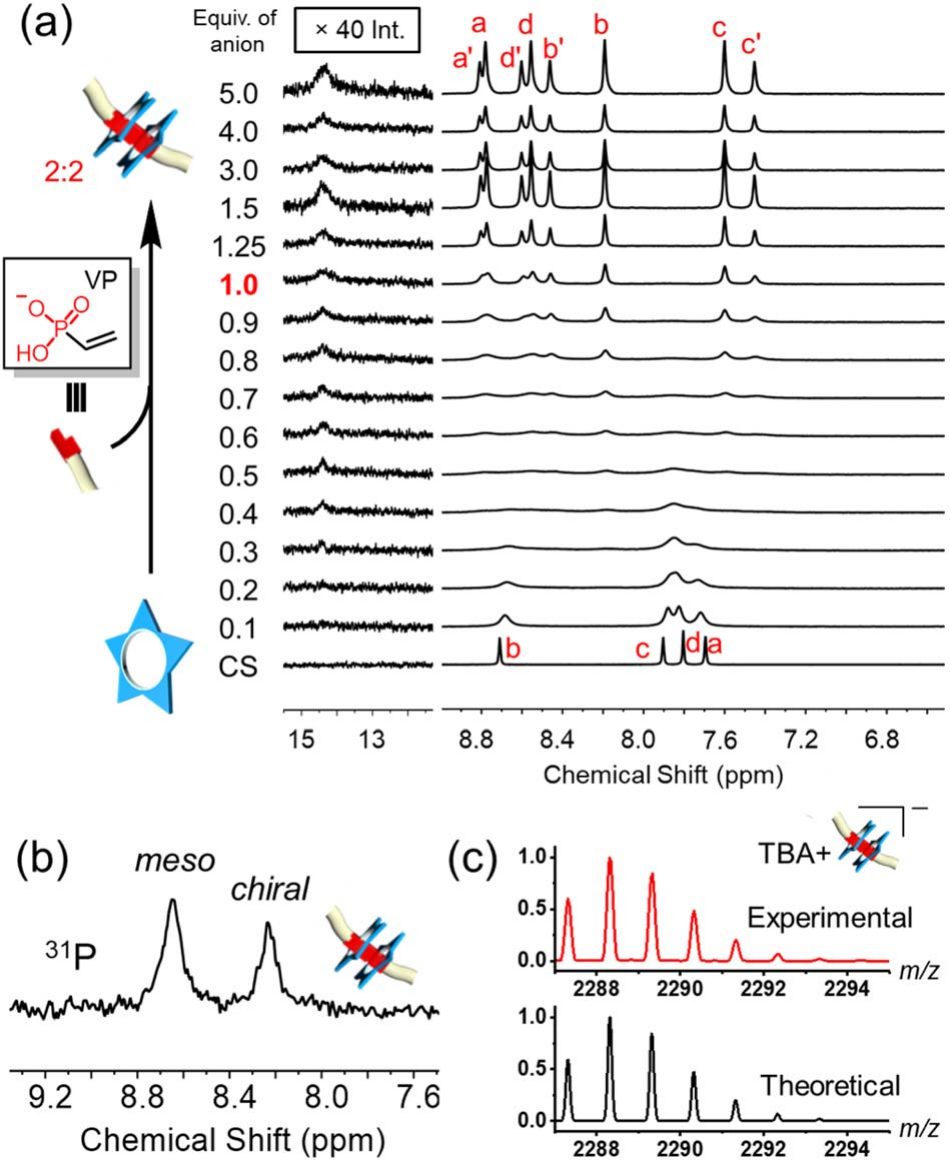
(a) Titration of vinyl phosphonate (VP) into cyanostar (1 mM, CD_2_Cl_2_, 298 K, 600 MHz), the downfield region was subjected to exponential apodization to make hydrogen bond signals easier to observe. (b) ^31^P NMR spectrum at equivalence point (1 mM, CD_2_Cl_2_, 298 K, 202 MHz). (c) High-resolution mass spectrum of cyanostar : vinyl phosphonate complex, ratio of cyanostar and vinyl phosphonate is 1 : 1.

Less bulky substituents on phosphates or phosphonates are known to produce a mixture of 2 : 2 stoichiometric and other 3 : 2 cyanostar : anion complexes that lower the reliability of dimerization.^43^ Therefore, to evaluate the limits of reliability, we tested the smallest analogue in the series, vinyl phosphonate (VP). When 1.0 equivalent of this anion was added to cyanostar, a clear signature for the diastereomeric cyanostar dimer and the 14 ppm hydrogen bonding shows formation of the 2 : 2 cyanostar : anion complex (Fig. 4a) consistent with the larger fluorene vinyl phosphonate (9). The hydrogen bonding signal at ∼14.5 ppm has low intensity. This phenomenon was also observed in the 2 : 2 cyanostar : bisulfate dimer in which the anion is small.^44^

All evidence supports formation of the target complex with 2 : 2 stoichiometry. Consistently, the diastereomeric phosphorous signals for the 2 : 2 complex can be observed by ^31^P NMR spectroscopy (Fig. 4b) and the parent ion is observed in the high-resolution mass spectrum at 2288.3193 *m*/*z* (Fig. 4c). Furthermore, addition of excess anion does not change the 8-line pattern seen in the aromatic region of the ^1^H-NMR spectra, indicating the 2 : 2 complex is the exclusive cyanostar-stabilized assembly beyond 1.0 equivalent.

Prior to the equivalence point, we observe a high degree of exchange broadening. The broadened NMR spectra could result from exchange between the 2 : 2 complex and either 3 : 2 species^43,61^ or the uncomplexed macrocycle. The absence of any peaks around 6.9 and 7.2 ppm (ref. 61) as well as the absence of a downfield shifted H-bond signature at around 13 ppm (ref. 43) exclude formation of the higher order 3 : 2 species (Fig. 4a). Therefore, we attribute the broadening of aromatic signals from 0.2 to 0.6 equivalents to exchange between complexed and uncomplexed macrocycle that occurs at rates close to the NMR time scale. This faster rate of exchange, relative to the larger fluorene analog (Fig. 2), likely results from the low steric profile of the smaller vinyl group.

### Solvent dependence of cyanostar–vinyl phosphonate dimers

Solvent polarity is known^61^ to affect the stoichiometry of complexation between cyanostar and anion. In the more polar acetonitrile solvent, the solvophobic effect favors formation of a triple-stack of macrocycle to form the 3 : 2 macrocycle : anion complex.^61^ To test if this behavior also emerges with vinyl phosphonates, the titration was performed in a polar solvent mixture selected to ensure solubility of all components (2 : 1 v/v dichloromethane: acetonitrile).^43,61^ Instead of the vinyl phosphonate anion, which displays broad proton signals, phenyl vinyl phosphonate (1) was selected. This anion shows distinct signals and maintains a high-fidelity 2 : 2 signature in pure dichloromethane. Consistent with previous reports,^43,61^ the titration shows signatures for both 3 : 2 and 2 : 2 complexes that maximize at their respective equivalence points, *i.e.*, 0.67 and >1.0 equivalents (Fig. S85†). These results show solvent polarity plays the same role in directing assembly outcome of vinyl phosphonate systems as with other cyanostar-stabilized anion dimers.

### Demonstrating the versatility of vinyl phosphonates as cyanostar-stabilized dimers

The reliable formation of 2 : 2 cyanostar : anion complexes motivated us to examine if the versatility of the vinyl phosphonates also support their use as a general platform for supramolecular dimerization. A series of monosubstituted aryl vinyl phosphonates were prepared using a variety of π systems, *e.g.*, benzene (1), 4-trifluoromethyl benzene (2), naphthalene (3), anthracene (4), pyrene (5), azobenzene (6), stilbene (7) and carbazole (8).

**Fig. 5:**
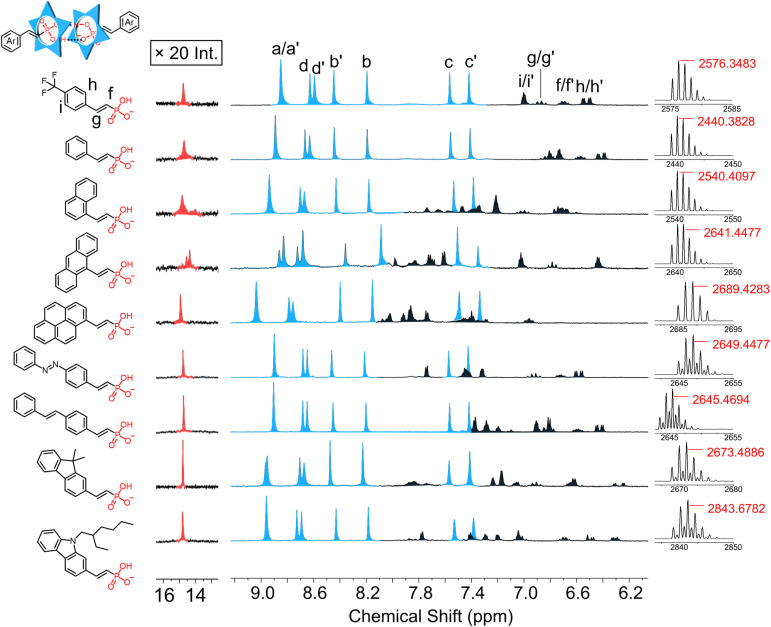
Stack of ^1^H NMR spectra of 2 : 2 dimers of cyanostar with 1 equivalent of the aryl vinyl phosphonate anions (1 mM, CD_2_Cl_2_, 298 K, 600 MHz). Peaks from cyanostar are shown in blue, peaks from phosphonates are shown in black, and the hydrogen bonding signal is colored red. The downfield region (∼14 ppm) was subjected to exponential apodization (3 Hz) to improve the signal-to-noise ratio of the hydrogen-bond region. The remainder of the spectrum apodization was set to 0.3 Hz. All counter-cations are tetrabutylammonium. High-resolution electrospray ionization mass spectra of 2 : 2 dimers of cyanostar with aryl vinyl phosphonate anions (1 mM, CD_2_Cl_2_) at the 1 : 1 equivalence point. Samples for high resolution ESI mass analysis were directly infused with a constant spray voltage of 2.7 kV.

Assembly of 4-trifluoromethylphenyl vinyl phosphonate (2), with cyanostar is exemplary of dimerization. Upon addition of 1 equivalent of the phosphonate to the cyanostar solution, we observed the characteristic aromatic and hydrogen bonding pattern of the 2 : 2 dimer (Fig. 5, top). The proton assignments for the complexed dimers were based on their splitting pattern and coupling constants. Interestingly, these vinyl phosphonate signals are also subject to the influence of the *meso* and *chiral* cyanostar dimers with intensity ratios for the diastereomers that match cyanostar. Consistent with the complexed architecture, ROESY NMR experiments show correlations between macrocycle protons assigned to the *meso* dimer (*e.g.*, H_d′_ at 8.62 ppm) and the *meso* form of the aryl-vinyl-phosphonate (*e.g.*, H_f′_ at 6.68 ppm) (Fig. S88†). Similar correlations were observed between the macrocycle and phosphonate for the *chiral* isomers. These correlations confirm that the splitting observed in the aromatic signals for the aryl-vinyl-phosphonate anion stems from *meso* and *chiral* forms of the cyanostar dimers.

All other aryl vinyl phosphonates showed identical signatures with high-fidelity formation of 2 : 2 cyanostar : anion complexes at the equivalence point (Fig. 5). Diastereomeric peaks for the cyanostar dimers of these complexes are located in the same aromatic region (7.3–9.1 ppm). The assembly of anthracene vinyl phosphonate with cyanostar also shows visible splitting of proton H_a_ into its diastereomers. This enhanced splitting is carried over to splitting in the resonance for the hydrogen bond between each phosphonate dimer. All others show a single broaden peak and are located in a range 14–15.5 ppm.

The high-resolution electrospray ionization (ESI) mass spectra (Fig. 5) are all consistent with 2 : 2 dimer formation. The parent ions of these complexes were all observed in the range from 2400 to 2900 *m*/*z* as a consequence of different substituents. In the case of a few, we also observe multimers that are attributed to aggregation in the electrospray experiment.

We also obtained the crystal structure for the phenyl vinyl phosphonate (1) dimer in both its solvated and fully desolvated state (Fig. S119,† CCDC 2263986 and 2263987), showing a crystal-to-crystal transition of the 2 : 2 dimer upon desolvation.^77^

### Pure statistical sorting across the modular anion library

We designed the library to introduce various functional groups from the aryl substituents while leaving the linkage chemistry to be determined solely by the vinyl phosphonate without influence from sterics or electronics. The sterics are the same across the library and the calculated ESP values do not differ much suggesting support for this hypothesis (Fig. S134†). To test this idea, we studied the sorting behavior of the anions.^62^

**Fig. 6:**
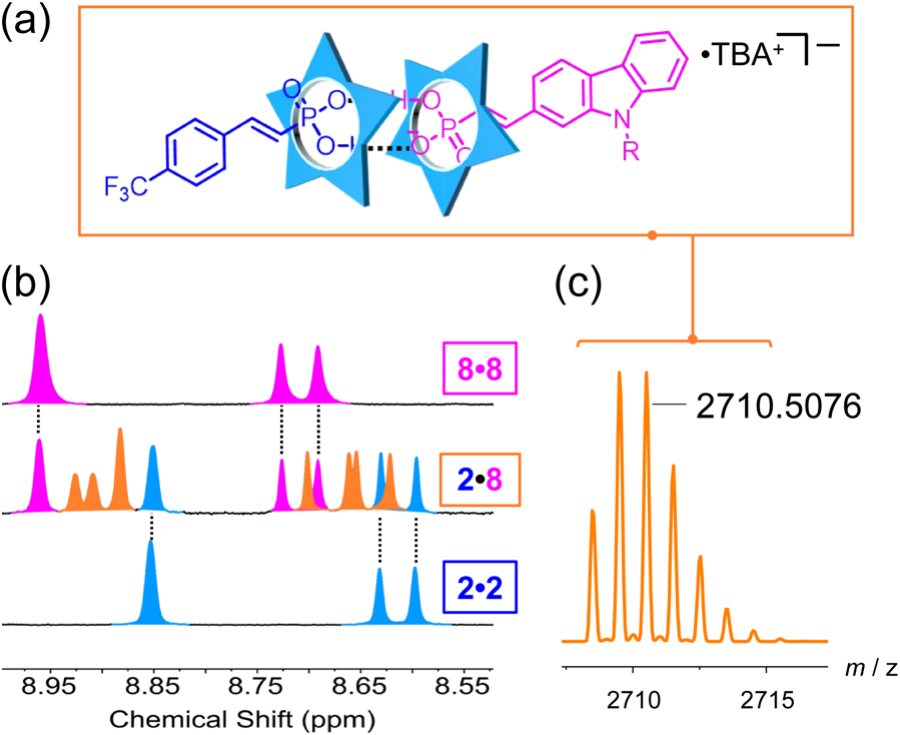
(a) Heterodimer structure containing 2 (blue) and 8 (magenta). (b) ^1^H NMR spectra of homodimer 2·2 (blue), heterodimer 2·8 (orange) and 8·8 (magenta). (c) High-resolution mass spectrum of the parent heterodimer ion (1 mM, 298 K, 600 MHz, CD_2_Cl_2_).

We selected the electron-poor phosphonate (2) with an electron withdrawing group and the electron-rich carbazole phosphonate (8) to test sorting. We expect to see the 2·8 heterodimer (Fig. 6a) together with the homodimers (2·2 and 8·8). When the two vinyl phosphonates are mixed together, we observe a new set of peaks between the parents (Fig. 6b) assigned to the heterodimer 2·8, which was verified by an ESI-MS peak at 2710.5076 *m*/*z* (Fig. 6c). The integration ratio of the protons for 2·2, 2·8 and 8·8 is in the 1 : 2 : 1 ratio expected of statistical self-sorting (Fig. S125†).^62–64^ A competition titration supports this interpretation (Fig. S126†) indicating the affinities of anions 2 and 8 are the same. These results confirm our idea that binding of vinyl phosphonates to cyanostar do not depend significantly on their substituents.

To connect the vinyl phosphonates with different hydroxyanions, we also performed sorting and competition studies between vinyl phosphonate 8 and naphthyl phosphate (P). Interestingly, we also observed statistical self-sorting where the heterodimer was confirmed by ^1^H NMR and ESI-MS (Fig. S128 and S133†). However, a competition study shows that the 1-naphthyl phosphate binds to cyanostar stronger than the vinyl phosphate (Fig. S129 and S130†).

We leveraged this stronger binding guest to drive depolymerization of a supramolecular polymer formed from ditopic vinyl phosphonate (see below). In this case, the depolymerization was produced by adding 1.0 equiv. of 1-naphthyl phosphate (Fig. S131†).

### Polymerization of the ditopic vinyl phosphonate with cyanostar macrocycle

**Fig. 7:**
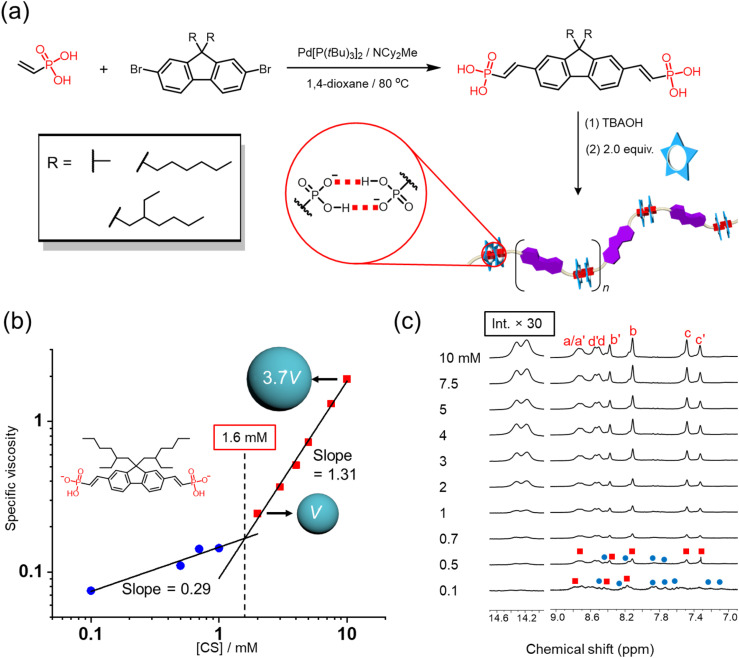
(a) Scheme of fluorene-based monomer synthesis and supramolecular polymerization. (b) Characterization of polymerization using double logarithmic plots of specific viscosity *versus* cyanostar concentration with 0.5 equiv. of the diphosphonate 12 (room temperature, CH_2_Cl_2_) and the normalized species size in 2 and 10 mM solution based on diffusion NMR. (c) Stack of ^1^H NMR spectra of variable concentrations complex solution with 2 : 1 stoichiometry of cyanostar : phosphonate (CD_2_Cl_2_, 298 K, 600 MHz).

The modular synthesis and reliable dimerization of aryl vinyl phosphonates provide an opportunity to consider formation of supramolecular polymers. For this purpose, dibromofluorene-based building blocks were subjected to Heck coupling to yield the di-vinyl phosphonic acid analogues. The diacid was ionized with TBAOH to obtain the dianion as a divergent ditopic monomer, then mixed with cyanostar macrocycle in a 1 : 2 ratio to match the ideal stoichiometry of polymers (Fig. 7a). The first ditopic monomer we prepared was dimethyl fluorene bis(vinyl phosphonate) (11). When cyanostar is added to drive anion dimerization, the solubility of the resulting polymer is quite low with irreversible precipitation appearing at about 1 mM, which was also observed in a prior polymer driven by anion dimerization^45^ and in other supramolecular polymers.^37,78^ By taking advantage of the modular synthesis, we prepared the fluorene monomer (10) with two hexyl chains in the 9-position but observed precipitation at 2 mM. Subsequently, we prepared a ditopic monomer based on a substituted carbazole bearing one 2-ethylhexyl side chain (13) but again precipitation occurred at 1 mM. Ultimately, use of a fluorene monomer with branched alkyl chains (12), *i.e.*, 2-ethylhexyl, significantly improved polymer solubility. As a consequence, variable concentration studies were performed without precipitation to help determine the likely mechanism of polymerization.

As observed previously,^45,57^ a key signature of supramolecular polymerization is the observation of threshold behavior in viscosity as a function of concentration. To determine this threshold concentration, *i.e.*, critical polymerization concentration (CPC), the specific viscosity of the polymer solutions was recorded from 0.1 to 10 mM based on the cyanostar concentration. The double-logarithmic plot of specific viscosity *versus* concentration (Fig. 7b) shows a linear relationship with an initial slope of 0.29, indicating noninteracting assemblies in solution.^79,80^ However, at a higher concentration range, the slope changes to 1.31. This stronger concentration dependence indicates the formation of polymers with increased size capable of entaglement under flow.^79,80^ Consistently, the diffusion coefficient derived from NMR peaks (H_b_, H_b′_, H_c_, H_c′_) assigned to the polymers decreases from 9.6 ± 0.3 to 6.2 ± 0.1 × 10^−11^ m^2^ s^−1^ at 2 and 10 mM, respectively. Based on the Stokes–Einstein equation for a spherical particle,^45^ we calculated that the normalized volume of the average solution species increases 4-fold from 2 to 10 mM (Fig. 7b). Consistent with previous reports,^45^ variable concentration ^1^H NMR spectra also provide evidence of the existence of oligomers below CPC (Fig. 7c). At 0.1 mM, multiple peaks appear in the range from 7.3–9.1 ppm, instead of typical 8-line pattern, which indicate the coexistence of oligomers and polymers. As concentration increases, the intensity of peaks assigned to polymer gradually increases concomitant with the disappearance of peaks assigned to oligomers beyond 2 mM, consistent with the CPC.

The intersection of the two trendlines determines the CPC as 1.6 mM based on cyanostar.^2^ This concentration lies between the previously reported critical polymerization concentration of rigid ditopic phosphate (below 1 mM)^57^ and flexible ditopic phosphonate (5 mM) system that is driven by the anion dimerization inside the cyanostars.^45^ According to the relationship between monomer rigidity and critical polymerization concentration predicted by polymer physics, the more rigid monomer gives lower polymerization concentration.^81^ Therefore, this moderate critical polymerization concentration is consistent with semi-rigid property of the fluorene-based monomer.

### Copolymerization between vinyl phosphonate monomers

**Fig. 8:**
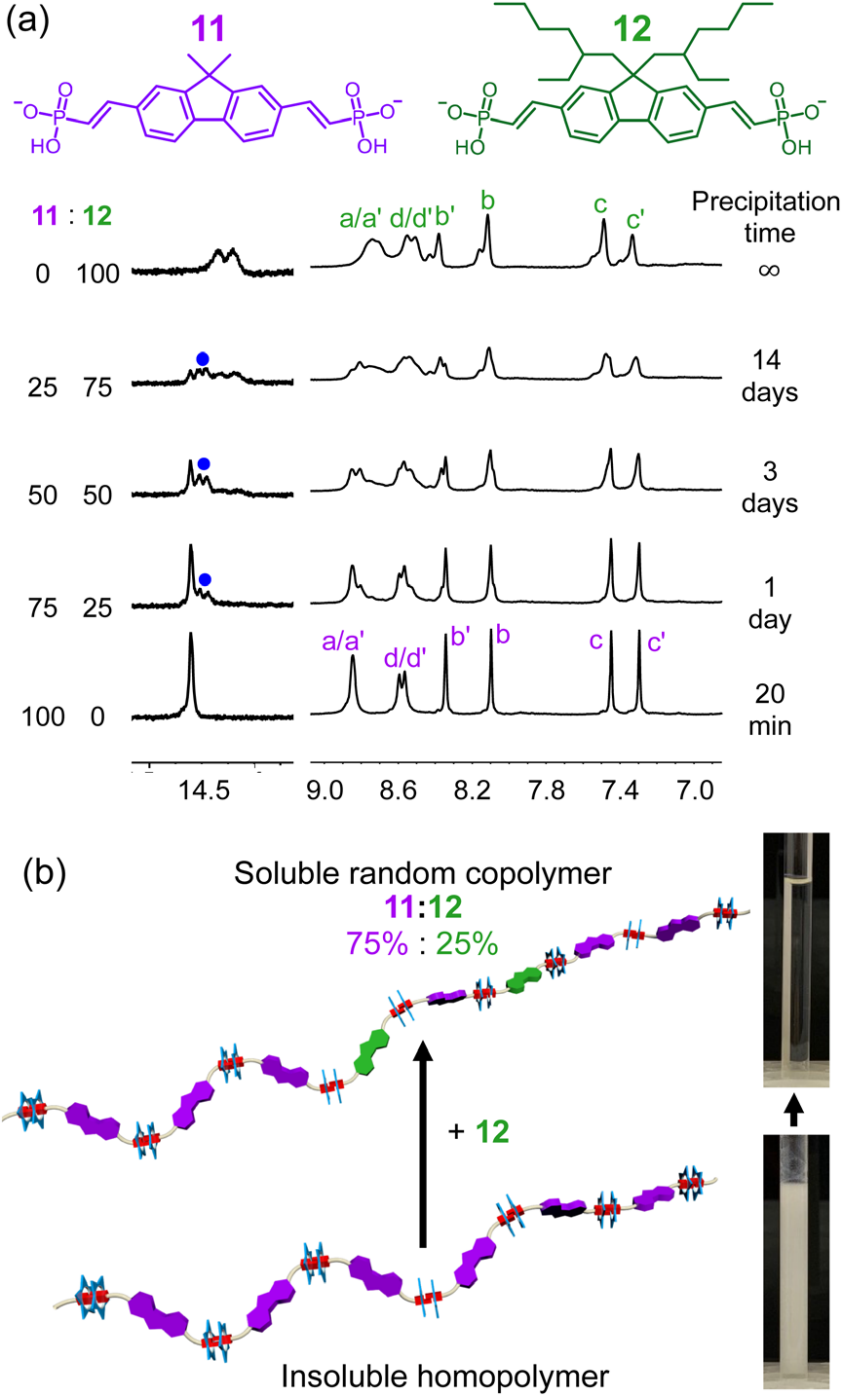
(a) ^1^H NMR spectra of homopolymers and copolymers involving 11 and 12 (5 mM cyanostar, 298 K, 600 MHz, CD_2_Cl_2_). (b) Pictures of solutions and representations of the homopolymer formed with monomer 11 and a copolymer of monomer 11 and 12.

In addition to using iterative synthesis to yield soluble monomers, the sorting results inspired us to explore copolymerization by doping the high solubility monomers into the insoluble homopolymer (Fig. 8a). The dimethyl-decorated monomer (11) of poorest solubility was selected for forming a copolymer with the fluorene monomer with 2-ethylhexyl side chain (12). When equal amounts of 11 and 12 were mixed with cyanostar, a hydrogen bond signature of the heterodimer 11–12 is observed at 14.5 ppm (Fig. 8a, blue dot), which sits between the two homodimer signals at 14.25 and 14.6 ppm. When the molar percentage of 11 is increased, we observed a change in intensities consistent with statistical sorting.

Macroscopically, precipitation of the homopolymer involving 11 occurred after 20 minutes, while no precipitation was observed in the copolymers over the same time frame. Consistent with copolymerization, the diffusion coefficient derived from NMR peaks (H_b_, H_b′_, H_c_, H_c′_) of the copolymer formed from a 50–50 mix of 11 and 12 ranges 6.2–6.5 × 10^−11^ m^2^ s^−1^ at 5 mM, indicating the two monomers are converted to one single polymeric assembly instead of different homopolymers. The diffusion coefficient of the homopolymer formed from 12 and the cyanostar (9.3 ± 0.2 × 10^−11^ m^2^ s^−1^, 5 mM) is larger than the copolymer and indicates that the average size is smaller. Based on the observation that polymerization of 11 gives insoluble precipitation, we reason that the copolymer exhibits stronger aggregation than the homopolymer of 12 alone.

## Conclusion

Aryl vinyl phosphonates fulfill all the hallmarks of a versatile platform for anion–anion dimerization inside a pair of cyanostar macrocycles including modular access to 14 building blocks that form small molecules, macromolecules, and statistical self-sorting combinations. All the assemblies form 2 : 2 linkages in high fidelity as confirmed by characteristic ^1^H NMR signals, mass spectra and X-ray crystal structures. The on-demand synthesis of vinyl phosphonic acids and corresponding vinyl phosphonates allowed access to solubility-tuned ditopic monomers for preparing supramolecular polymers. The concentration-driven polymerization exhibits a critical polymerization concentration of 1.6 mM, which is consistent with prediction from polymer physics for semi-rigid monomers in our anion-driven supramolecular polymerization system. The isosteric vinyl phosphonate headgroups allow statistical self-sorting between building blocks with different aryl substituents. Copolymerization between two monomers provided the basis for tuning macromolecular solubility. The versatility of vinyl phosphonates, their modular synthesis, and their identical intermolecular affinities opens up cyanostar-stabilized anion dimers as a platform for prototyping functional supramolecular materials.

## Data availability

The ESI† is available free of charge on the journal website. General methods, synthetic procedure, NMR titrations, 2D NMR spectroscopy, X-ray diffraction analyses, and ESI-MS analyses are included.

## Author contributions

YC and AK conceived the project under the supervision of AHF and AS; AK synthesized and characterized all phosphonic acids; YC prepared all phosphonates and conducted assembly and crystal growing experiments; MP collected X-ray diffraction data, solved and refined the crystal structures; YC, AK, AS and AHF analyzed the data; YC and AHF wrote and edited the manuscript with input from AK, AS and MP.

## Conflicts of interest

The authors declare no competing financial interests.

## Supplementary Material

SC-015-D3SC03685E-s001

SC-015-D3SC03685E-s002
