# Supplementary material for: A library of vinyl phosphonate anions dimerize with cyanostars, form supramolecular polymers and undergo statistical sorting
Source: Chem Sci. 2023 Nov 29;15(1):389–98. doi: 10.1039/d3sc03685e (PMC10732014; doi:10.1039/d3sc03685e)
Supplement: SC-015-D3SC03685E-s001 [file SC-015-D3SC03685E-s001.pdf]

Electronic Supplementary Material (ESI) for Chemical Science.
This journal is © The Royal Society of Chemistry 2023

Electronic Supplementary Information

A Library of Vinyl Phosphonate Anions Dimerize with Cyanostars, form Supramolecular Polymers and Undergo Statistical Sorting

Yusheng Chen,[a] Anastasia Kuvayskaya,[b] Maren Pink,[a] Alan Sellinger[b,c] and Amar H. Flood[a]

a. Department of Chemistry, Indiana University, 800 E. Kirkwood Avenue, Bloomington, Indiana, 47405, U.S.A.
b. Department of Chemistry, Colorado School of Mines, 1012 14th Street, Golden, Colorado, 80401, U.S.A.
c. National Renewable Energy Laboratory (NREL), 15013 Denver West Parkway, Golden, Colorado 80401, USA
*Corresponding E-mail: aflood@indiana.edu

Table of Contents:

S1. General Methods
S2. Synthesis and Characterization of Vinyl Phosphonic Acids
S3. Standard Procedure of Converting Phosphonic Acid to Tetrabutylammonium Phosphonate
S4. ^{1}H NMR Spectra of Titration Experiments
S5. HRMS of Versatile Cyanostar-anion Dimers
S6. Characterization of Supramolecular Polymer based on Di-vinyl Phosphonates
S7. X-ray Diffraction Data Analysis
S8. Sorting, Competition and Depolymerization Based on Modular Anion Library
S9. Supramolecular Polymer Literature Including Multiple Monomers
S10. References

S1. General Methods

Reagents were obtained from commercial suppliers and used as received unless otherwise indicated. Column chromatography was performed on silica gel (160–200 mesh, Sorbent Technologies, USA). Thin-layer chromatography (TLC) was performed on pre-coated silica gel plates (0.25 mm thick) and observed under UV light. 1D Nuclear magnetic resonance (NMR) spectra and titrations were recorded on Varian Inova (500 and 600 MHz) and Bruker Avance Neospectrometers (500 MHz) at room temperature (298 K). ROESY NMR was recorded on Bruker Avance Neospectrometers (500 MHz) spectrometer at room temperature (298 K). Chemical shifts were referenced to residual solvent peaks. Samples for high resolution ESI mass analysis were directly infused into a ThermoFisher LTQ Orbitrap XL at a rate of 8 – 12 uL/min from THF/DCM. The HESI II source was kept at 50 °C with a spray voltage of 2.7 kV. Sheath and Aux gases were set to 20 and 5 (arbitrary units) but were varied as needed to maintain a stable spray. Ion transfer tube was held at 275 °C. Tube lens and capillary voltages were varied to ensure transmission of ions to the detector.

Experimental method for NMR titration

A solution of cyanostar macrocycle was prepared in an NMR tube sealed with a silicone septum and an initial spectrum was taken. A solution of phosphonate salt was also prepared and added to the solution of cyanostar macrocycle with known quantities, the spectrum was recorded after each addition. All the spectra data were analyzed by using MestReNova software.

Experimental method for diffusion NMR

The diffusion coefficients were then obtained based on the method of pulse gradient spin echo (PGSE) experiments. Aromatic regions were analyzed in this way to determine diffusion coefficients using Vnmrj's analysis. Average diffusion coefficients and errors were generated from multiple peaks used in analyses.

Experimental method for viscosity experiments

A series of solutions of cyanostar macrocycle with 0.5 equiv. of fluorene-based diphosphonate as tetrabutylammonium salt were prepared. Every sample was filtered with a syringe filter membrane before collection. Each sample was measured three time and averaged for comparison. Efflux time were collected and converted to specific viscosity.

S2. Synthesis and Characterization of Vinyl Phosphonic Acids

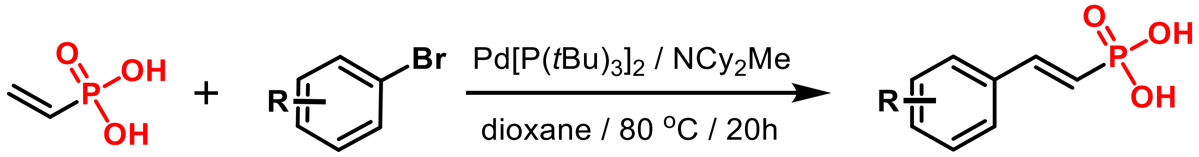

Scheme S1. General synthetic scheme of vinyl phosphonic acid.

(E)-Styrylphosphonic acid (Phenyl vinylphosphonic acid (1))

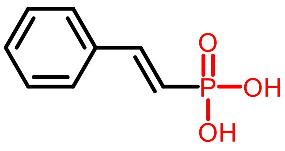

A 250 mL Schlenk flask with stir bar was oven dried. The Schlenk flask was charged with bromobenzene (1.74 g, 11.1 mmol) and bis(tri-tert-butyl phosphine)palladium(0) (0.142 g, 0.276 mmol), followed by vacuum and N_2 refill cycles (3×). Vinyl phosphonic acid (VPA) (1.44 g, 13.3 mmol) was dissolved in 10 mL of anhydrous dioxane and gently bubbled with N_2 for 20 min. Anhydrous dioxane (70 mL) and the solution of VPA was added to the Schenk flask *via* syringe with stirring. N,N-Dicyclohexylmethylamine (7.10 mL, 33.3 mmol) was added to the reaction mixture dropwise *via* syringe. The reaction was stirred to 80°C for 20 h. The progress was monitored with thin layer chromatography (EtOAc/Hexanes 1:4). Upon reaction completion, mixture was cooled to room temperature. The product was extracted with EtOAc and washed with 5% HCl (3×). Organic phase was dried over $MgSO_4$ and filtered. Obtained organic phase was condensed to a few mL by rotary evaporator. The product was precipitated into DCM and filtered precipitate (white flaky crystals) was dried overnight at 45°C under vacuum. Yield of 1.79 g (88%). ^{1}H NMR (500 MHz, DMSO-d_6) δ 10.25 (s, 2H), 7.59 (d, J = 6.2 Hz, 2H), 7.37 (t, J = 8.2 Hz, 3H), 7.21 (dd, J = 22.0, 17.5 Hz, 1H), 6.51 (dd, J = 17.4, 16.5 Hz, 1H). ^{31}P NMR (202 MHz, DMSO-d_6) δ 14.37. ^{13}C NMR (126 MHz, DMSO-d_6) δ 143.17 (d, J = 6.5 Hz), 135.48, 135.30, 129.57, 128.92, 127.48, 121.19, 119.73.

The purity of the compound was determined from ^{1}H, ^{13}C, and ^{31}P NMR. Integration of the ^{1}H NMR in addition to the single peak in the ^{31}P NMR and the absence of the signal attributed to the starting VPA indicated high compound purity (>99%). The doublet at 143.17 ppm in ^{13}C NMR is assigned to the carbon adjacent to the phosphorus, the splitting is attributed to the ^{31}P-C coupling.

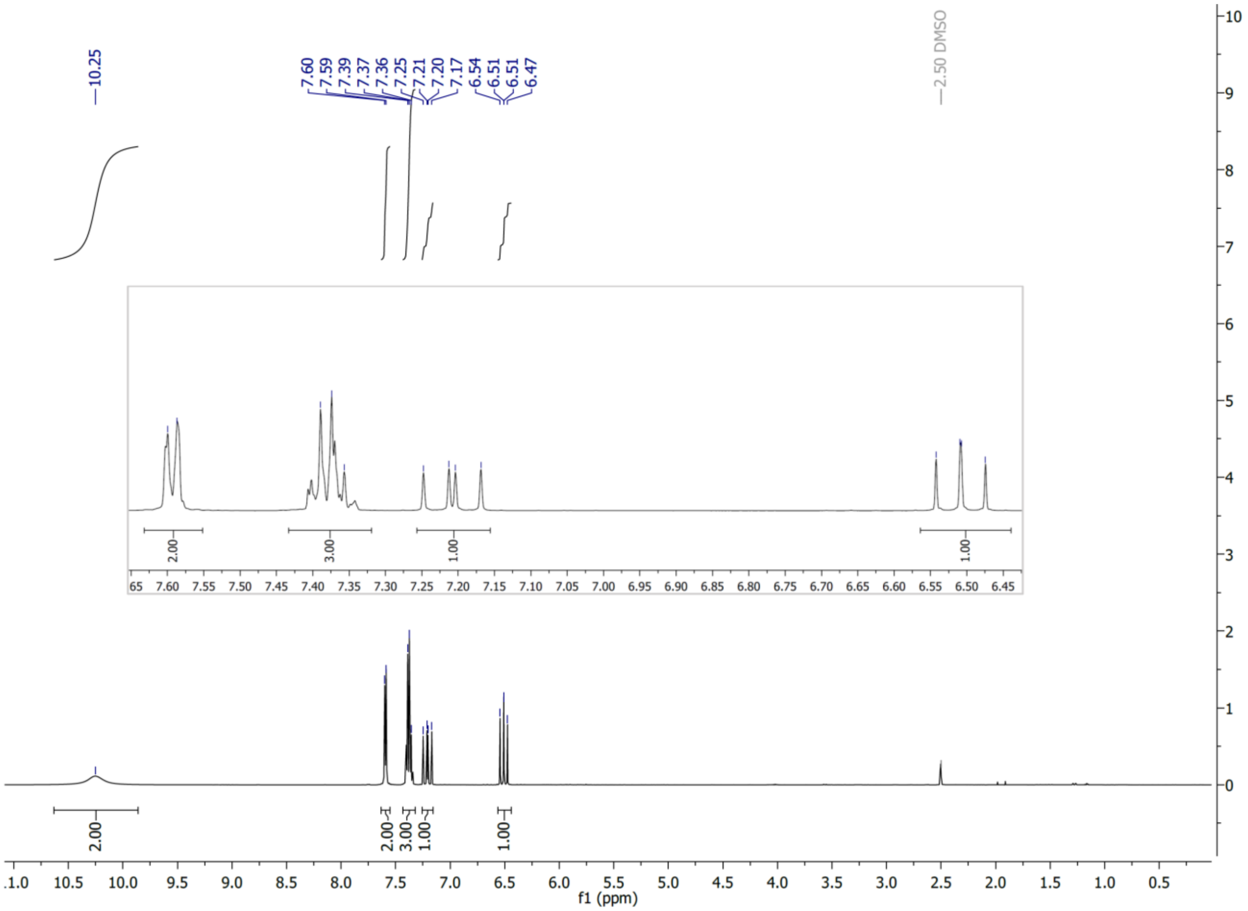

Figure S1. ^{1}H NMR spectrum of (E)-styrylphosphonic acid (DMSO- d_6 δ 2.50, 500 MHz, 298 K)

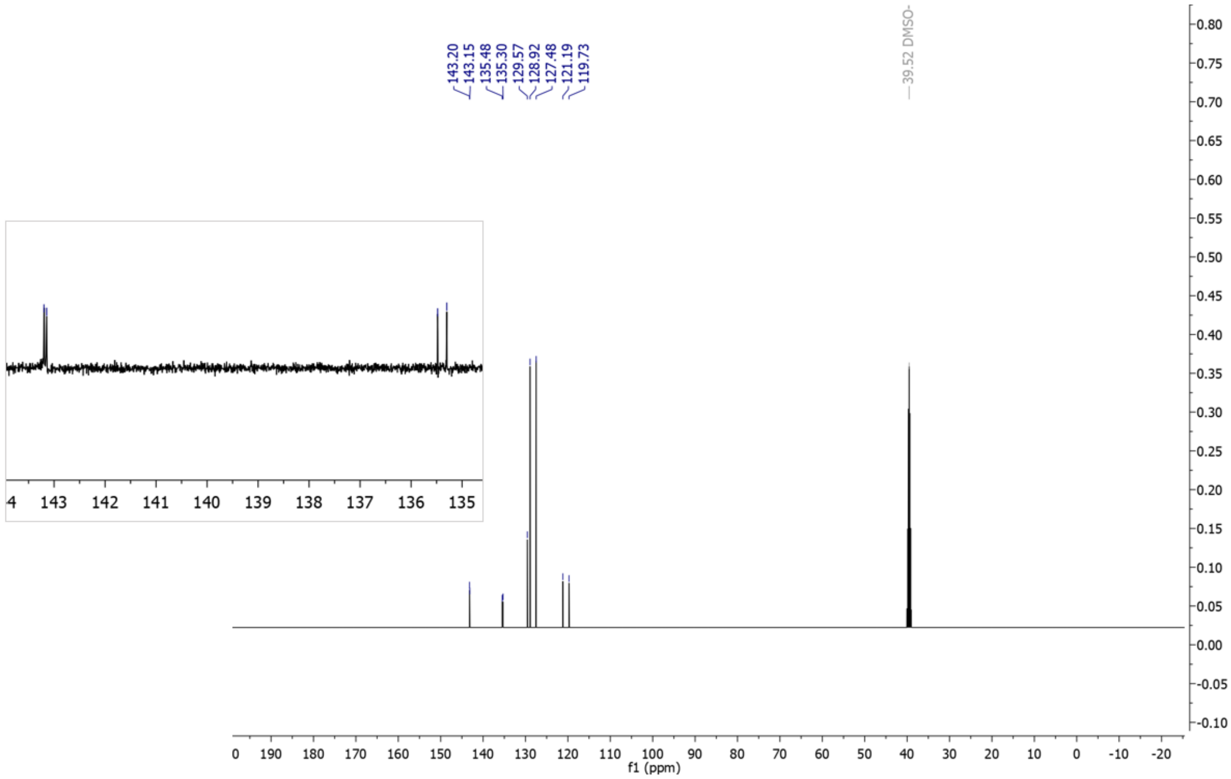

Figure S2. ^{13}C NMR spectrum of (E)-styrylphosphonic acid (DMSO- d_6, 126 MHz, 298 K)

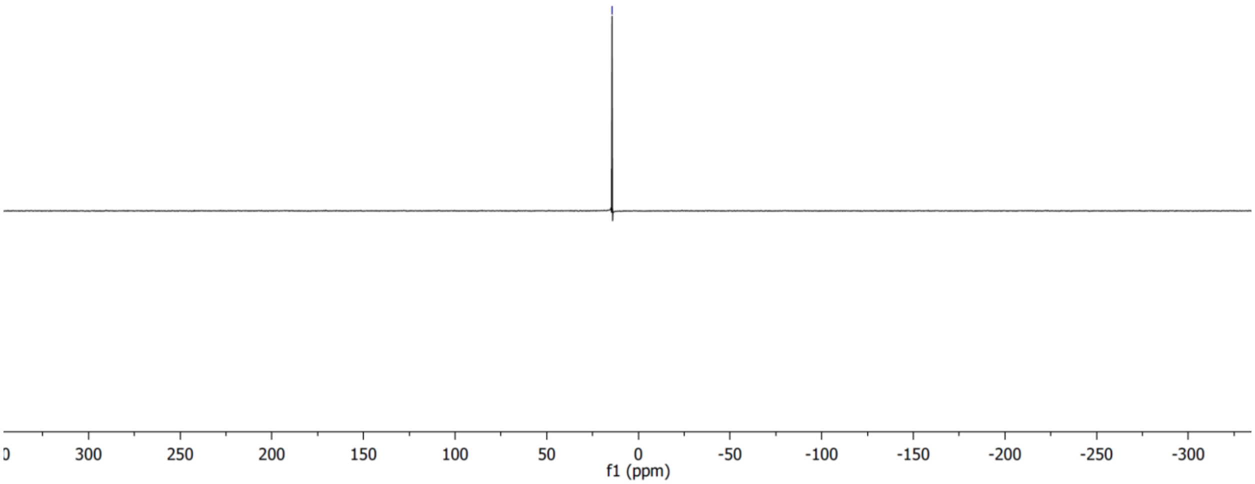

Figure S3. ^{31}P NMR spectrum of (E)-styrylphosphonic acid (DMSO-d_6, 202 MHz, 298 K)

(E)-(4-(Trifluoromethyl)styryl)phosphonic acid (CF_3Phenyl vinylphosphonic acid (2))

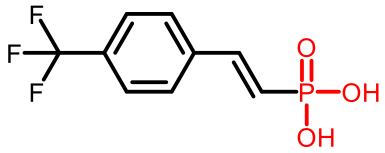

A 250 mL Schlenk flask with stir bar was oven dried. The Schlenk flask was charged with 4-bromo-benzotrifluoride (5.01 g, 22.2 mmol) and bis(tri-tert-butyl phosphine)palladium(0) (0.284 g, 0.555 mmol), followed by vacuum and N_2 refill cycles (3×). Vinyl phosphonic acid (VPA) (2.88 g, 26.6 mmol) was dissolved in 10 mL of anhydrous dioxane and gently bubbled with N_2 for 20 min. Anhydrous dioxane (150 mL) and the solution of VPA was added to the Schenk flask via syringe with stirring. N,N-Dicyclohexylmethylamine (14.2 mL, 66.6 mmol) was added to the reaction mixture dropwise via syringe. The reaction was stirred at 80°C for 20 h. The progress was monitored with thin layer chromatography (EtOAc/Hexanes 1:4). Upon reaction completion, mixture was cooled to room temperature. The product was extracted with EtOAc and washed with 5% HCl (3×). Organic phase was dried over $MgSO_4$ and filtered. Obtained organic phase was condensed to a few mL by rotary evaporator. The product was precipitated into DCM and the filtered precipitate (white flaky crystals) was dried overnight at 45°C under vacuum. Yield of 4.71 g (84%). ^{1}H NMR (500 MHz, DMSO-d_6) δ 9.59 (s, 2H), 7.83 (d, J = 14.1 Hz, 2H), 7.73 (s, 2H), 7.34 – 7.19 (m, 1H), 6.76 – 6.62 (m, 1H). ^{31}P NMR (202 MHz, DMSO-d_6) δ 12.99. ^{13}C NMR (126 MHz, DMSO-d_6) δ 141.26 (d, J = 6.1 Hz), 139.51, 139.34, 129.42, 129.17, 128.12, 125.68, 124.75, 123.32.

The purity of the compound was determined from ^{1}H, ^{13}C, and ^{31}P NMR. Integration of the ^{1}H NMR in addition to the single peak in the ^{31}P NMR and the absence of the signal attributed to the starting VPA indicated high compound purity (>99%). The doublet at 141.26 ppm in ^{13}C NMR is assigned to the carbon adjacent to phosphorus, the splitting is attributed to the ^{31}P-C coupling.

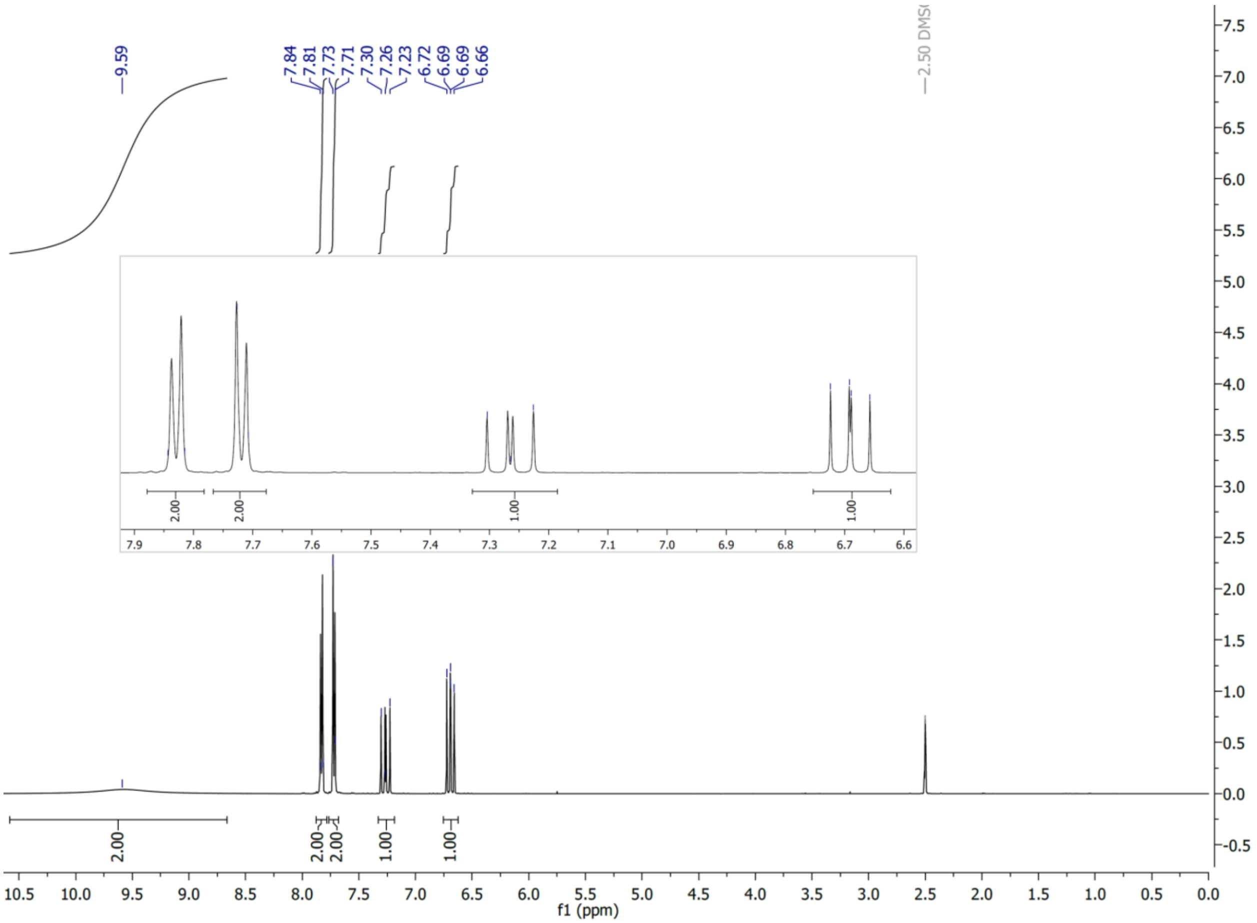

Figure S4. ^{1}H NMR spectrum of (E)-(4-(trifluoromethyl)styryl)phosphonic acid (DMSO- d_6 δ 2.50, 500 MHz, 298 K)

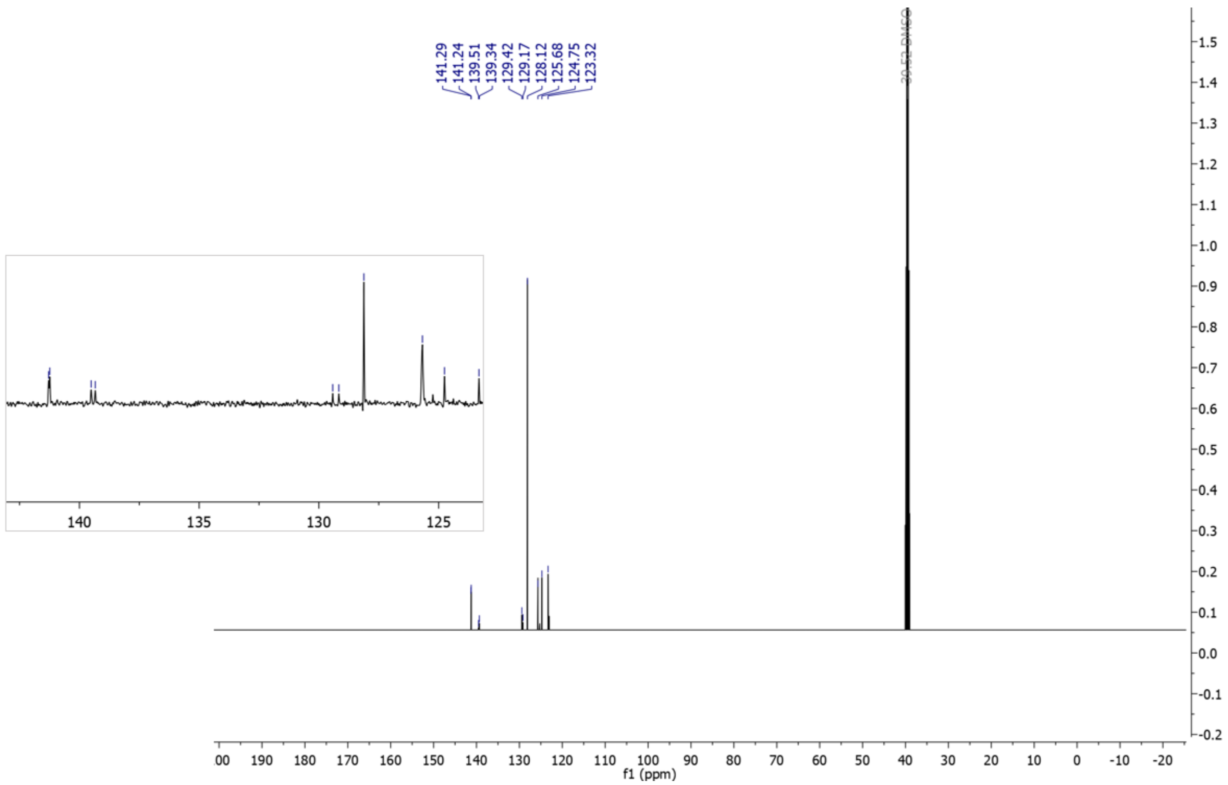

Figure S5. ^{13}C NMR spectrum of (E)-(4-(trifluoromethyl)styryl)phosphonic acid (DMSO- d_6, 126 MHz, 298 K)

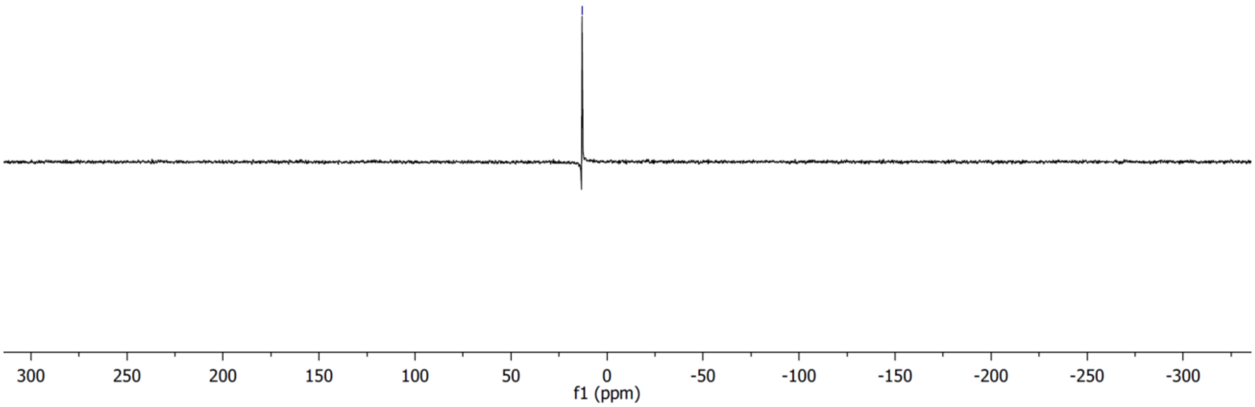

Figure S6. ^{31}P NMR spectrum of (E)-(4-(trifluoromethyl)styryl)phosphonic acid (DMSO-d_6, 202 MHz, 298 K)

(E)-(2-(Naphthalen-1-yl)vinyl)phosphonic acid (Naphthyl vinyl phosphonic acid (3))

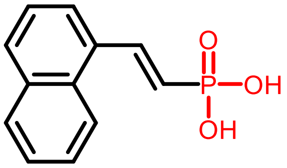

A 250 mL Schlenk flask with stir bar was oven dried. The Schlenk flask was charged with 1-bromonaphthalene (4 g, 19.32 mmol) and bis(tri-tert-butyl phosphine)palladium(0) (0.394 g, 0.770 mmol), followed by vacuum and N_2 refill cycles (3×). Vinyl phosphonic acid (VPA) (2.50 g, 23.2 mmol) was dissolved in 10 mL of anhydrous dioxane and gently bubbled with N_2 for 20 min. Anhydrous dioxane (175 mL) and the solution of VPA was added to the Schenk flask via syringe with stirring. N,N-Dicyclohexylmethylamine (8.22 mL, 38.64 mmol) was added to the reaction mixture dropwise via syringe. The reaction was stirred at 80°C for 20 h. The progress was monitored with thin layer chromatography (EtOAc/Hexanes 1:4). Upon reaction completion, mixture was cooled to room temperature. The product was extracted with EtOAc and washed with 5% HCl (3×). Organic phase was dried over $MgSO_4$ and filtered. Obtained organic phase was condensed to a few mL by rotary evaporator. The product was precipitated into DCM and filtered product (white crystals) was dried overnight at 45°C under vacuum. Yield of 3.499 g (77%). ^{1}H NMR (500 MHz, DMSO-d_6) δ 8.08 (s, 1H), 7.93 (d, J = 6.3 Hz, 3H), 7.83 (d, J = 9.4 Hz, 1H), 7.55 (d, J = 7.2 Hz, 2H), 7.40 – 7.30 (m, 1H), 6.66 (d, J = 16.7 Hz, 1H). ^{31}P NMR (202 MHz, DMSO-d_6) δ 13.73. ^{13}C NMR (126 MHz, DMSO-d_6) δ 143.18 (d, J = 6.1 Hz), 133.45, 133.06 (d, J = 2.2 Hz), 132.90, 128.50, 128.40, 127.71, 126.95, 126.69, 123.62, 121.64, 120.19.

The purity of the compound was determined from ^{1}H, ^{13}C, and ^{31}P NMR. Integration of the ^{1}H NMR in addition to the single peak in the ^{31}P NMR and the absence of the signal attributed to the starting VPA indicated high compound purity (>98%). The doublets at 143.18 and 133.06 ppm in ^{13}C NMR are assigned to the vinyl linkage carbons adjacent to phosphorus, the splitting is attributed to the ^{31}P-C coupling.

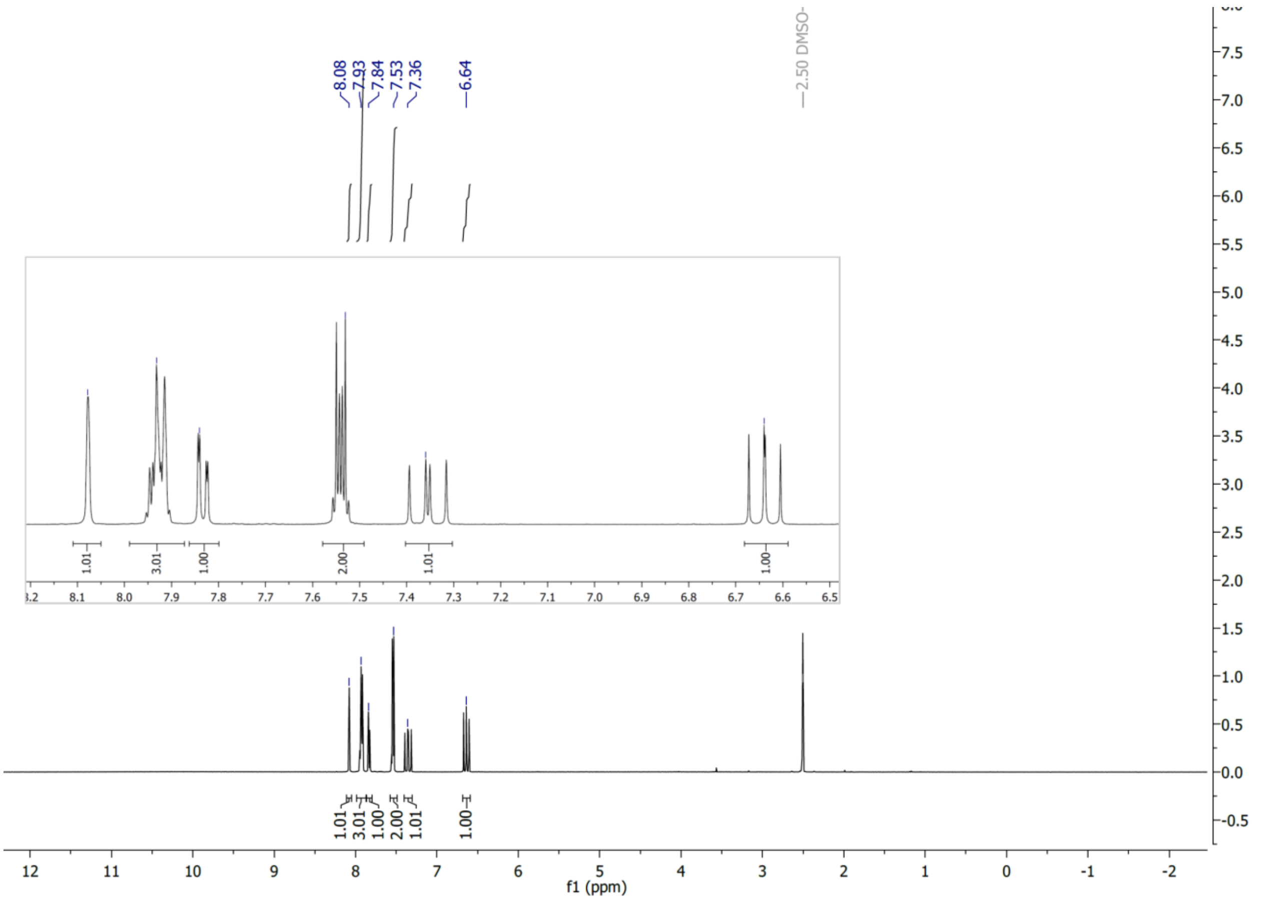

Figure S7. ^{1}H NMR spectrum of (E)-(2-(naphthalen-1-yl)vinyl)phosphonic acid (DMSO- d_6 δ 2.50, 500 MHz, 298 K)

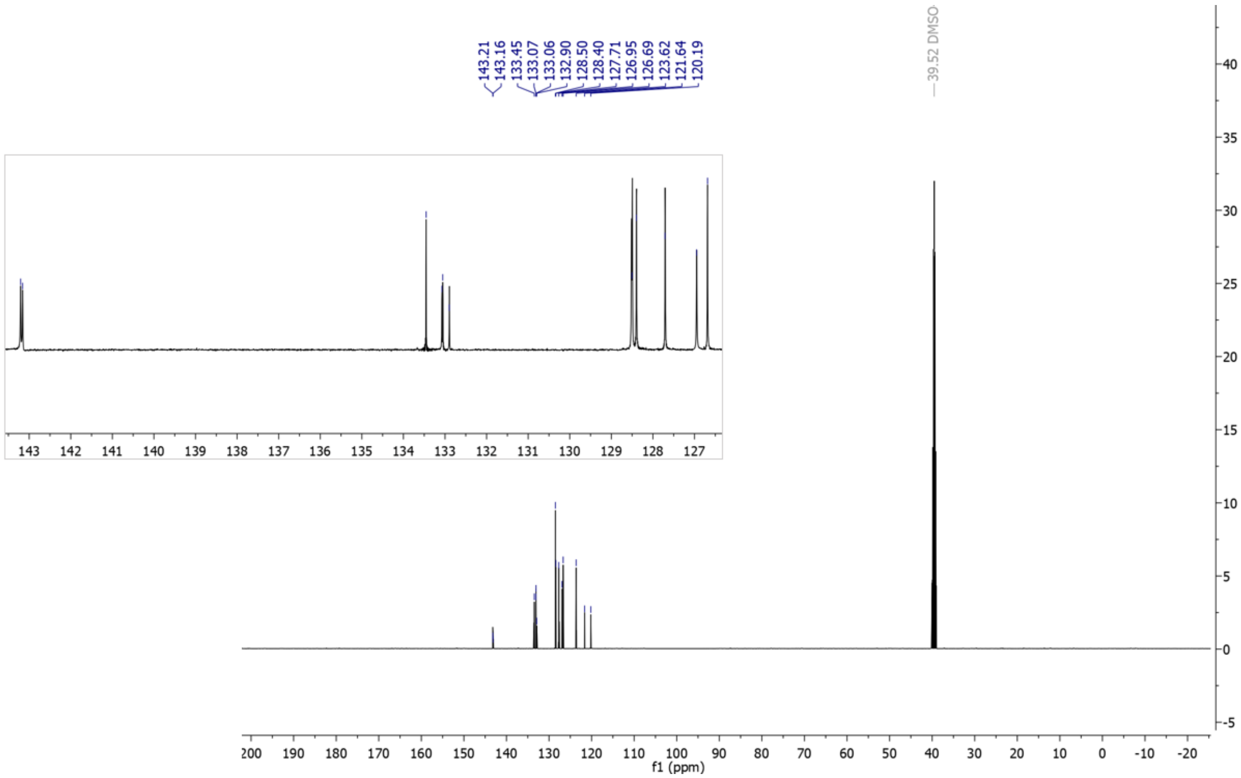

Figure S8. ^{13}C NMR spectrum of (E)-(2-(naphthalen-1-yl)vinyl)phosphonic acid (DMSO- d_6, 126 MHz, 298 K)

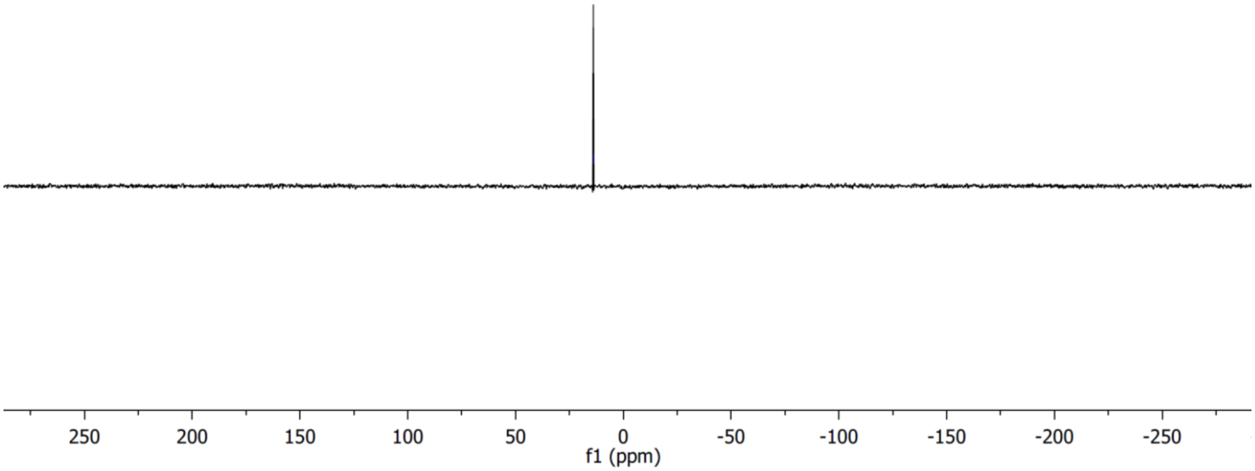

Figure S9. ^{31}P NMR spectrum of (E)-(2-(naphthalen-1-yl)vinyl)phosphonic acid (DMSO-d_6, 202 MHz, 298 K)

(E)-(2-(Anthracen-9-yl)vinyl)phosphonic acid (Anthracene vinylphosphonic acid (4))

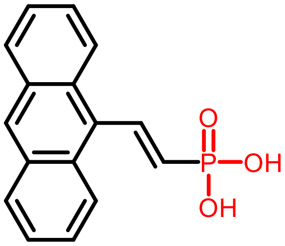

A 100 mL Schlenk flask with stir bar was oven dried. The Schlenk flask was charged with 9-bromoanthracene (1.20 g, 4.7 mmol) and bis(tri-tert-butyl phosphine)palladium(0) (0.096 g, 0.19 mmol), followed by vacuum and N_2 refill cycles (3×). Vinyl phosphonic acid (VPA) (0.61 g, 5.64 mmol) was dissolved in 5 mL of anhydrous dioxane and gently bubbled with N_2 for 20 min. Anhydrous dioxane (35 mL) and the solution of VPA was added to the Schenk flask via syringe with stirring. N,N-Dicyclohexylmethylamine (2.0 mL, 9.4 mmol) was added to the reaction mixture dropwise via syringe. The reaction was stirred at 80°C for 20 h. The progress was monitored with thin layer chromatography (EtOAc/Hexanes 1:4). Upon reaction completion, mixture was cooled to room temperature. The product was extracted with EtOAc and washed with 5% HCl (3×). Organic phase was dried over $MgSO_4$ and filtered. Obtained organic phase was condensed to a few mL by rotary evaporator. The product was precipitated into DCM and filtered product (bright yellow crystals) was dried overnight at 45°C under vacuum. Yield of 0.962 g (72%). ^{1}H NMR (500 MHz, DMSO-d_6) δ 8.61 (s, 1H), 8.17 (d, 2H), 8.11 (d, 1H), 8.04 (t, 1H), 7.56 (p, 4H), 6.31 (t, 1H). ^{31}P NMR (202 MHz, DMSO-d_6) δ 11.60. ^{13}C NMR (126 MHz, DMSO-d_6) δ 139.75 (d, J = 5.4 Hz), 131.61, 131.11, 130.91 (d, J = 7.2 Hz), 130.22, 128.81, 128.38, 127.29, 126.35, 125.53, 124.93.

The purity of the compound was determined from ^{1}H, ^{13}C, and ^{31}P NMR. Integration of the ^{1}H NMR in addition to the single peak in the ^{31}P NMR and the absence of the signal attributed to the starting VPA indicated high compound purity (>98%). The doublets at 139.75 and 130.91 ppm in ^{13}C NMR are assigned to the vinyl linkage carbons adjacent to phosphorus, the splitting is attributed to the ^{31}P-C coupling.

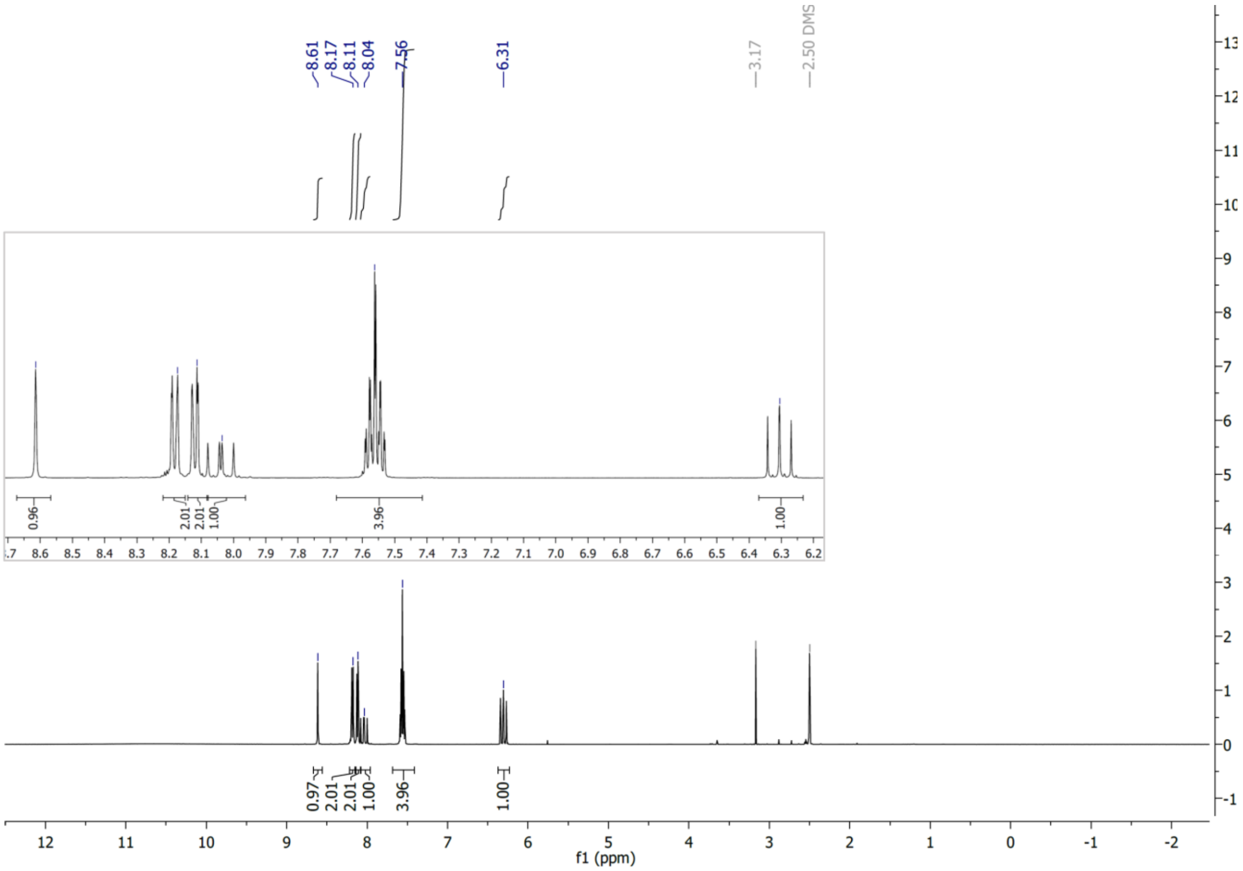

Figure S10. ^{1}H NMR spectrum of (E)-(2-(anthracen-9-yl)vinyl)phosphonic acid (DMSO- d_6 δ 2.50, 500 MHz, 298 K)

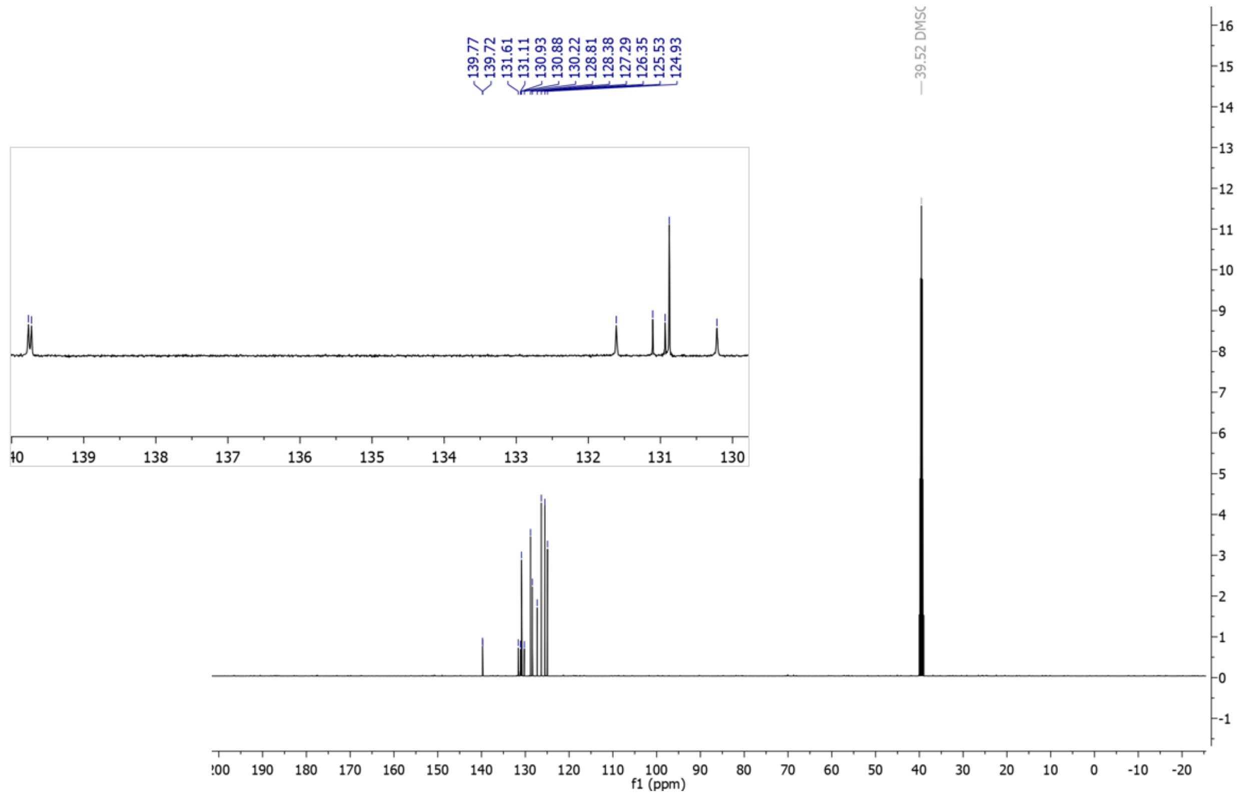

Figure S11. ^{13}C NMR spectrum of (E)-(2-(anthracen-9-yl)vinyl)phosphonic acid (DMSO- d_6, 126 MHz, 298 K)

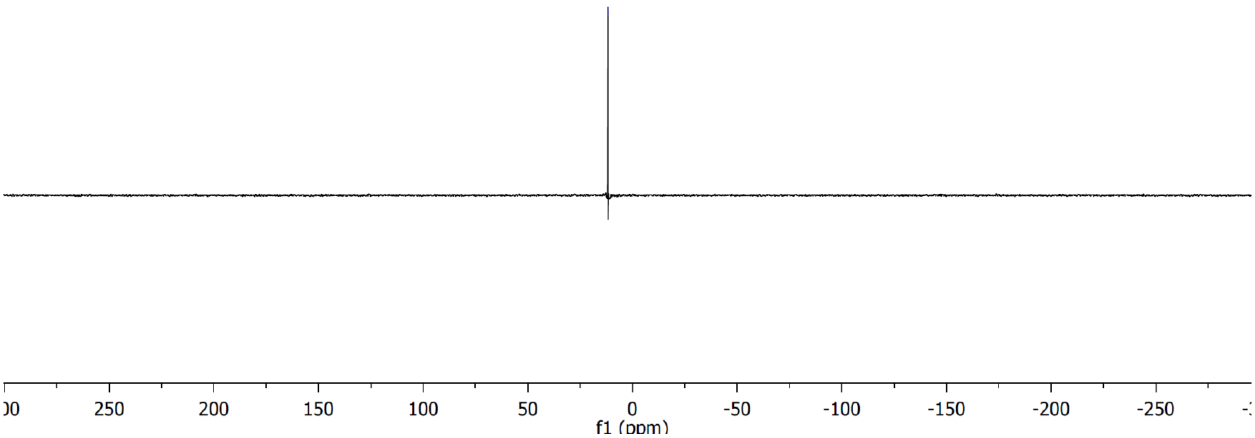

Figure S12. ^{31}P NMR spectrum of (E)-(2-(anthracen-9-yl)vinyl)phosphonic acid (DMSO-d_6, 202 MHz, 298 K)

(E)-(2-(Pyren-1-yl)vinyl)phosphonic acid (Pyrene vinylphosphonic acid (5))

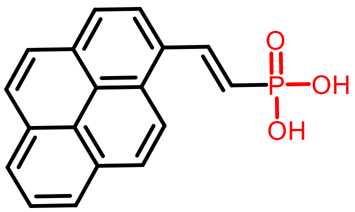

A 250 mL Schlenk flask with stir bar was oven dried. The Schlenk flask was charged with 2-bromopyrene (4 g, 14.22 mmol) and bis(tri-tert-butyl phosphine)palladium(0) (0.394 g, 0.770 mmol), followed by vacuum and N_2 refill cycles (3×). Vinyl phosphonic acid (VPA) (2.50 g, 23.2 mmol) was dissolved in 10 mL of anhydrous dioxane and gently bubbled with N_2 for 20 min. Anhydrous dioxane (175 mL) and the solution of VPA was added to the Schenk flask via syringe with stirring. N,N-Dicyclohexylmethylamine (8.22 mL, 38.64 mmol) was added to the reaction mixture dropwise via syringe. The reaction was stirred at 80°C for 20 h. The progress was monitored with thin layer chromatography (EtOAc/Hexanes 1:4). Upon reaction completion, mixture was cooled to room temperature. 500 mL of 5% HCl was added to the reaction mixture and mixed well. Solid was filtered and washed with MeOH, followed by filtration with celite plug. Organic phase was dried over $MgSO_4$ and filtered. Obtained organic phase was condensed to a few mL by rotary evaporator. The product was precipitated into DCM and filtered product (olive-green solid) was dried overnight at 45°C under vacuum. Yield of 3.12 g (52%). ^{1}H NMR (500 MHz, DMSO-d_6) δ 8.54 – 7.94 (m, 10H), 6.80 (s, 1H). ^{31}P NMR (202 MHz, DMSO-d_6) δ 12.52. ^{13}C NMR (126 MHz, DMSO-d_6) δ 139.23 (d, J = 6.6 Hz), 131.45, 130.85, 130.14, 129.12 (d, J = 7.2 Hz), 128.35, 128.18, 128.02, 127.29, 126.48, 125.89, 125.56, 125.26, 124.27, 123.98, 123.83, 122.83, 122.00.

The purity of the compound was determined from ^{1}H, ^{13}C, and ^{31}P NMR. Integration of the ^{1}H NMR in addition to the single peak in the ^{31}P NMR and the absence of the signal attributed to the starting VPA indicated high compound purity (>97%). The doublets at 139.23 and 129.12 ppm in ^{13}C NMR are assigned to the vinyl linkage carbons adjacent to phosphorus, the splitting is attributed to the ^{31}P-C coupling.

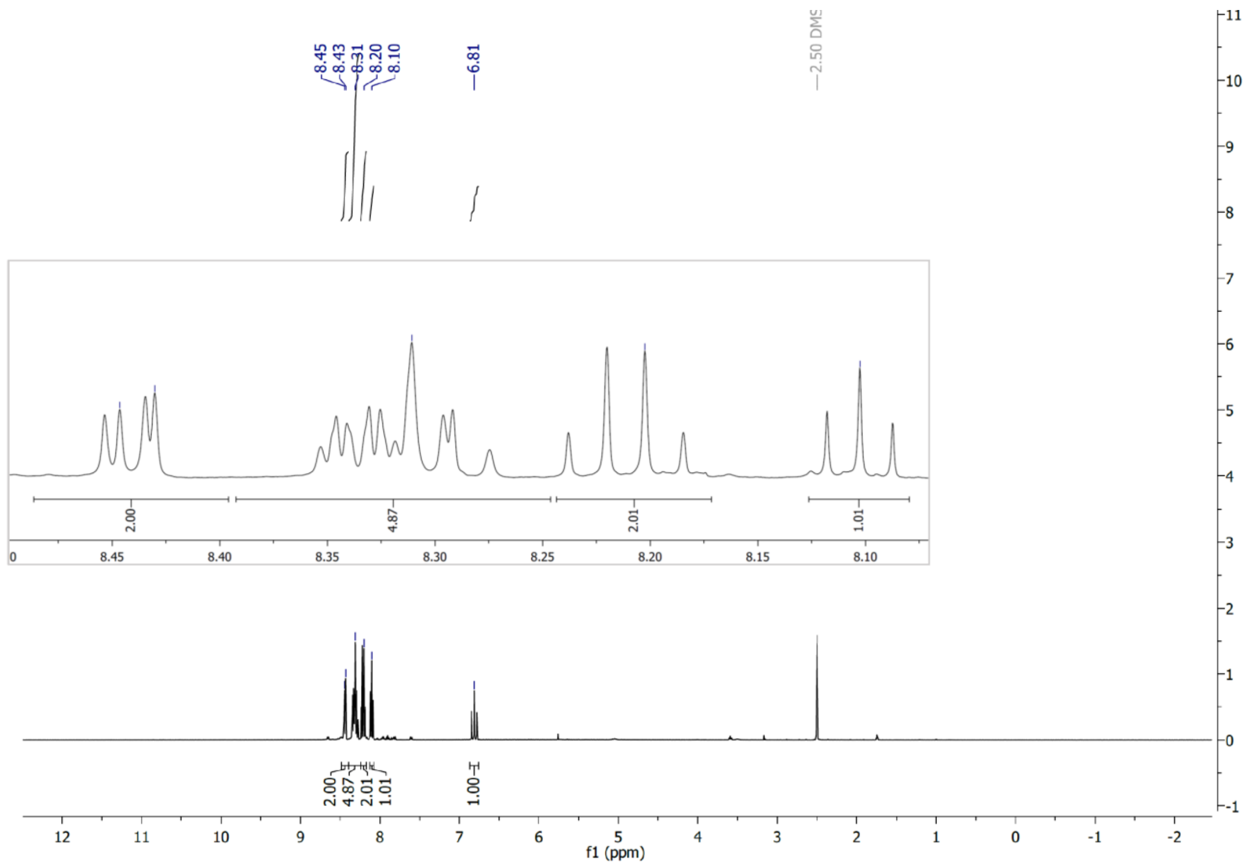

Figure S13. ^{1}H NMR spectrum of (E)-(2-(pyren-1-yl)vinyl)phosphonic acid (DMSO- d_6 δ 2.50, 500 MHz, 298 K)

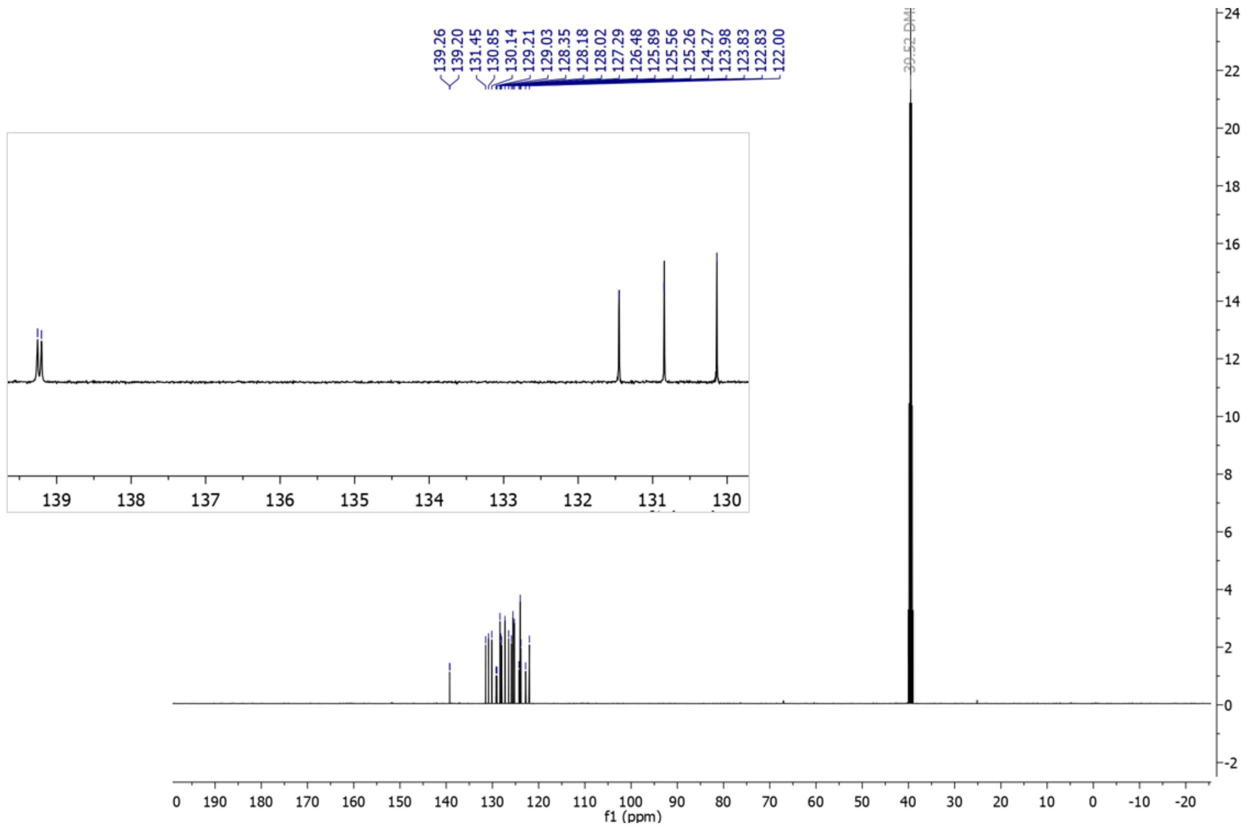

Figure S14. ^{13}C NMR spectrum of (E)-(2-(pyren-1-yl)vinyl)phosphonic acid (DMSO- d_6, 126 MHz, 298 K)

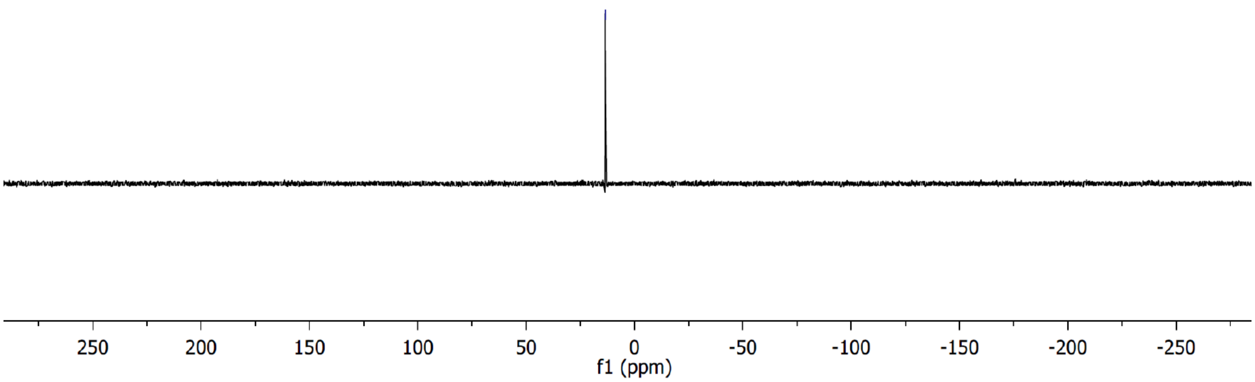

Figure S15. ^{31}P NMR spectrum of (E)-(2-(pyren-1-yl)vinyl)phosphonic acid (DMSO-d_6, 202 MHz, 298 K)

((E)-4-((E)-phenyldiazenyl)styryl)phosphonic acid (Azobenzene vinylphosphonic acid (6))

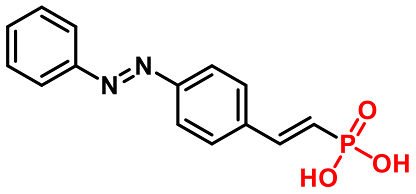

A 100 mL Schlenk flask with stir bar was oven dried. The Schlenk flask was charged with 4-bromoazobenzene (1.0 g, 3.8 mmol) and bis(tri-tert-butyl phosphine)palladium(0) (0.049 g, 0.096 mmol), followed by vacuum and N_2 refill cycles (3×). Vinyl phosphonic acid (VPA) (0.41 g, 3.8 mmol) was dissolved in 5 mL of anhydrous dioxane and gently bubbled with N_2 for 20 min. Anhydrous dioxane (35 mL) and the solution of VPA was added to the Schenk flask via syringe with stirring. N,N-Dicyclohexylmethylamine (1.6 mL, 7.6 mmol) was added to the reaction mixture dropwise via syringe. The reaction was stirred at 80°C for 20 h. The progress was monitored with thin layer chromatography (EtOAc/Hexanes 1:4). Upon reaction completion, mixture was cooled to room temperature. The product was extracted with EtOAc and washed with 5% HCl (3×). Organic phase was dried over $MgSO_4$ and filtered. Obtained organic phase was condensed to a few mL by rotary evaporator. The product was precipitated into DCM and filtered product (bright yellow crystals) was dried overnight at 45°C under vacuum. Yield of 0.581 g (53%) ^{1}H NMR (500 MHz, DMSO-d_6) δ 7.90 (d, J = 8.1 Hz, 4H), 7.84 (d, J = 8.3 Hz, 2H), 7.60 (d, J = 7.2 Hz, 3H), 7.28 (t, J = 21.6, 17.5 Hz, 1H), 6.67 (t, J = 16.7 Hz, 1H). ^{31}P NMR (202 MHz, DMSO-d_6) δ 13.37. ^{13}C NMR (126 MHz, DMSO-d_6) δ 152.12, 152.01, 141.94, 141.69 (d, J = 9.0 Hz), 138.34 (d, J = 22.0 Hz), 131.85, 129.55 (d, J = 18.2 Hz), 128.61 (d, J = 23.1 Hz), 127.99 (d, J = 17.0 Hz), 123.40 – 122.90 (m), 122.76, 122.63.

The purity of the compound was determined from ^{1}H, ^{13}C, and ^{31}P NMR. Integration of the ^{1}H NMR in addition to the single peak in the ^{31}P NMR and the absence of the signal attributed to the starting VPA indicated high compound purity (>98%). Due to the solubility limitations, we were unable to saturate the NMR sample to obtain high intensity signal spectrum. In addition, ^{13}C NMR was collected sometime after the sample was prepared, which could lead to the presence of E and Z azobenzene isomers that were not detected while collecting ^{1}H and ^{31}P NMR.

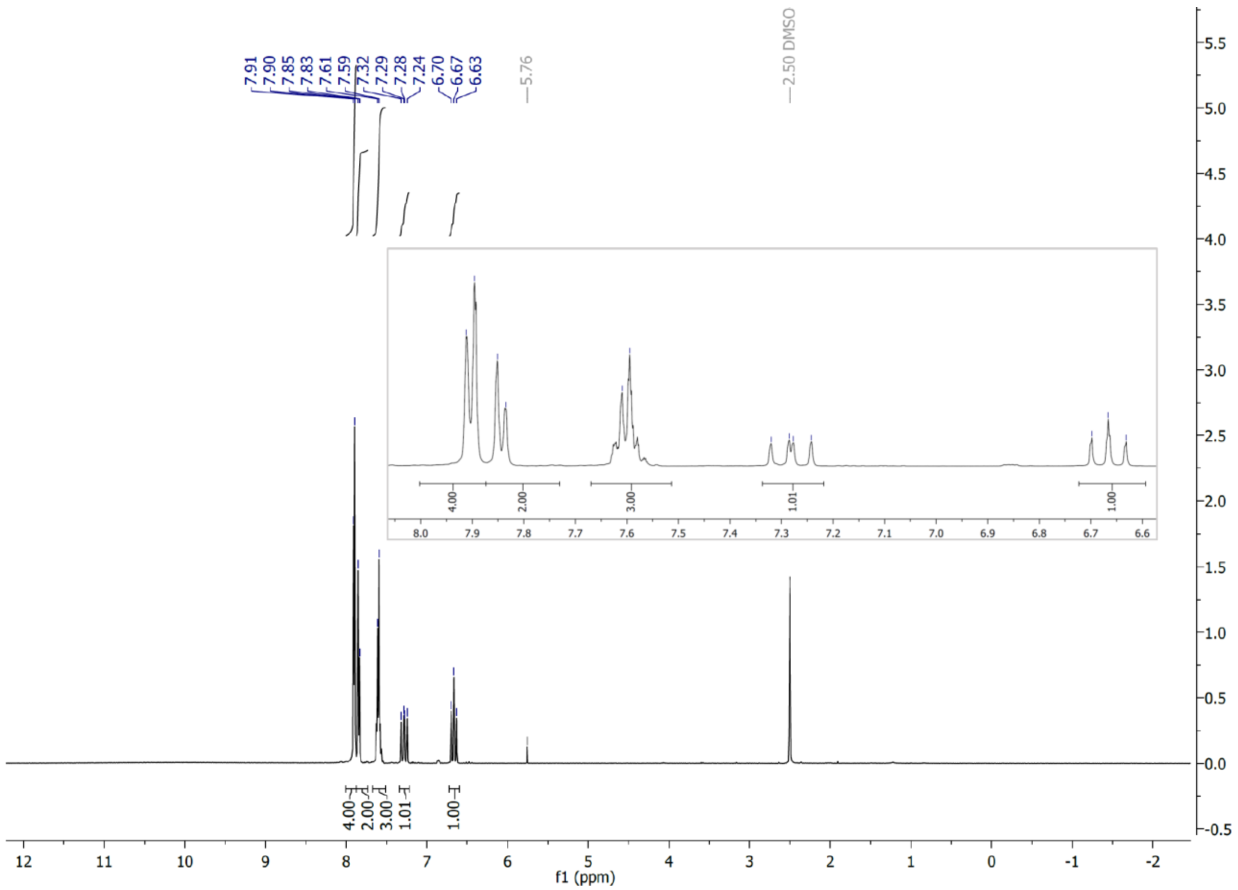

Figure S16. ^{1}H NMR spectrum of ((E)-4-((E)-phenyldiazenyl)styryl)phosphonic acid (DMSO-d_6 δ 2.50, 500 MHz, 298 K)

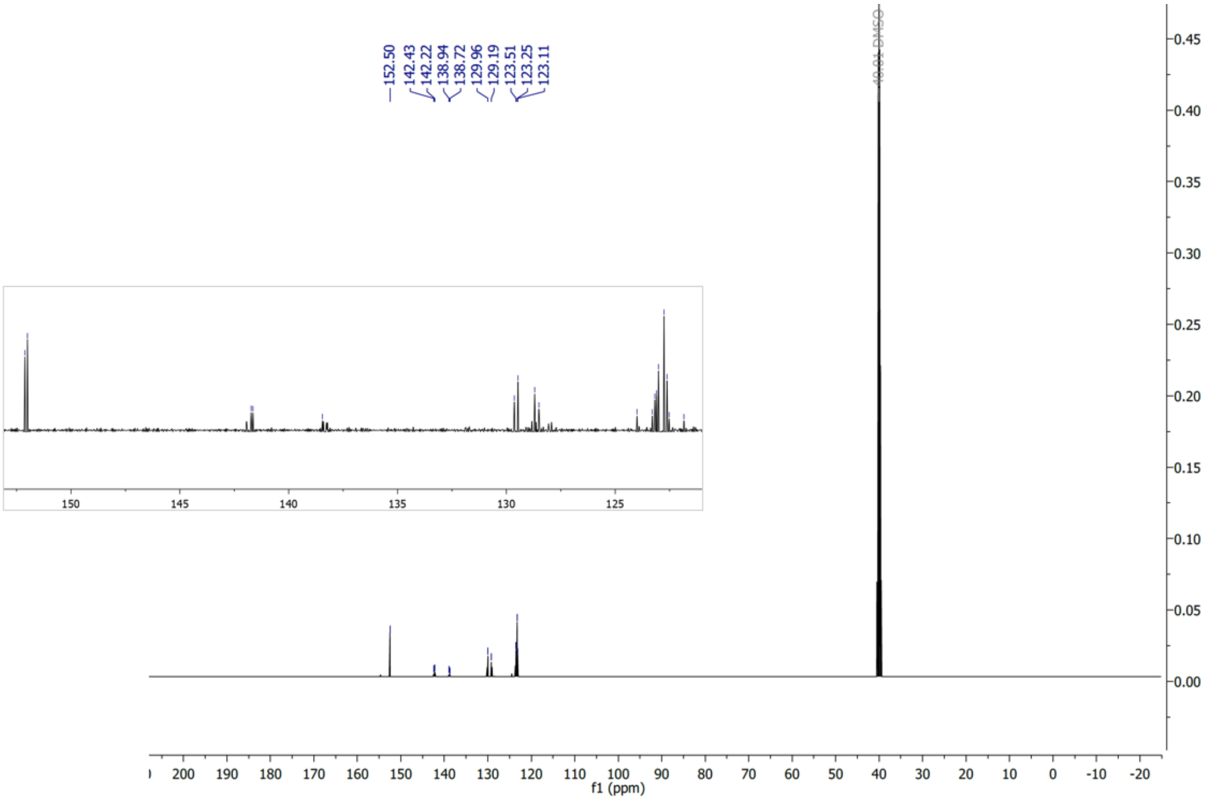

Figure S17. ^{13}C NMR spectrum of ((E)-4-((E)-phenyldiazenyl)styryl)phosphonic acid (DMSO-*d*$_6$, 126 MHz, 298 K)

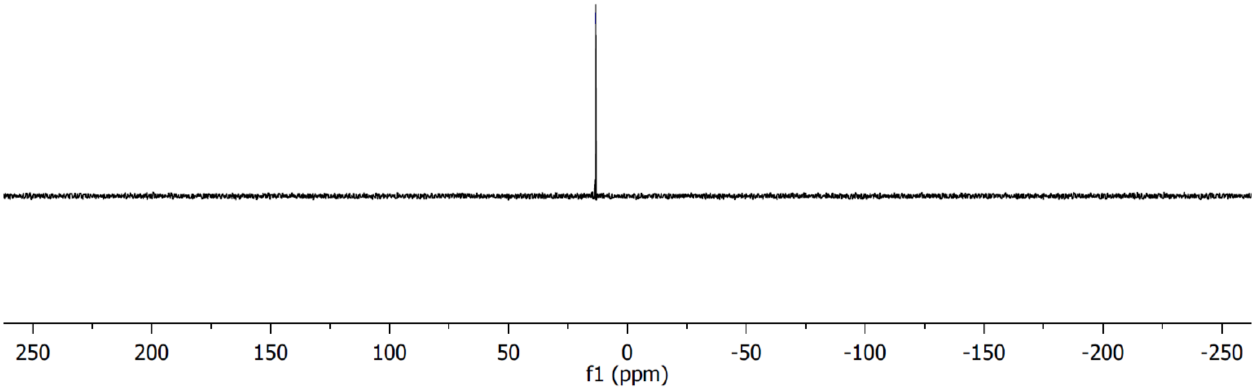

Figure S18. ^{31}P NMR spectrum of ((E)-4-((E)-phenyldiazenyl)styryl)phosphonic acid (DMSO-*d*$_6$, 202 MHz, 298 K)

((E)-4-((E)-styryl)styryl)phosphonic acid (Stilbene vinylphosphonic acid (7))

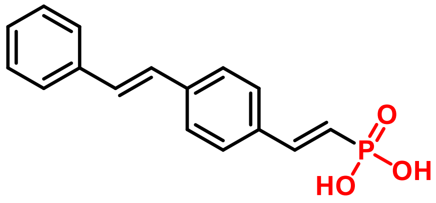

A 100 mL Schlenk flask with stir bar was oven dried. The Schlenk flask was charged with 4-bromostilbene (0.80 g, 3.1 mmol) and bis(tri-tert-butyl phosphine)palladium(0) (0.040 g, 0.078 mmol), followed by vacuum and N_2 refill cycles (3×). Vinyl phosphonic acid (VPA) (0.40 g, 3.7 mmol) was dissolved in 5 mL of anhydrous acetonitrile and gently bubbled with N_2 for 20 min. Anhydrous acetonitrile (35 mL) and the solution of VPA was added to the Schenk flask via syringe with stirring. N,N-Dicyclohexylmethylamine (1.3 mL, 6.2 mmol) was added to the reaction mixture dropwise via syringe. The reaction was stirred at 70°C for 20 h. The progress was monitored with thin layer chromatography (EtOAc/Hexanes 1:4). Upon reaction completion, mixture was cooled to room temperature. The product was extracted with EtOAc and washed with 5% HCl (3×). Organic phase was dried over $MgSO_4$ and filtered. Obtained organic phase was condensed to a few mL by rotary evaporator. The product was precipitated into DCM and filtered product (bright white crystals) was dried overnight at 45°C under vacuum. Yield of 0.615 g (69%) ^{1}H NMR (500 MHz, DMSO-d_6) δ 7.62 (s, 6H), 7.39 (s, 2H), 7.29 (s, 3H), 7.18 (s, 1H), 6.51 (s, 1H). ^{31}P NMR (202 MHz, DMSO-d_6) δ 14.01. ^{13}C NMR (126 MHz, DMSO-d_6) δ 142.41, 138.05, 136.82, 134.55 (d, J = 21.5 Hz), 129.19, 128.61, 127.70, 126.79, 126.51, 120.94, 119.48.

The purity of the compound was determined from ^{1}H, ^{13}C, and ^{31}P NMR. Integration of the ^{1}H NMR in addition to the single peak in the ^{31}P NMR and the absence of the signal attributed to the starting VPA indicated high compound purity (>98%). The doublet at 120.58 ppm in ^{13}C NMR is assigned to the β-carbon, the splitting is attributed to the ^{31}P-C coupling.

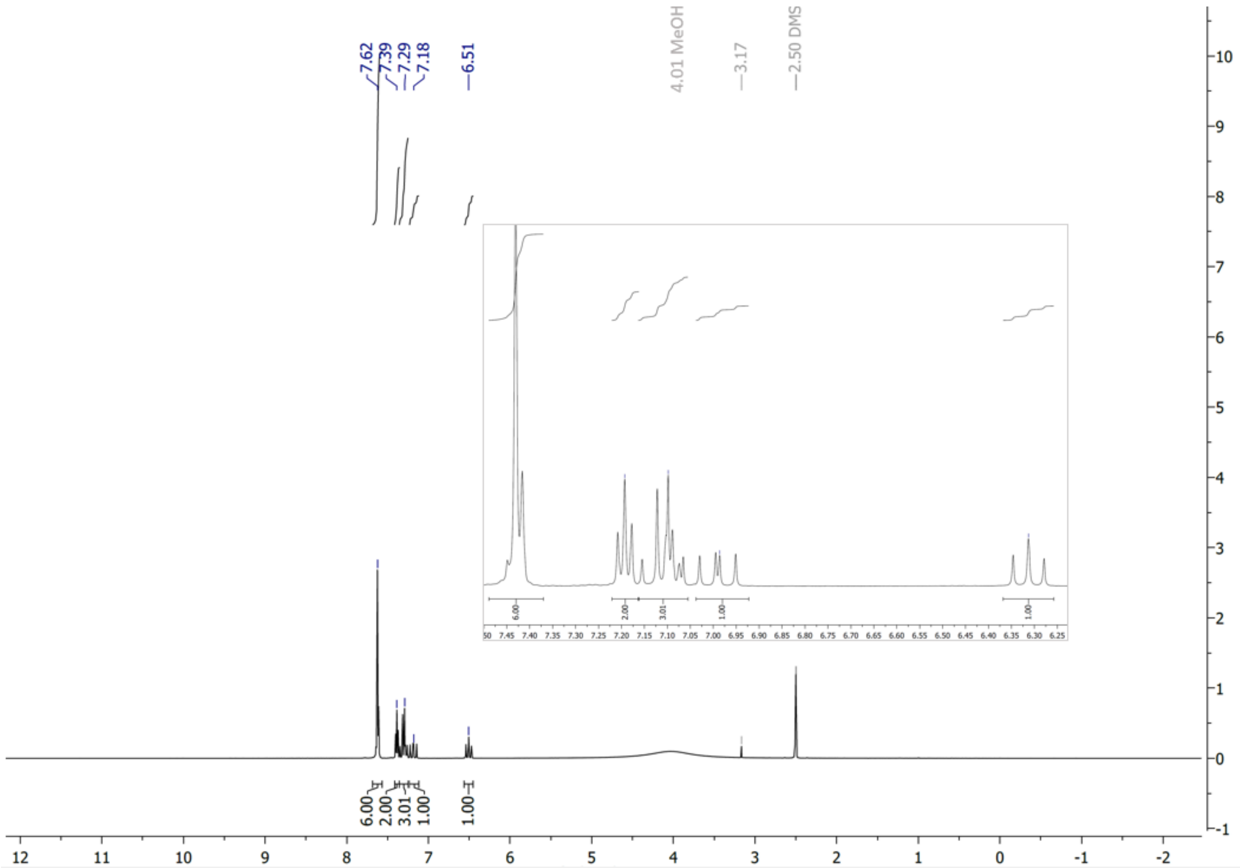

Figure S19. ^{1}H NMR spectrum of ((E)-4-((E)-styryl)styryl)phosphonic acid (DMSO-*d*$_6$ δ 2.50, 500 MHz, 298 K)

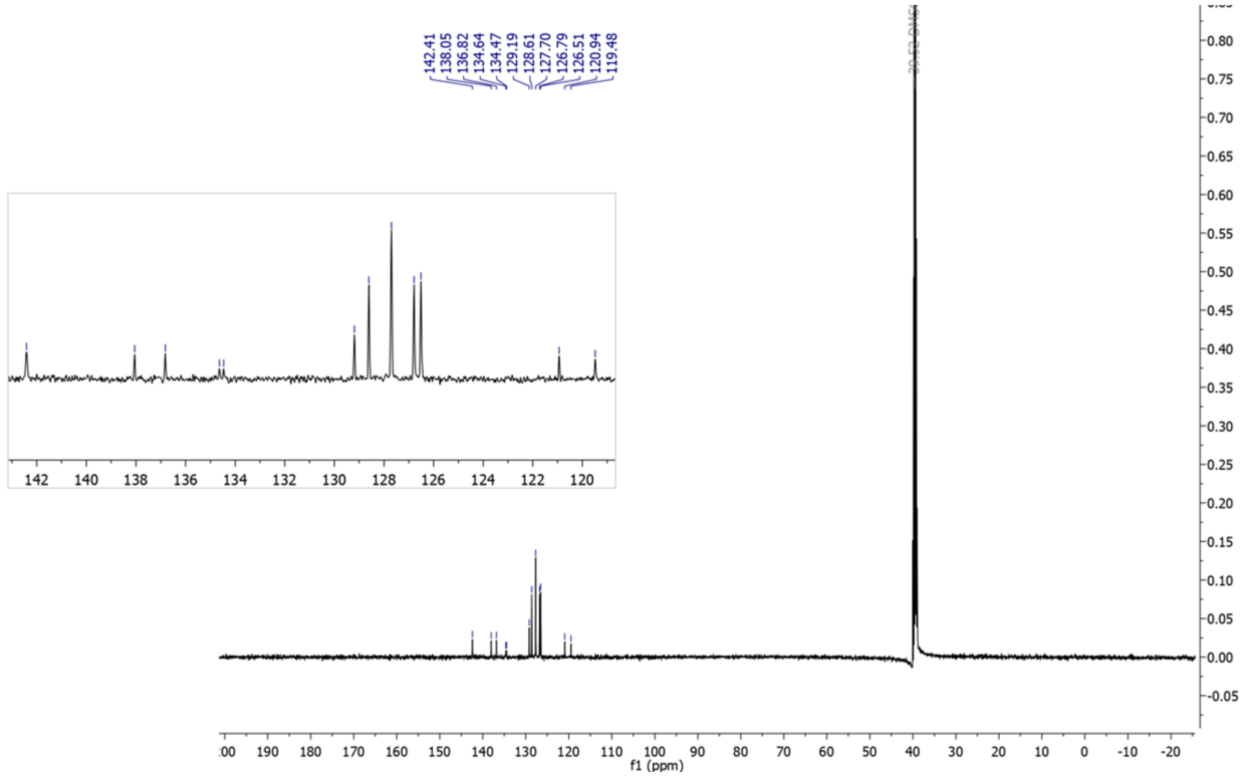

Figure S20. ^{13}C NMR spectrum of ((E)-4-((E)-styryl)styryl)phosphonic acid (DMSO- d_6, 126 MHz, 298 K)

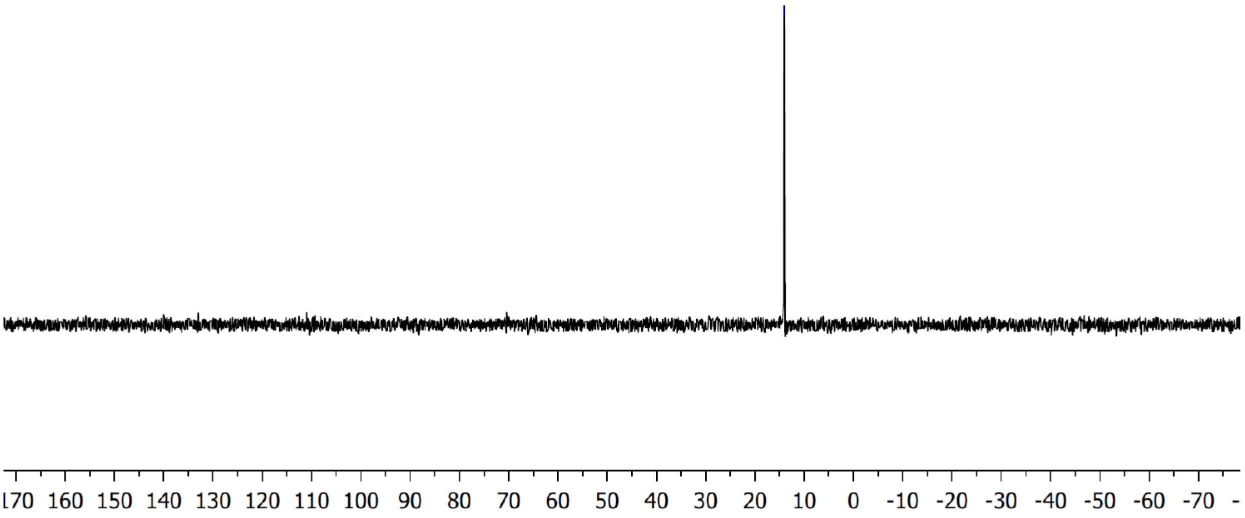

Figure S21. ^{31}P NMR spectrum of ((E)-4-((E)-styryl)styryl)phosphonic acid (DMSO-d_6, 202 MHz, 298 K)

(E)-(2-(9-(2-ethylhexyl)-9H-carbazol-2-yl)vinyl)phosphonic acid (2-ethylhexyl carbazole vinylphosphonic acid (8))

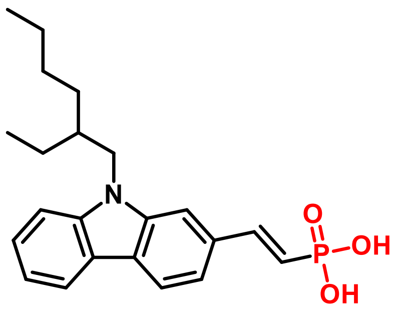

A 250 mL Schlenk flask with stir bar was oven dried. The Schlenk flask was charged with 2-bromo-9-(2-ethylhexyl)-9H-carbazole (4.01 g, 11.2 mmol) and bis(tri-tert-butyl phosphine)palladium(0) (0.286 g, 0.560 mmol), followed by vacuum and N_2 refill cycles (3×). Vinyl phosphonic acid (VPA) (1.45 g, 13.4 mmol) was dissolved in 10 mL of anhydrous dioxane and gently bubbled with N_2 for 20 min. Anhydrous dioxane (175 mL) and the solution of VPA was added to the Schenk flask via syringe with stirring. N,N-Dicyclohexylmethylamine (7.15 mL, 33.6 mmol) was added to the reaction mixture dropwise via syringe. The reaction was stirred at 80°C for 20 h. The progress was monitored with thin layer chromatography (EtOAc/Hexanes 1:4). Upon reaction completion, mixture was cooled to room temperature. 500 mL of 5% HCl was added to the reaction mixture and mixed well. Solid was filtered and washed with MeOH, followed by filtration with celite plug. Organic phase was dried over $MgSO_4$ and filtered. Obtained organic phase was condensed to a few mL by rotary evaporator. The product was precipitated into DCM and filtered product (pale blue solid) was dried overnight at 45°C under vacuum. Yield of 2.34 g (54%) ^{1}H NMR (500 MHz, DMSO-d_6) δ 8.14 (s, 2H), 7.84 (s, 1H), 7.57 – 7.32 (m, 4H), 7.20 (s, 1H), 6.62 (s, 1H), 4.29 (s, 2H), 1.23 (d, J = 76.8 Hz, 8H), 0.85 (s, 3H), 0.77 (s, 3H). ^{31}P NMR (202 MHz, DMSO-d_6) δ 14.51. ^{13}C NMR (126 MHz, DMSO-d_6) δ 144.18 (d, J = 7.9 Hz), 141.12, 140.76, 133.00 (d, J = 23.2 Hz), 126.19 (d, J = 17.9 Hz), 123.02, 121.79, 121.36, 120.51, 119.85, 119.12, 118.17 (d, J = 18.8 Hz), 109.60, 108.87 (d, J = 22.2 Hz), 46.44, 38.59, 30.08, 27.98, 23.62, 22.56, 13.90, 10.61.

The purity of the compound was determined from ^{1}H, ^{13}C, and ^{31}P NMR. Integration of the ^{1}H NMR in addition to the single peak in the ^{31}P NMR and the absence of the signal attributed to the starting VPA indicated high compound purity (>96%). The doublet at 144.18 ppm in ^{13}C NMR is assigned to the carbon adjacent to phosphorus, the splitting is attributed to the ^{31}P-C coupling.

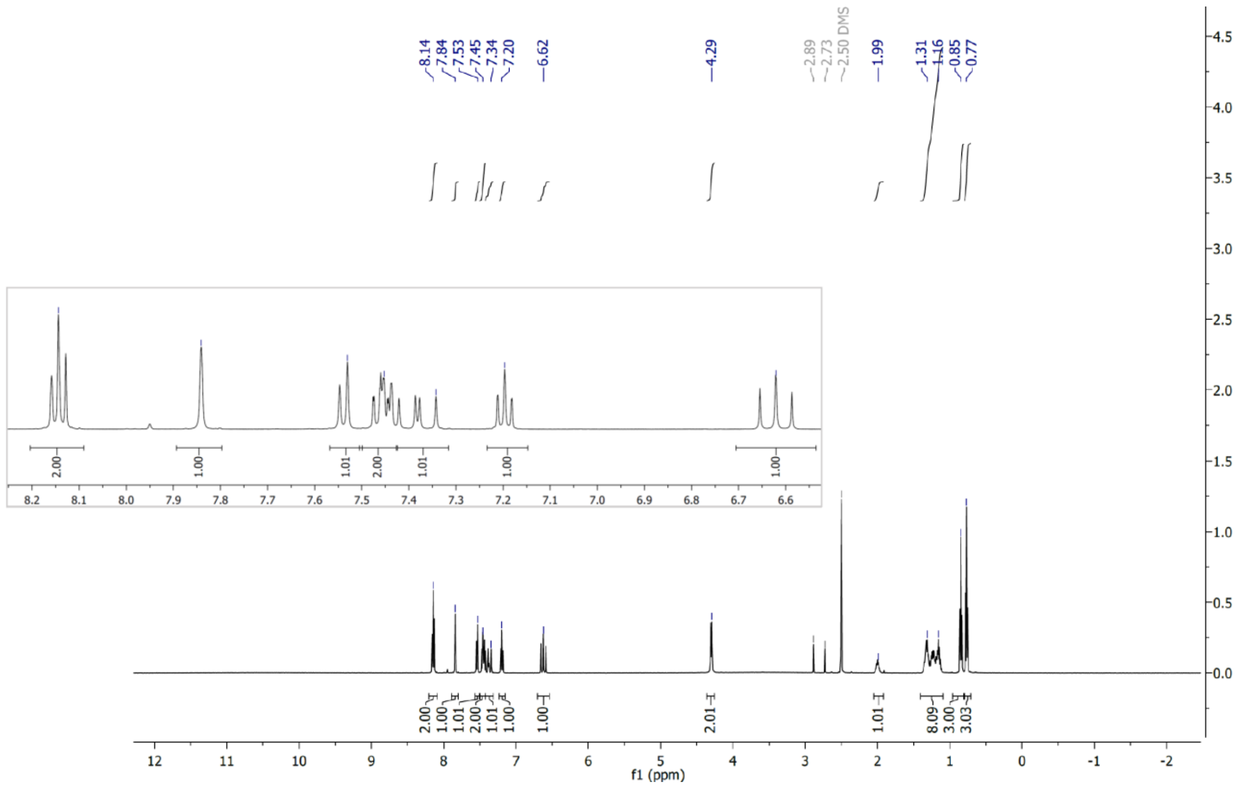

Figure S22. ^{1}H NMR spectrum of (E)-(2-(9-(2-ethylhexyl)-9H-carbazol-2-yl)vinyl)phosphonic acid (DMSO-d_6 δ 2.50, 500 MHz, 298 K)

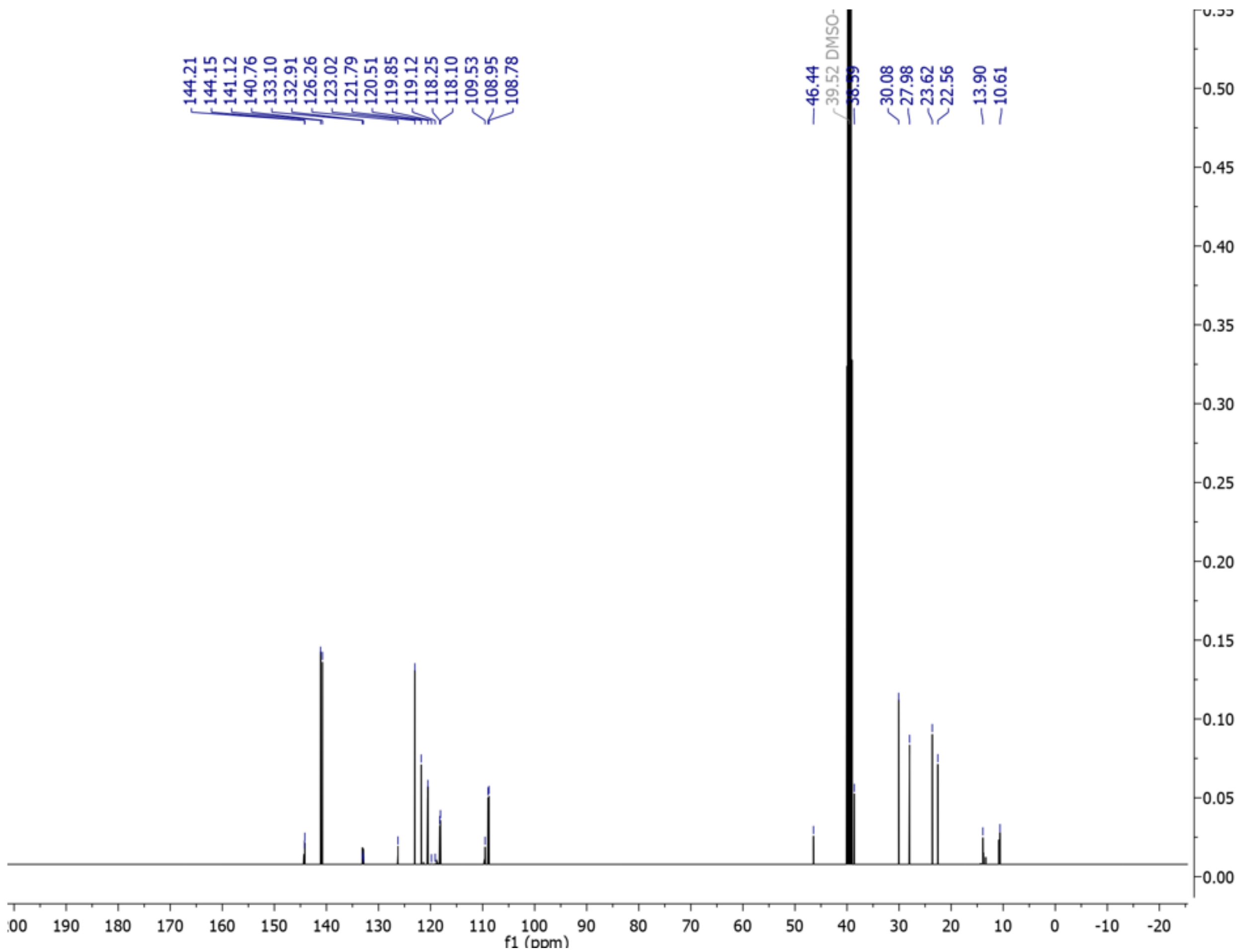

Figure S23. ^{13}C NMR spectrum of (E)-(2-(9-(2-ethylhexyl)-9H-carbazol-2-yl)vinyl)phosphonic acid (DMSO- d_6, 126 MHz, 298 K)

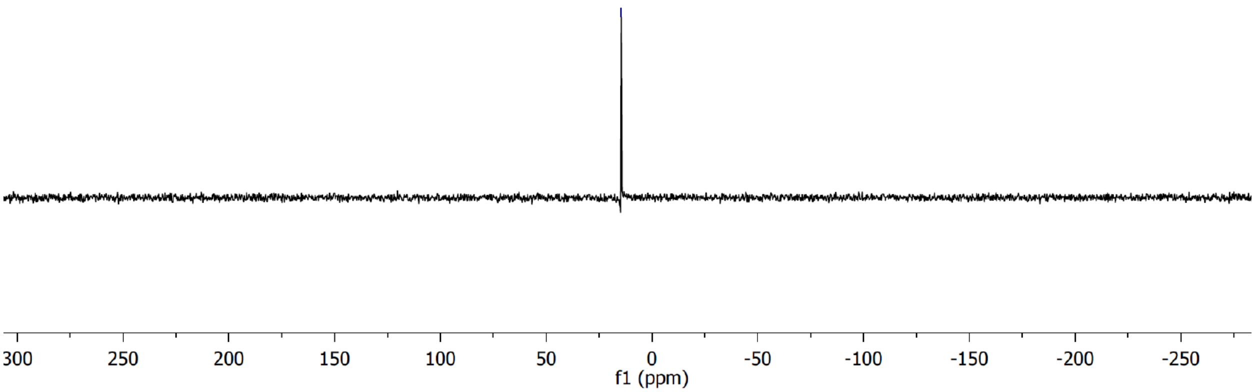

Figure S24. ^{31}P NMR spectrum of (E)-(2-(9-(2-ethylhexyl)-9H-carbazol-2-yl)vinyl)phosphonic acid (DMSO-d_6, 202 MHz, 298 K)

(E)-(2-(9,9-dimethyl-9H-fluoren-2-yl)vinyl)phosphonic acid (Dimethyl fluorene vinylphosphonic acid (9))

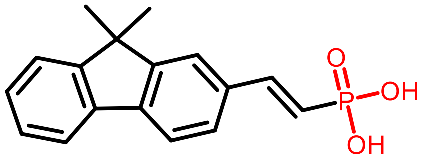

A 250 mL Schlenk flask with stir bar was oven dried. The Schlenk flask was charged with 2-bromo-9,9-dimethyl-9H-fluorene (4 g, 19.32 mmol) and bis(tri-tert-butyl phosphine)palladium(0) (0.394 g, 0.770 mmol), followed by vacuum and N_2 refill cycles (3×). Vinyl phosphonic acid (VPA) (2.50 g, 23.2 mmol) was dissolved in 10 mL of anhydrous dioxane and gently bubbled with N_2 for 20 min. Anhydrous dioxane (175 mL) and the solution of VPA was added to the Schenk flask via syringe with stirring. N,N-Dicyclohexylmethylamine (8.22 mL, 38.64 mmol) was added to the reaction mixture dropwise via syringe. The reaction was stirred at 80°C for 20 h. The progress was monitored with thin layer chromatography (EtOAc/Hexanes 1:4). Upon reaction completion, mixture was cooled to room temperature. 500 mL of 5% HCl was added to the reaction mixture and mixed well. The product was extracted with EtOAc and washed with 5% HCl (3×). Organic phase was dried over $MgSO_4$ and filtered. Obtained organic phase was condensed to a few mL by rotary evaporator. The product was precipitated into DCM and filtered product (light grey solid) was dried overnight at 45°C under vacuum. Yield of 3.42 g (59%). ^{1}H NMR (500 MHz, DMSO-d_6) δ 7.88 (s, 2H), 7.86 – 7.78 (m, 2H), 7.54 (s, 1H), 7.39 – 7.30 (m, 2H), 7.26 (dd, 1H), 6.59 (t, 1H), 1.45 (s, 16H). ^{31}P NMR (202 MHz, DMSO-d_6) δ 14.16. ^{13}C NMR (126 MHz, DMSO-d_6) δ 153.94 (d, J = 9.6 Hz), 143.47, 139.99, 138.00, 134.73, 134.55, 127.85, 127.38, 127.19, 122.89, 121.47, 120.52, 120.46, 119.03, 46.51, 26.74.

The purity of the compound was determined from ^{1}H, ^{13}C, and ^{31}P NMR. Integration of the ^{1}H NMR in addition to the single peak in the ^{31}P NMR and the absence of the signal attributed to the starting VPA indicated high compound purity (>98%). The doublet at 153.94 ppm in ^{13}C NMR is assigned to the carbon adjacent to phosphorus, the splitting is attributed to the ^{31}P-C coupling.

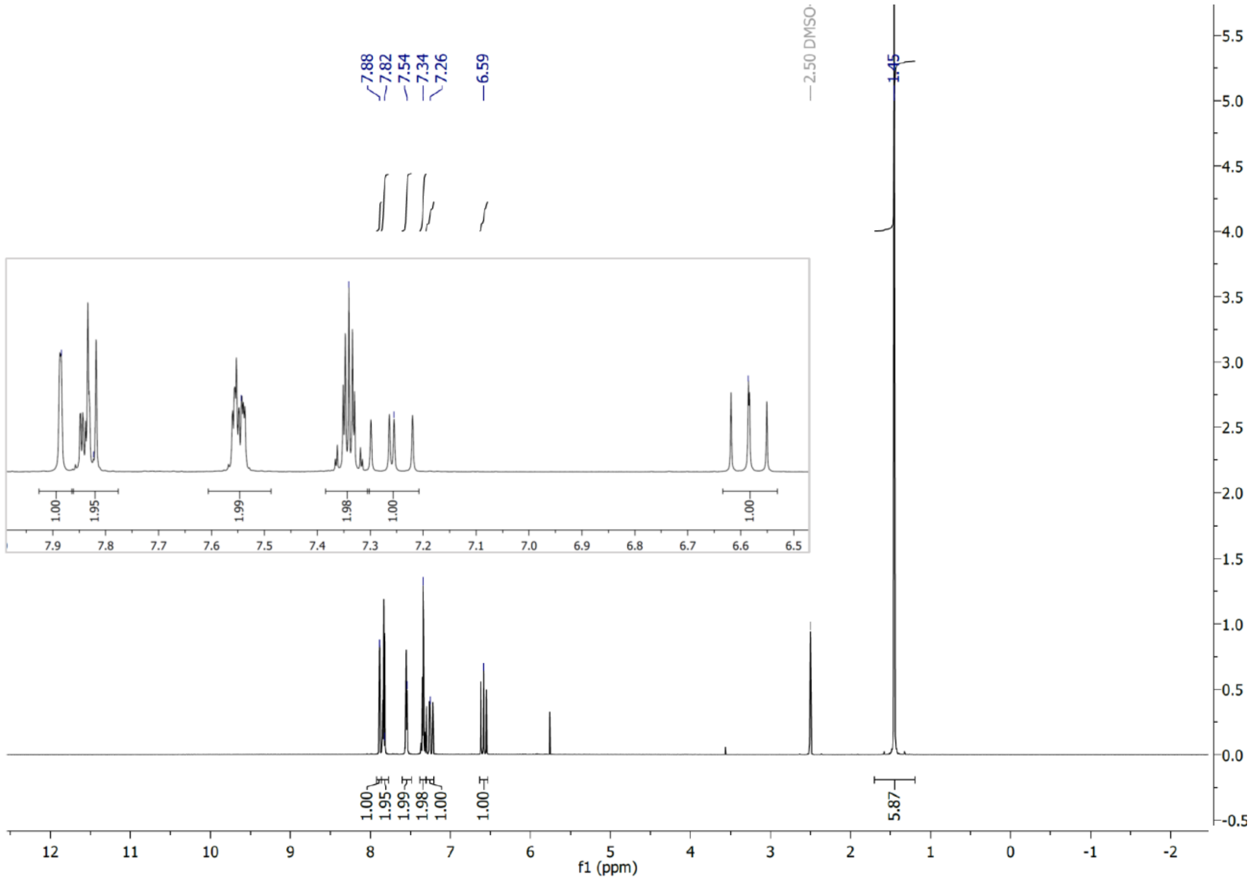

Figure S25. ^{1}H NMR spectrum of (E)-(2-(9,9-dimethyl-9H-fluoren-2-yl)vinyl)phosphonic acid (DMSO- d_6 δ 2.50, 500 MHz, 298 K)

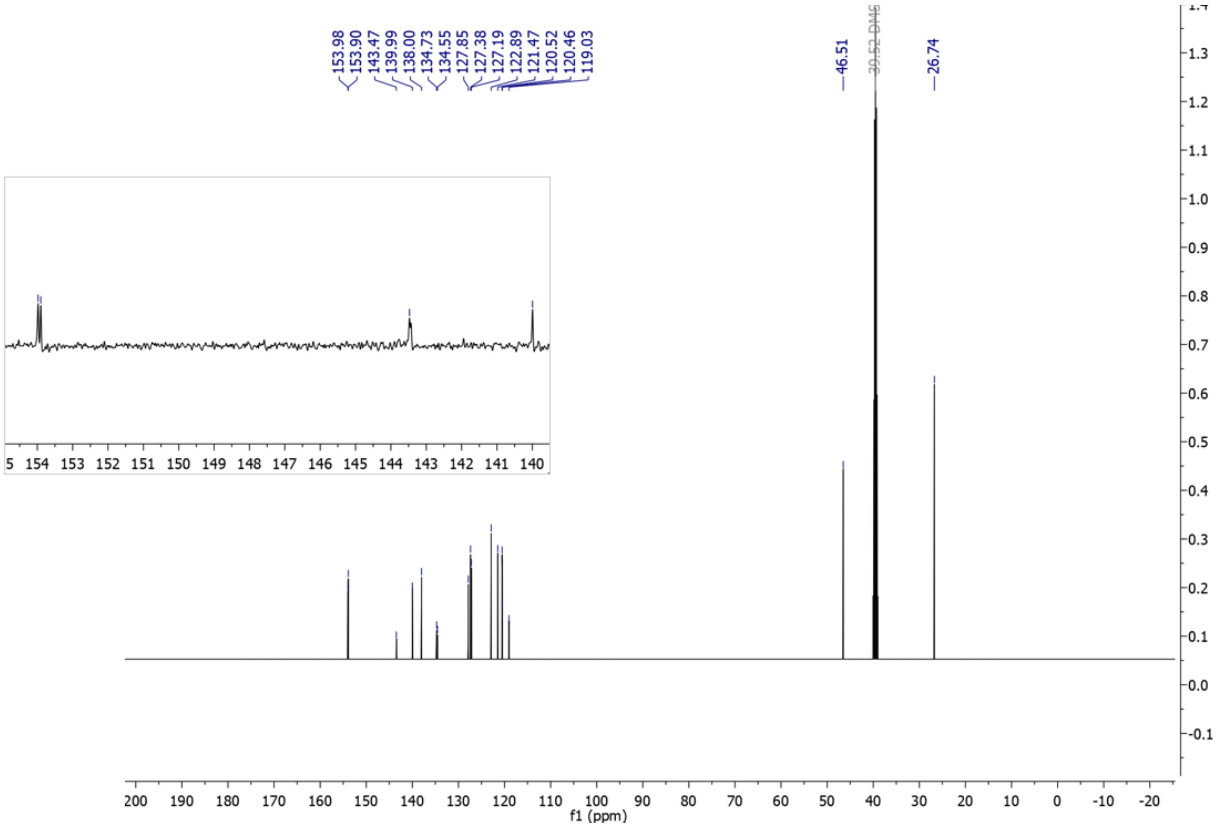

Figure S26. ^{13}C NMR spectrum of (E)-(2-(9,9-dimethyl-9H-fluoren-2-yl)vinyl)phosphonic acid (DMSO- d_6, 126 MHz, 298 K)

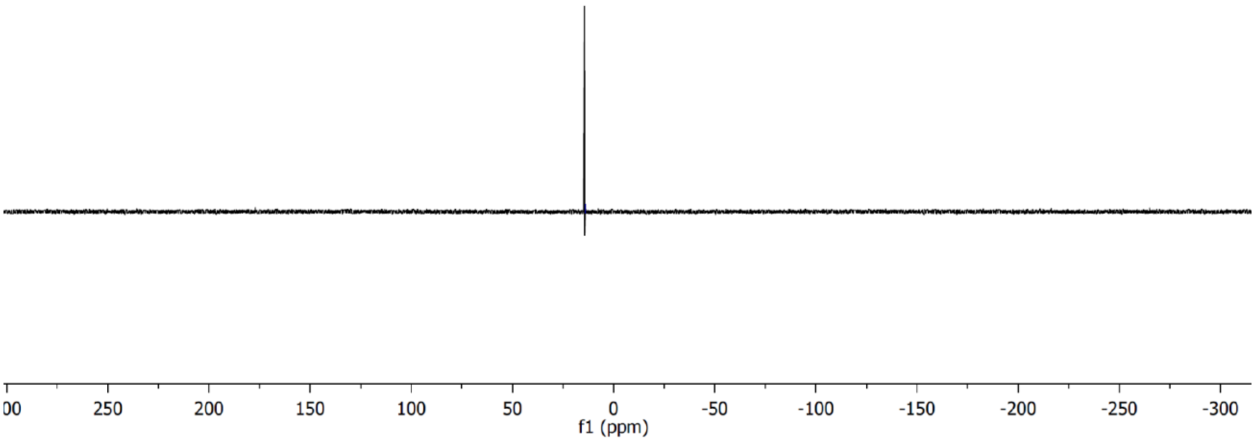

Figure S27. ^{31}P NMR spectrum of (E)-(2-(9,9-dimethyl-9H-fluoren-2-yl)vinyl)phosphonic acid (DMSO-d_6, 202 MHz, 298 K)

((1E,1'E)-(9,9-dihexyl-9H-fluorene-2,7-diyl)bis(ethene-2,1-diyl))bis(phosphonic acid) (Dihexyl fluorene bis(vinylphosphonic acid) (10))

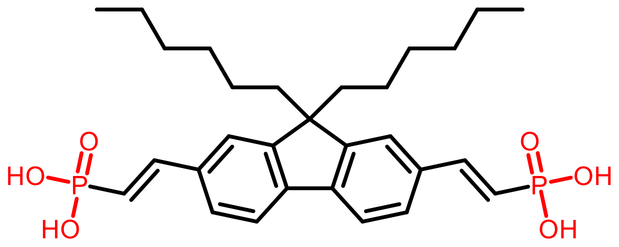

A 100 mL Schlenk flask with stir bar was oven dried. The Schlenk flask was charged with 2,7-dibromo-9,9-dihexyl-9H-fluorene (2.80 g, 5.68 mmol) and bis(tri-tert-butyl phosphine)palladium(0) (0.116 g, 0.23 mmol), followed by vacuum and N_2 refill cycles (3×). Vinyl phosphonic acid (VPA) (1.35 g, 12.5 mmol) was dissolved in 5 mL of anhydrous dioxane and gently bubbled with N_2 for 20 min. Anhydrous dioxane (55 mL) and the solution of VPA was added to the Schenk flask *via* syringe with stirring. N,N-Dicyclohexylmethylamine (3.62 mL, 17.04 mmol) was added to the reaction mixture dropwise *via* syringe. The reaction was stirred at 80°C for 20 h. The progress was monitored with thin layer chromatography (EtOAc/Hexanes 1:4). Upon reaction completion, mixture was cooled to room temperature. The product was extracted with EtOAc and washed with 5% HCl (3×). Organic phase was dried over $MgSO_4$ and filtered. Obtained organic phase was condensed to a few mL by rotary evaporator. The product was precipitated into DCM and filtered product (light grey solid) was dried overnight at 45°C under vacuum. Yield of 1.74 g (53%). ^{1}H NMR (500 MHz, DMSO-d_6) δ 7.80 (s, 2H), 7.74 (s, 2H), 7.55 (s, 2H), 7.21 (s, 2H), 6.58 (s, 2H), 2.04 (s, 4H), 0.97 (s, 12H), 0.70 (s, 6H), 0.45 (s, 4H). ^{31}P NMR (202 MHz, DMSO-d_6) δ 14.19. ^{13}C NMR (126 MHz, DMSO-d_6) δ 151.43, 143.49, 141.44, 134.91, 134.73, 127.14, 121.60, 120.58 (d, J = 16.2 Hz), 119.19, 54.81, 39.24, 30.93, 28.98, 23.45, 21.99, 13.83.

The purity of the compound was determined from ^{1}H, ^{13}C, and ^{31}P NMR. Integration of the ^{1}H NMR in addition to the single peak in the ^{31}P NMR and the absence of the signal attributed to the starting VPA indicated high compound purity (>98%). The doublet at 120.58 ppm in ^{13}C NMR is assigned to the β-carbon, the splitting is attributed to the ^{31}P-C coupling.

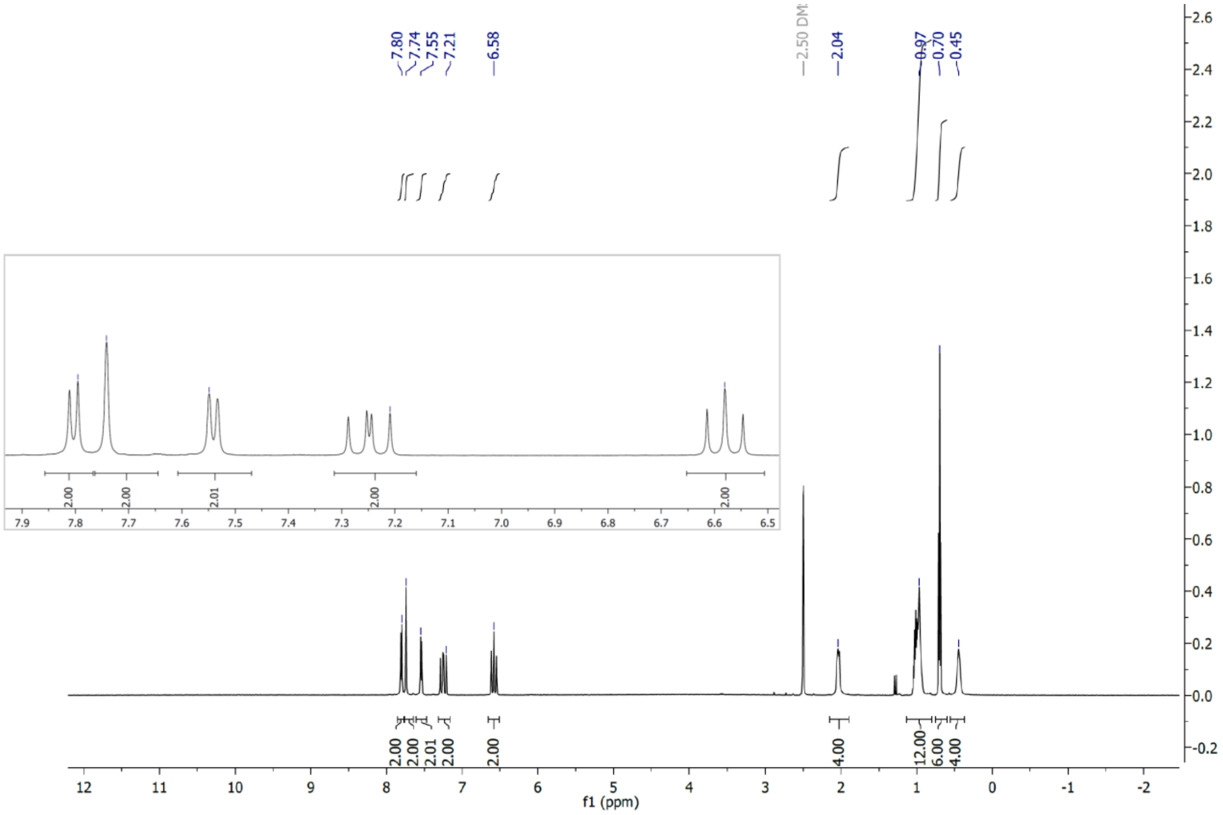

Figure S28. ^{1}H NMR spectrum of ((1E,1'E)-(9,9-dihexyl-9H-fluorene-2,7-diyl)bis(ethene-2,1-diyl))bis(phosphonic acid) (DMSO- d_6 δ 2.50, 500 MHz, 298 K)

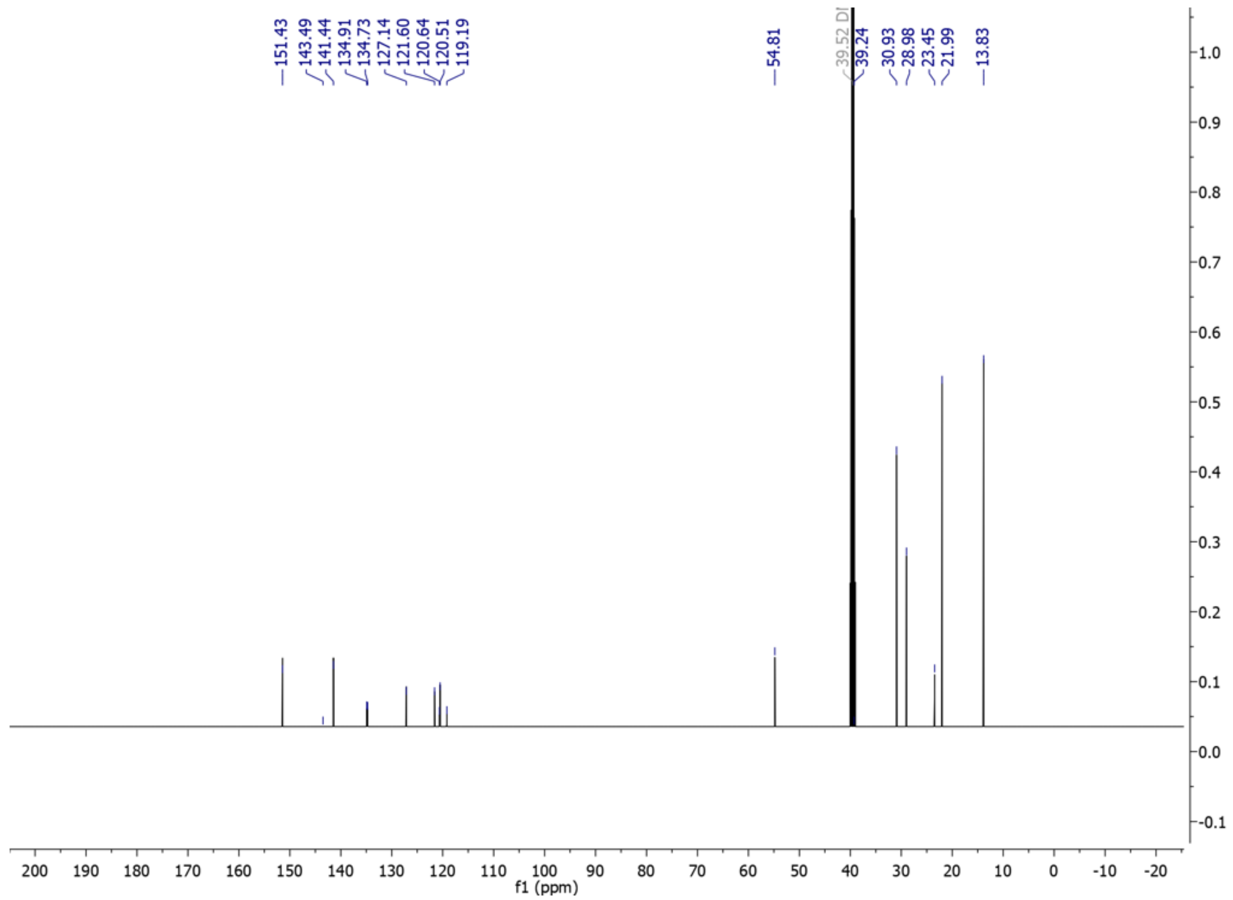

Figure S29. ^{13}C NMR spectrum of ((1E,1'E)-(9,9-dihexyl-9H-fluorene-2,7-diyl)bis(ethene-2,1-diyl))bis(phosphonic acid) (DMSO- d_6, 126 MHz, 298 K)

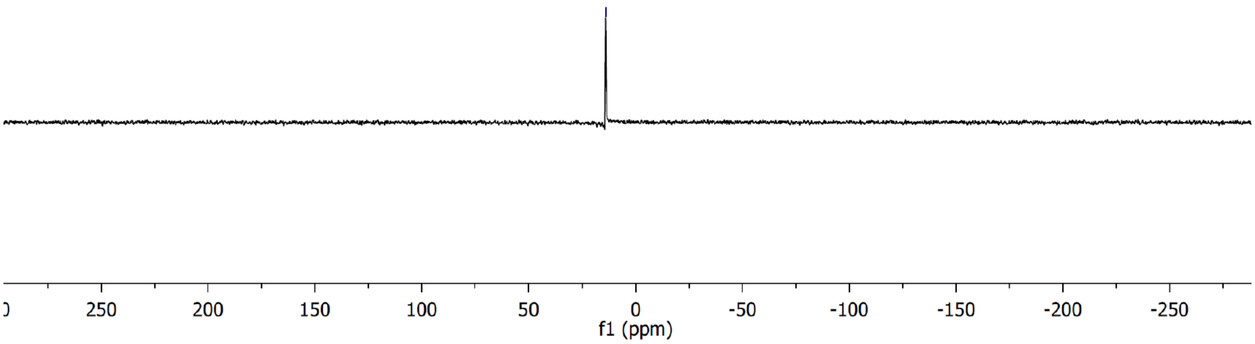

Figure S30. ^{31}P NMR spectrum of ((1E,1'E)-(9,9-dihexyl-9H-fluorene-2,7-diyl)bis(ethene-2,1-diyl))bis(phosphonic acid) (DMSO-d_6, 202 MHz, 298 K)

((1E,1'E)-(9,9-dimethyl-9H-fluorene-2,7-diyl)bis(ethene-2,1-diyl))bis(phosphonic acid) (Dimethyl fluorene bis(vinylphosphonic acid) (11))

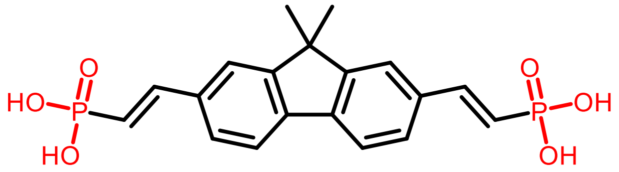

A 100 mL Schlenk flask with stir bar was oven dried. The Schlenk flask was charged with 2,7-dibromo-9,9-dimethyl-9H-fluorene (2.00 g, 5.68 mmol) and bis(tri-tert-butyl phosphine)palladium(0) (0.116 g, 0.23 mmol), followed by vacuum and N_2 refill cycles (3×). Vinyl phosphonic acid (VPA) (1.35 g, 12.5 mmol) was dissolved in 5 mL of anhydrous dioxane and gently bubbled with N_2 for 20 min. Anhydrous dioxane (55 mL) and the solution of VPA was added to the Schenk flask via syringe with stirring. N,N-Dicyclohexylmethylamine (3.62 mL, 17.04 mmol) was added to the reaction mixture dropwise via syringe. The reaction was stirred at 80°C for 20 h. The progress was monitored with thin layer chromatography (EtOAc/Hexanes 1:4). Upon reaction completion, mixture was cooled to room temperature. The product was extracted with EtOAc and washed with 5% HCl (3×). Organic phase was dried over $MgSO_4$ and filtered. Obtained organic phase was condensed to a few mL by rotary evaporator. The product was precipitated into DCM and filtered product (light grey solid) was dried overnight at 45°C under vacuum. Yield of 1.43 g (62%). ^{1}H NMR (500 MHz, DMSO-d_6) δ 7.89 (d, 1H), 7.75 (s, 2H), 7.61 (d, 1H), 7.26 (dd, 1H), 6.53 (t, 1H). ^{31}P NMR (202 MHz, DMSO-d_6) δ 14.33. ^{13}C NMR (126 MHz, DMSO-d_6) δ 154.57, 143.25 (d, J = 6.0 Hz), 139.33, 135.10, 134.93, 127.46, 121.48, 120.84 (d, J = 6.1 Hz), 119.41, 46.55, 26.60.

The purity of the compound was determined from ^{1}H, ^{13}C, and ^{31}P NMR. Integration of the ^{1}H NMR in addition to the single peak in the ^{31}P NMR and the absence of the signal attributed to the starting VPA indicated high compound purity (>97%). The doublets at 143.25 and 120.84 ppm in ^{13}C NMR are assigned to the vinyl linkage carbons adjacent to phosphorus, the splitting is attributed to the ^{31}P-C coupling.

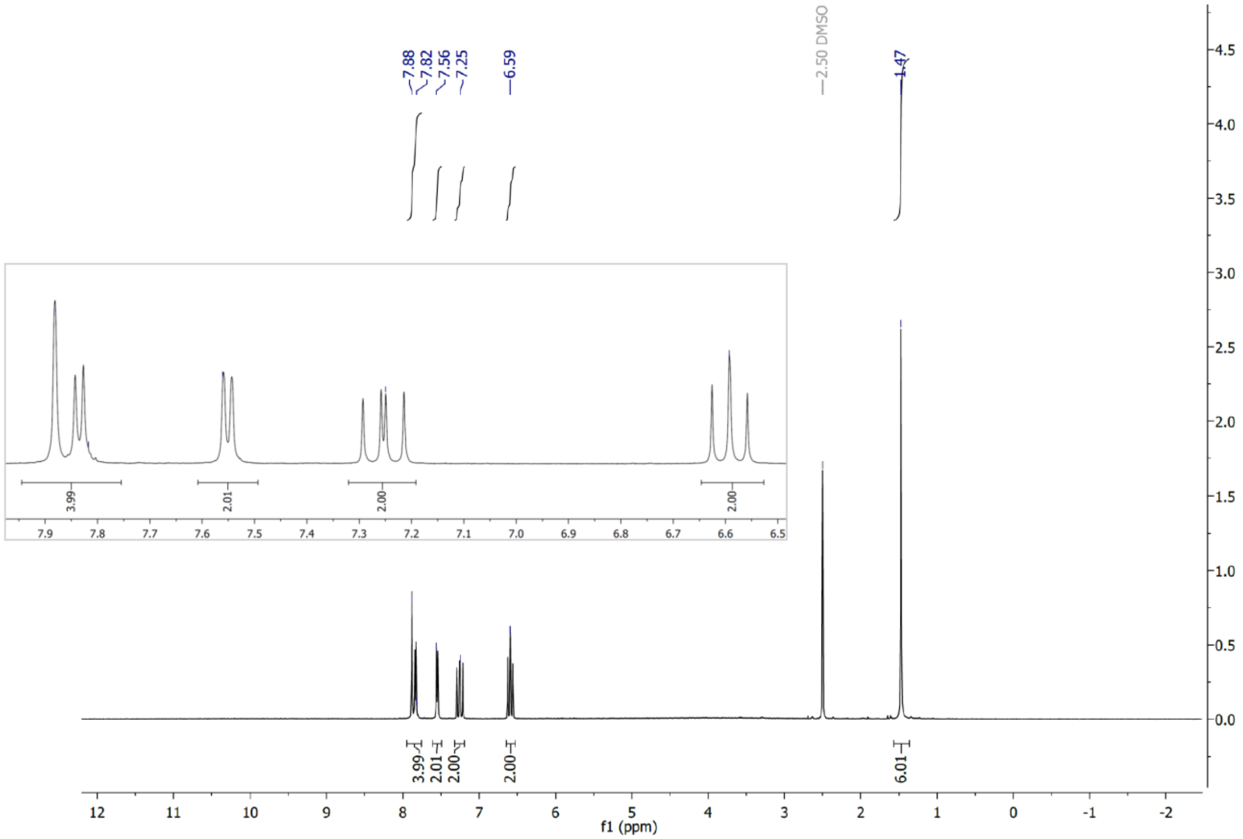

Figure S31. ^{1}H NMR spectrum of ((1E,1'E)-(9,9-dimethyl-9H-fluorene-2,7-diyl)bis(ethene-2,1-diyl))bis(phosphonic acid) (DMSO- *d_6* δ 2.50, 500 MHz, 298 K)

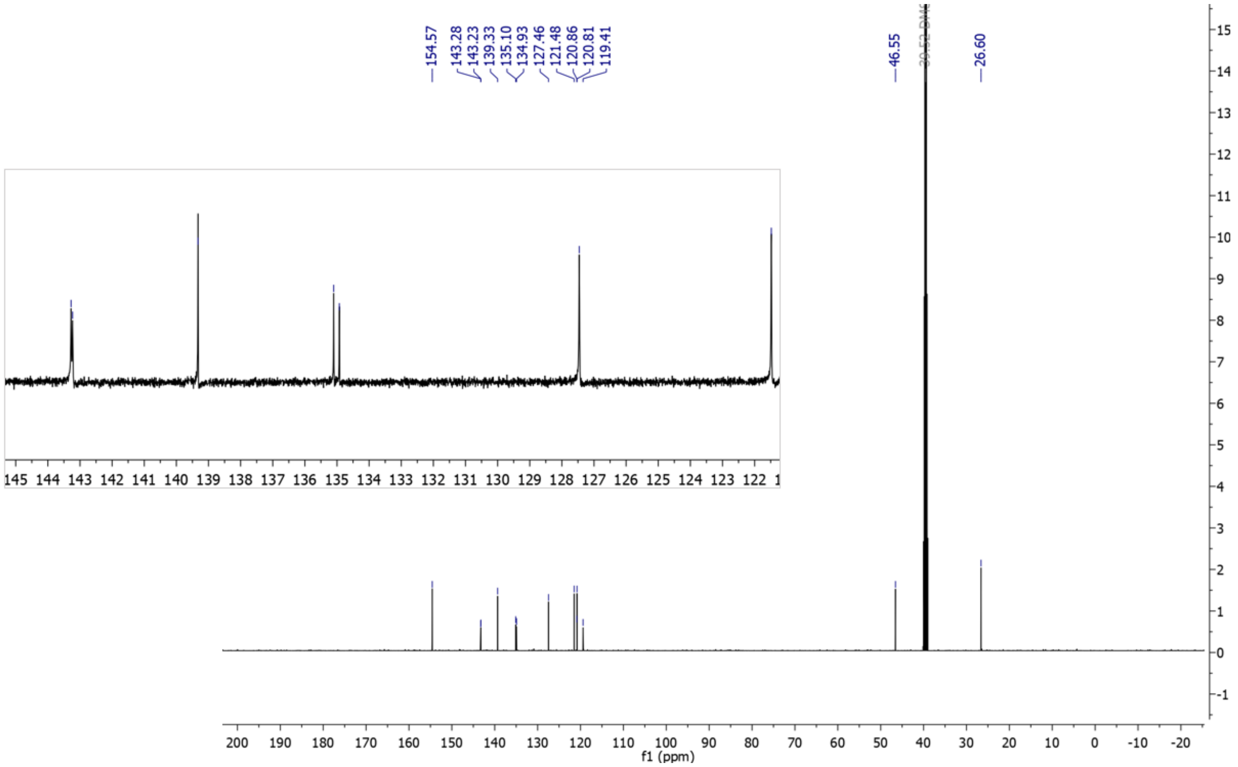

Figure S32. ^{13}C NMR spectrum of ((1E,1'E)-(9,9-dimethyl-9H-fluorene-2,7-diyl)bis(ethene-2,1-diyl))bis(phosphonic acid) (DMSO- *d*$_6$, 126 MHz, 298 K)

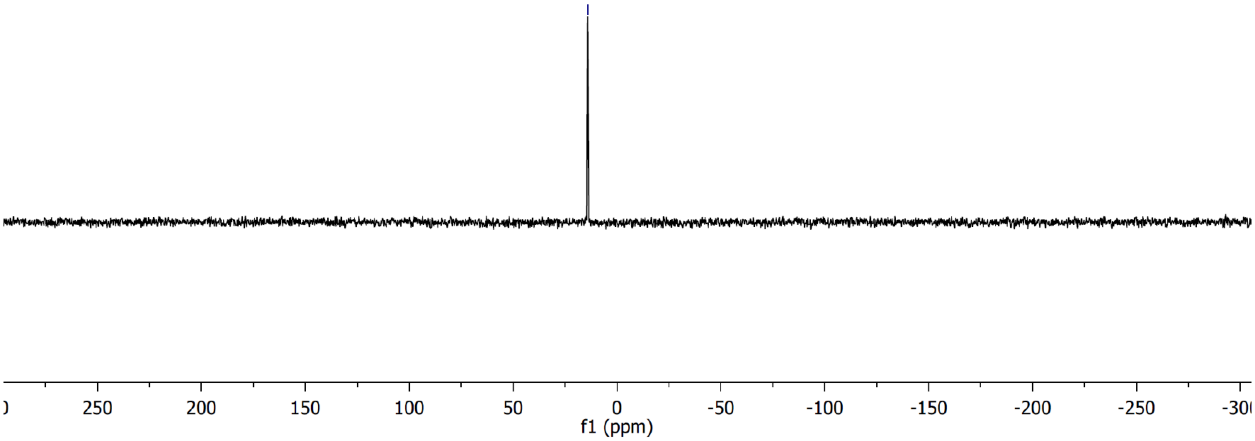

Figure S33. ^{31}P NMR spectrum of ((1E,1'E)-(9,9-dimethyl-9H-fluorene-2,7-diyl)bis(ethene-2,1-diyl))bis(phosphonic acid) (DMSO-*d*$_6$, 202 MHz, 298 K)

((1E,1'E)-(9,9-bis(2-ethylhexyl)-9H-fluorene-2,7-diyl)bis(ethene-2,1-diyl))bis(phosphonic acid) (Di(2-ethylhexyl) fluorene bis(vinylphosphonic acid) (12))

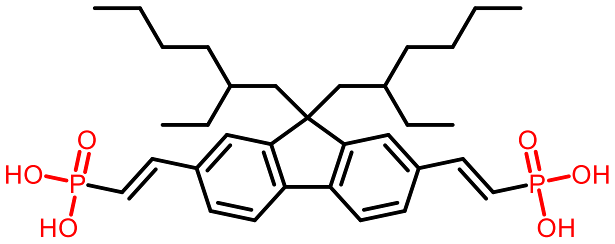

A 100 mL Schlenk flask with stir bar was oven dried. The Schlenk flask was charged with 2,7-dibromo-9,9-bis(2-ethylhexyl)-9H-fluorene (1.00 g, 1.89 mmol) and bis(tri-tert-butyl phosphine)palladium(0) (0.024 g, 0.047 mmol), followed by vacuum and N_2 refill cycles (3×). Vinyl phosphonic acid (VPA) (0.51 g, 4.73 mmol) was dissolved in 5 mL of anhydrous dioxane and gently bubbled with N_2 for 20 min. Anhydrous dioxane (55 mL) and the solution of VPA was added to the Schenk flask via syringe with stirring. N,N-Dicyclohexylmethylamine (2.01 mL, 9.45 mmol) was added to the reaction mixture dropwise via syringe. The reaction was stirred at 80°C for 20 h. The progress was monitored with thin layer chromatography (EtOAc/Hexanes 1:4). Upon reaction completion, mixture was cooled to room temperature. The product was extracted with EtOAc and washed with 5% HCl (3x). Organic phase was dried over $MgSO_4$ and filtered. Obtained organic phase was condensed to a few mL by rotary evaporator. The product was precipitated into DCM and filtered product (light grey solid) was dried overnight at 45°C under vacuum. Yield of 0.950 g (66%). ^{1}H NMR (500 MHz, DMSO-d_6) δ 7.82 (s, 3H), 7.57 (dd, 2H), 7.26 (dd, 1H), 6.54 (t, 10H), 2.07 (s, 8H), 0.89 – 0.57 (m, 27H), 0.48 – 0.42 (m, 7H), 0.36 (hept, 2H). ^{31}P NMR (202 MHz, DMSO-d_6) δ 14.48. ^{13}C NMR (126 MHz, DMSO-d_6) δ 151.24, 143.52 (d, J = 7.4 Hz), 141.60, 134.26 (dt, J = 23.6, 12.0 Hz), 126.84 (t, J = 10.4 Hz), 123.02 (t, J = 17.0 Hz), 120.38 (d, J = 13.4 Hz), 118.97, 54.55, 43.24, 34.12, 33.09, 33.04, 27.44, 26.56, 22.09, 13.86, 10.25, 10.22.

The purity of the compound was determined from ^{1}H, ^{13}C, and ^{31}P NMR. Integration of the ^{1}H NMR in addition to the single peak in the ^{31}P NMR and the absence of the signal attributed to the starting VPA indicated high compound purity (>98%). The doublet at 143.52 ppm in ^{13}C NMR is assigned to the carbon adjacent to phosphorus, the splitting is attributed to the ^{31}P-C coupling. The presence of two 2-ethylhexyl chains increased Ar-VPA solubility and resulted in a saturated NMR sample, thus, the splitting of aromatic carbons is attributed to the formation of polymeric species in the solution.

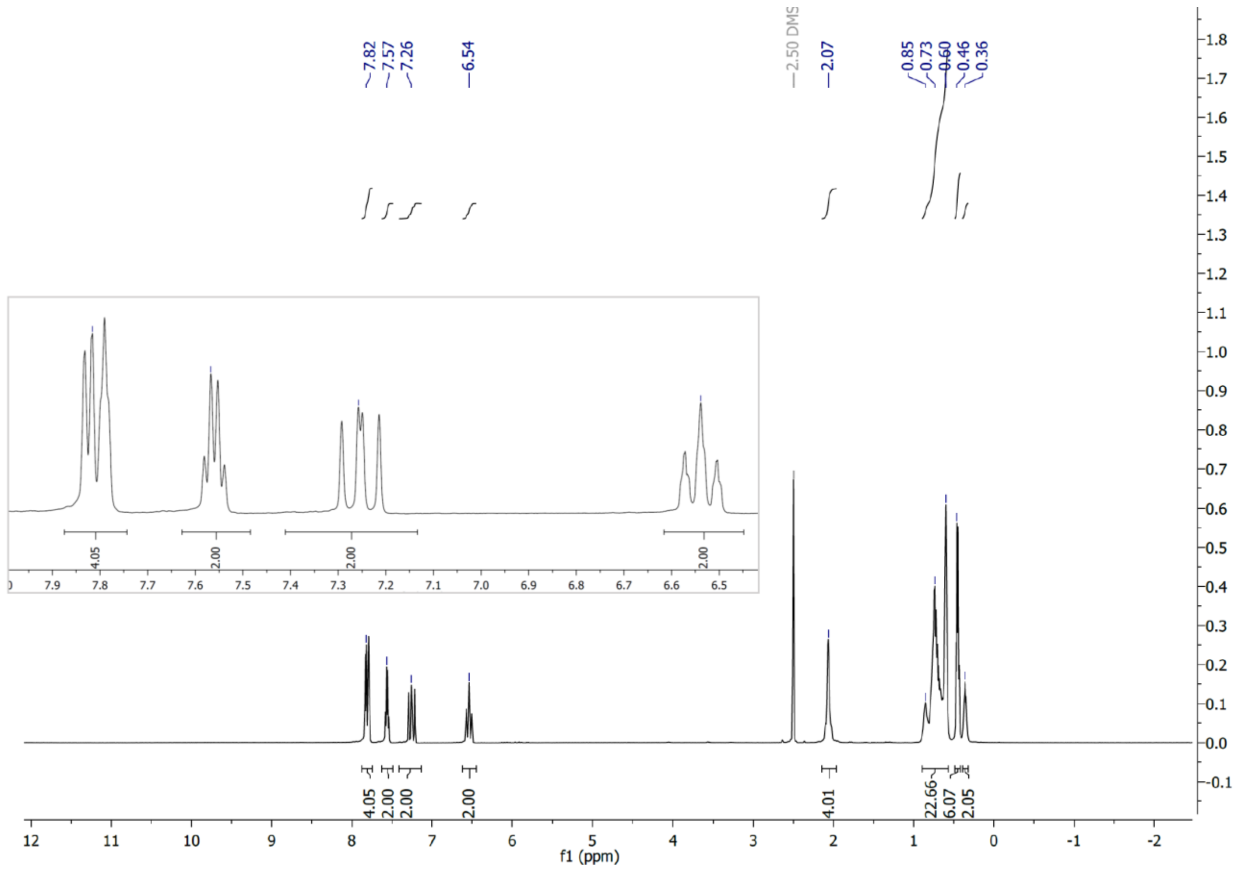

Figure S34. ^{1}H NMR spectrum of ((1E,1'E)-(9,9-bis(2-ethylhexyl)-9H-fluorene-2,7-diyl)bis(ethene-2,1-diyl))bis(phosphonic acid) (DMSO-d_6 δ 2.50, 500 MHz, 298 K)

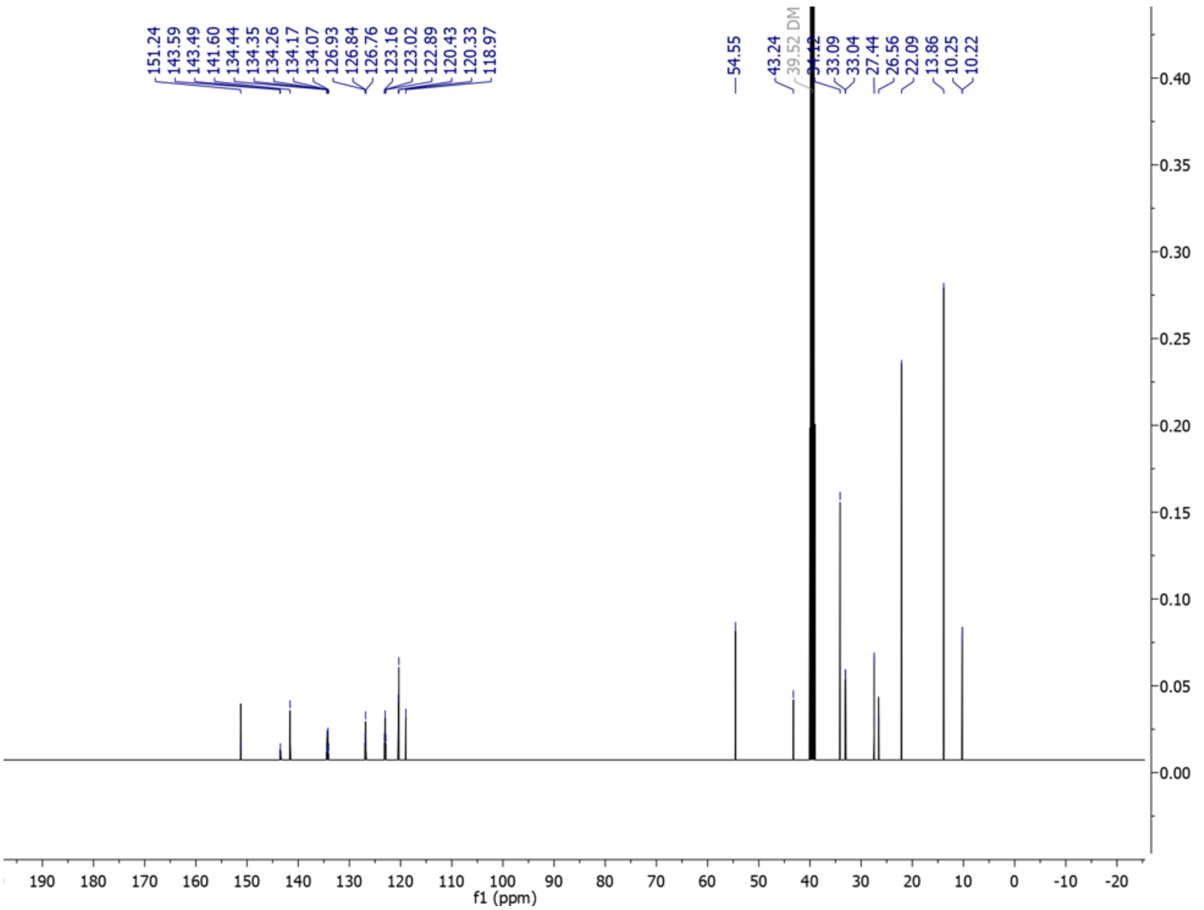

Figure S35. ^{13}C NMR spectrum of ((1E,1'E)-(9,9-bis(2-ethylhexyl)-9H-fluorene-2,7-diyl)bis(ethene-2,1-diyl))bis(phosphonic acid) (DMSO- d_6, 126 MHz, 298 K)

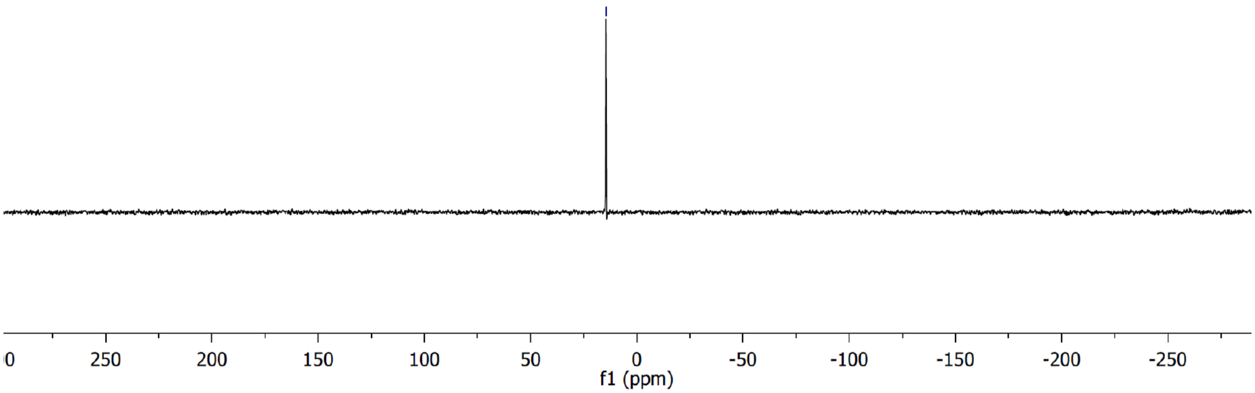

Figure S36. ^{31}P NMR spectrum of ((1E,1'E)-(9,9-bis(2-ethylhexyl)-9H-fluorene-2,7-diyl)bis(ethene-2,1-diyl))bis(phosphonic acid) (DMSO-d_6, 202 MHz, 298 K)

((1E,1'E)-(9-(2-ethylhexyl)-9H-carbazole-2,7-diyl)bis(ethene-2,1-diyl))bis(phosphonic acid) (2-ethylhexyl carbazole bis(vinylphosphonic acid) (13))

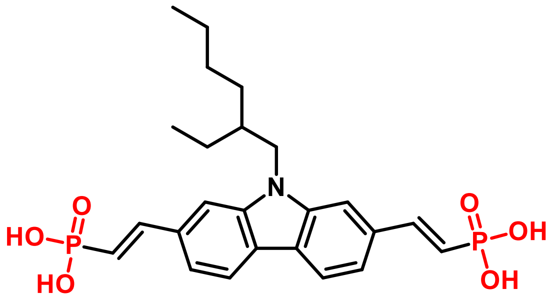

A 100 mL Schlenk flask with stir bar was oven dried. The Schlenk flask was charged with 2,7-dibromo-9-(2-ethylhexyl)-9H-carbazole (1.0 g, 2.4 mmol) and bis(tri-tert-butyl phosphine)palladium(0) (0.049 g, 0.096 mmol), followed by vacuum and N_2 refill cycles (3×). Vinyl phosphonic acid (VPA) (0.57 g, 5.3 mmol) was dissolved in 15 mL of anhydrous dioxane and gently bubbled with N_2 for 20 min. Anhydrous dioxane (35 mL) and the solution of VPA was added to the Schenk flask via syringe with stirring. N,N-Dicyclohexylmethylamine (2.0 mL, 9.5 mmol) was added to the reaction mixture dropwise via syringe. The reaction was stirred at 70°C for 20 h. The progress was monitored with thin layer chromatography (EtOAc/Hexanes 1:4). Upon reaction completion, mixture was cooled to room temperature. Product was extracted with EtOAc and washed with 5% HCl (3x). Organic phase was dried over $MgSO_4$ and filtered. Obtained organic phase was condensed to a few mL by rotary evaporator. The product was precipitated into DCM and filtered product (bright yellow crystals) was dried overnight at 45°C under vacuum. Yield of 0.714 g (61%) ^{1}H NMR (500 MHz, DMSO-d_6) δ 8.08 (d, 2H), 7.69 (s, 2H), 7.45 (s, 2H), 7.36 (s, 2H), 6.62 (s, 2H), 4.18 (s, 2H), 1.96 (s, 1H), 1.20 (d, J = 74.6 Hz, 8H), 0.78 (d, J = 24.3 Hz, 6H). ^{31}P NMR (202 MHz, DMSO-d_6) δ 14.62. ^{13}C NMR (126 MHz, DMSO-d_6) δ 144.04 (d, J = 8.6 Hz), 141.37, 133.51, 133.33, 122.69, 120.73, 118.27 (d, J = 18.6 Hz), 109.21, 46.26, 38.44, 29.96, 27.84, 23.55, 22.58, 13.92, 10.81.

The purity of the compound was determined from ^{1}H, ^{13}C, and ^{31}P NMR. Integration of the ^{1}H NMR in addition to the single peak in the ^{31}P NMR and the absence of the signal attributed to the starting VPA indicated high compound purity (>96%). The doublets at 144.04 and 118.27 ppm in ^{13}C NMR are assigned to the vinyl linkage carbons adjacent to phosphorus, the splitting is attributed to the ^{31}P-C coupling.

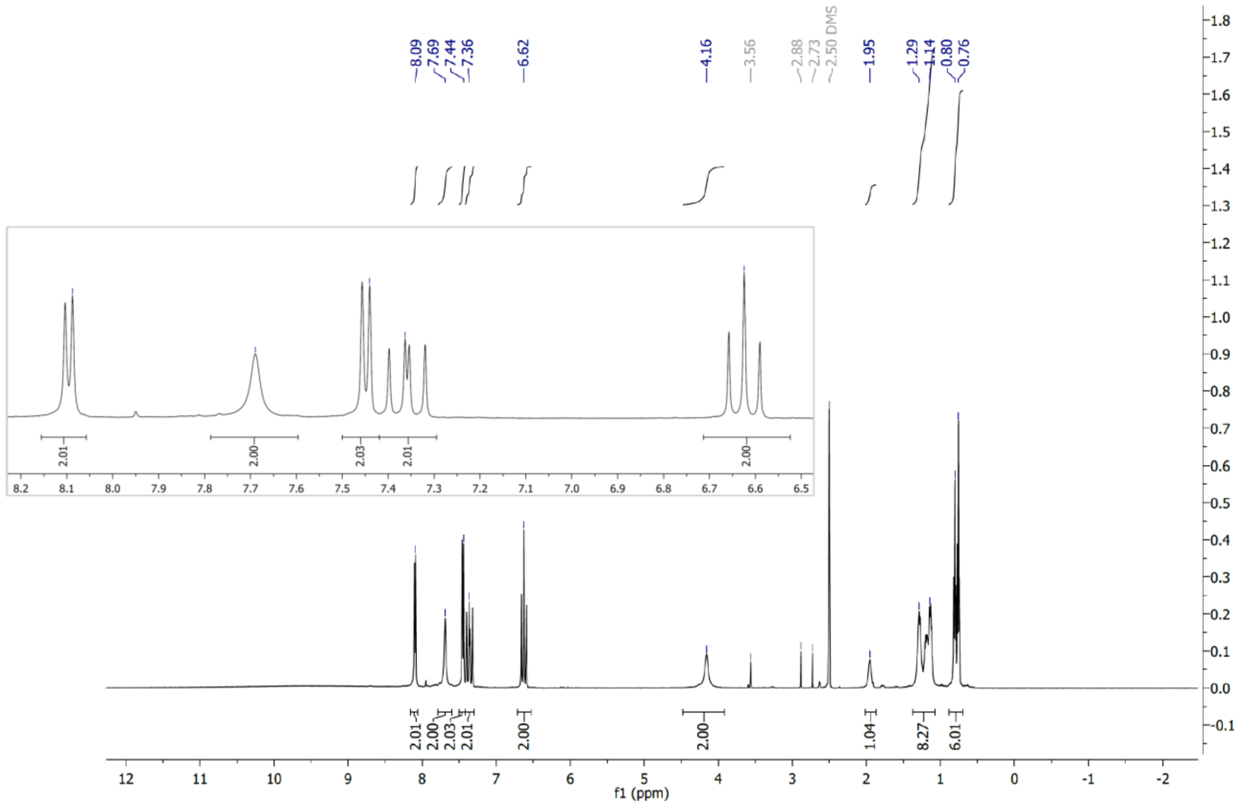

Figure S37. ^1H NMR spectrum of ((1E,1'E)-(9-(2-ethylhexyl)-9H-carbazole-2,7-diyl)bis(ethene-2,1-diyl))bis(phosphonic acid) (DMSO-d_6 δ 2.50, 500 MHz, 298 K)

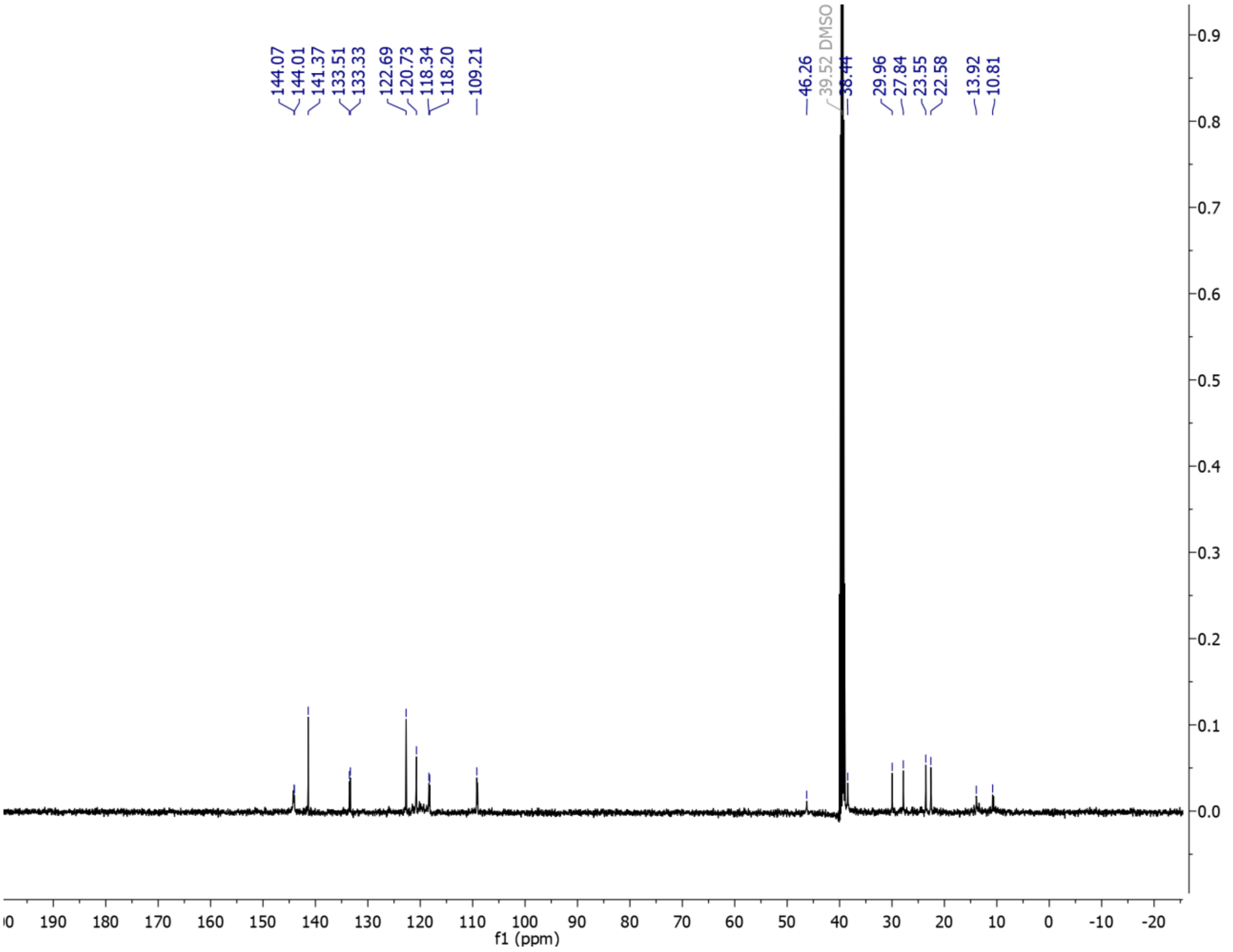

Figure S38. ^{13}C NMR spectrum of ((1E,1'E)-(9-(2-ethylhexyl)-9H-carbazole-2,7-diyl)bis(ethene-2,1-diyl))bis(phosphonic acid) (DMSO- d_6, 126 MHz, 298 K)

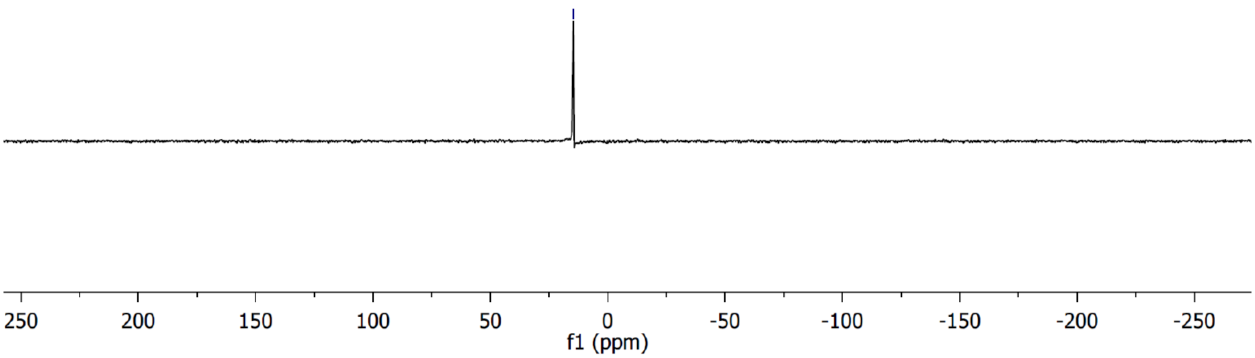

Figure S39. ^{31}P NMR spectrum of ((1E,1'E)-(9-(2-ethylhexyl)-9H-carbazole-2,7-diyl)bis(ethene-2,1-diyl))bis(phosphonic acid) (DMSO-d_6, 202 MHz, 298 K)

S3. Standard Procedure of Converting Phosphonic Acid to Tetrabutylammonium Phosphonate

Small scale tetrabutylammonium phosphonate preparation: (< 10 mg phosphonic acid)[1] The phosphonate tetrabutylammonium salt was formed by titrating the corresponding acid with aliquots of tetrabutylammonium hydroxide acetonitrile solution (tetrabutylammonium hydroxide 30-hydrate dissolved in deuterium acetonitrile) until deprotonation was complete as verified by ^{1}H NMR spectroscopy based on the integration of α-proton of tetrabutylammonium and one proton of vinyl group from phosphonic acid. Then the solvent was evaporated, and the resulting salt was dried under vacuum for at least two days at room temperature before using for complexation.

Large scale tetrabutylammonium phosphonate preparation: (> 100 mg tetrabutylammonium hydroxide 30-hydrate)[2, 3] The phosphonic acid was weighted out and dissolved in around 5 mL MeOH. The amount of tetrabutylammonium hydroxide 30-hydrate needed to deprotonate one proton of the acid was weighted out accurately and dissolved in 2 mL MeOH. Two solutions were mixed and stirred for 15 min. Then the solvent was evaporated and the resulting salt was dried under vacuum for at least two days at room temperature before using for complexation.

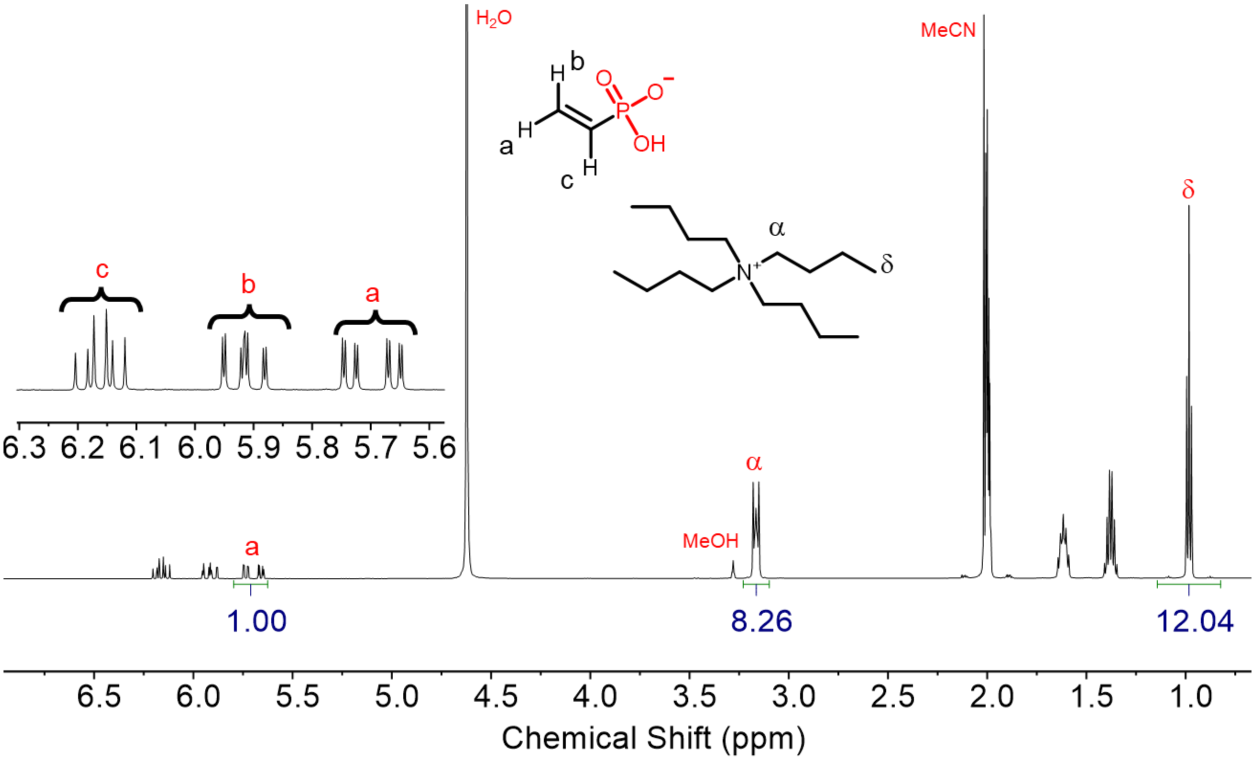

Figure S40. ^{1}H NMR spectrum of tetrabutylammonium salt of vinylphosphonate. (CD_3OD-CD_3CN, 600 MHz, 298 K)

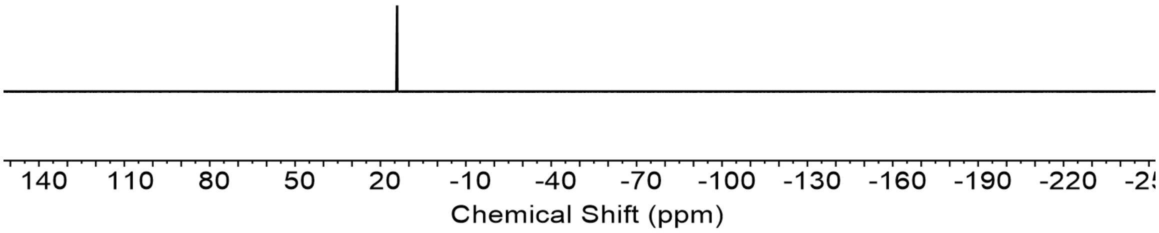

Figure S41. ^{31}P NMR spectrum of tetrabutylammonium salt of vinylphosphonate, δ=14.15 ppm. (CD_3OD-CD_3CN, 202 MHz, 298 K)

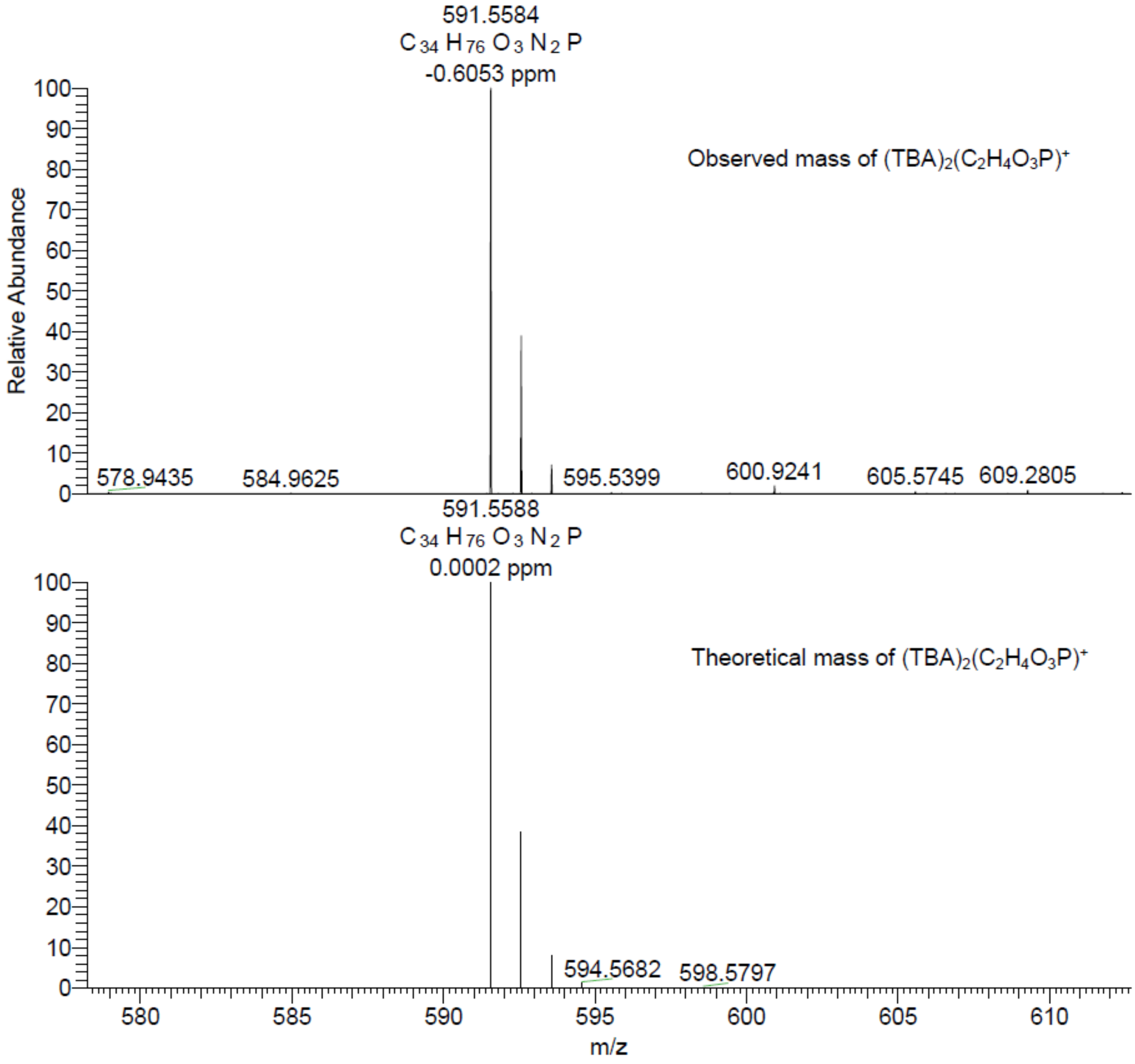

Figure S42. HRMS spectrum of tetrabutylammonium salt of vinylphosphonate.

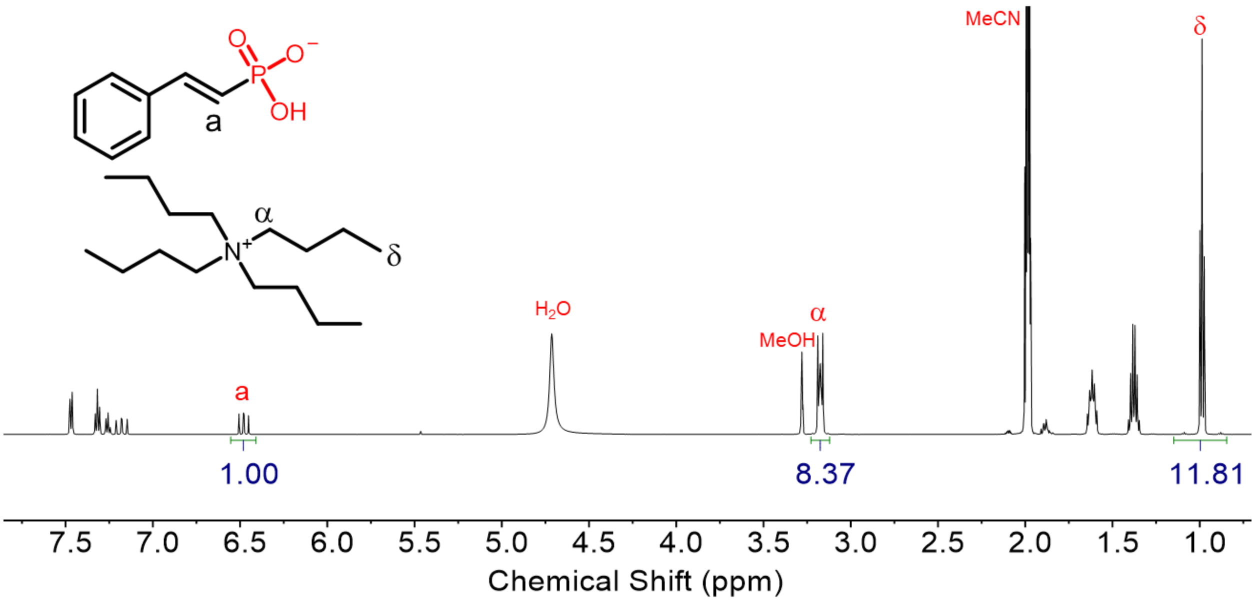

Figure S43. ^{1}H NMR spectrum of tetrabutylammonium salt of phenyl vinylphosphonate. (CD_3OD-CD_3CN, 600 MHz, 298 K)

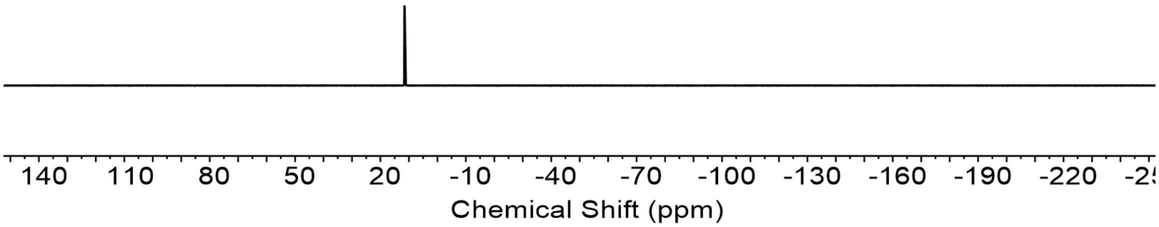

Figure S44. ^{31}P NMR spectrum of tetrabutylammonium salt of phenyl vinylphosphonate, δ=11.55 ppm. (CD_3OD-CD_3CN, 202 MHz, 298 K)

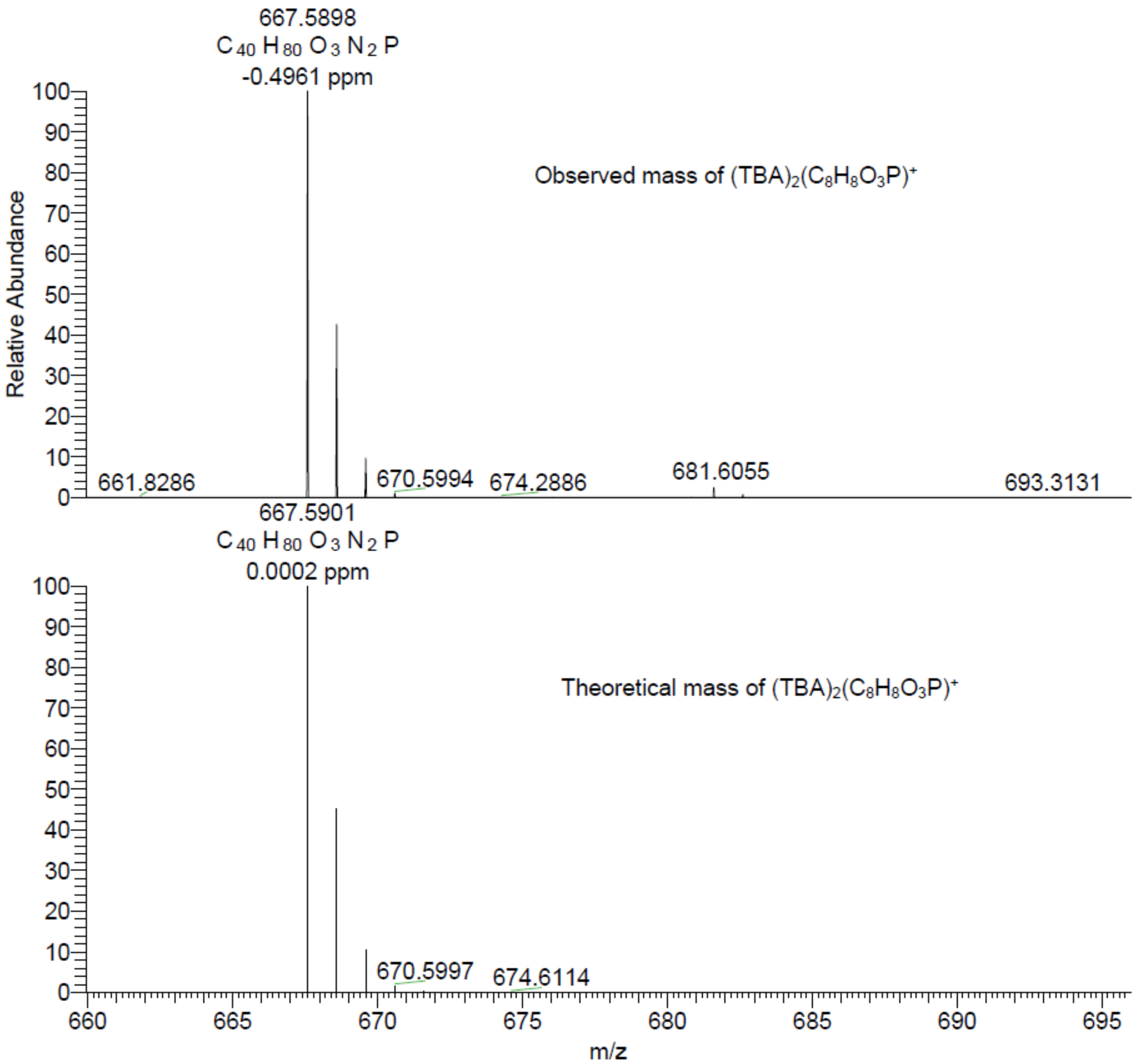

Figure S45. HRMS spectrum of tetrabutylammonium salt of phenyl vinylphosphonate.

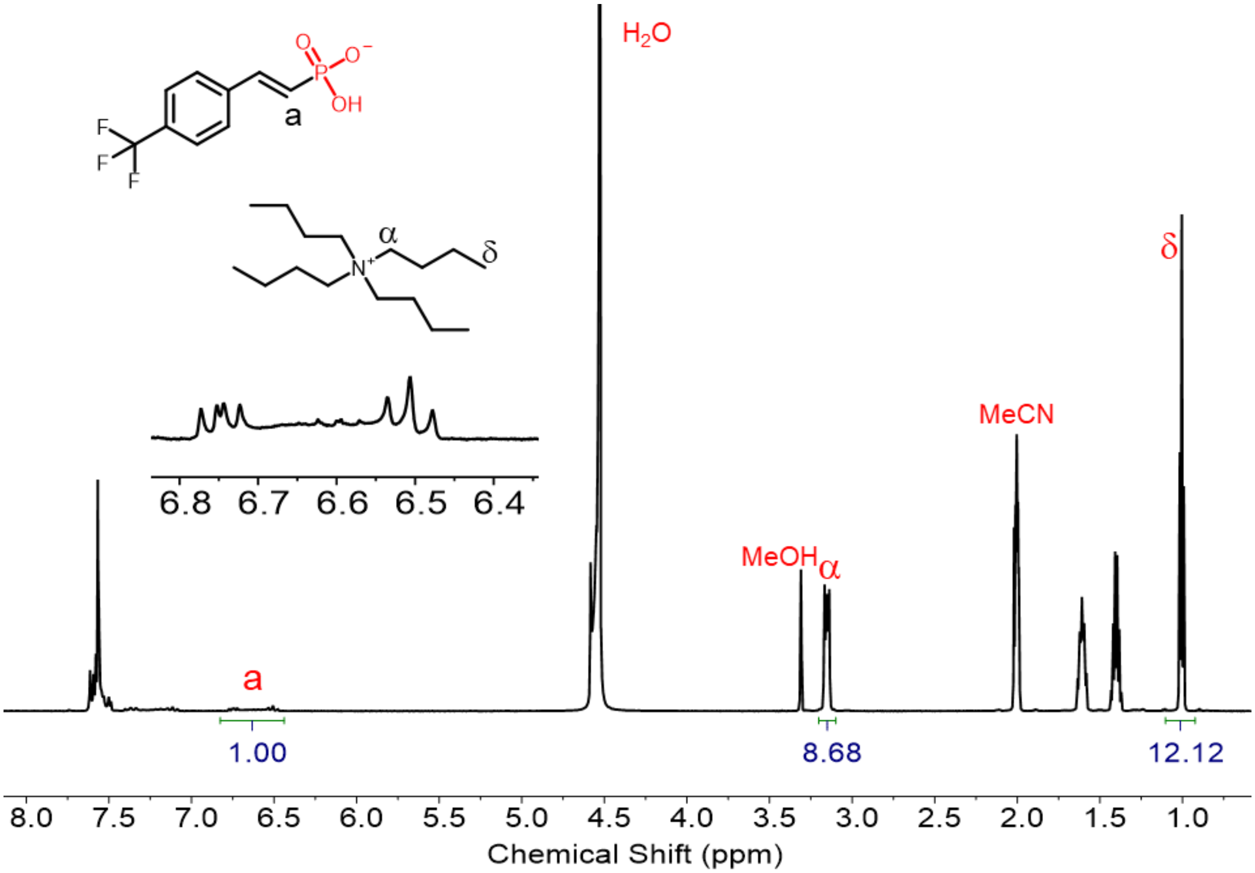

Figure S46. ^{1}H NMR spectrum of tetrabutylammonium salt of 4-trifluoromethylbenzyl vinylphosphonate. (CD_3OD-CD_3CN, 600 MHz, 298 K)

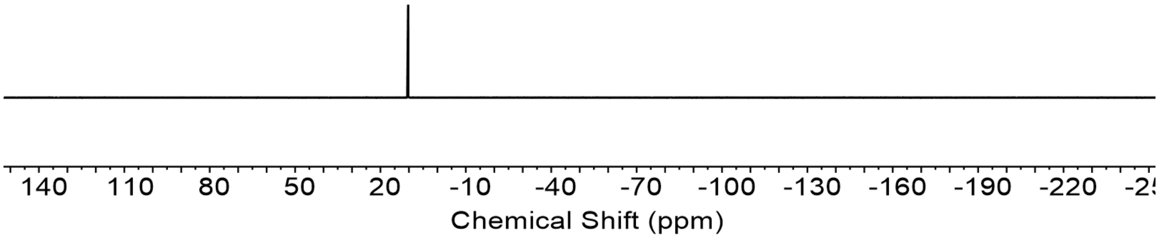

Figure S47. ^{31}P NMR spectrum of tetrabutylammonium salt of 4-trifluoromethylbenzyl vinylphosphonate, δ=10.28 ppm. (CD_3OD-CD_3CN, 202 MHz, 298 K)

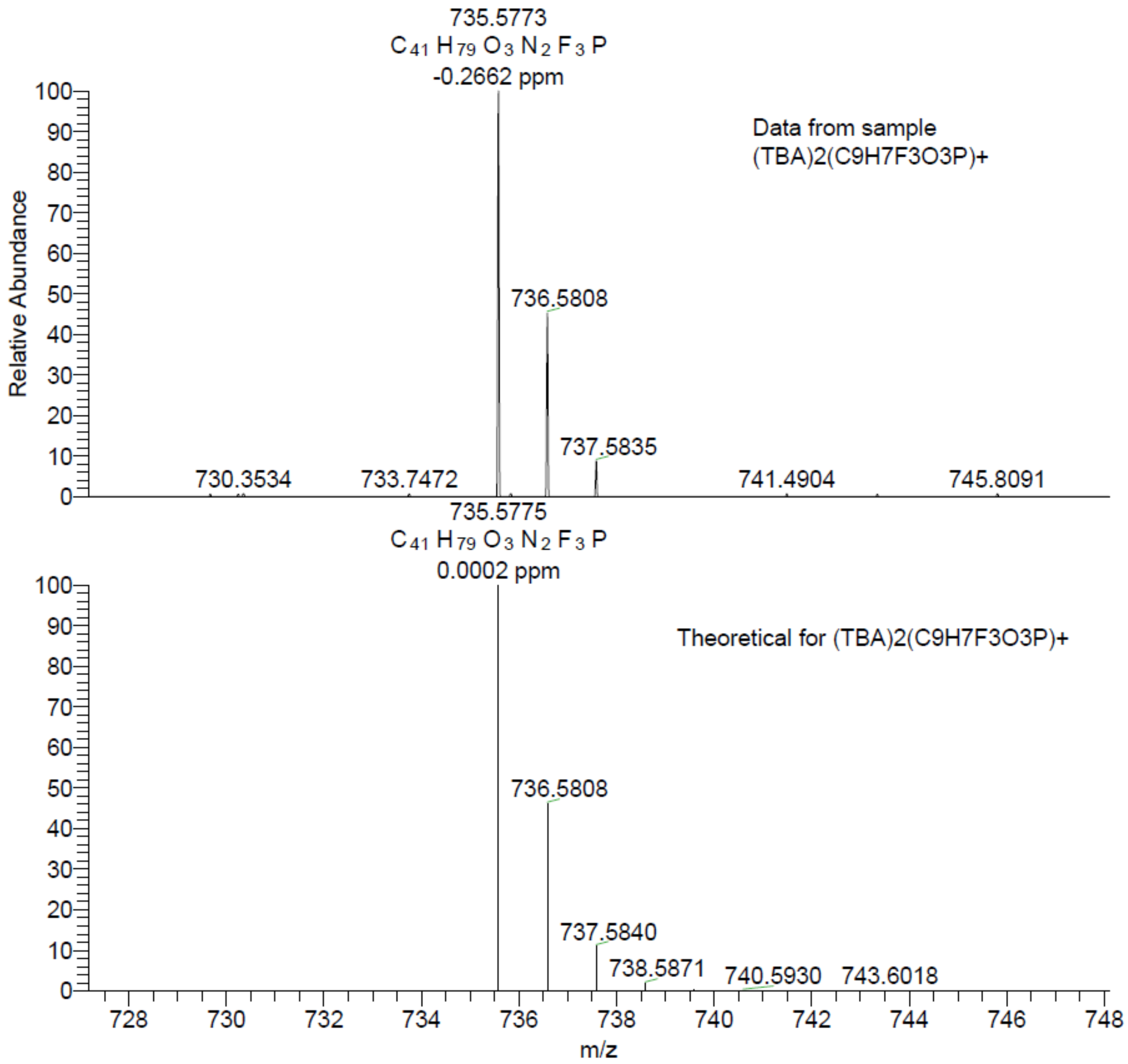

Figure S48. HRMS spectrum of tetrabutylammonium salt of 4-trifluoromethylbenzyl vinylphosphonate.

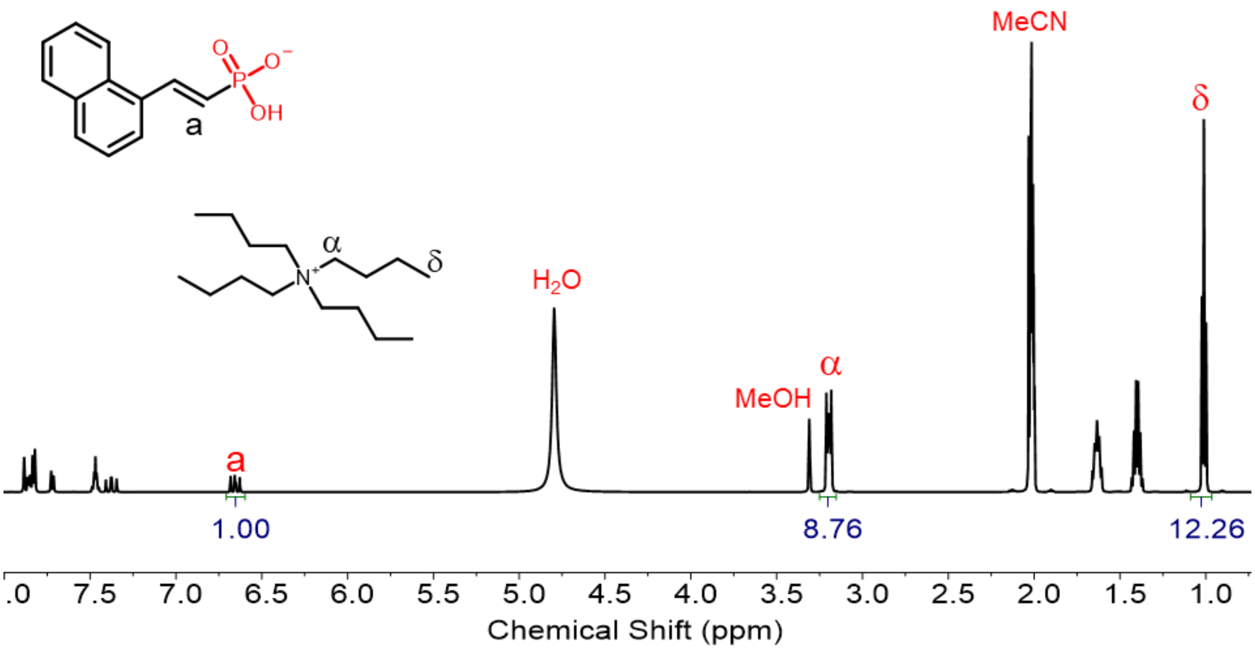

Figure S49. ^{1}H NMR spectrum of tetrabutylammonium salt of naphthyl vinylphosphonate. (CD_3OD-CD_3CN, 600 MHz, 298 K)

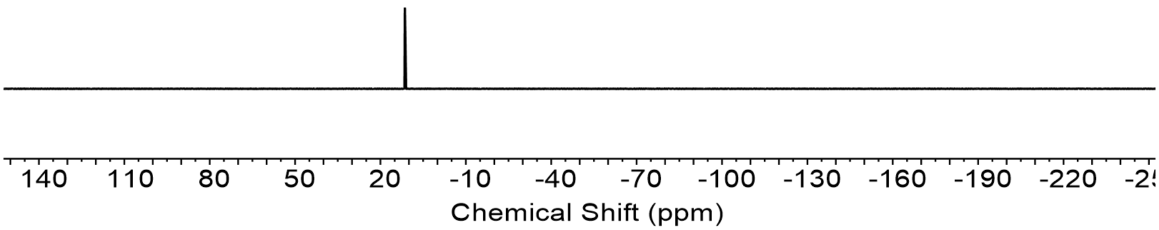

Figure S50. ^{31}P NMR spectrum of tetrabutylammonium salt of naphthyl vinylphosphonate, δ=11.44 ppm. (CD_3OD-CD_3CN, 202 MHz, 298 K)

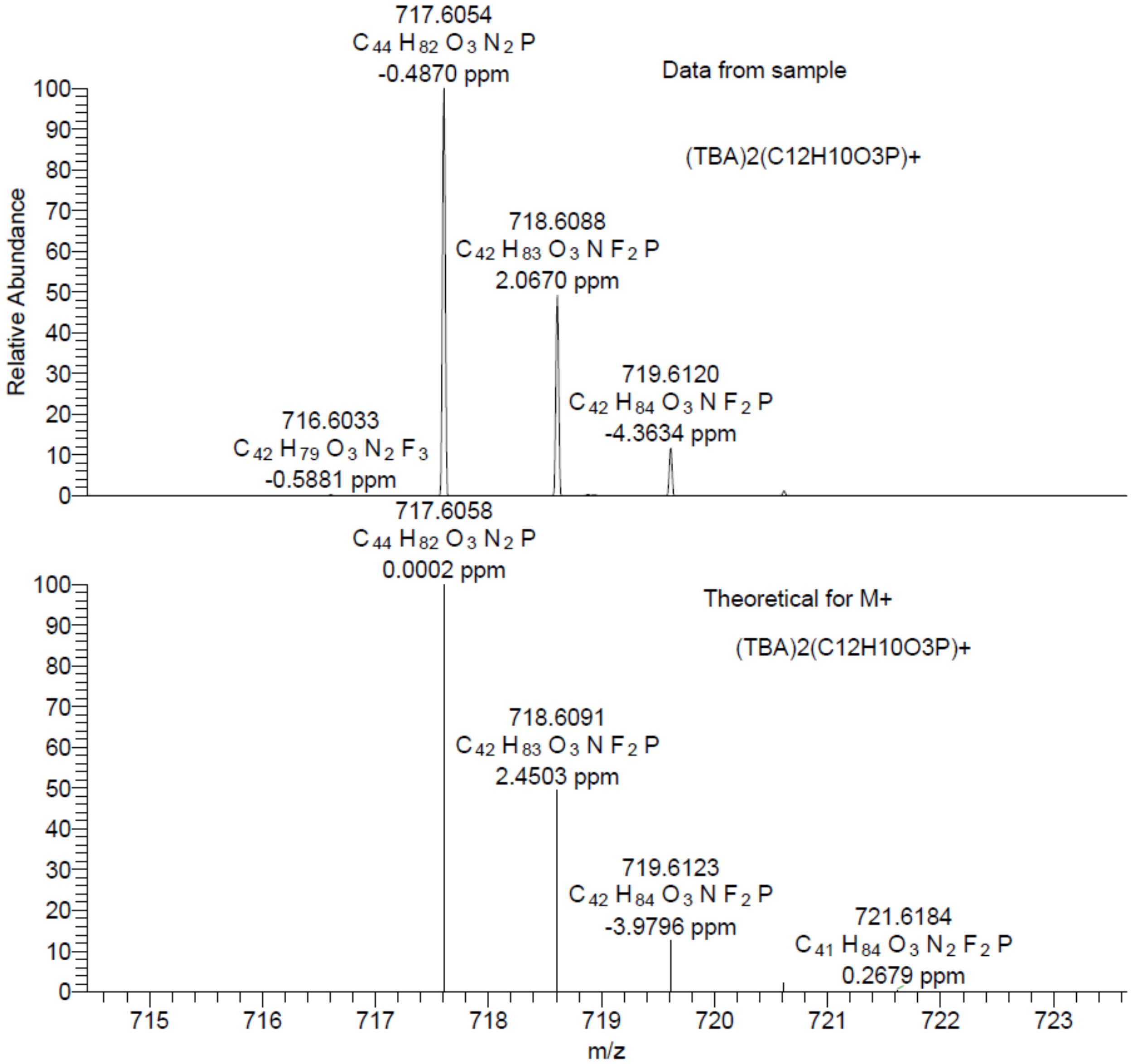

Figure S51. HRMS spectrum of tetrabutylammonium salt of naphthyl vinylphosphonate.

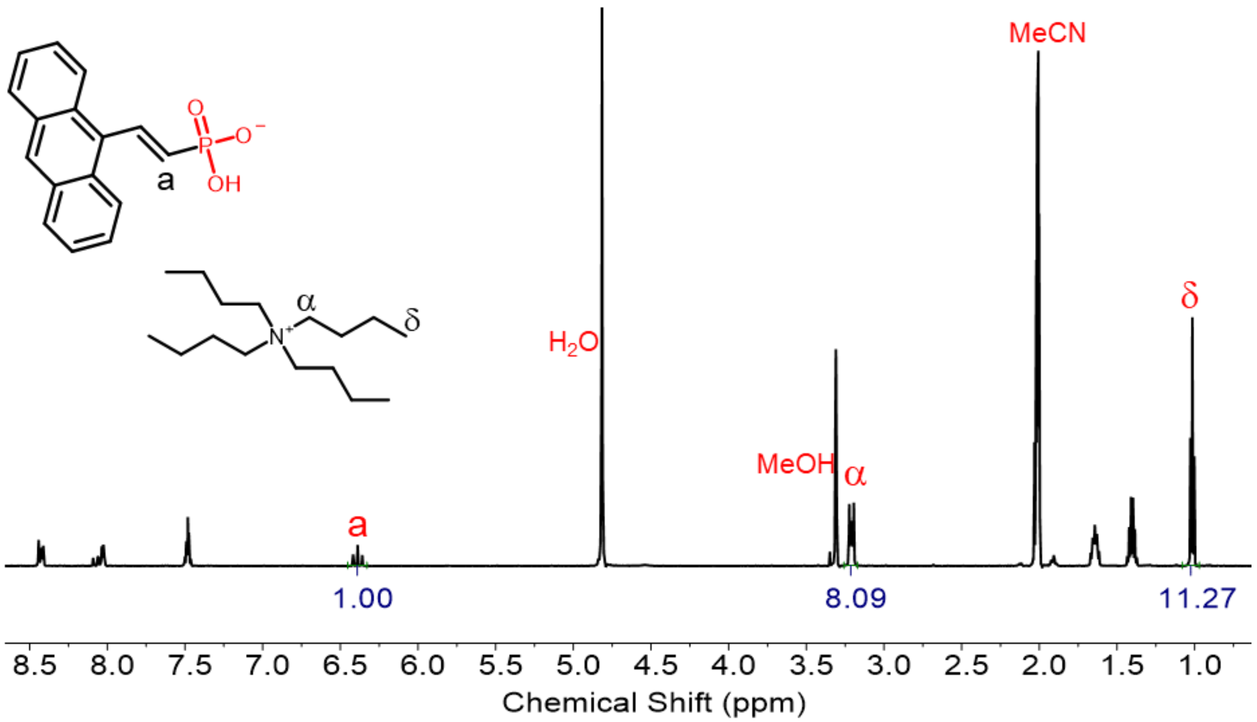

Figure S52. ^{1}H NMR spectrum of tetrabutylammonium salt of anthracene vinylphosphonate. (CD_3OD-CD_3CN, 600 MHz, 298 K)

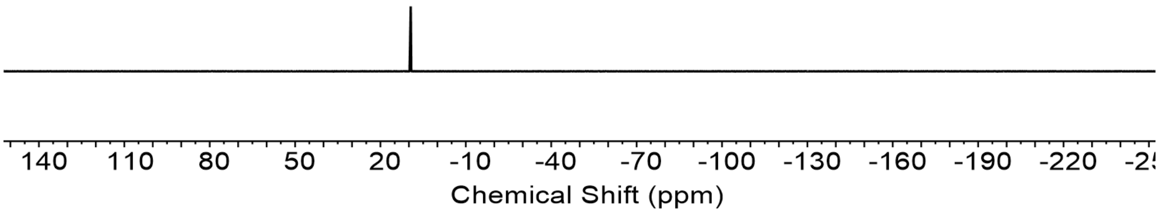

Figure S53. ^{31}P NMR spectrum of tetrabutylammonium salt of anthracene vinylphosphonate, δ=9.37 ppm. (CD_3OD-CD_3CN, 202 MHz, 298 K)

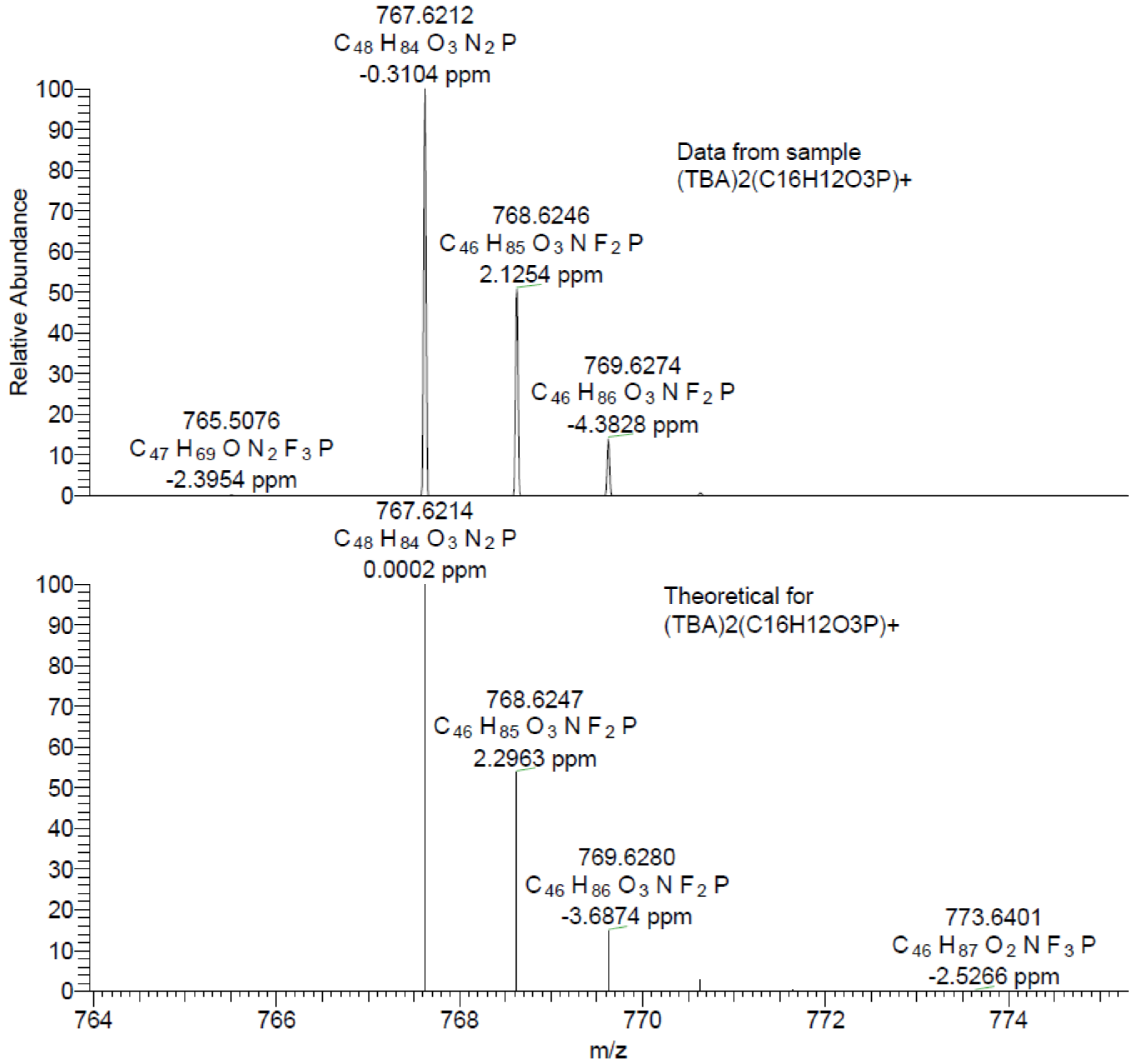

Figure S54. HRMS spectrum of tetrabutylammonium salt of anthracene vinylphosphonate.

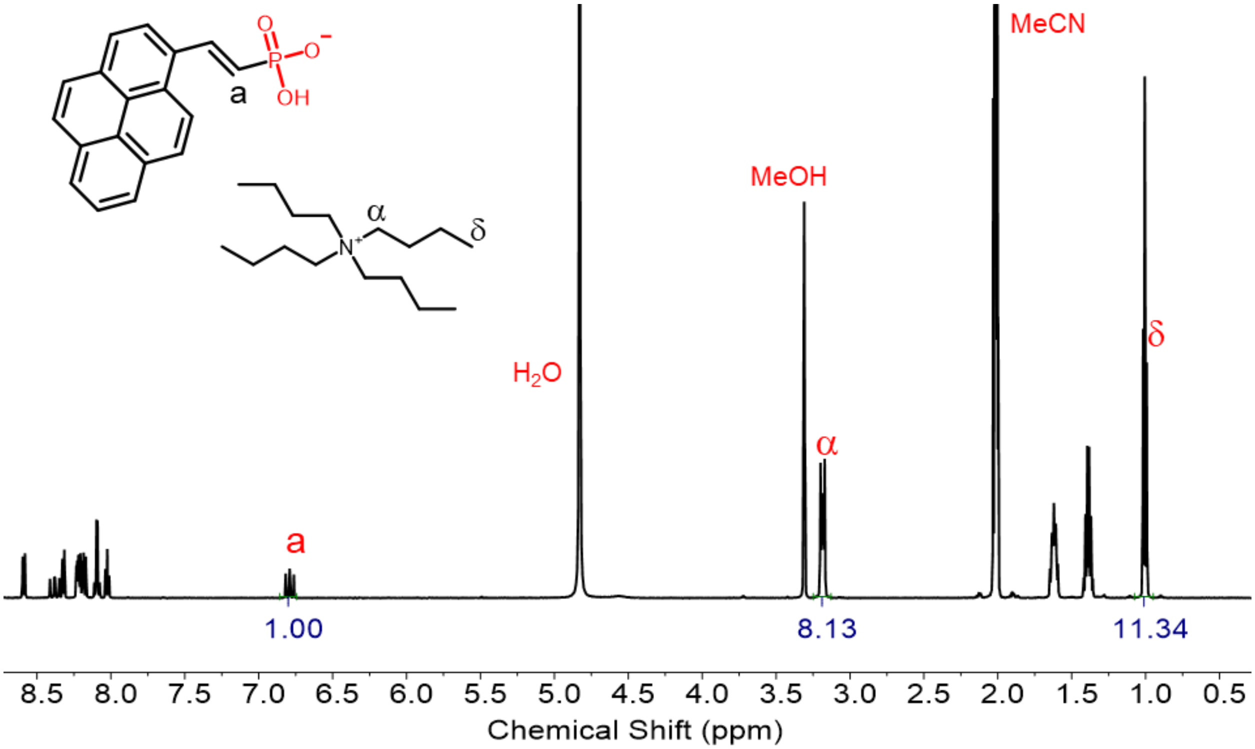

Figure S55. ^{1}H NMR spectrum of tetrabutylammonium salt of pyrene vinylphosphonate. (CD_3OD-CD_3CN, 600 MHz, 298 K)

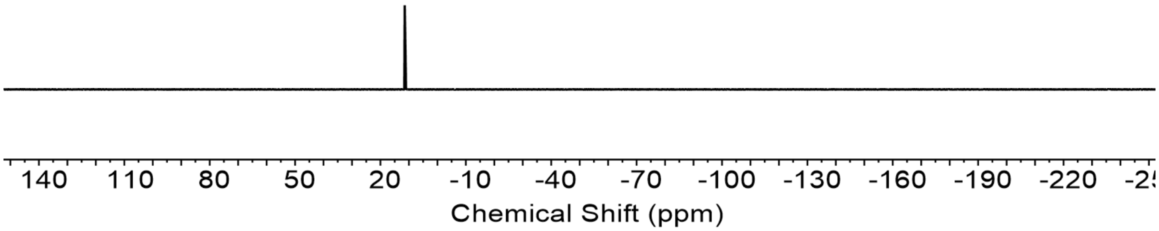

Figure S56. ^{31}P NMR spectrum of tetrabutylammonium salt of pyrene vinylphosphonate, δ=11.48 ppm. (CD_3OD-CD_3CN, 202 MHz, 298 K)

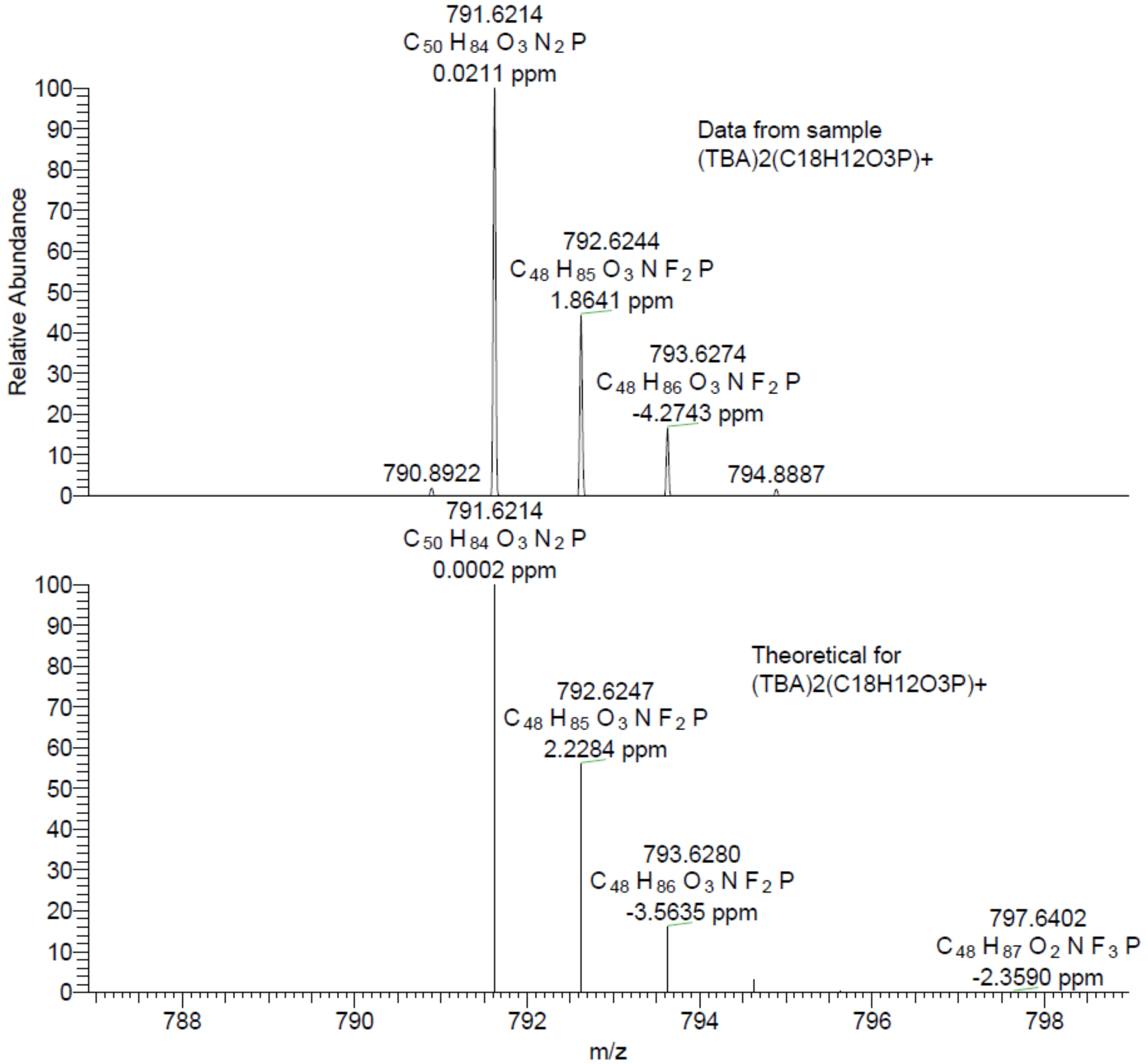

Figure S57. HRMS spectrum of tetrabutylammonium salt of pyrene vinylphosphonate.

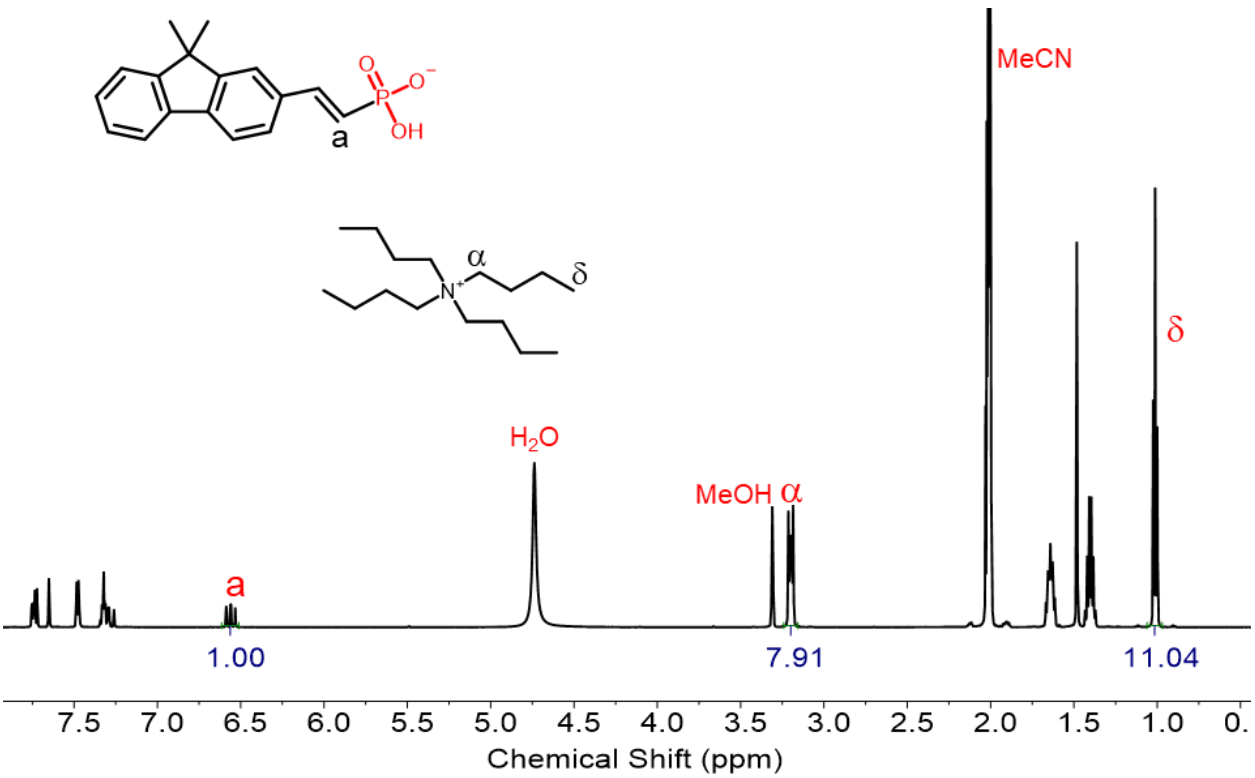

Figure S58. ^{1}H NMR spectrum of tetrabutylammonium salt of dimethyl fluorene vinylphosphonate. (CD_3OD-CD_3CN, 600 MHz, 298 K)

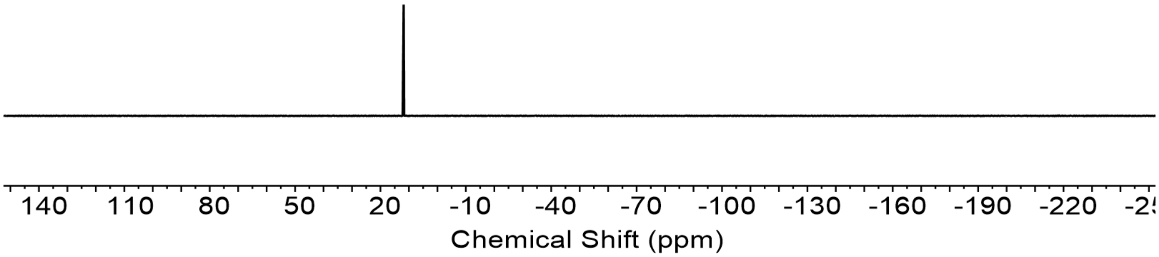

Figure S59. ^{31}P NMR spectrum of tetrabutylammonium salt of dimethyl fluorene vinylphosphonate, δ=11.86 ppm. (CD_3OD-CD_3CN, 202 MHz, 298 K)

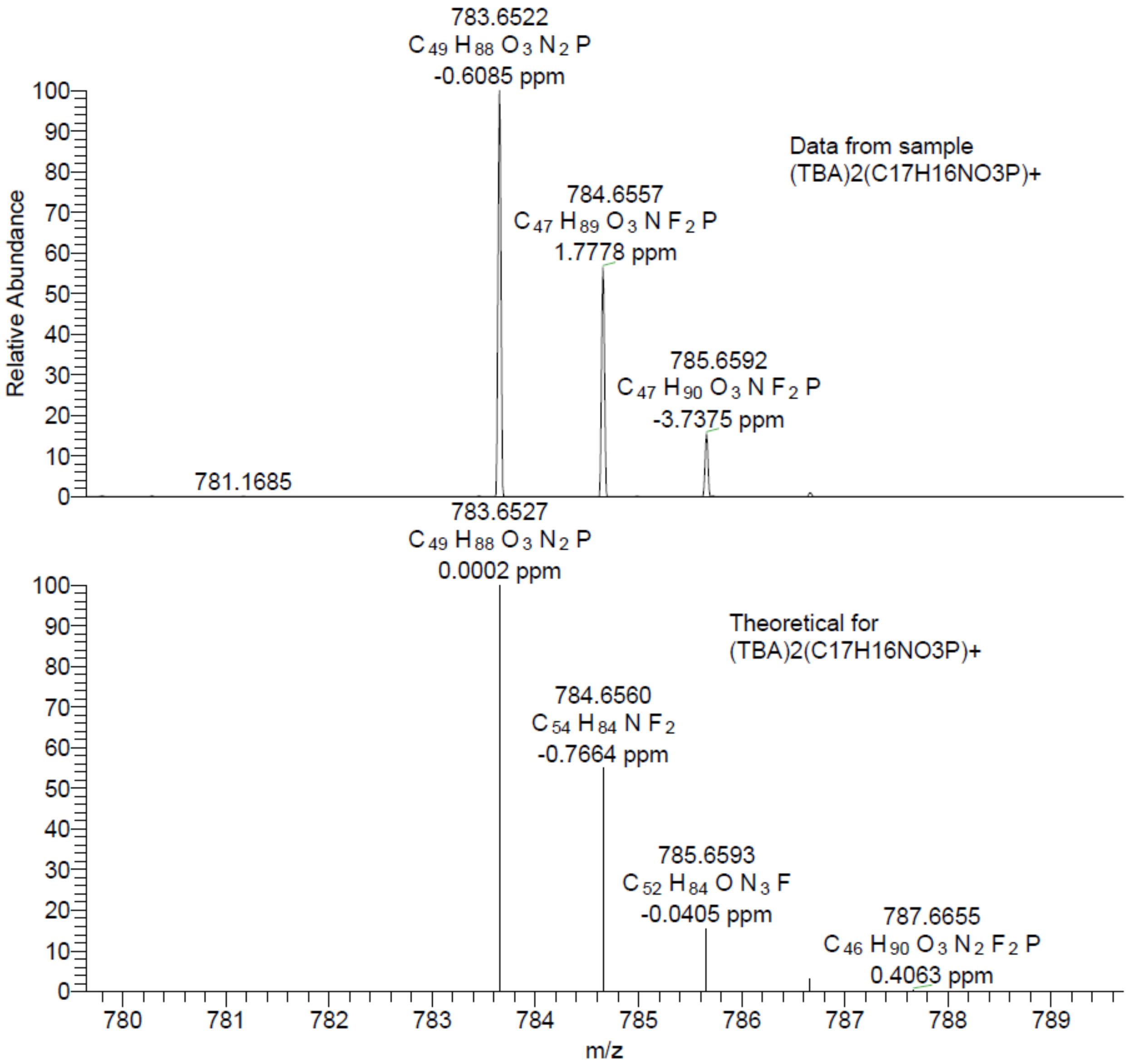

Figure S60. HRMS spectrum of tetrabutylammonium salt of dimethyl fluorene vinylphosphonate.

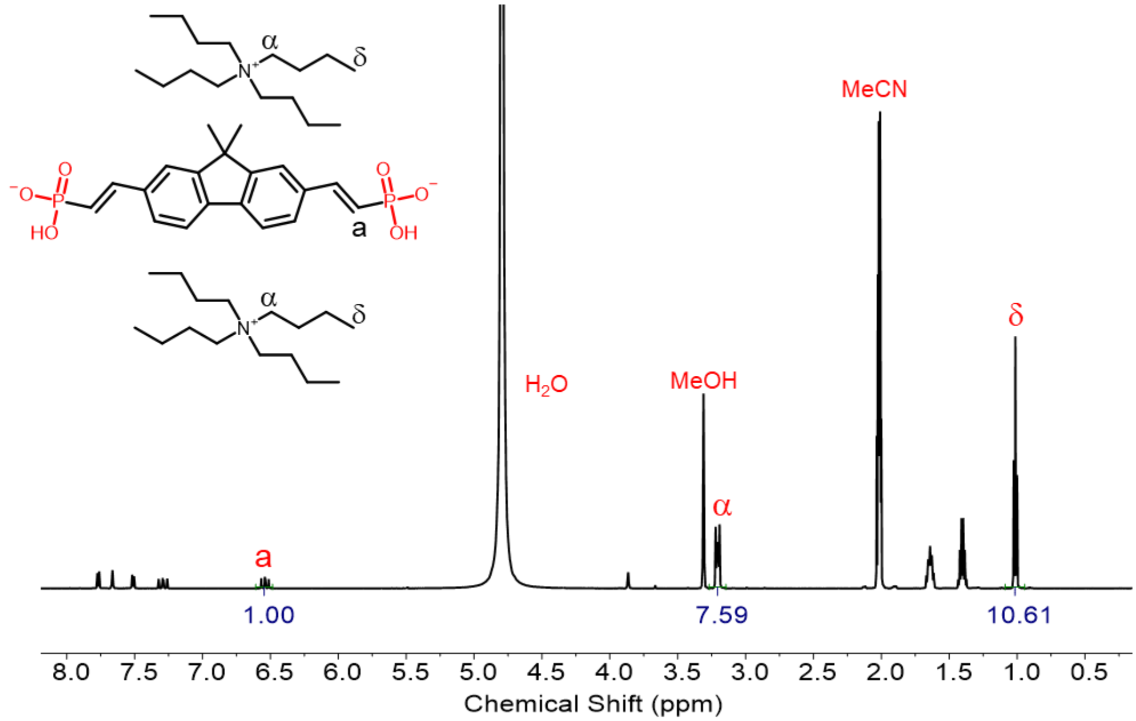

Figure S61. ^{1}H NMR spectrum of tetrabutylammonium salt of dimethyl fluorene bis(vinylphosphonate). (CD_3OD-CD_3CN, 600 MHz, 298 K)

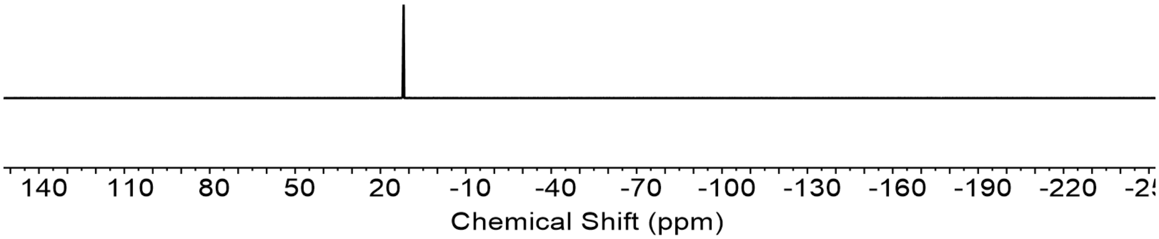

Figure S62. ^{31}P NMR spectrum of tetrabutylammonium salt of dimethyl fluorene bis(vinylphosphonate), δ=11.87 ppm. (CD_3OD-CD_3CN, 202 MHz, 298 K)

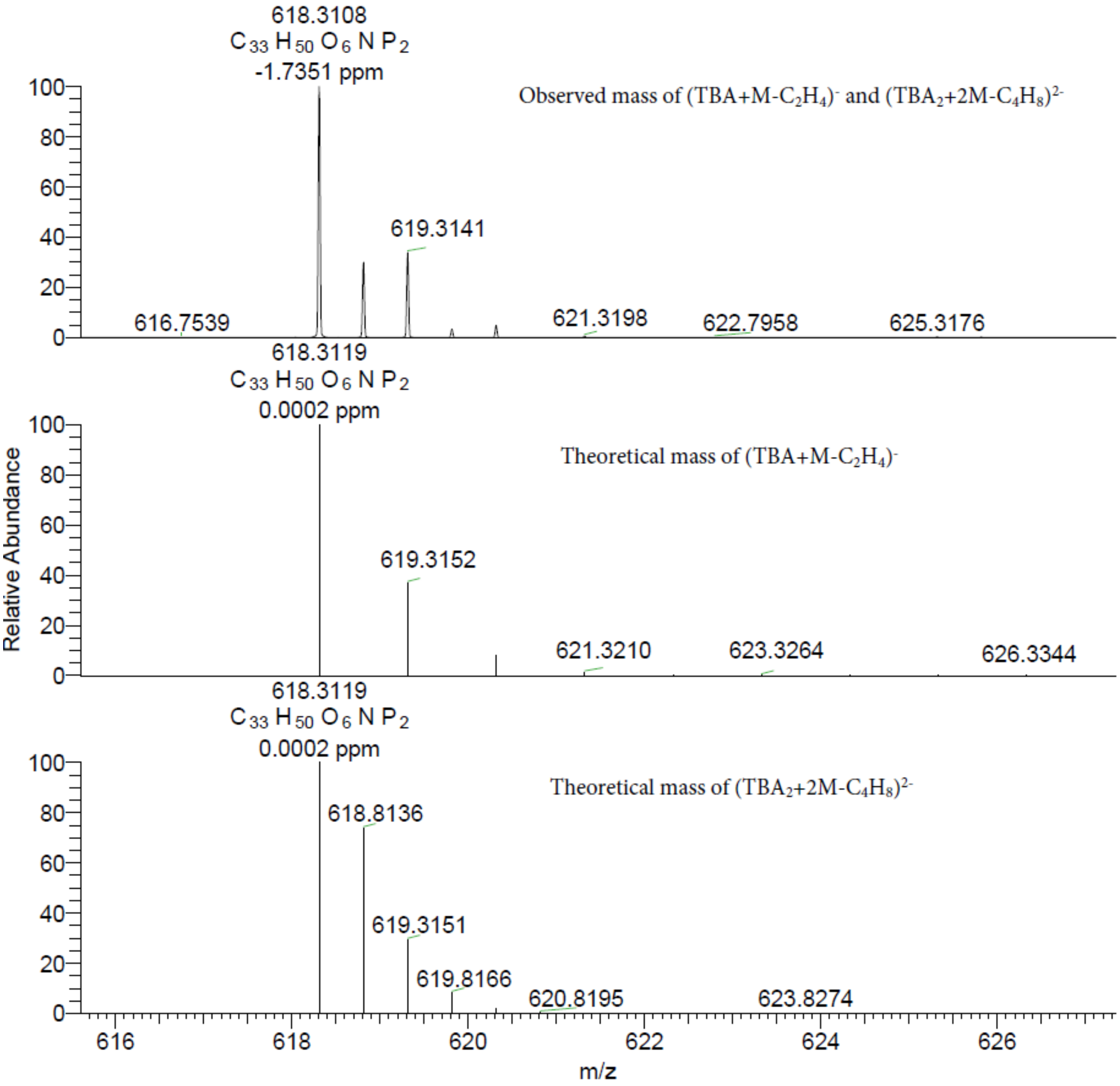

Figure S63. HRMS spectrum of tetrabutylammonium salt of dimethyl fluorene bis(vinylphosphonate).

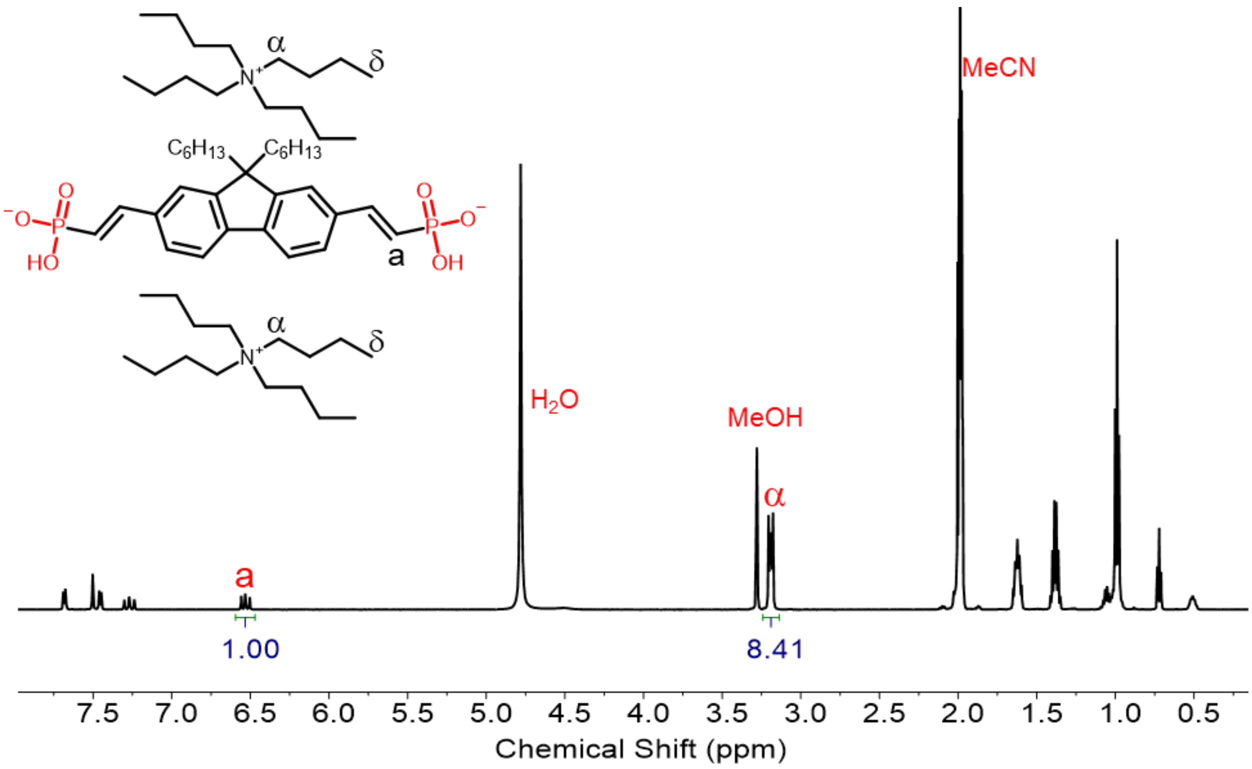

Figure S64. ^{1}H NMR spectrum of tetrabutylammonium salt of dihexyl fluorene bis(vinylphosphonate). (CD_3OD-CD_3CN, 600 MHz, 298 K)

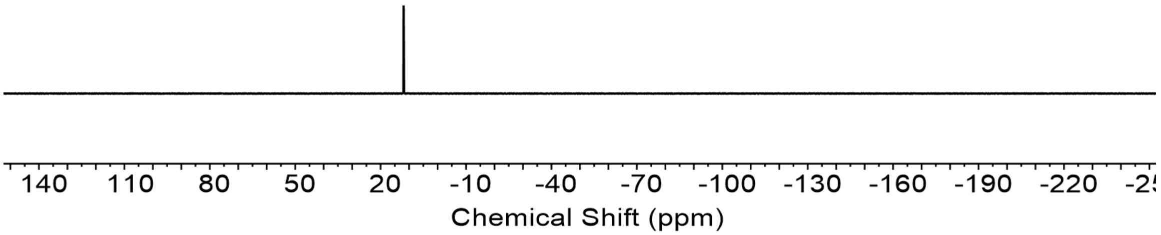

Figure S65. ^{31}P NMR spectrum of tetrabutylammonium salt of dihexyl fluorene bis(vinylphosphonate), δ=11.95 ppm. (CD_3OD-CD_3CN, 202 MHz, 298 K)

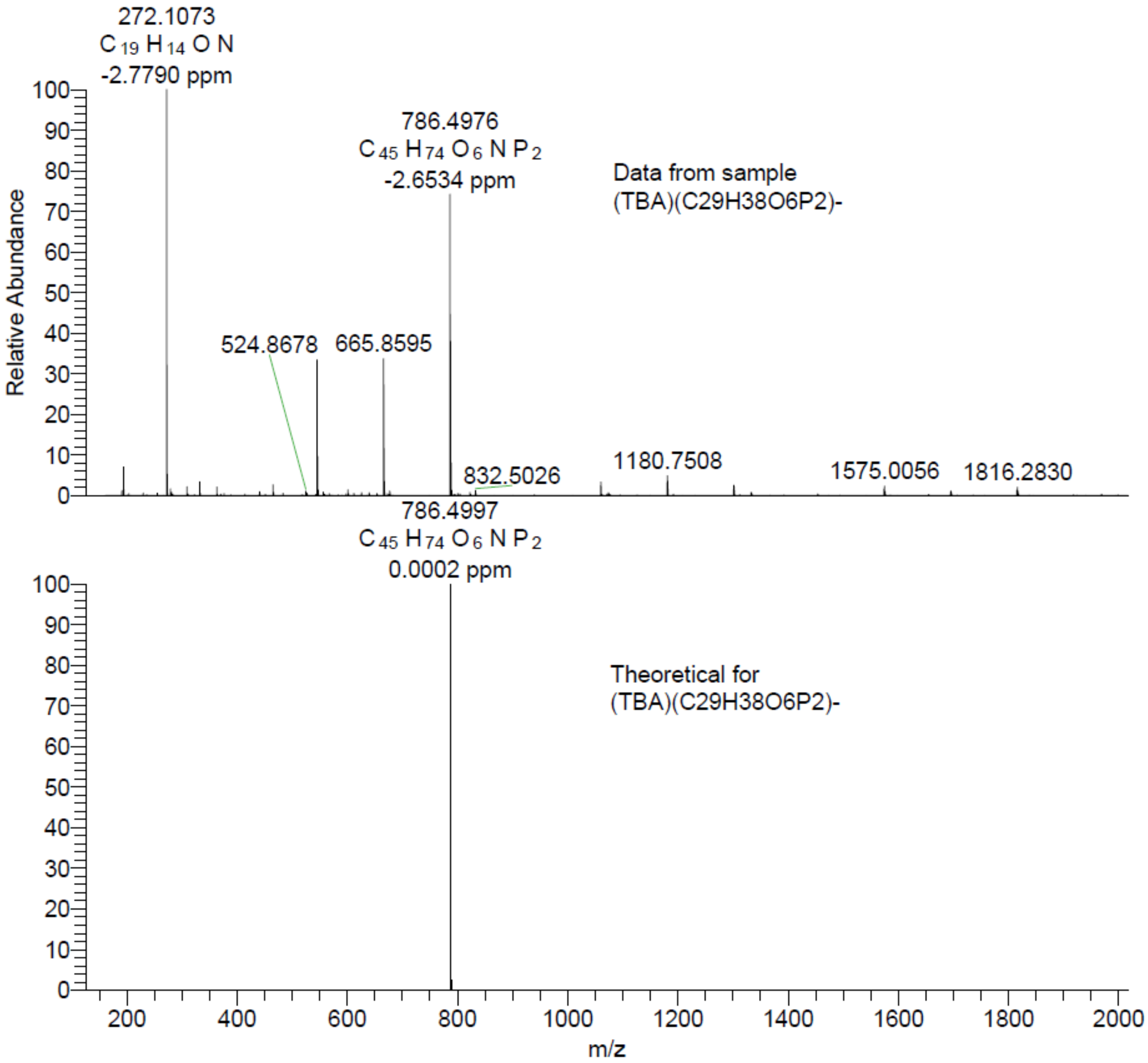

Figure S66. HRMS spectrum of tetrabutylammonium salt of dihexyl fluorene bis(vinylphosphonate).

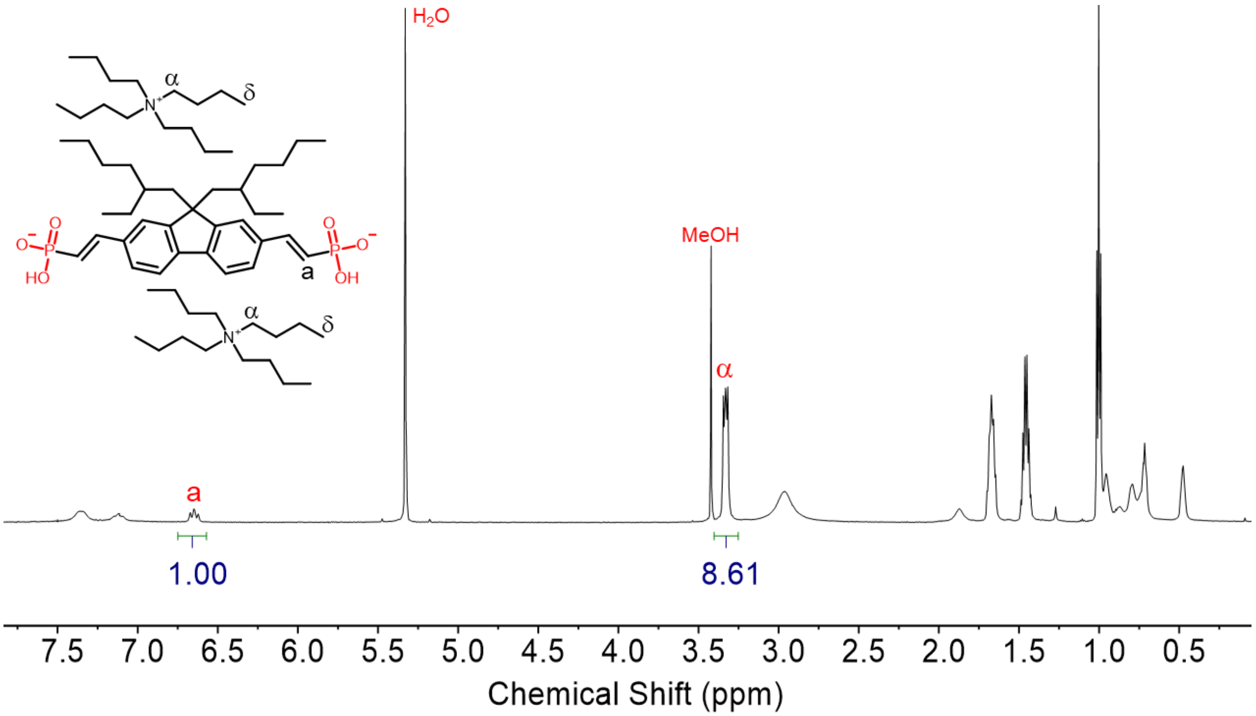

Figure S67. ^{1}H NMR spectrum of tetrabutylammonium salt of di(2-ethylhexyl) fluorene bis(vinylphosphonate). (CD_3OD, 600 MHz, 298 K)

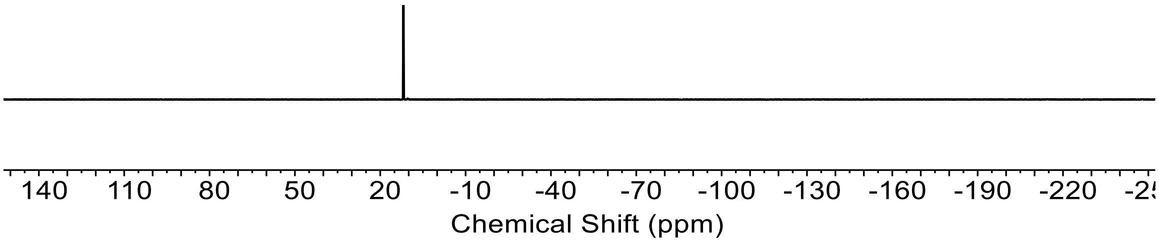

Figure S68. ^{31}P NMR spectrum of tetrabutylammonium salt of di(2-ethylhexyl) fluorene bis(vinylphosphonate), δ=11.85 ppm. (CD_3OD-CD_3CN, 202 MHz, 298 K)

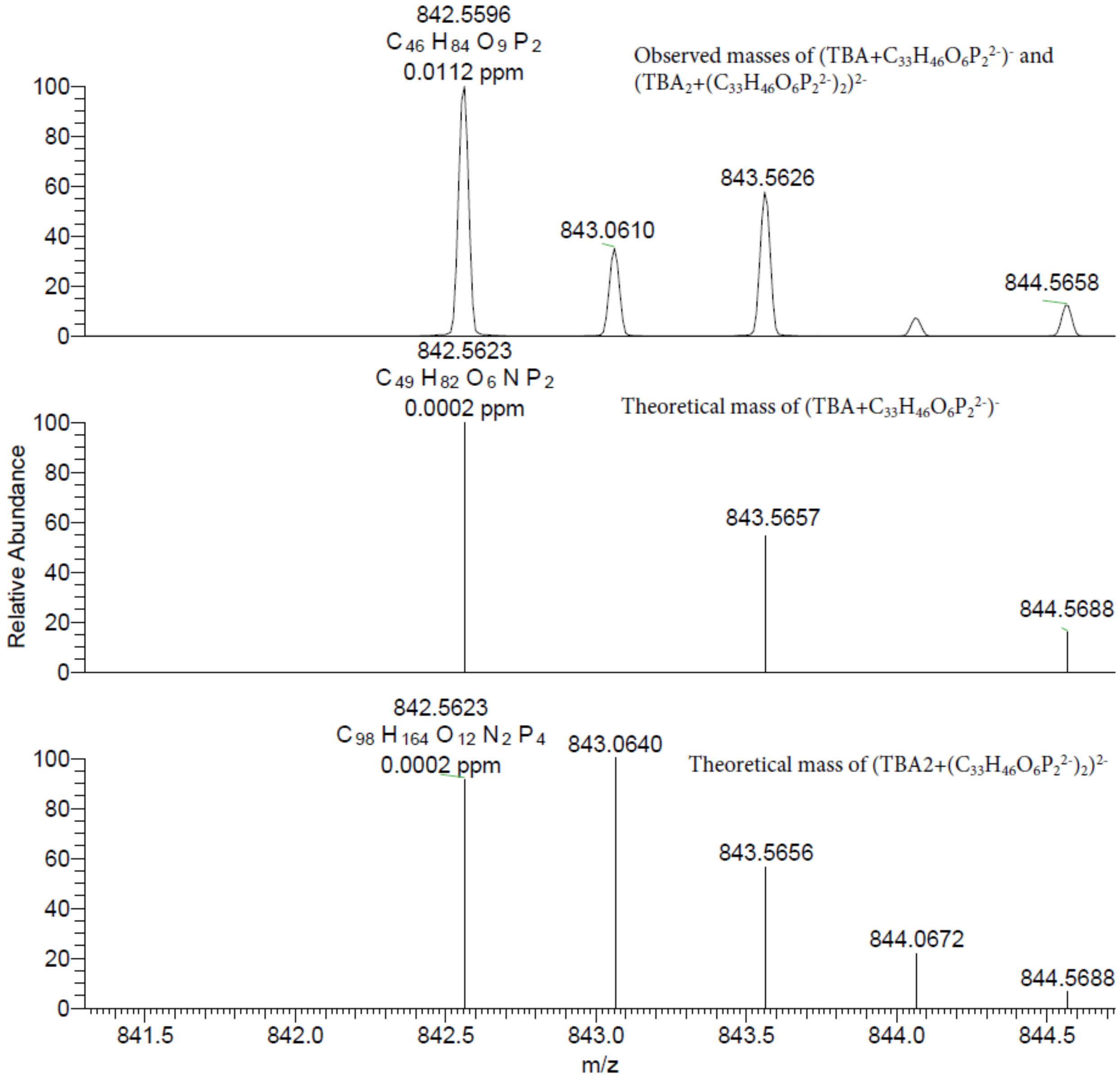

Figure S69. HRMS spectrum of tetrabutylammonium salt of di(2-ethylhexyl) fluorene bis(vinylphosphonate).

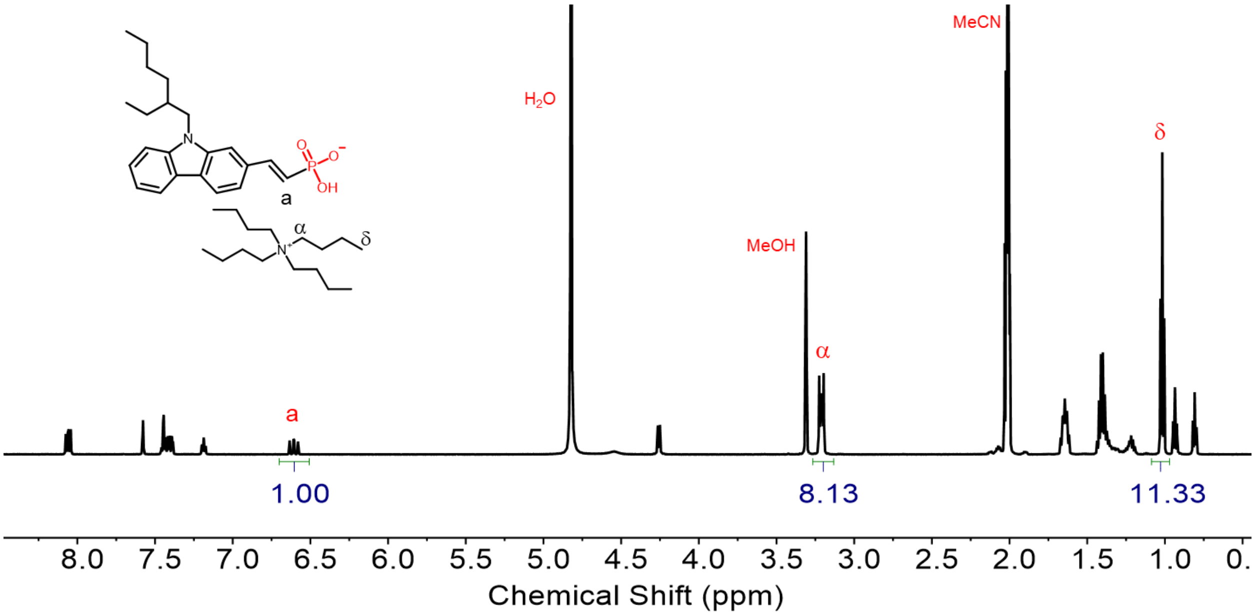

Figure S70. ^{1}H NMR spectrum of tetrabutylammonium salt of 2-ethylhexyl carbazole vinylphosphonate. (CD_3OD-CD_3CN, 600 MHz, 298 K)

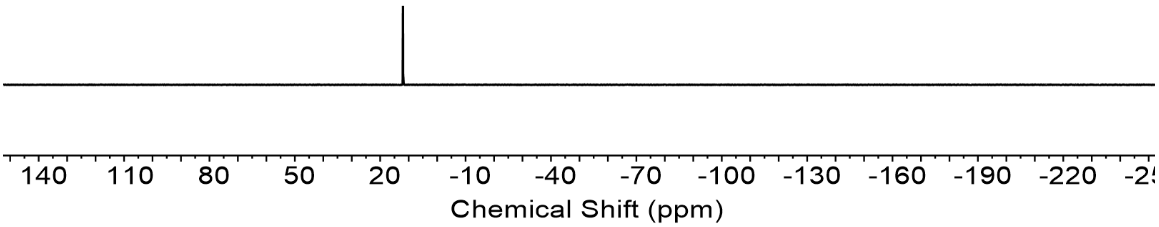

Figure S71. ^{31}P NMR spectrum of tetrabutylammonium salt of 2-ethylhexyl carbazole vinylphosphonate, δ=12.00 ppm. (CD_3OD-CD_3CN, 202 MHz, 298 K)

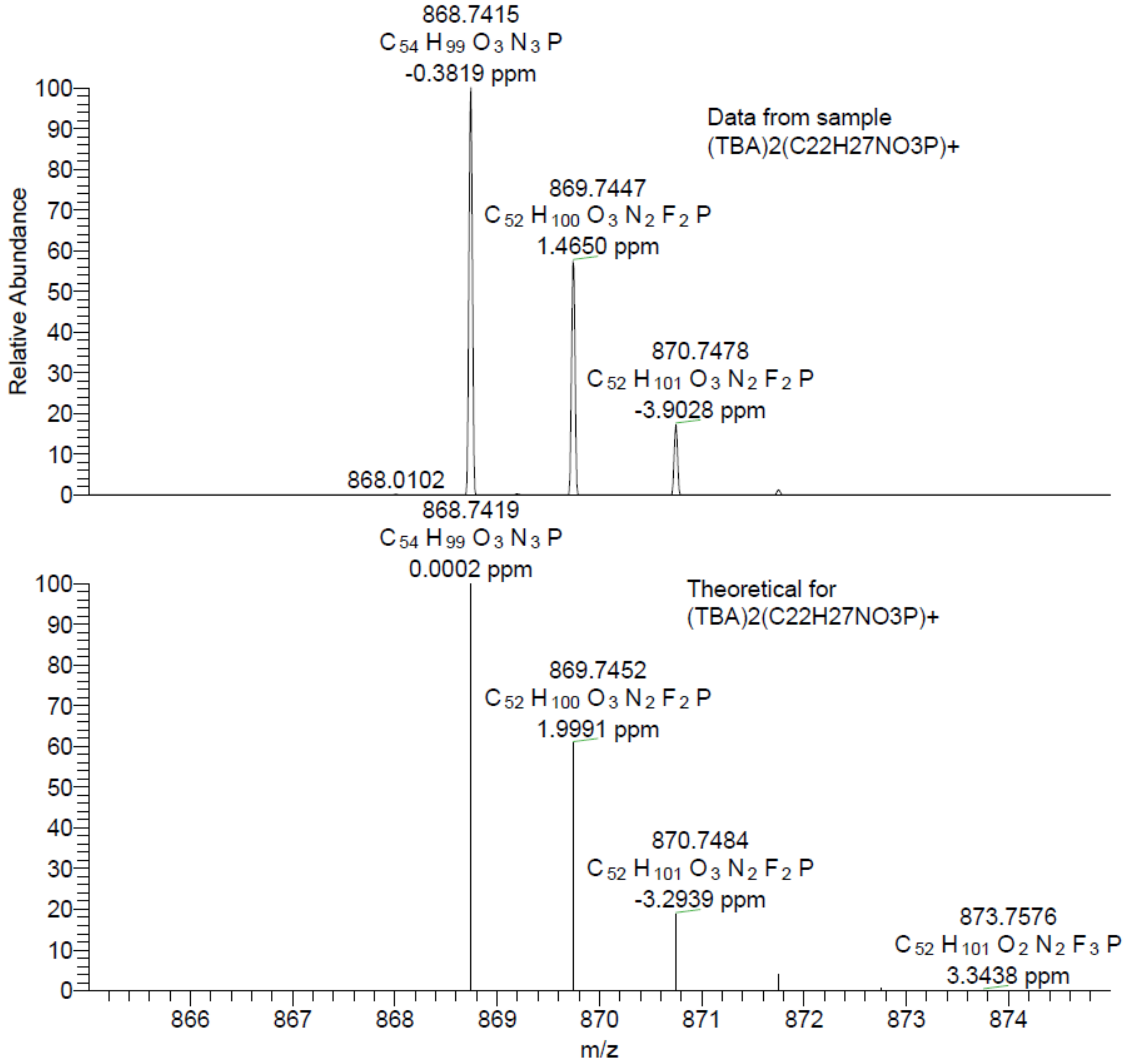

Figure S72. HRMS spectrum of tetrabutylammonium salt of 2-ethylhexyl carbazole vinylphosphonate.

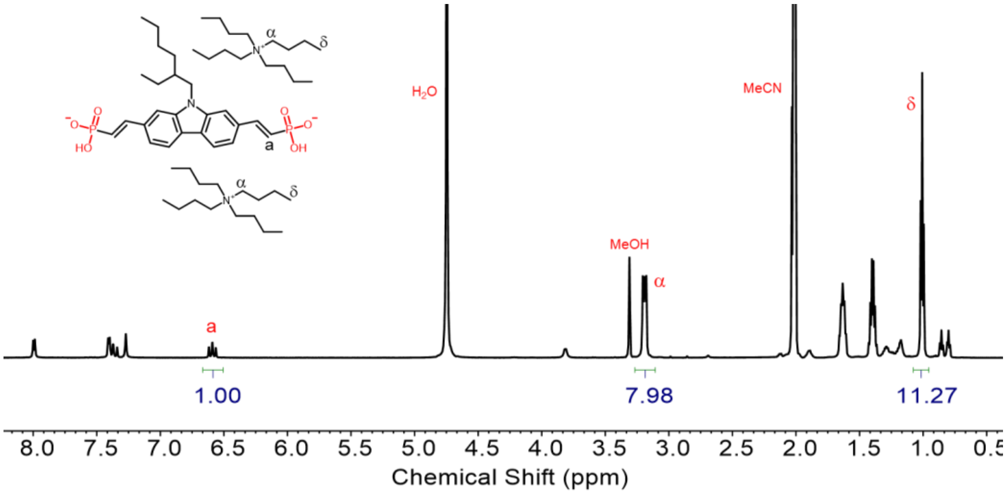

Figure S73. ^{1}H NMR spectrum of tetrabutylammonium salt of 2-ethylhexyl carbazole bis(vinylphosphonate). (CD_3OD-CD_3CN, 600 MHz, 298 K)

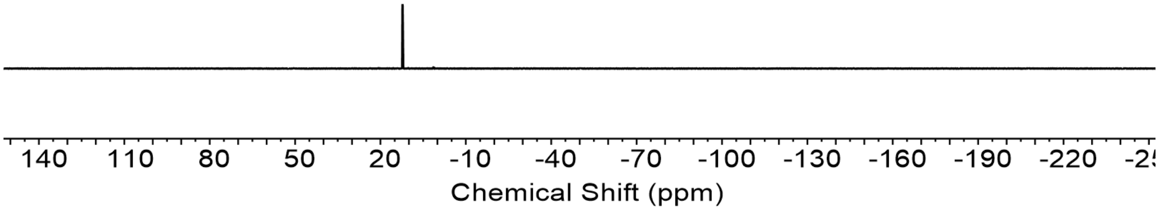

Figure S74. ^{31}P NMR spectrum of tetrabutylammonium salt of 2-ethylhexyl carbazole bis(vinylphosphonate), δ=12.25 ppm. (CD_3OD-CD_3CN, 202 MHz, 298 K)

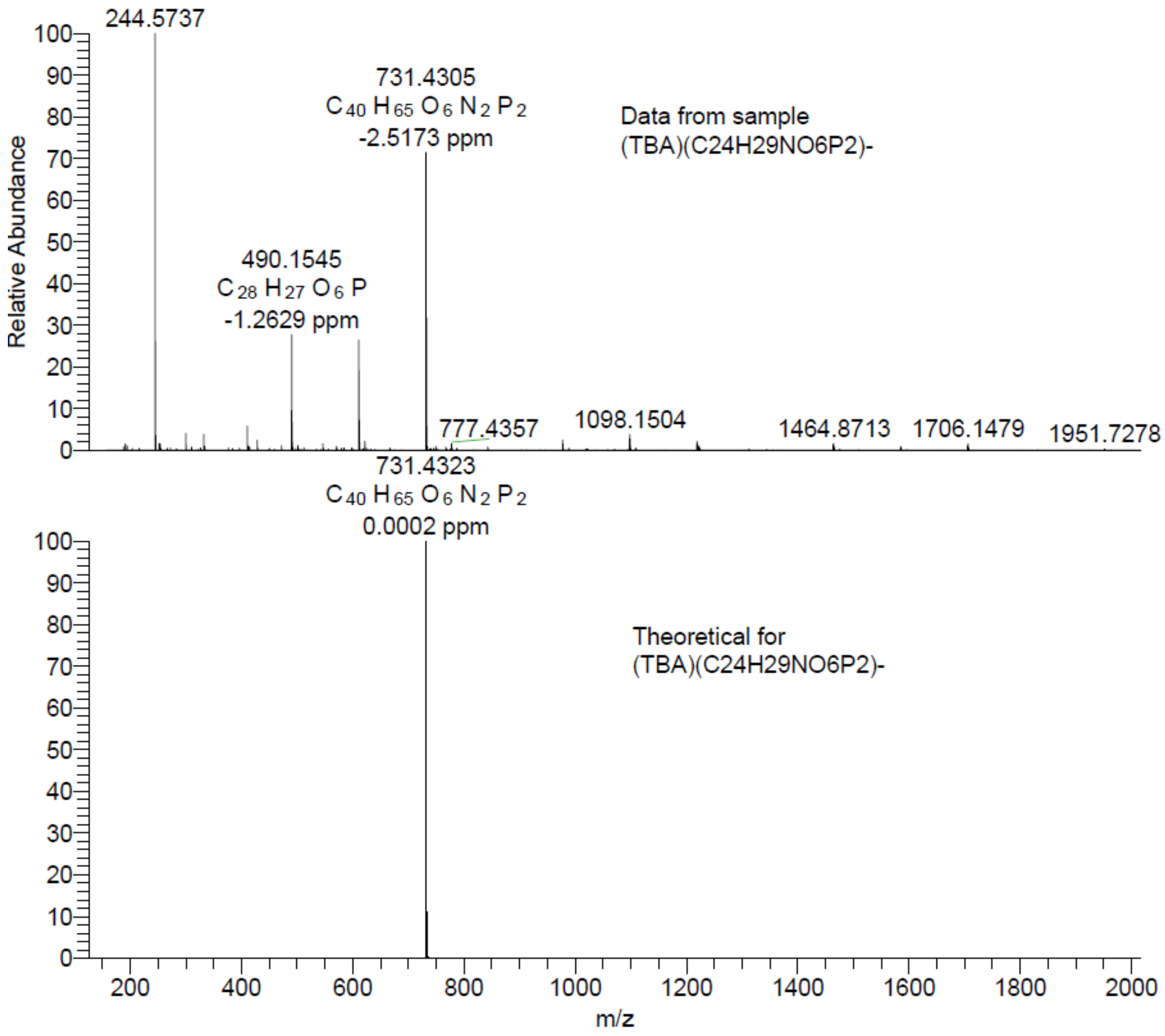

Figure S75. HRMS spectrum of tetrabutylammonium salt of 2-ethylhexyl carbazole bis(vinylphosphonate).

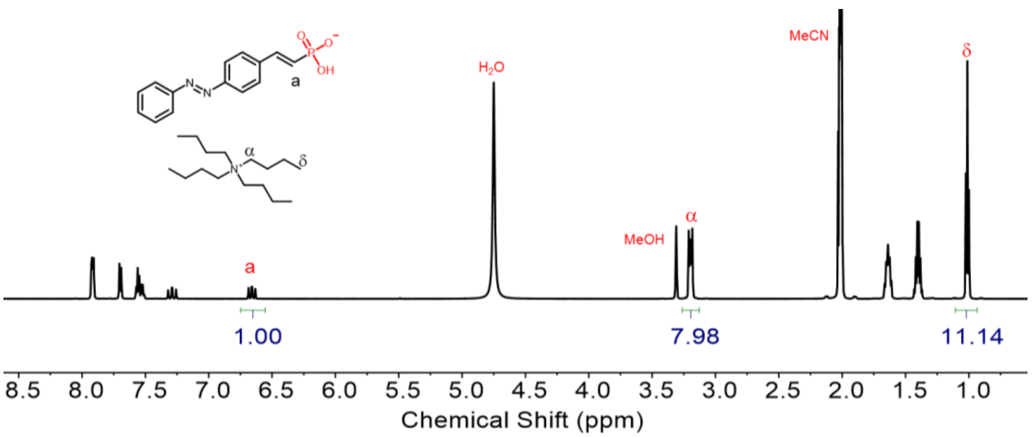

Figure S76. ^{1}H NMR spectrum of tetrabutylammonium salt of azobenzene vinylphosphonate. (CD_3OD-CD_3CN, 600 MHz, 298 K)

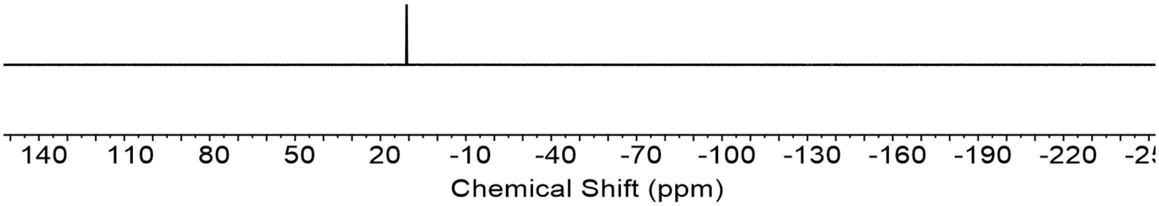

Figure S77. ^{31}P NMR spectrum of tetrabutylammonium salt of azobenzene vinylphosphonate, δ=10.82 ppm. (CD_3OD-CD_3CN, 202 MHz, 298 K)

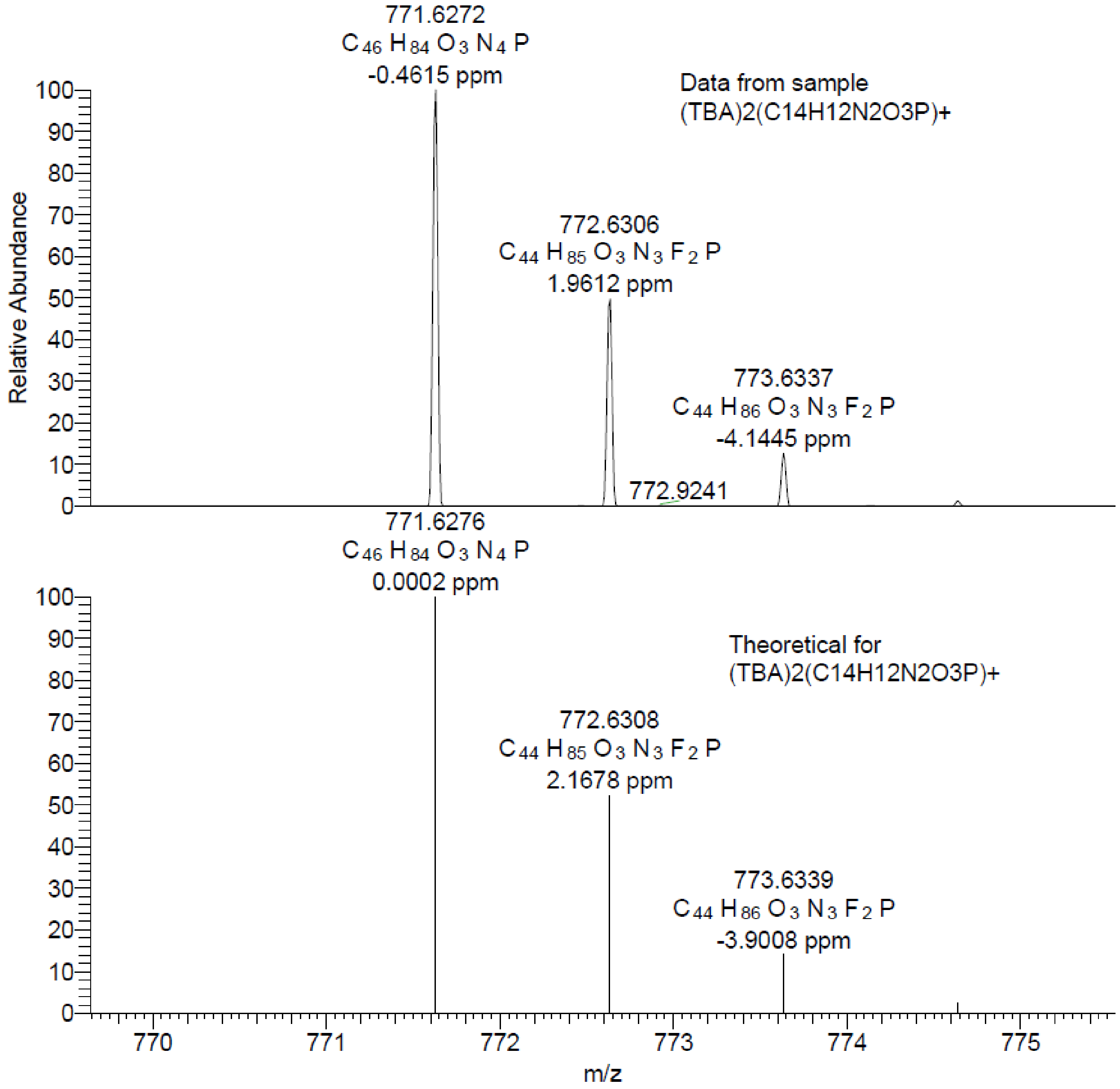

Figure S78. HRMS spectrum of tetrabutylammonium salt of azobenzene vinylphosphonate.

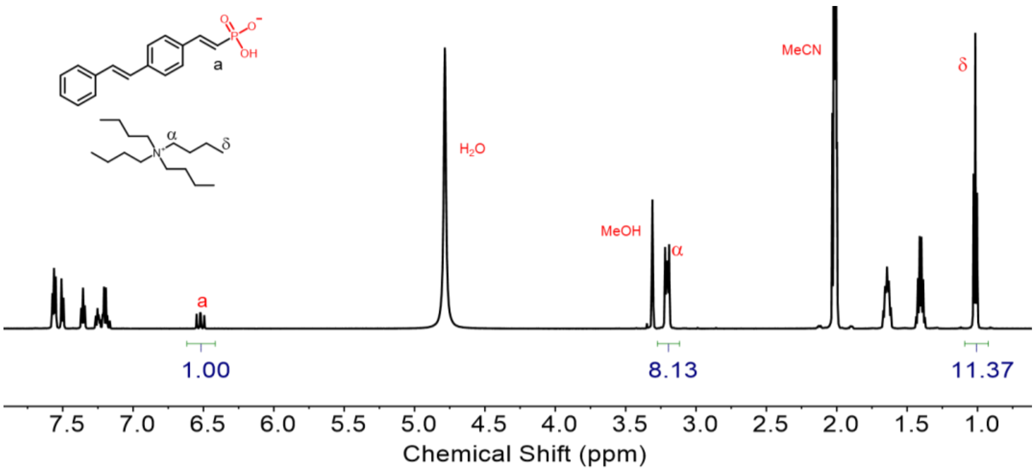

Figure S79. ^{1}H NMR spectrum of tetrabutylammonium salt of stilbene vinylphosphonate. (CD_3OD-CD_3CN, 600 MHz, 298 K)

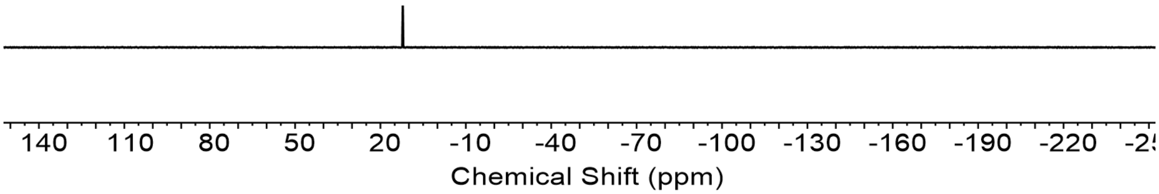

Figure S80. ^{31}P NMR spectrum of tetrabutylammonium salt of stilbene vinylphosphonate, δ=12.17 ppm. (CD_3OD-CD_3CN, 202 MHz, 298 K)

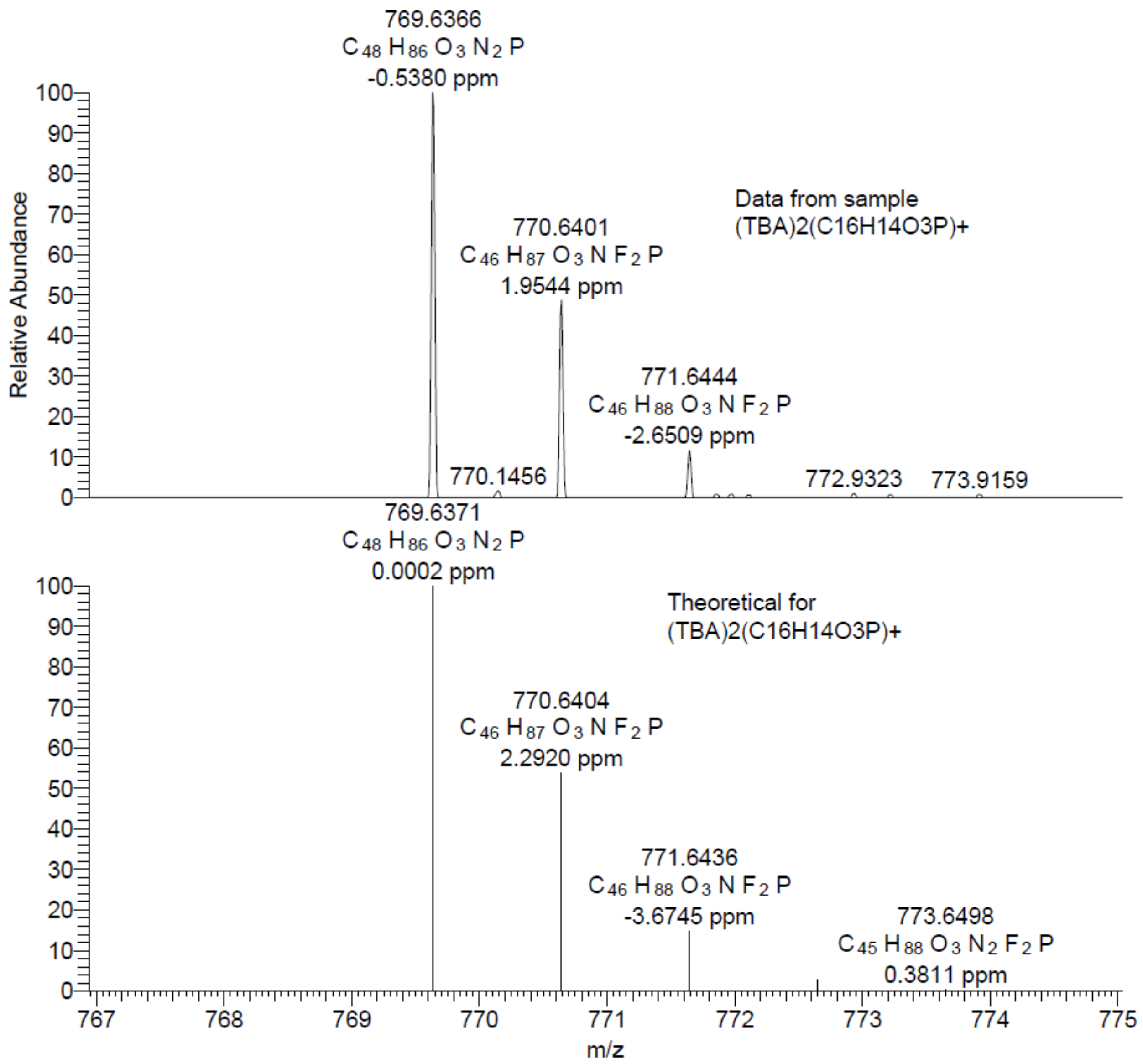

Figure S81. HRMS spectrum of tetrabutylammonium salt of stilbene vinylphosphonate.

S4. ^{1}H NMR Spectra of Titration Experiments

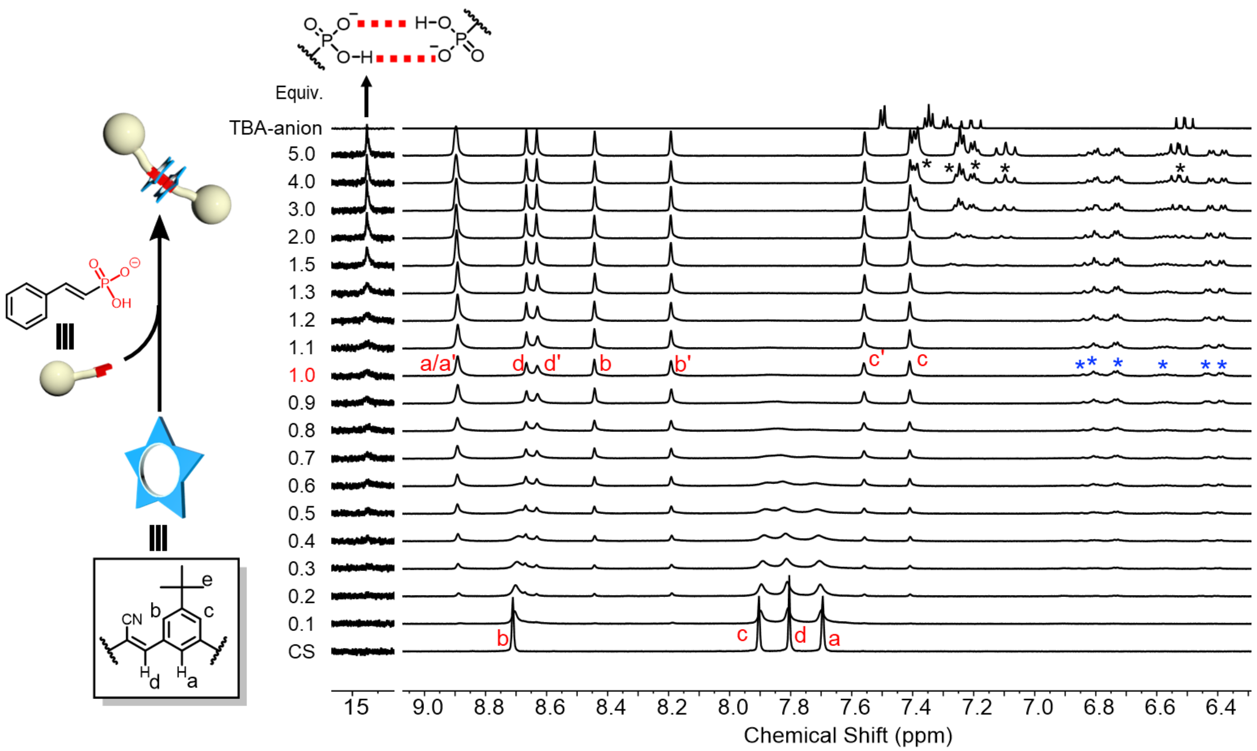

Figure S82. ^{1}H NMR titration of phenyl vinylphosphonate into 1 mM cyanostar solution. (CD_2Cl_2, 600 MHz, 298 K) Peaks marked with blue asterisks are complexed anions and black asterisks mark excess anions.

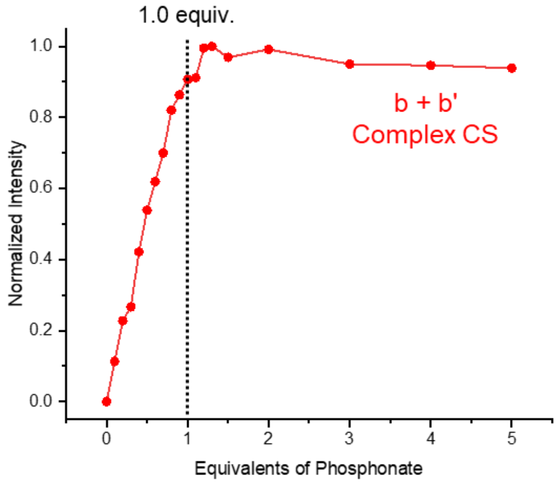

Figure S83. Proton intensity versus equivalents of complexed cyanostar in the titration of phenyl vinylphosphonate into 1 mM cyanostar solution.

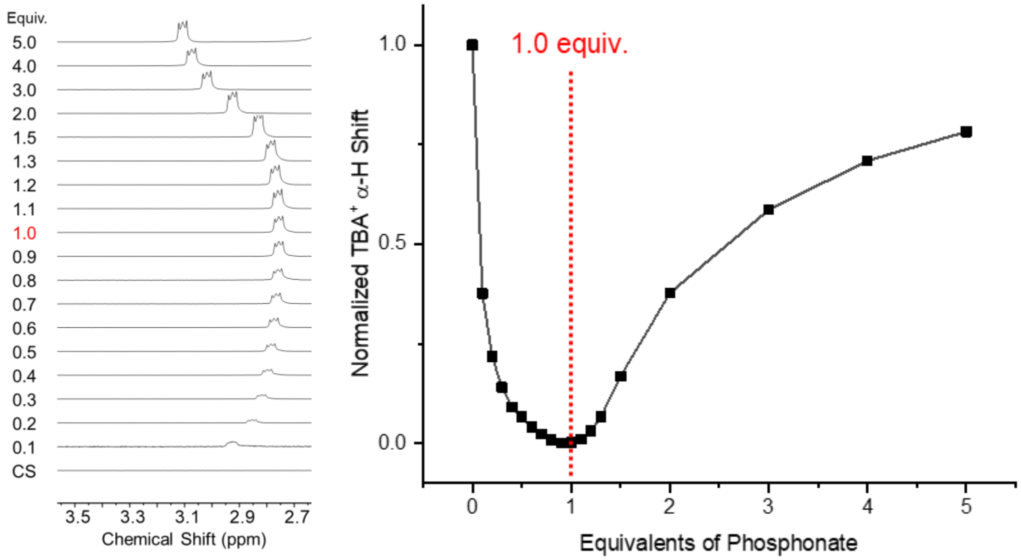

Figure S84. α-Proton shift of TBA^+ versus equivalents of anion in the titration of phenyl vinylphosphonate into 1 mM cyanostar solution.

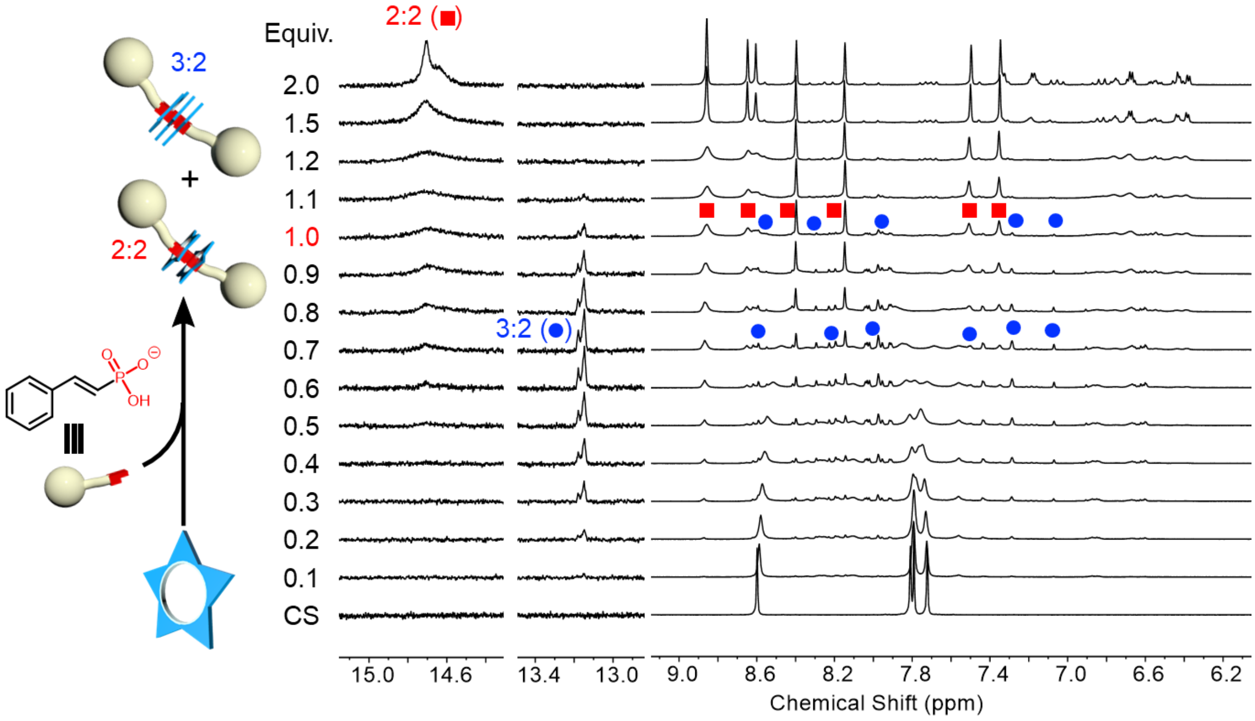

Figure S85. ^{1}H NMR titration of phenyl vinylphosphonate into 5 mM cyanostar solution. (2:1 *v/v* CD_2Cl_2:CD_3CN, 600 MHz, 298 K)

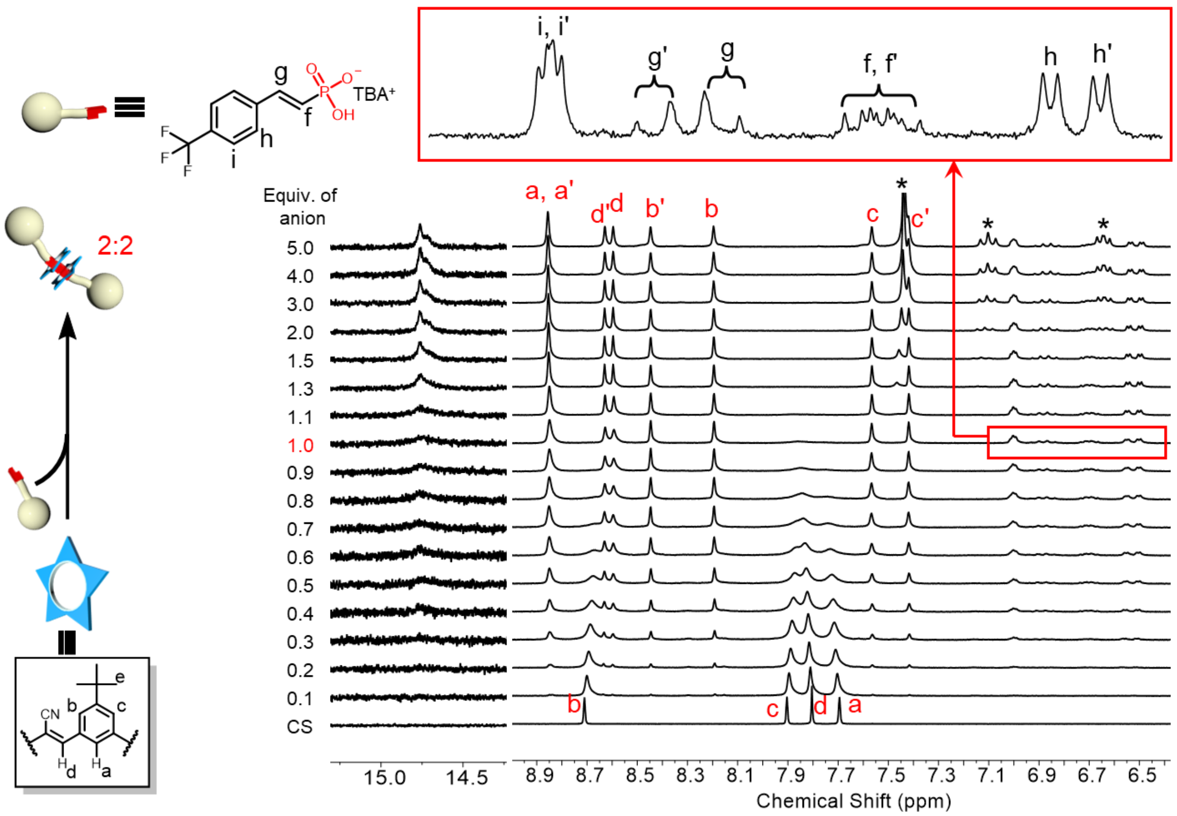

Figure S86. ^{1}H NMR titration of 4-trifluoromethylbenzyl vinylphosphonate into 1 mM cyanostar solution. (CD_2Cl_2, 600 MHz, 298 K) Peaks marked with asterisk are excess anions.

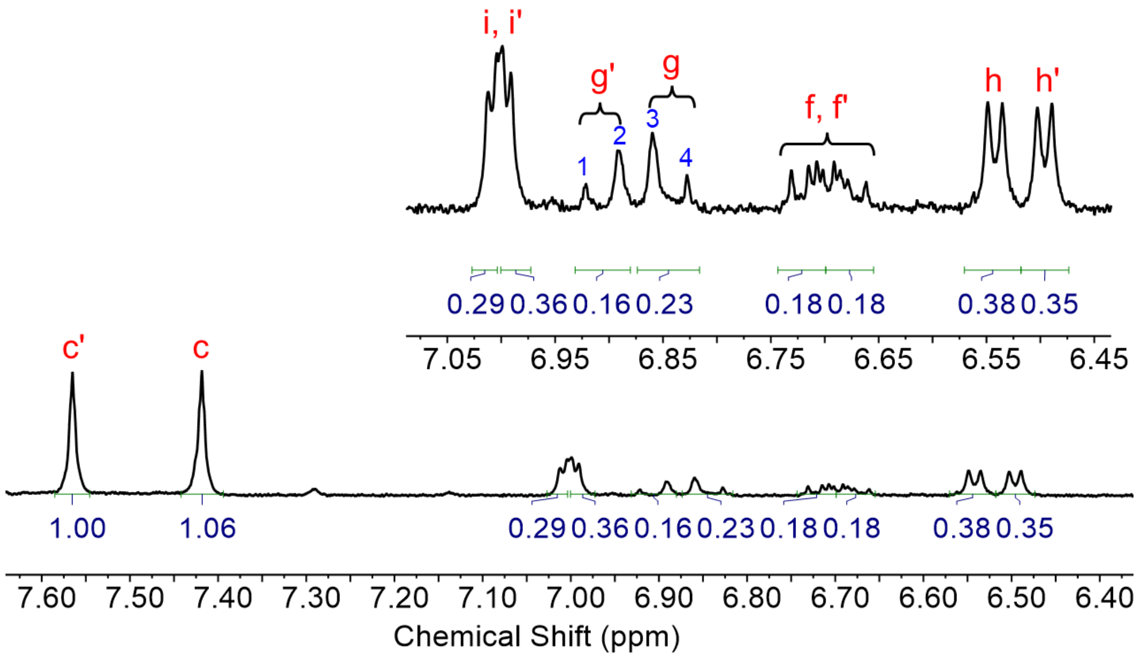

Figure S87. ^{1}H NMR signal intensity comparison of 4-trifluoromethylbenzyl vinylphosphonate and cyanostar. (CD_2Cl_2, 600 MHz, 298 K)

The assignment of peaks g and g’ was based on the *J*-coupling constant comparison of peak 1 and 2, 1 and 3. The *J*-coupling constant of peak 1 and 2 is 17.43 Hz, which is identical to the coupling constant of proton g caused by the splitting of proton f in the acid format. Similarly, the *J*-coupling constant of peak 3 and 4 is 17.78 Hz. Therefore, 1 and 2 are in one set, 3 and 4 are in another set, considering the integration ratio between two sets, they were assigned to be g and g’.

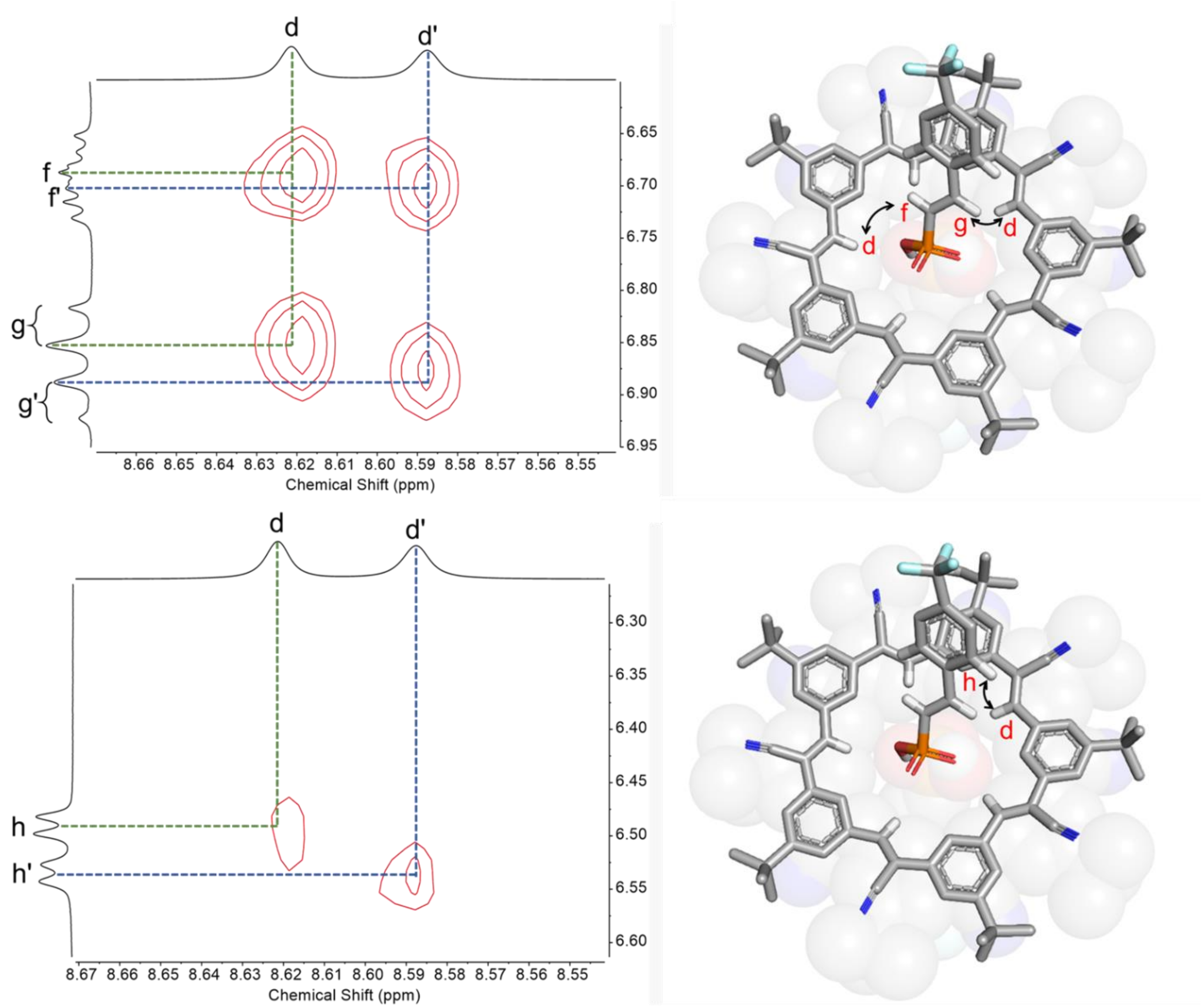

Figure S88. ROESY NMR spectra of 3.5 mM cyanostar solution with 0.5 equiv. 4-trifluoromethylbenzyl vinylphosphonate. (CD_2Cl_2, 500 MHz, 298 K) Dimer model was modified from crystal structure.

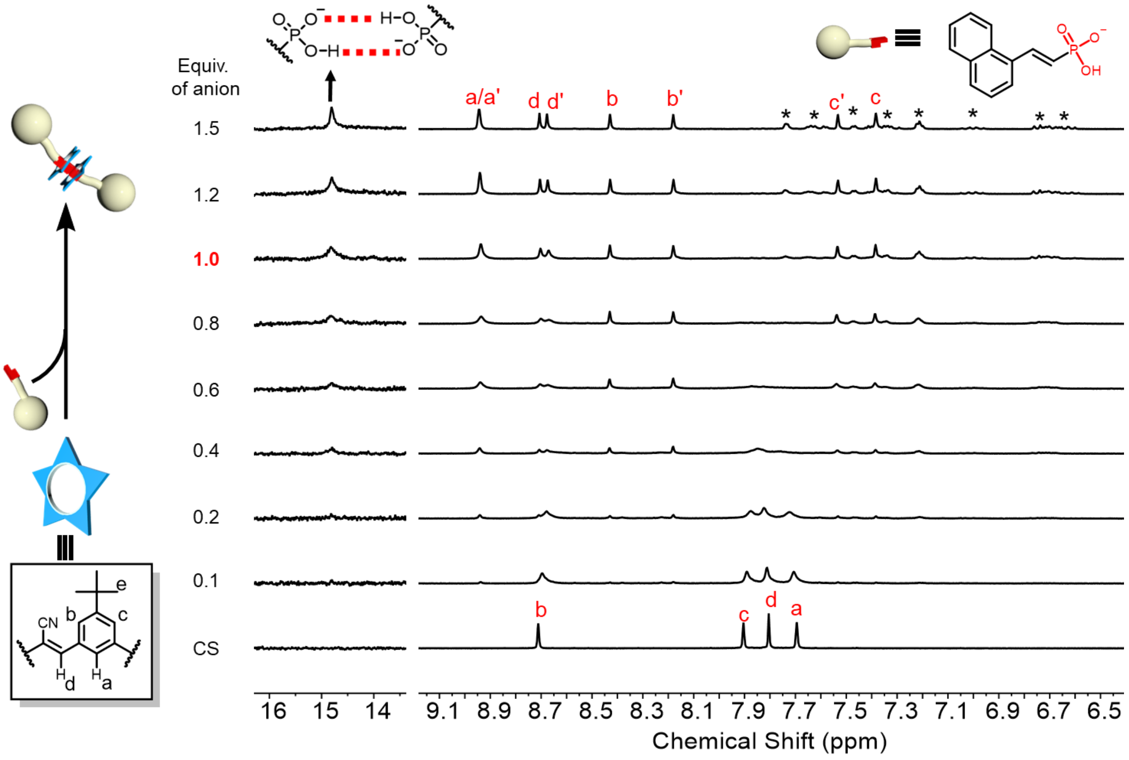

Figure S89. ^{1}H NMR titration of naphthyl vinylphosphonate into 1 mM cyanostar solution. (CD_2Cl_2, 600 MHz, 298 K) Peaks marked with asterisk are complexed anions.

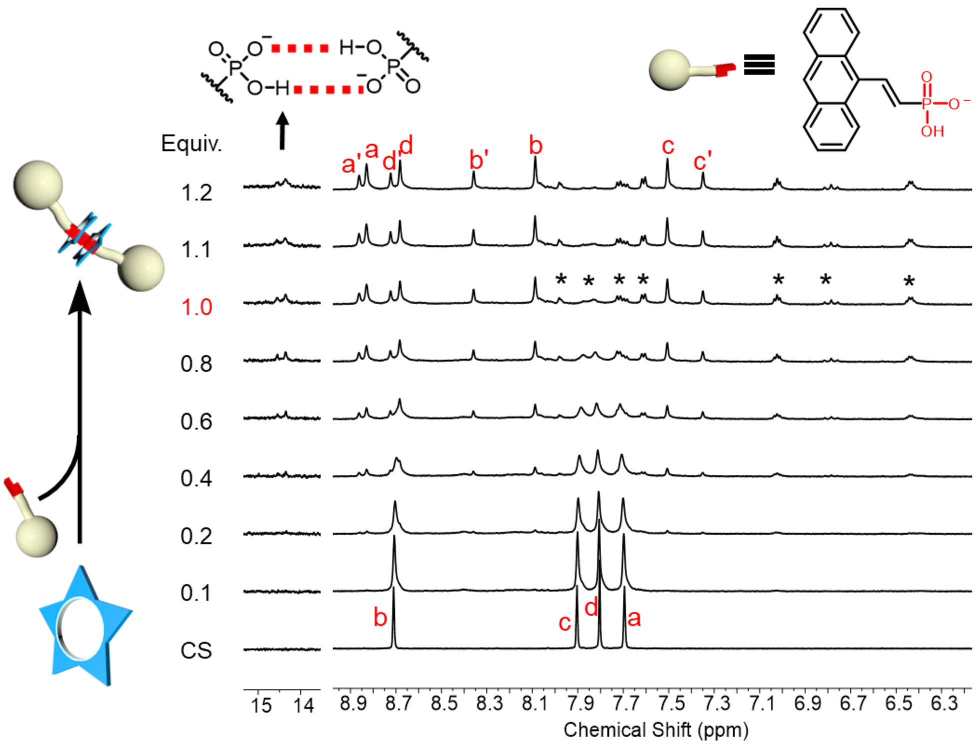

Figure S90. ^{1}H NMR titration of anthracene vinylphosphonate into 1 mM cyanostar solution. (CD_2Cl_2, 600 MHz, 298 K) Peaks marked with asterisk are complexed anions.

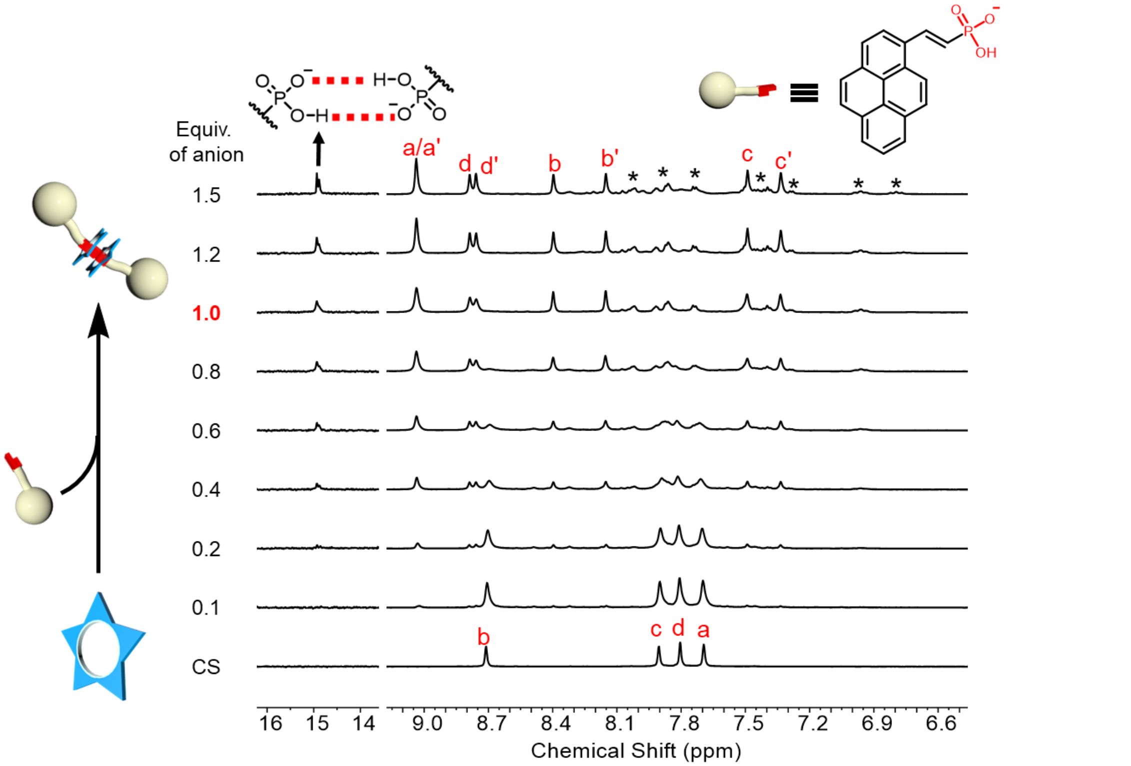

Figure S91. ^{1}H NMR titration of pyrene vinylphosphonate into 1 mM cyanostar solution. (CD_2Cl_2, 600 MHz, 298 K) Peaks marked with asterisk are complexed anions.

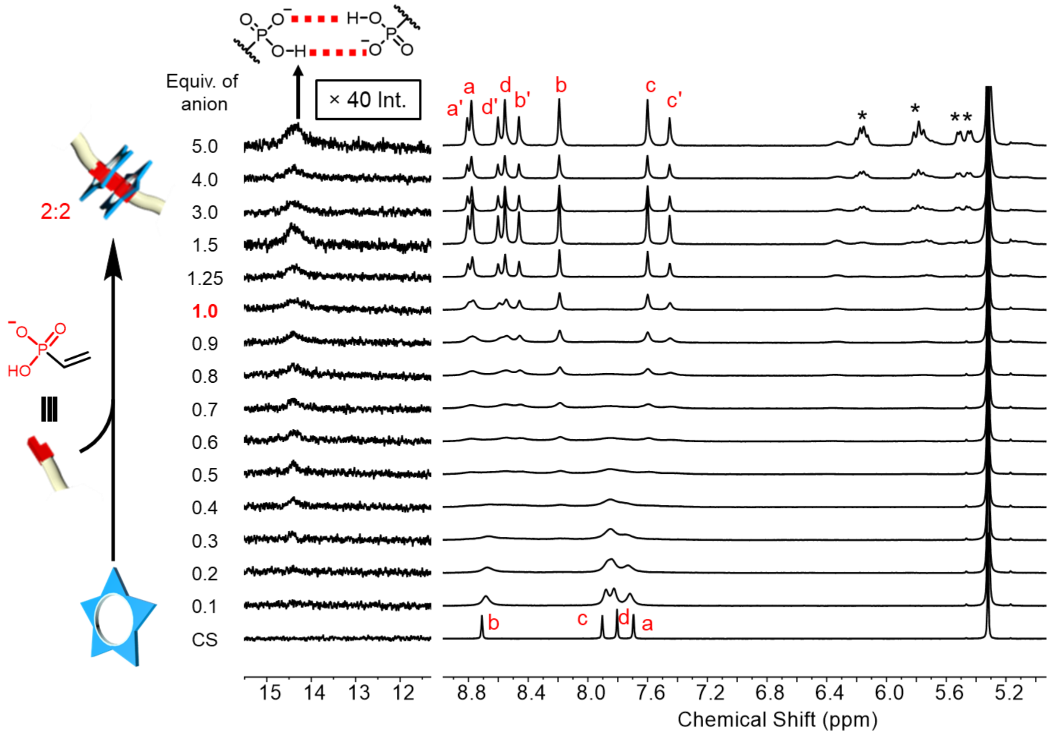

Figure S92. ^{1}H NMR titration of vinylphosphonate into 1 mM cyanostar solution. (CD_2Cl_2, 600 MHz, 298 K) Peaks marked with asterisk are excess anions.

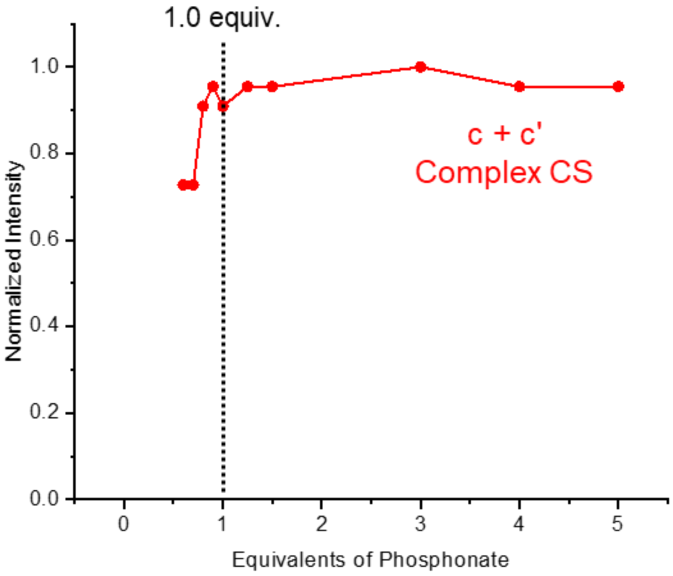

Figure S93. Proton intensity versus equivalents of complexed cyanostar in the titration of vinylphosphonate into 1 mM cyanostar solution. Data points start at 0.6 equivalents, as before this equivalent, free cyanostar and complexed cyanostar resonances are in intermediate exchange.

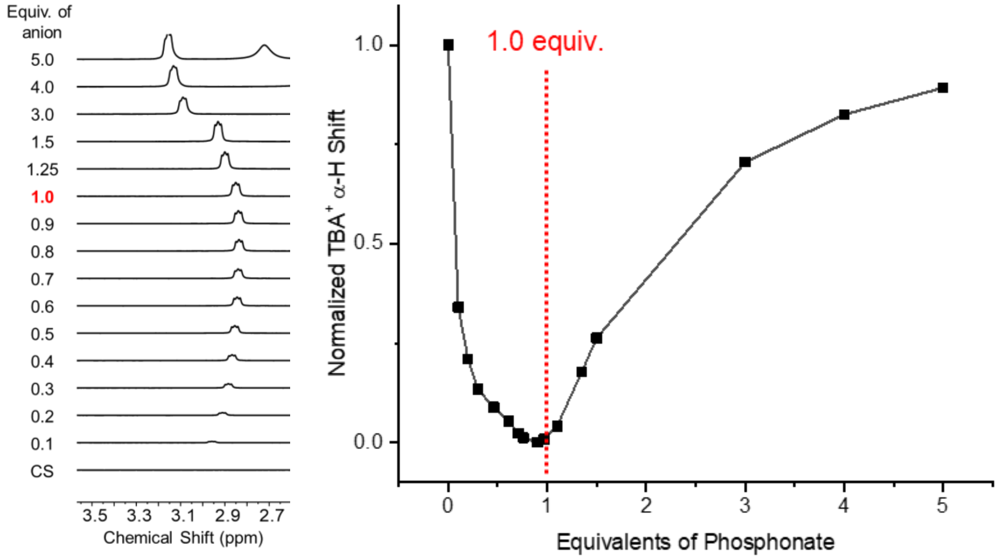

Figure S94. α-Proton shift of TBA^+ versus equivalents of anion in the titration of vinylphosphonate into 1 mM cyanostar solution.

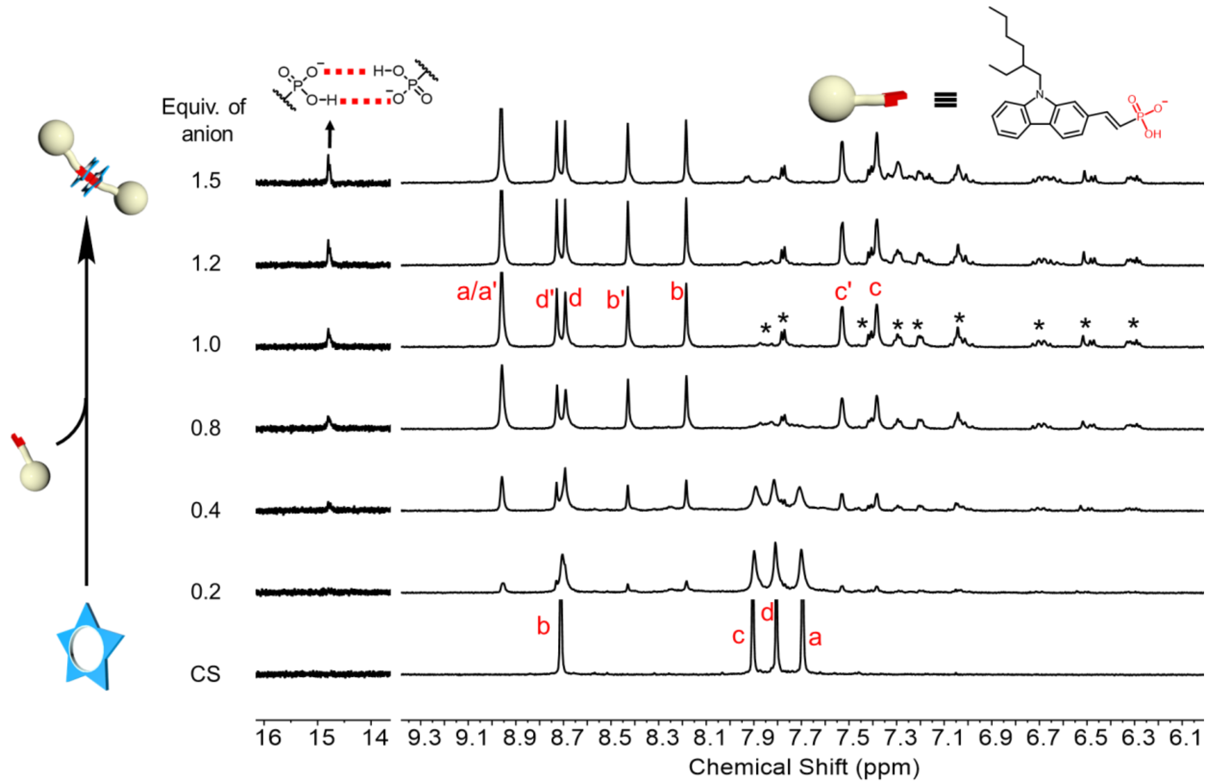

Figure S95. ^{1}H NMR titration of 2-ethylhexyl carbazole vinylphosphonate into 1 mM cyanostar solution. (CD_2Cl_2, 600 MHz, 298 K) Peaks marked with asterisk are complexed anions.

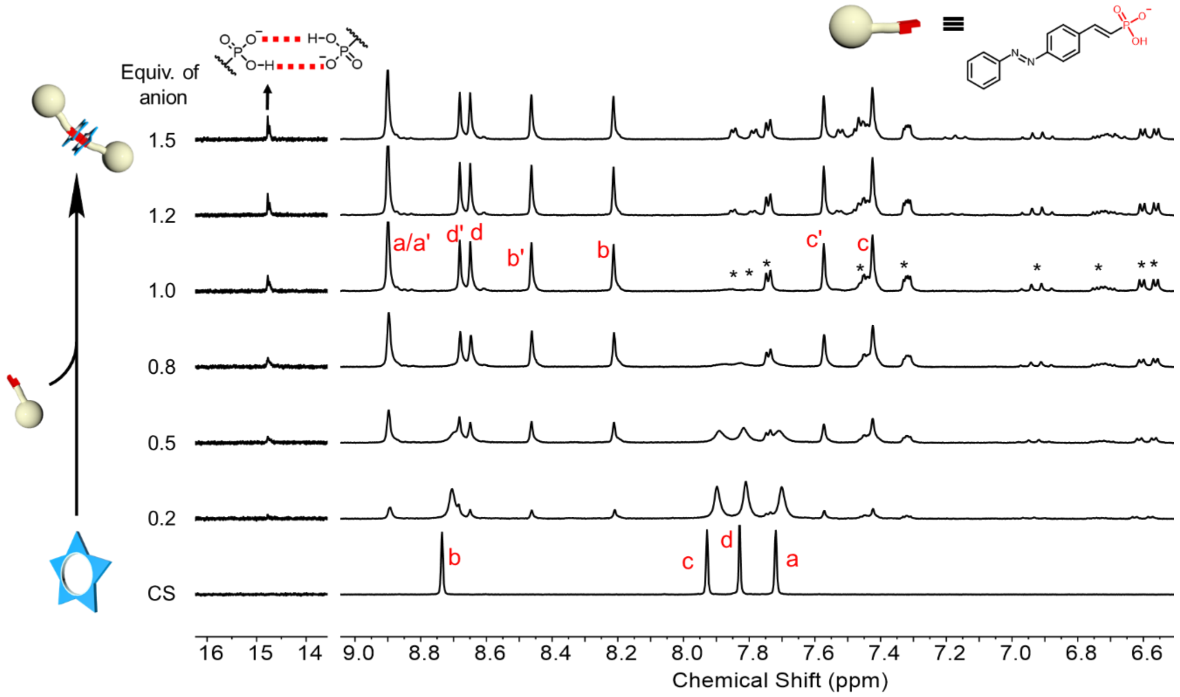

Figure S96. ^{1}H NMR titration of azobenzene vinylphosphonate into 1 mM cyanostar solution. (CD_2Cl_2, 600 MHz, 298 K) Peaks marked with asterisk are complexed anions.

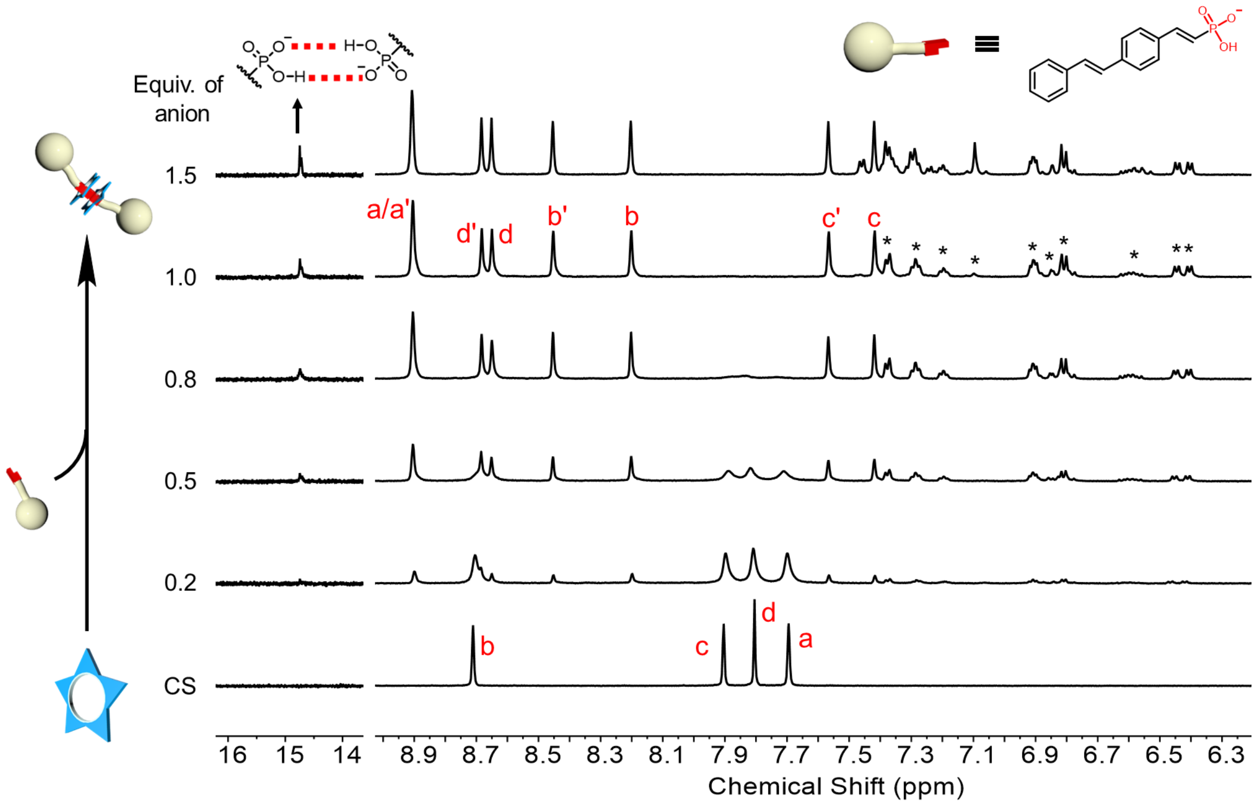

Figure S97. ^{1}H NMR titration of stilbene vinylphosphonate into 1 mM cyanostar solution. (CD_2Cl_2, 600 MHz, 298 K) Peaks marked with asterisk are complexed anions.

S5. HRMS of Versatile Cyanostar-anion Dimers

Samples for high resolution ESI mass analysis were directly infused into a ThermoFisher LTQ Orbitrap XL at a rate of 8 – 12 uL/min from THF/DCM. The HESI II source was kept at 50 ^oC with a spray voltage of 2.7 kV. Sheath and Aux gases were set to 20 and 5 (arbitrary units) but were varied as needed to maintain a stable spray. Ion transfer tube was held at 275 ^oC. Tube lens and capillary voltages were varied to ensure transmission of ions to the detector.

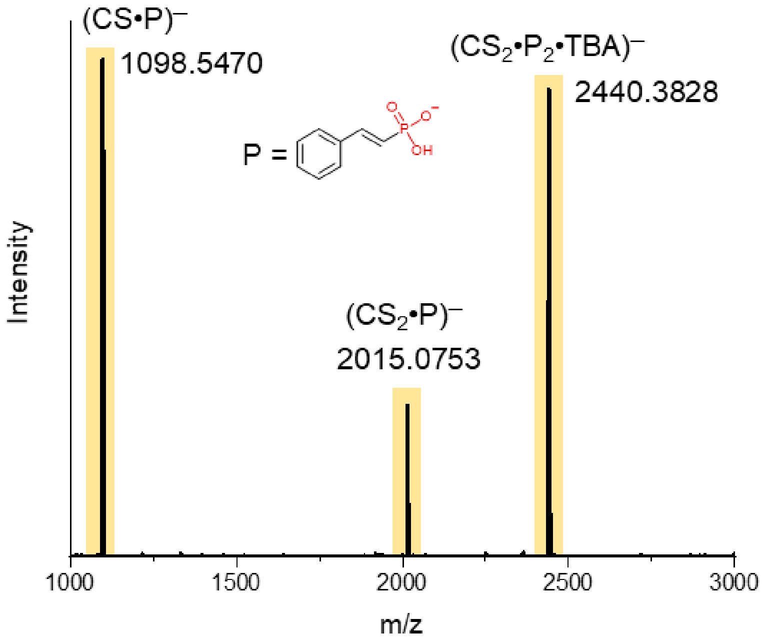

Figure S98. HRMS spectrum of 1 mM cyanostar dichloromethane solution and 1.0 equiv. of phenyl vinylphosphonate.

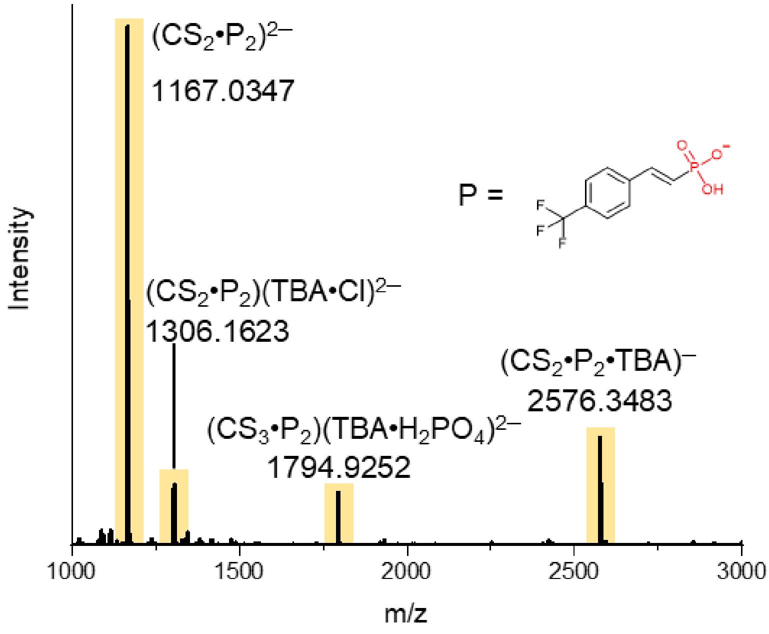

Figure S99. HRMS spectrum of 1 mM cyanostar dichloromethane solution and 1.0 equiv. of 4-trifluoromethylbenzyl vinylphosphonate.

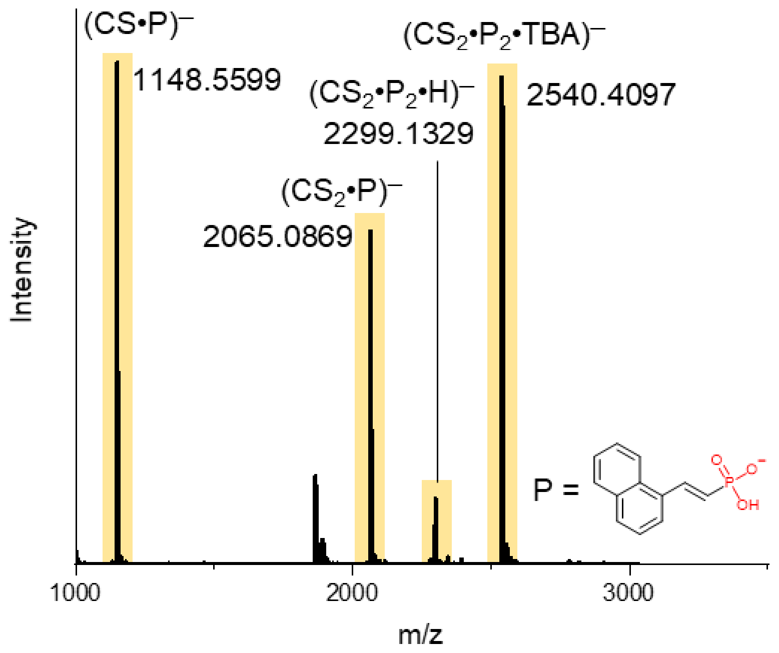

Figure S100. HRMS spectrum of 1 mM cyanostar dichloromethane solution and 1.0 equiv. of naphthyl vinylphosphonate.

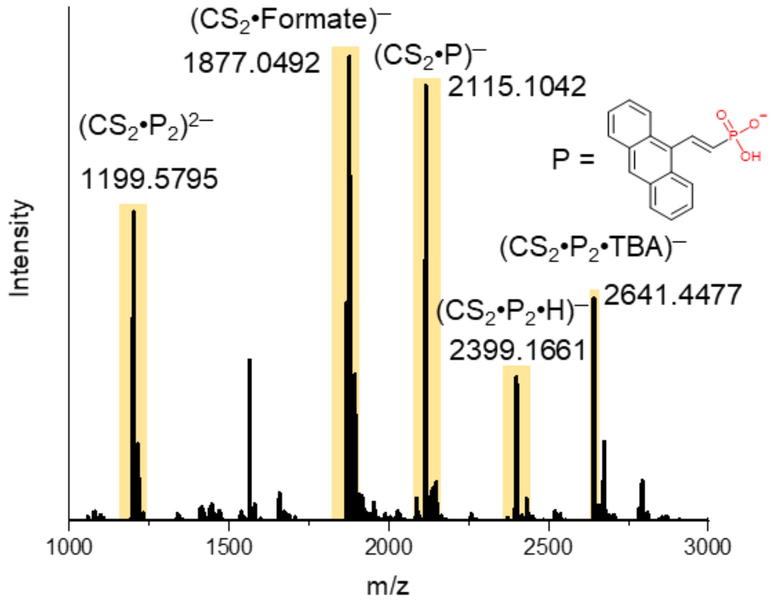

Figure S101. HRMS spectrum of 1 mM cyanostar dichloromethane solution and 1.0 equiv. of anthracene vinylphosphonate.

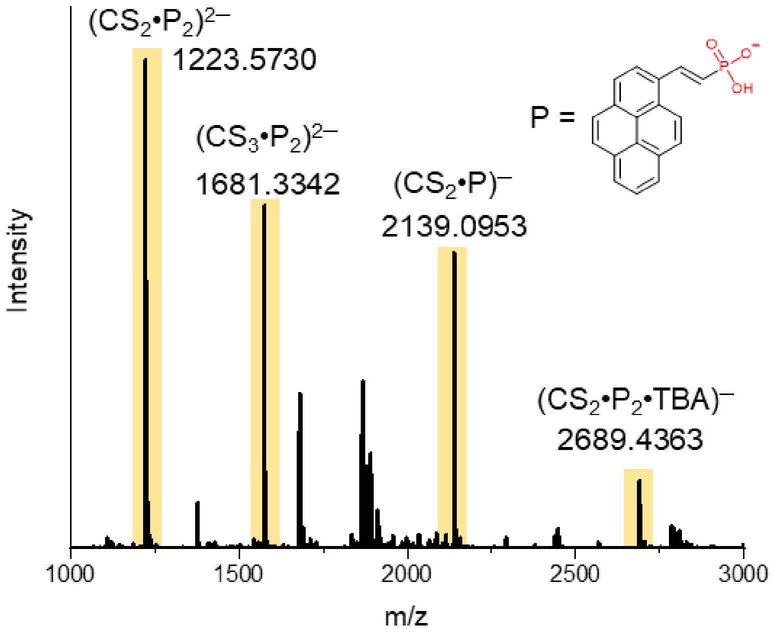

Figure S102. HRMS spectrum of 1 mM cyanostar dichloromethane solution and 1.0 equiv. of pyrene vinylphosphonate.

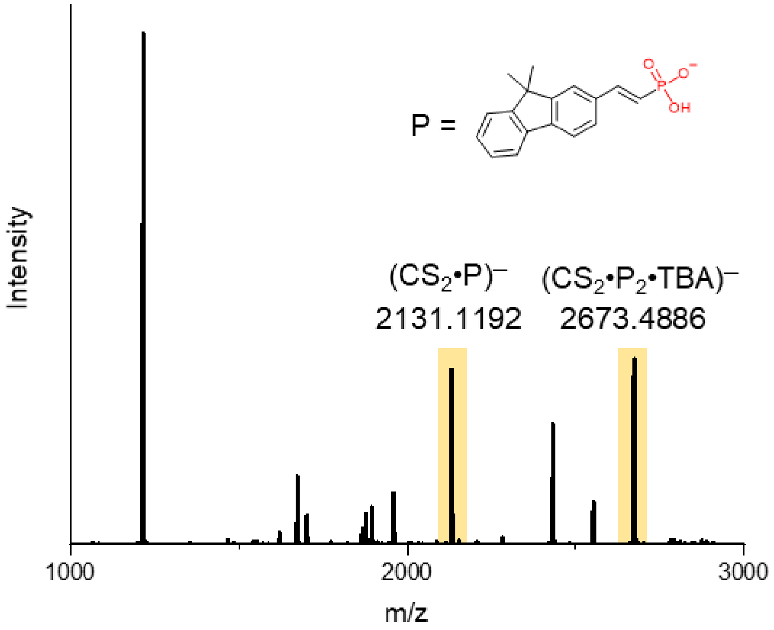

Figure S103. HRMS spectrum of 1 mM cyanostar dichloromethane solution and 1.0 equiv. of dimethyl fluorene vinylphosphonate.

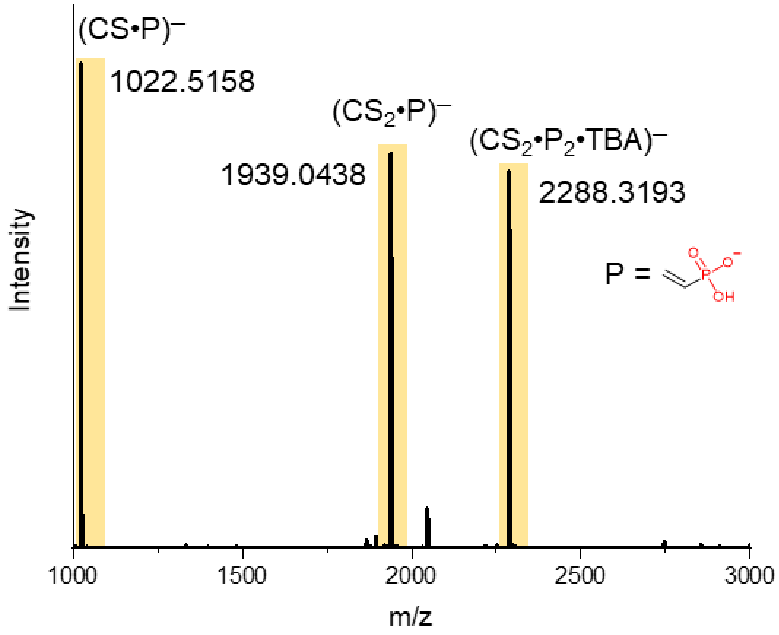

Figure S104. HRMS spectrum of 1 mM cyanostar dichloromethane solution and 1.0 equiv. of vinyl phosphonate.

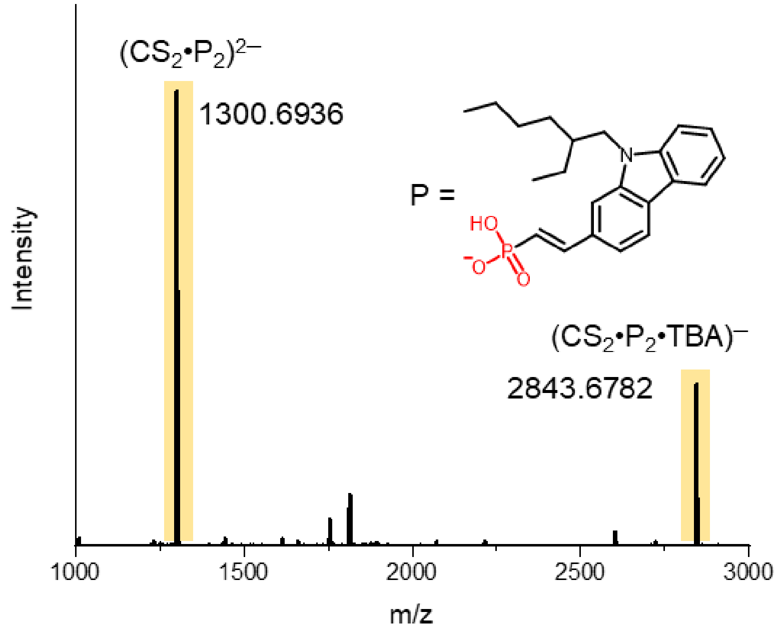

Figure S105. HRMS spectrum of 1 mM cyanostar dichloromethane solution and 1.0 equiv. of 2-ethylhexyl carbazole vinyl phosphonate.

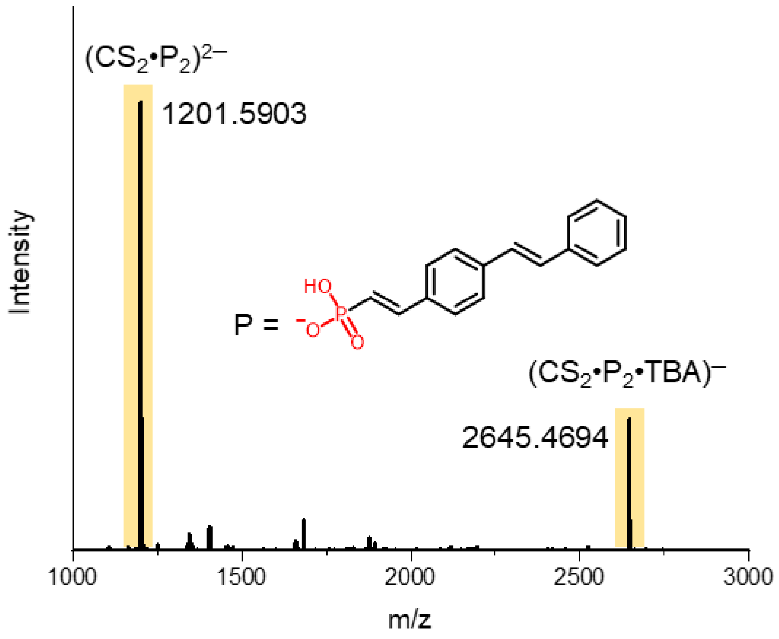

Figure S106. HRMS spectrum of 1 mM cyanostar dichloromethane solution and 1.0 equiv. of stilbene vinyl phosphonate.

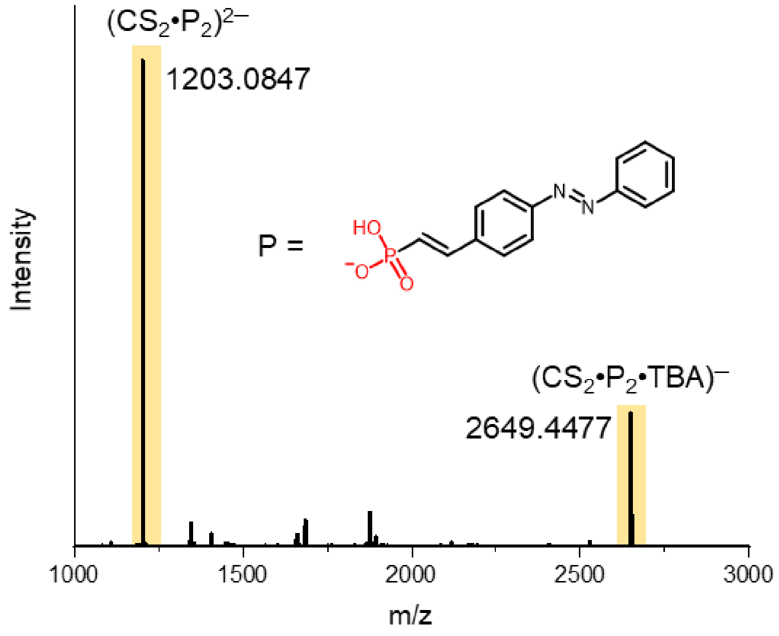

Figure S107. HRMS spectrum of 1 mM cyanostar dichloromethane solution and 1.0 equiv. of azobenzene vinyl phosphonate.

S6. Characterization of Supramolecular Polymer based on Di-vinyl Phosphonates

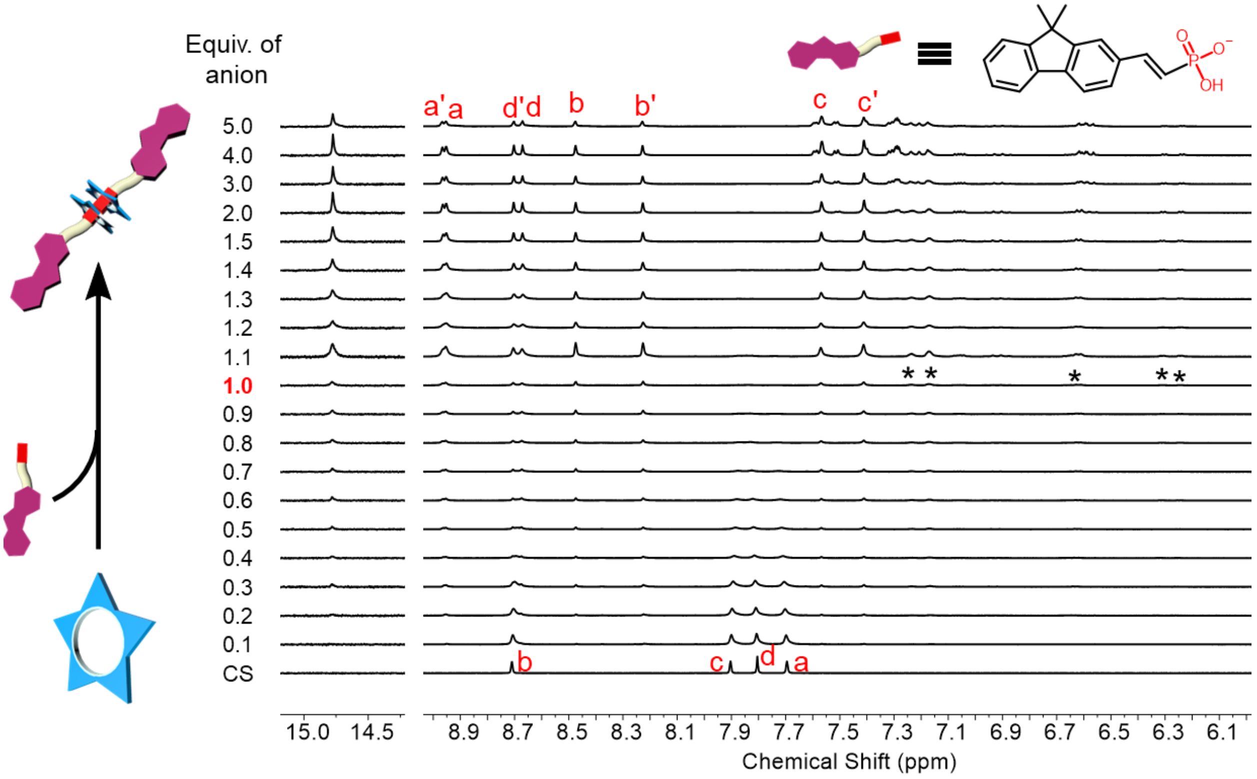

Figure S108. ^{1}H NMR titration of **Me2fluorene-VP** into 1 mM cyanostar solution. (CD_2Cl_2, 600 MHz, 298 K) Peaks marked with asterisk are complexed anions.

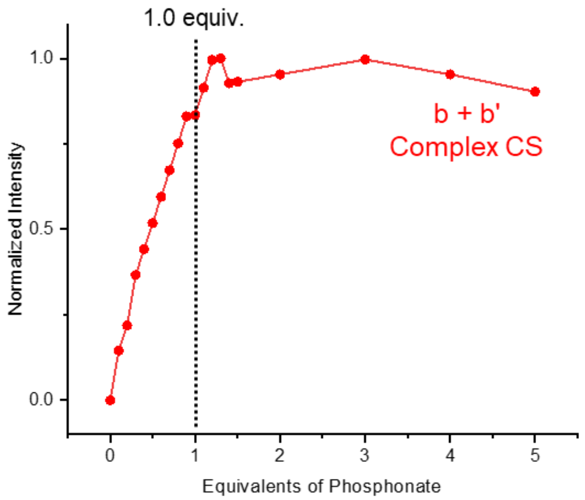

Figure S109. Proton intensity versus equivalents of complexed cyanostar in the titration of dimethyl fluorene vinylphosphonate into 1 mM cyanostar solution.

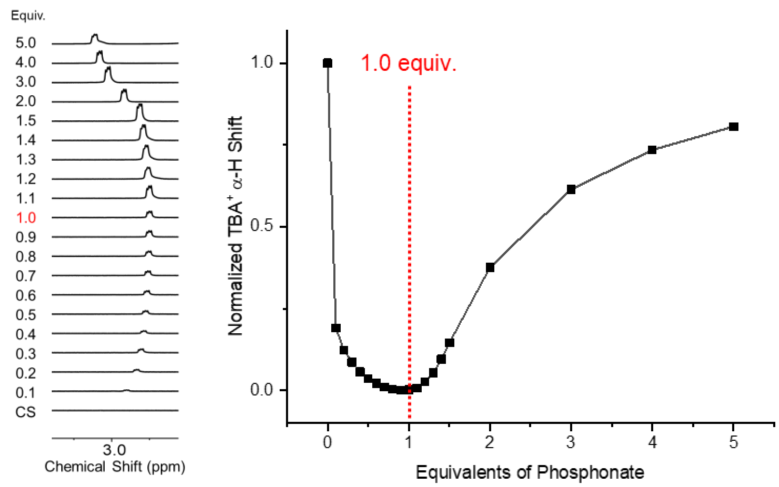

Figure S110. α-Proton shift of TBA^+ versus equivalents of anion in the titration of dimethyl fluorene vinylphosphonate into 1 mM cyanostar solution.

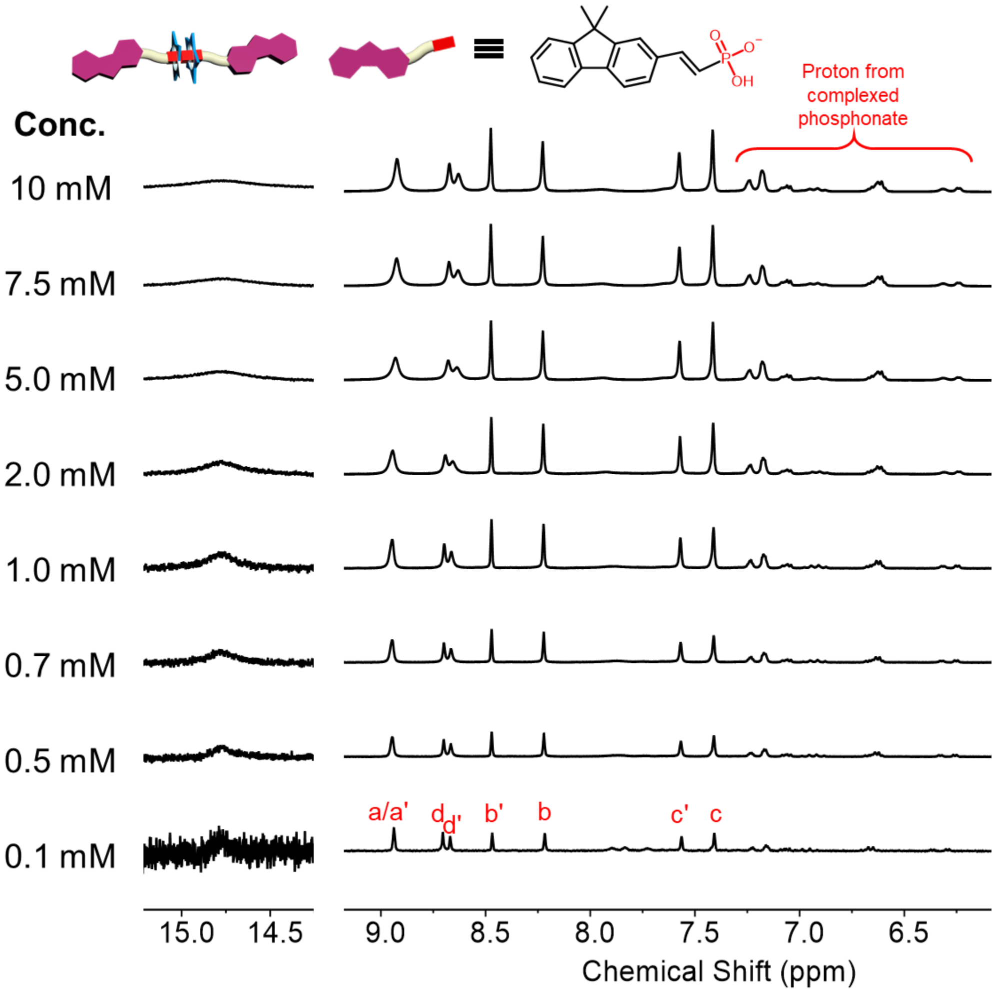

Figure S111. Variable concentration ^1H NMR of 2:2 cyanostar:**Me$_2$fluorene-VP** complex solution. (CD_2Cl_2, 600 MHz, 298 K)

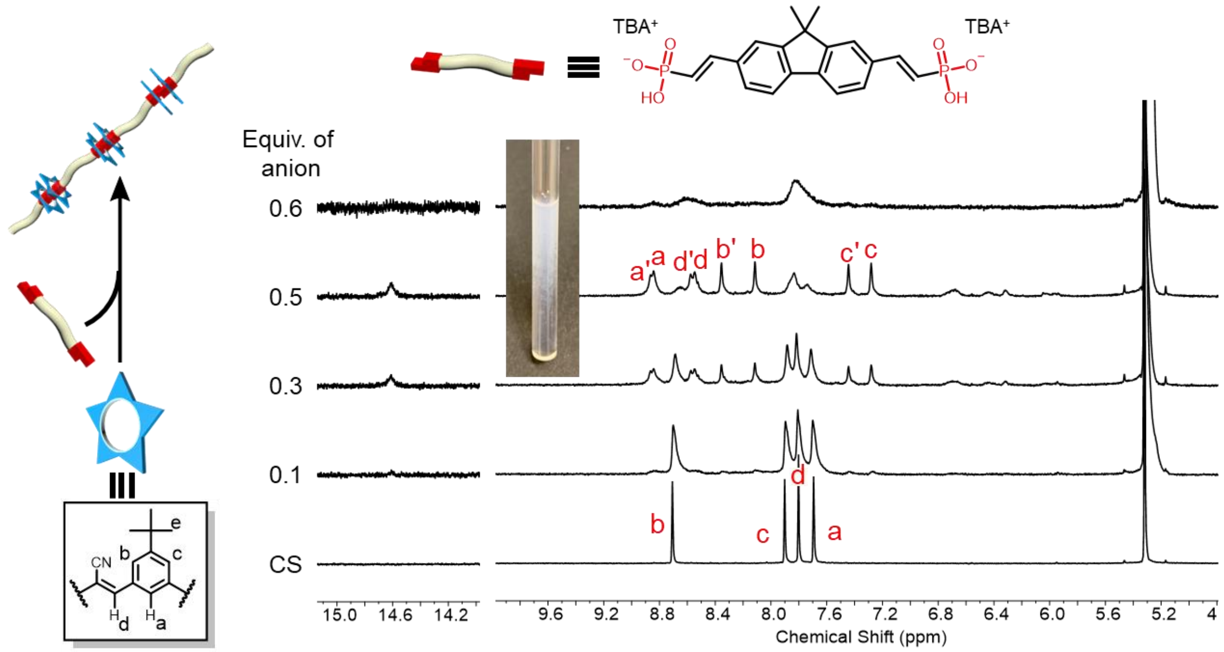

Figure S112. ^{1}H NMR titration of methyl ditopic monomer into 1 mM cyanostar solution. (CD_2Cl_2, 600 MHz, 298 K) The inserted image is the sample after addition of 0.5 equivalent anion.

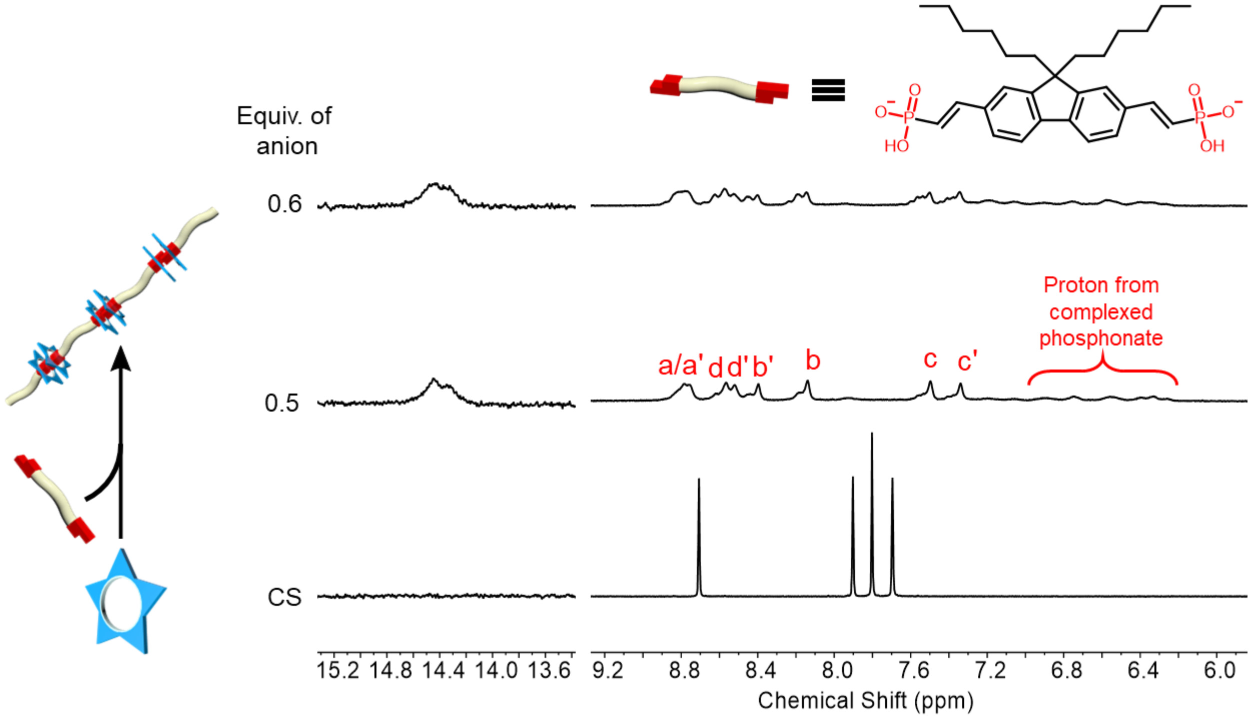

Figure S113. ^{1}H NMR titration of hexyl ditopic monomer into 2 mM cyanostar solution. (CD_2Cl_2, 600 MHz, 298 K)

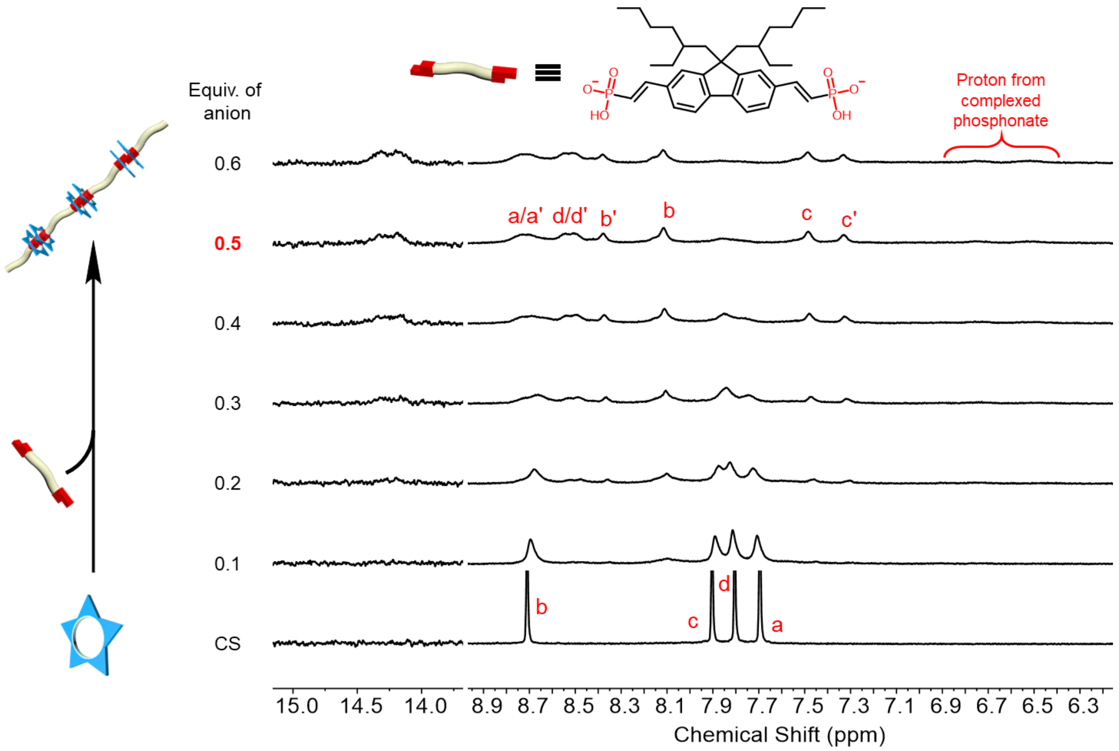

Figure S114. ^{1}H NMR titration of 2-ethylhexyl ditopic monomer into 1 mM cyanostar solution. (CD_2Cl_2, 600 MHz, 298 K)

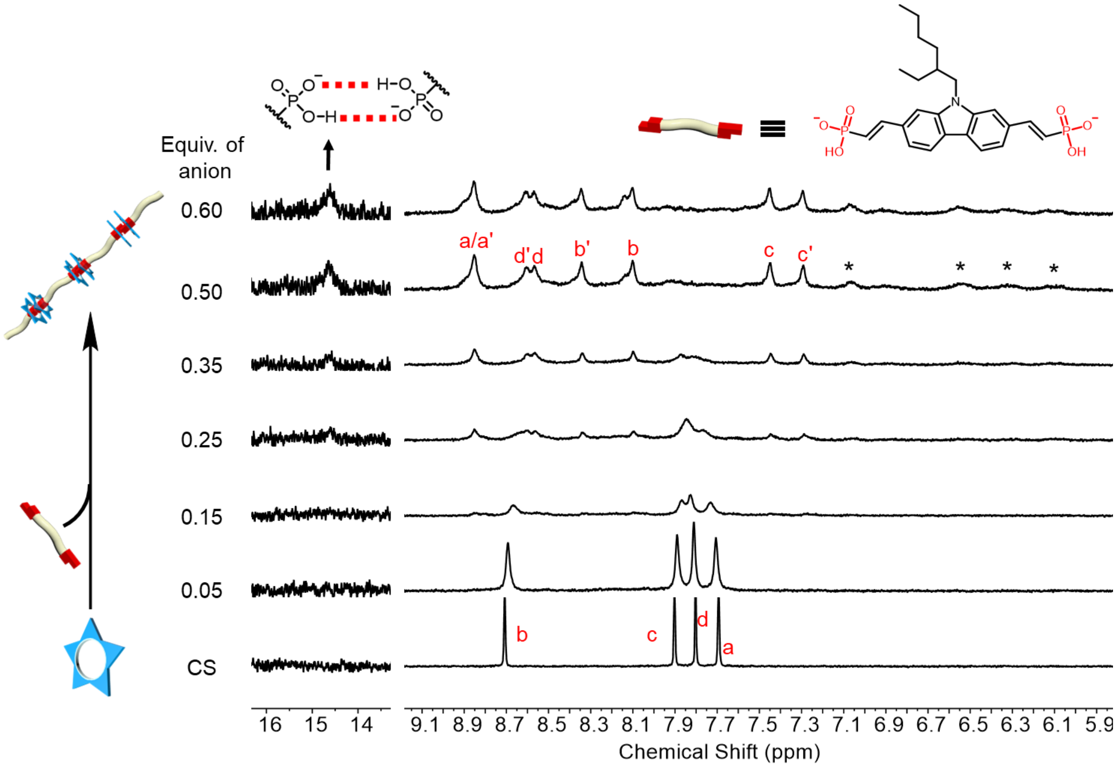

Figure S115. ^{1}H NMR titration of 2-ethylhexyl carbazole bis(vinylphosphonate) into 1 mM cyanostar solution. (CD_2Cl_2, 600 MHz, 298 K) Peaks marked with asterisk are complexed anions.

Comments on solubility: The fluorene-based monomers with dimethyl and dihexyl side chains forms precipitation immediately at 1 and 2 mM cyanostar dichloromethane solution when 0.5 equivalents of ditopic monomer was added (equivalence point). The carbazole-based monomer with 2-ethylhexyl chain forms precipitation at 1 mM cyanostar dichloromethane solution in one day after the addition of 0.5 equivalents of ditopic monomer.

Table 1. Diffusion data of supramolecular polymer consist of 2-ethylhexthyl ditopic monomer and cyanostar.

Cyanostar Concentration (mM)	Proton Signal (ppm)	Diffusion Coefficient (10^{-11} m^2/s)	Average (10^{-11} m^2/s)
2	8.38	9.18884	9.6 ± 0.3
	8.12	9.65264	
	7.49	9.94793	
	7.34	9.47507	
10	8.38	6.17641	6.2 ± 0.1
	8.12	6.25744	
	7.49	6.33665	
	7.34	6.20028	

S7. X-ray Diffraction Data Analysis

S7.1 Me_2fluorene-VP-CS (label 22005)

Data collection

A colorless crystal (approximate dimensions 0.221 × 0.216 × 0.109 mm^3) was placed onto the tip of a MiTeGen loop and mounted on a Bruker Venture D8 diffractometer equipped with a PhotonIII detector at 153(2) K.

The data collection was carried out using Mo Kα radiation (graphite monochromator) with a frame time of 59 and 2 seconds and a detector distance of 5.00 cm. A collection strategy was calculated and complete data to a resolution of 0.80 Å with a redundancy of 10.7 were collected. Thirteen major sections of frames were collected with 1° ω and ϕ scans. A total of 2011 frames were collected. The total exposure time was 19.82 hours. The frames were integrated with the Bruker SAINT software package[4] using a narrow-frame algorithm. The integration of the data using a triclinic unit cell yielded a total of 185443 reflections to a maximum θ angle of 25.09° (0.84 Å resolution), of which 17340 were independent (average redundancy 10.695, completeness = 99.7%, R_{int} = 6.09%, R_{sig} = 3.24%) and 11473 (66.16%) were greater than $2\sigma(F^2)$. The final cell constants of a = 15.9708(14) Å, b = 16.8532(14) Å, c = 19.6348(16) Å, α = 98.989(2)°, β = 107.266(2)°, γ = 97.797(2)°, volume = 4891.0(7) $Å^3$, are based upon the refinement of the XYZ-centroids of 9946 reflections above 20 σ(I) with 4.619° < 2θ < 45.48°. Data were corrected for

absorption effects using the Multi-Scan method (SADABS[5]). The ratio of minimum to maximum apparent transmission was 0.923. The calculated minimum and maximum transmission coefficients (based on crystal size) are 0.9830 and 0.9910.

Structure solution and refinement

The space group P-1 was determined based on intensity statistics and the lack of systematic absences. The structure was solved and refined using the SHELX suite of programs.[6, 7] An intrinsic-methods solution was calculated, which provided most non-hydrogen atoms from the E-map. Full-matrix least squares / difference Fourier cycles were performed, which located the remaining non-hydrogen atoms. All non-hydrogen atoms were refined with anisotropic displacement parameters. The hydrogen atoms were placed in ideal positions and refined as riding atoms with relative isotropic displacement parameters. Disorder was refined for CS, tba, and the anion. Sets of restraints and constraints were applied. The final anisotropic full-matrix least-squares refinement on F^2 with 1351 variables converged at R1 = 9.24%, for the observed data and wR2 = 30.30% for all data. The goodness-of-fit was 1.030. The largest peak in the final difference electron density synthesis was 1.753 $e^-/Å^3$ and the largest hole was -0.378 $e^-/Å^3$ with an RMS deviation of 0.064 $e^-/Å^3$. On the basis of the final model, the calculated density was 1.078 g/cm^3 and F(000), 1722 e^-.

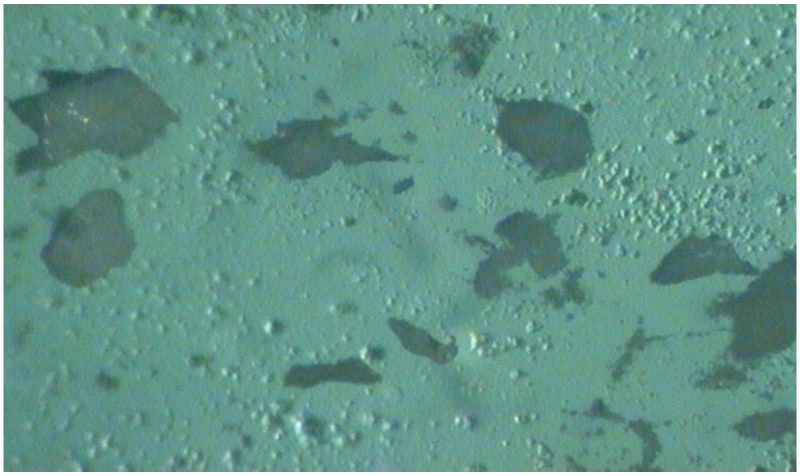

Figure S116. Image of bulk material.

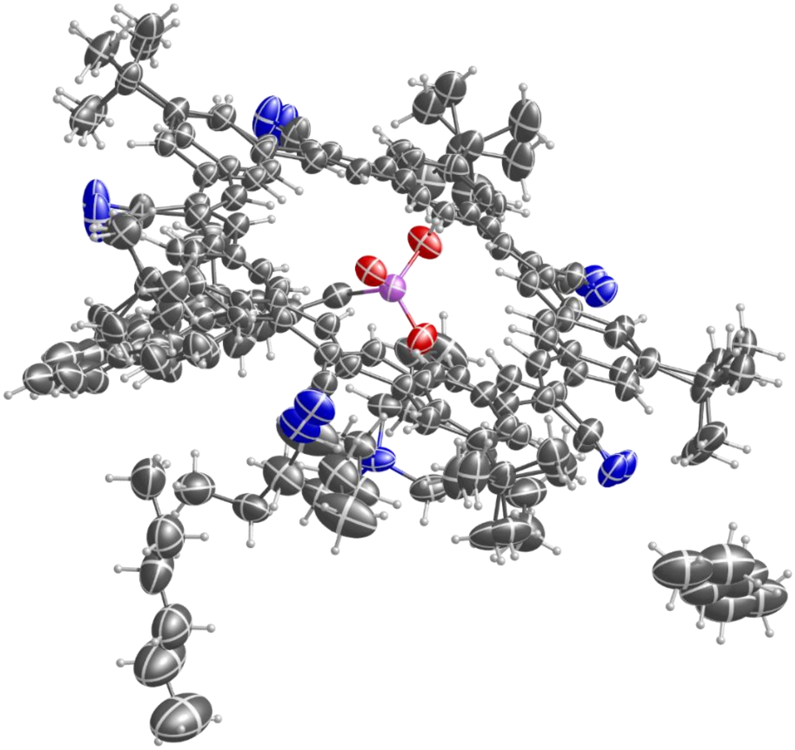

Figure S117. Formula unit.

Table 2. Crystal data and structure refinement.

CCDC deposition number	2263985	
Empirical formula	C107 H138 N6 O3 P	
Formula weight	1587.20	
Crystal color, shape, size	colourless block, 0.221 × 0.216 × 0.109 mm^3	
Temperature	150(2) K	
Wavelength	0.71073 Å	
Crystal system, space group	Triclinic, P-1	
Unit cell dimensions	a = 15.9708(14) Å	α = 98.989(2)°.
	b = 16.8532(14) Å	β = 107.266(2)°.
	c = 19.6348(16) Å	γ = 97.797(2)°.
Volume	4891.0(7) $Å^3$	
Z	2	
Density (calculated)	1.078 Mg/m^3	
Absorption coefficient	0.079 mm^{-1}	
F(000)	1722	
Data collection		
Diffractometer	Venture D8, Bruker	
Source	IμS 3.0, Incoatec	
Detector	Photon III	
Theta range for data collection	1.891 to 25.088°.	
Index ranges	-19<=h<=19, -20<=k<=20, -23<=l<=23	
Reflections collected	185443	
Independent reflections	17340 [Rint = 0.0609]	
Observed Reflections	11473	
Completeness to theta = 25.088°	99.7 %	
Solution and Refinement		
Absorption correction	Semi-empirical from equivalents	
Max. and min. transmission	0.7452 and 0.6881	
Solution	Intrinsic methods	
Refinement method	Full-matrix least-squares on F^2	
Weighting scheme	$w = [\sigma^2 Fo^2 + AP^2 + BP]^{-1}$, with	
	$P = (Fo^2 + 2 Fc^2)/3$, A = 0.1656, B = 4.6390	
Data / restraints / parameters	17340 / 3352 / 1351	
Goodness-of-fit on F^2	1.030	
Final R indices [I>2σ(I)]	R1 = 0.0924, wR2 = 0.2653	
R indices (all data)	R1 = 0.1291, wR2 = 0.3030	
Largest diff. peak and hole	1.753 and -0.378 e.$Å^{-3}$	

Goodness-of-fit = $[\Sigma[w(F_o^2 - F_c^2)^2]/N_{observns} - N_{params})]^{1/2}$, all data.

R1 = $\Sigma(|F_o| - |F_c|) / \Sigma |F_o|$. $wR2 = [\Sigma[w(F_o^2 - F_c^2)^2] / \Sigma [w(F_o^2)^2]]^{1/2}$.

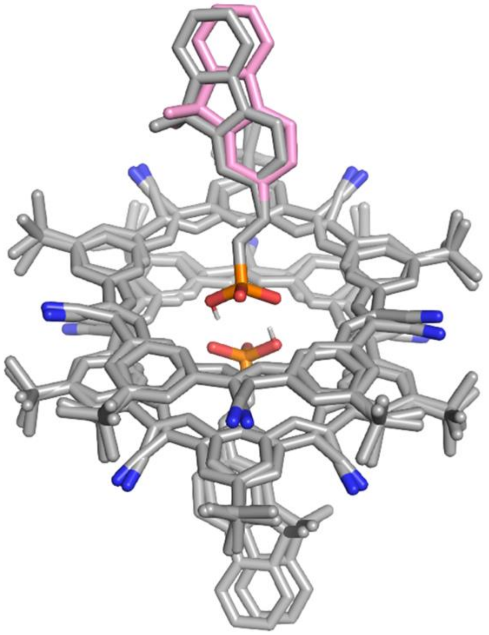

Figure S118. Tilt angle of fluorene moiety shown in the **Me$_2$fluorene-VP** cyanostar complex. One of the fluorene moiety shows pink and another shows gray. Counter-cations and solvent molecules omitted for clarity.

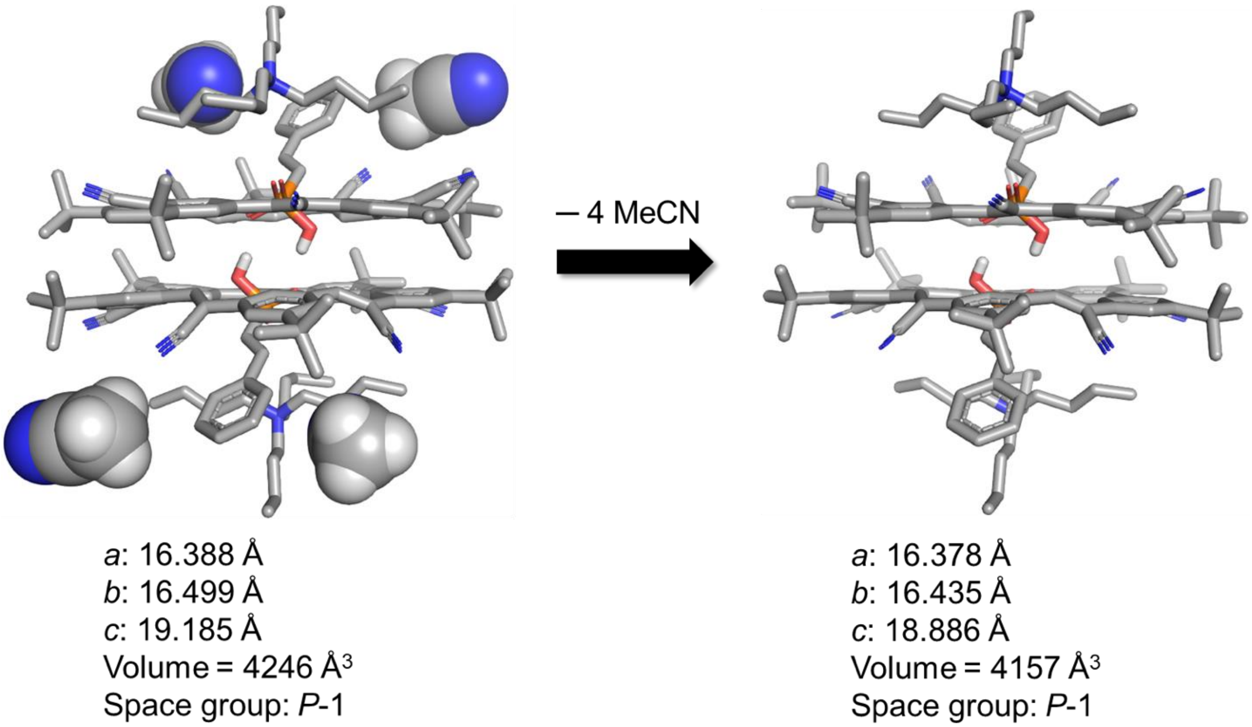

Figure S119. Crystal-to-crystal transition of the **Phenyl-VP** cyanostar complex. Disorder of cyanostar and counter-cation were omitted for clarity.

We obtained the crystal structure for **Phenyl-VP** dimer in both its solvated and fully desolvated state (Figure S119, CCDC 2263986 and 2263987). The single crystal was obtained by slowly evaporating its mixed solution in dichloromethane and acetonitrile (*v*/*v*, 3:2). Desolvation of cyanostar-based crystals typically deteriorates the quality of the diffraction data. In the present case, however, we observed a crystal-to-crystal transition upon loss of four molecules of acetonitrile per unit cell. The packing shows modest changes in relative location of the two cyanostars, the two anions and two cations. The *P*_1 space group does not change, and the unit cell parameters only slightly changed. The unit cell volume contracted 88 Å^3, which is less than the loss of four acetonitrile molecules (53 Å^3/per molecule). In general, the structures are loosely packed such that the volume change is not exactly the volume of the solvent molecules. Consistently, the distance between the two cyanostar planes is 3.6 Å for the solvated structure and expands a little to 3.7 Å for the desolvated one. The distance between donor and acceptor oxygen atoms also extend from 2.49 Å to 2.50 Å for the desolvated structure. Both of them are classified as very strong hydrogen bond[8], and in the range that is typical of hydrogen bonds between anionic dimers.[9, 10] We still observe the hydrogen bonding between two a-protons of TBA^+ and the phosphonate oxygen atom. In the solvated structure, we observe the nitrogen atom from acetonitrile interacts with two b-protons from TBA^+ (2.7 Å) and the hydrogen atom from acetonitrile interacts with the nitrogen atom from cyano-group on the macrocycle (2.7 Å).

S7.2 PhVP-CS (label 22149)

Data collection

A colorless crystal (approximate dimensions 0.319 × 0.160 × 0.154 mm^3) was placed onto the tip of a MiTeGen loop and mounted on a Bruker Venture D8 diffractometer equipped with a PhotonIII detector at 153(2) K.

The data collection was carried out using Mo Kα radiation (graphite monochromator) with a frame time of 90, 60, and 2 seconds and a detector distance of 5.00 cm. A collection strategy was calculated and complete data to a resolution of 0.77 Å with a redundancy of 10.1 were collected. Ten major sections of frames were collected with 1° ω and ϕ scans. A total of 1536 frames were collected. The total exposure time was 18.47 hours. The frames were integrated with the Bruker SAINT software package[4] using a narrow-frame algorithm. The integration of the data using a triclinic unit cell yielded a total of 192781 reflections to a maximum θ angle of 27.50° (0.77 Å resolution), of which 19054 were independent (average redundancy 10.118, completeness = 99.8%, R_{int} = 15.24%, R_{sig} = 9.21%) and 9623 (50.50%) were greater than $2\sigma(F^2)$. The final cell constants of a = 16.3782(4) Å, b = 16.4347(6) Å, c = 18.8864(6) Å, α = 111.4570(10)°, β = 112.340(2)°, γ = 97.4620(10)°, volume = 4157.2(2) Å^3, are based upon the refinement of the XYZ-centroids of 9695 reflections above 20 σ(I) with 4.622° < 2θ < 53.25°. Data were corrected for absorption effects using the Multi-Scan method (SADABS[5]). The ratio of minimum to maximum apparent transmission was 0.953. The calculated minimum and maximum transmission coefficients (based on crystal size) are 0.9740 and 0.9870.

Structure solution and refinement

The space group P-1 was determined based on intensity statistics and the lack of systematic absences. The structure was solved and refined using the SHELX suite of programs.[6, 7] An intrinsic-methods solution was calculated, which provided most non-hydrogen atoms from the E-

map. Full-matrix least squares / difference Fourier cycles were performed, which located the remaining non-hydrogen atoms. All non-hydrogen atoms were refined with anisotropic displacement parameters. The hydrogen atoms were placed in ideal positions and refined as riding atoms with relative isotropic displacement parameters. All parts of the structure are disordered and were refined with restraints and constraints. The CS disorder ratio is 52:48, the guest anion disorder ration is 73:27. The final anisotropic full-matrix least-squares refinement on F^2 with1038 variables converged at R1 = 8.00%, for the observed data and wR2 = 24.71% for all data. The goodness-of-fit was 1.086. The largest peak in the final difference electron density synthesis was 0.530 $e^-/Å^3$ and the largest hole was -0.444 $e^-/Å^3$ with an RMS deviation of 0.043 $e^-/Å^3$. On the basis of the final model, the calculated density was 1.072 g/cm^3 and F(000), 1448 e^-.

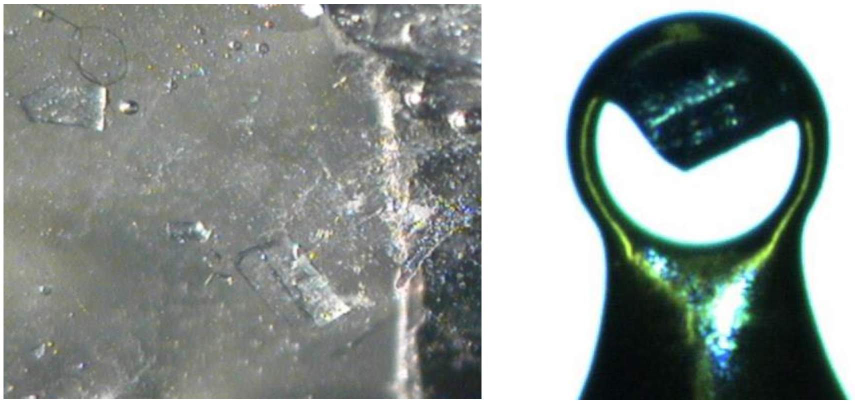

Figure S120. Image of bulk material and mounted crystal.

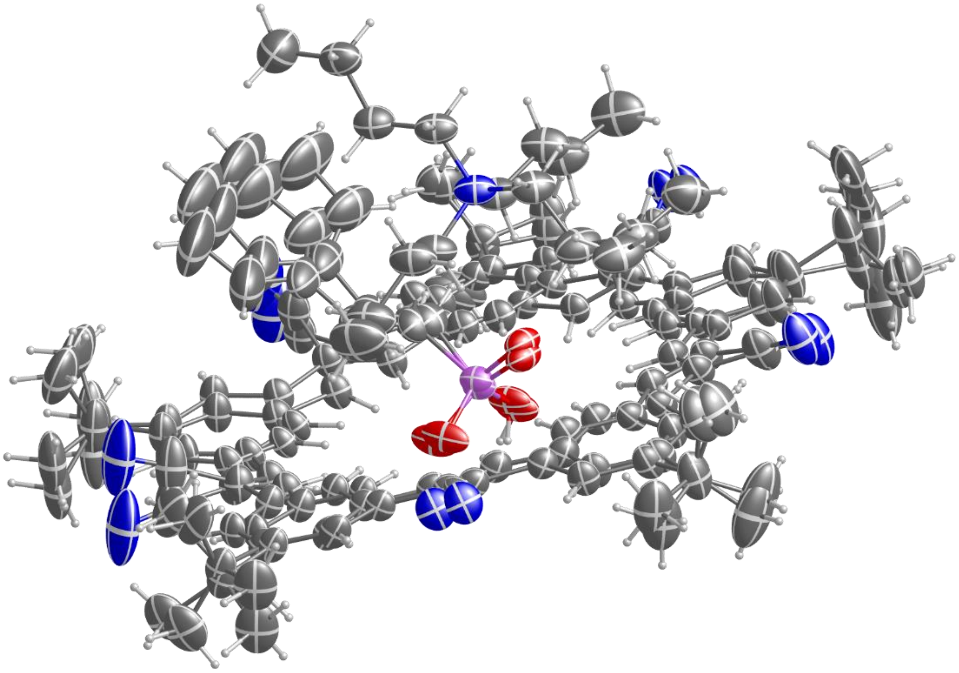

Figure S121. Formula unit.

Table 3. Crystal data and structure refinement.

CCDC deposition number	2263986	
Empirical formula	C89 H109 N6 O3 P	
Formula weight	1341.79	
Crystal color, shape, size	colorless block, 0.319 × 0.160 × 0.154 mm^3	
Temperature	153(2) K	
Wavelength	0.71073 Å	
Crystal system, space group	Triclinic, P-1	
Unit cell dimensions	a = 16.3782(4) Å	α = 111.4570(10)°.
	b = 16.4347(6) Å	β = 112.340(2)°.
	c = 18.8864(6) Å	γ = 97.4620(10)°.
Volume	4157.2(2) $Å^3$	
Z	2	
Density (calculated)	1.072 Mg/m^3	
Absorption coefficient	0.083 mm^{-1}	
F(000)	1448	
Data collection		
Diffractometer	Venture D8, Bruker	
Source	Iμ3.0, Incoatec	
Detector	Photon III	
Theta range for data collection	2.093 to 27.496°.	
Index ranges	-21<=h<=21, -21<=k<=21, -24<=l<=24	
Reflections collected	192781	
Independent reflections	19054 [Rint = 0.1524]	
Observed Reflections	9623	
Completeness to theta = 25.242°	99.9 %	
Solution and Refinement		
Absorption correction	Semi-empirical from equivalents	
Max. and min. transmission	0.7456 and 0.7107	
Solution	Intrinsic methods	
Refinement method	Full-matrix least-squares on F^2	
Weighting scheme	$w = [\sigma^2 Fo^2 + AP^2]^{-1}$, with $P = (Fo^2 + 2 Fc^2)/3$, A = 0.1344	
Data / restraints / parameters	19054 / 2581 / 1038	
Goodness-of-fit on F^2	1.086	
Final R indices [I>2σ(I)]	R1 = 0.0800, wR2 = 0.2243	
R indices (all data)	R1 = 0.1675, wR2 = 0.2471	
Largest diff. peak and hole	0.530 and -0.444 e.$Å^{-3}$	

Goodness-of-fit = $[\Sigma[w(F_o^2 - F_c^2)^2]/N_{observns} - N_{params})]^{1/2}$, all data.

R1 = $\Sigma(|F_o| - |F_c|) / \Sigma |F_o|$. $wR2 = [\Sigma[w(F_o^2 - F_c^2)^2] / \Sigma [w(F_o^2)^2]]^{1/2}$.

S7.2 Ph-VP-CS (label22149s)

Data collection

A colorless crystal (approximate dimensions 0.333 × 0.282 × 0.251 mm^3) was placed onto the tip of a MiTeGen loop and mounted on a Bruker Venture D8 diffractometer equipped with a PhotonIII detector at 153(2) K.

The data collection was carried out using Mo Kα radiation (graphite monochromator) with a frame time of 60, 45, and 1 seconds and a detector distance of 5.00 cm. A collection strategy was calculated and complete data to a resolution of 0.77 Å with a redundancy of 8.4 were collected. Thirteen major sections of frames were collected with 1º ω and ϕ scans. The total exposure time was 17.92 hours. The frames were integrated with the Bruker SAINT software package[4] using a narrow-frame algorithm. The integration of the data using a triclinic unit cell yielded a total of 165073 reflections to a maximum θ angle of 27.53° (0.77 Å resolution), of which 19524 were independent (average redundancy 8.455, completeness = 99.8%, R_{int} = 6.92%, R_{sig} = 4.41%) and 15468 (79.23%) were greater than $2\sigma(F^2)$. The final cell constants of a = 16.3882(7) Å, b = 16.4992(7) Å, c = 19.1849(8) Å, α = 111.8930(10)°, β = 111.740(2)°, γ = 97.594(2)°, volume = 4245.5(3) $Å^3$, are based upon the refinement of the XYZ-centroids of 9330 reflections above 20 σ(I) with 4.556° < 2θ < 54.53°. Data were corrected for absorption effects using the Multi-Scan method (SADABS[5]). The ratio of minimum to maximum apparent transmission was 0.740. The calculated minimum and maximum transmission coefficients (based on crystal size) are 0.9720 and 0.9790.

Structure solution and refinement

The space group P-1 was determined based on intensity statistics and the lack of systematic absences. The structure was solved and refined using the SHELX suite of programs.[6, 7] An intrinsic-methods solution was calculated, which provided most non-hydrogen atoms from the E-map. Full-matrix least squares / difference Fourier cycles were performed, which located the remaining non-hydrogen atoms. All non-hydrogen atoms were refined with anisotropic displacement parameters with exception of the solvent molecules. The hydrogen atoms were placed in ideal positions and refined as riding atoms with relative isotropic displacement parameters. Disorder was refined for CS, tba, and the solvent molecules. The disorder ratio for CS is 57:43. The final anisotropic full-matrix least-squares refinement on F^2 with 1202 variables converged at R1 = 9.27%, for the observed data and wR2 = 28.05% for all data. The goodness-of-fit was 1.031. The largest peak in the final difference electron density synthesis was 0.869 $e^-/Å^3$ and the largest hole was -0.459 $e^-/Å^3$ with an RMS deviation of 0.106 $e^-/Å^3$. On the basis of the final model, the calculated density was 1.114 g/cm^3 and F(000), 1536 e^-.

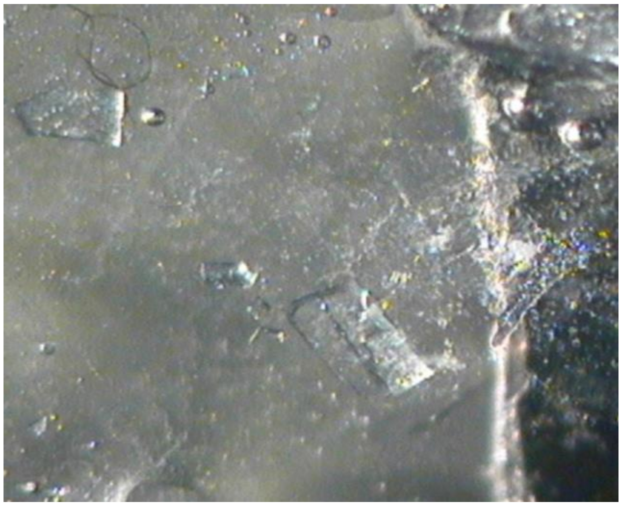

Figure S122. Image of bulk material.

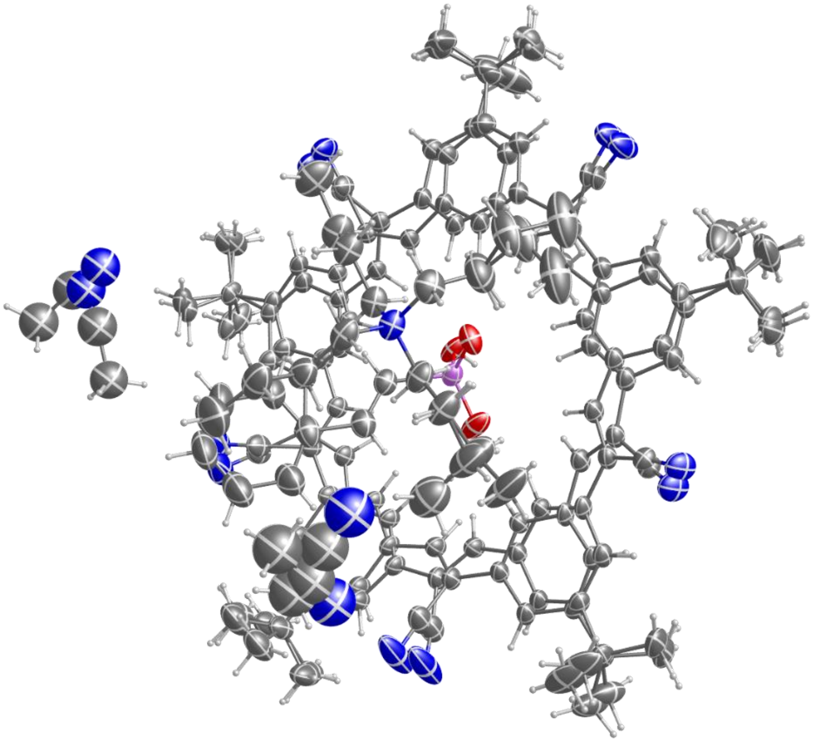

Figure S123. Formula unit.

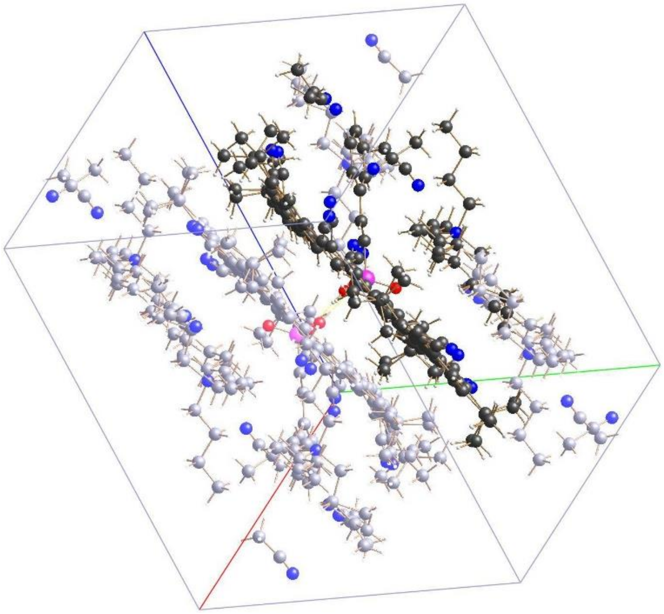

Figure 124. Dimer in solvent pocket.

Table 4. Crystal data and structure refinement.

CCDC deposition number	2263987	
Empirical formula	C93 H115 N8 O3 P	
Formula weight	1423.89	
Crystal color, shape, size	colorless block, 0.333 × 0.282 × 0.251 mm^3	
Temperature	153(2) K	
Wavelength	0.71073 Å	
Crystal system, space group	Triclinic, P-1	
Unit cell dimensions	a = 16.3882(7) Å	α = 111.8930(10)°.
	b = 16.4992(7) Å	β = 111.740(2)°.
	c = 19.1849(8) Å	γ = 97.594(2)°.
Volume	4245.5(3) $Å^3$	
Z	2	
Density (calculated)	1.114 Mg/m^3	
Absorption coefficient	0.085 mm^{-1}	
F(000)	1536	
Data collection		
Diffractometer	Venture D8, Bruker	
Source	Iμ3.0, Incoatec	
Detector	Photon III	
Theta range for data collection	2.088 to 27.528°.	
Index ranges	-21<=h<=21, -21<=k<=21, -24<=l<=24	
Reflections collected	165073	
Independent reflections	19524 [Rint = 0.0692]	
Observed Reflections	15468	
Completeness to theta = 25.242°	99.9 %	
Solution and Refinement		
Absorption correction	Semi-empirical from equivalents	
Max. and min. transmission	0.7456 and 0.5517	
Solution	Intrinsic methods	
Refinement method	Full-matrix least-squares on F^2	
Weighting scheme	w = $[\sigma^2 Fo^2 + AP^2 + BP]^{-1}$, with	
	P = $(Fo^2 + 2\ Fc^2)/3$, A = , B =	
Data / restraints / parameters	19524 / 2582 / 1202	
Goodness-of-fit on F^2	1.031	
Final R indices [I>2σ(I)]	R1 = 0.0927, wR2 = 0.2592	
R indices (all data)	R1 = 0.1078, wR2 = 0.2805	
Largest diff. peak and hole	0.869 and -0.459 e.$Å^{-3}$	

Goodness-of-fit = $[\Sigma[w(F_o^2 - F_c^2)^2]/N_{observns} - N_{params})]^{1/2}$, all data.

R1 = $\Sigma(|F_o| - |F_c|) / \Sigma |F_o|$. $wR2 = [\Sigma[w(F_o^2 - F_c^2)^2] / \Sigma [w(F_o^2)^2]]^{1/2}$.

S8. Sorting, Competition and Depolymerization Based on Modular Anion Library

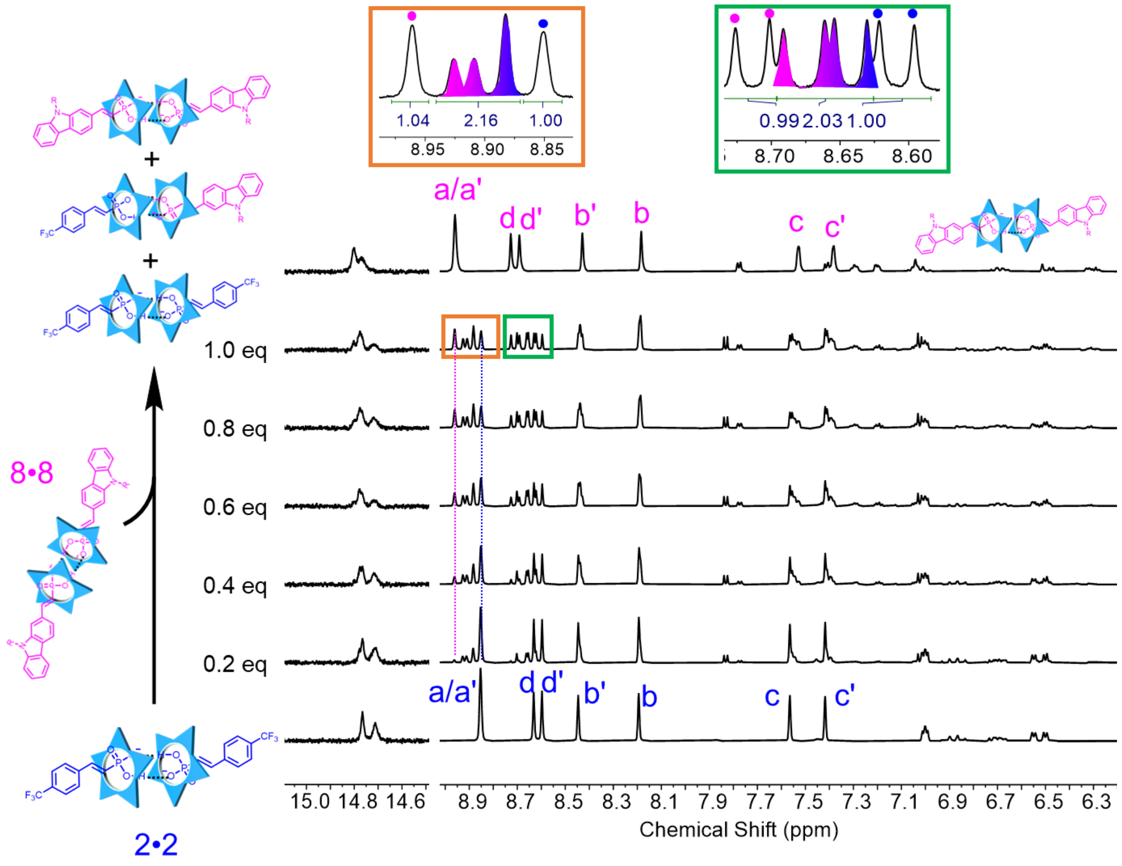

Figure 125. Self-sorting between anion dimers of **CF3Phenyl-VP** (**2,** blue) and **Carbazole-VP** (**8,** magenta), heterodimer (**2•8**) is produced (1 mM, 298 K, 600 MHz, CD_2Cl_2). NMR spectra of **2•2** and **8•8** dimers are presented at the very bottom and the very top. The proton patterns of proton a (orange box) and d (green box) are used to quantitatively analyze the distribution of homodimers and the heterodimer.

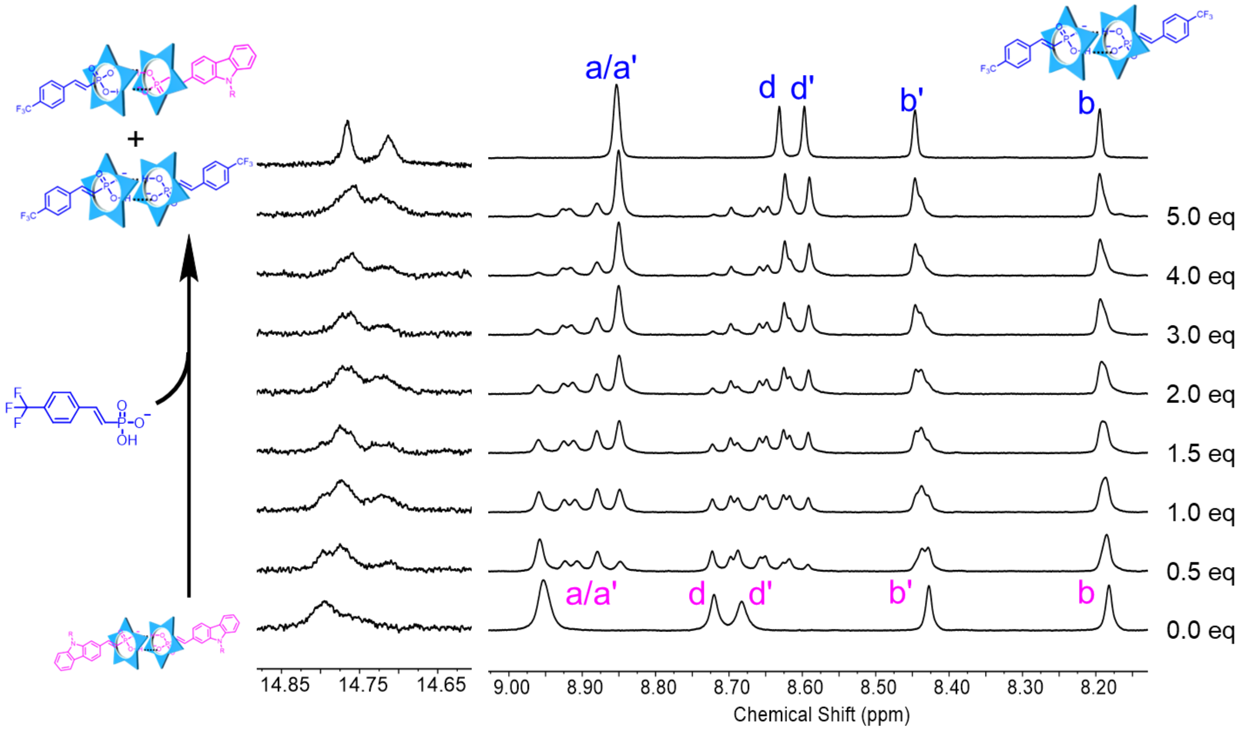

Figure 126. Competition between anion dimers of **8•8** and tetrabutylammonium salt of anion **2** (1 mM, 298 K, 600 MHz, CD_2Cl_2).

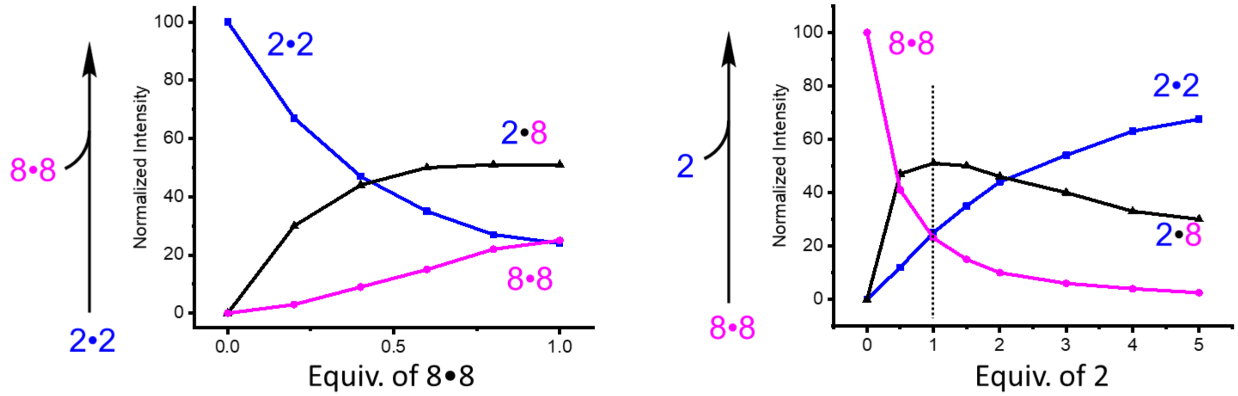

Figure 127. Quantitative analysis of homodimer and heterodimer distribution based on the integration of proton a from three types of dimers generated by vinyl phosphonate 2 and 8.

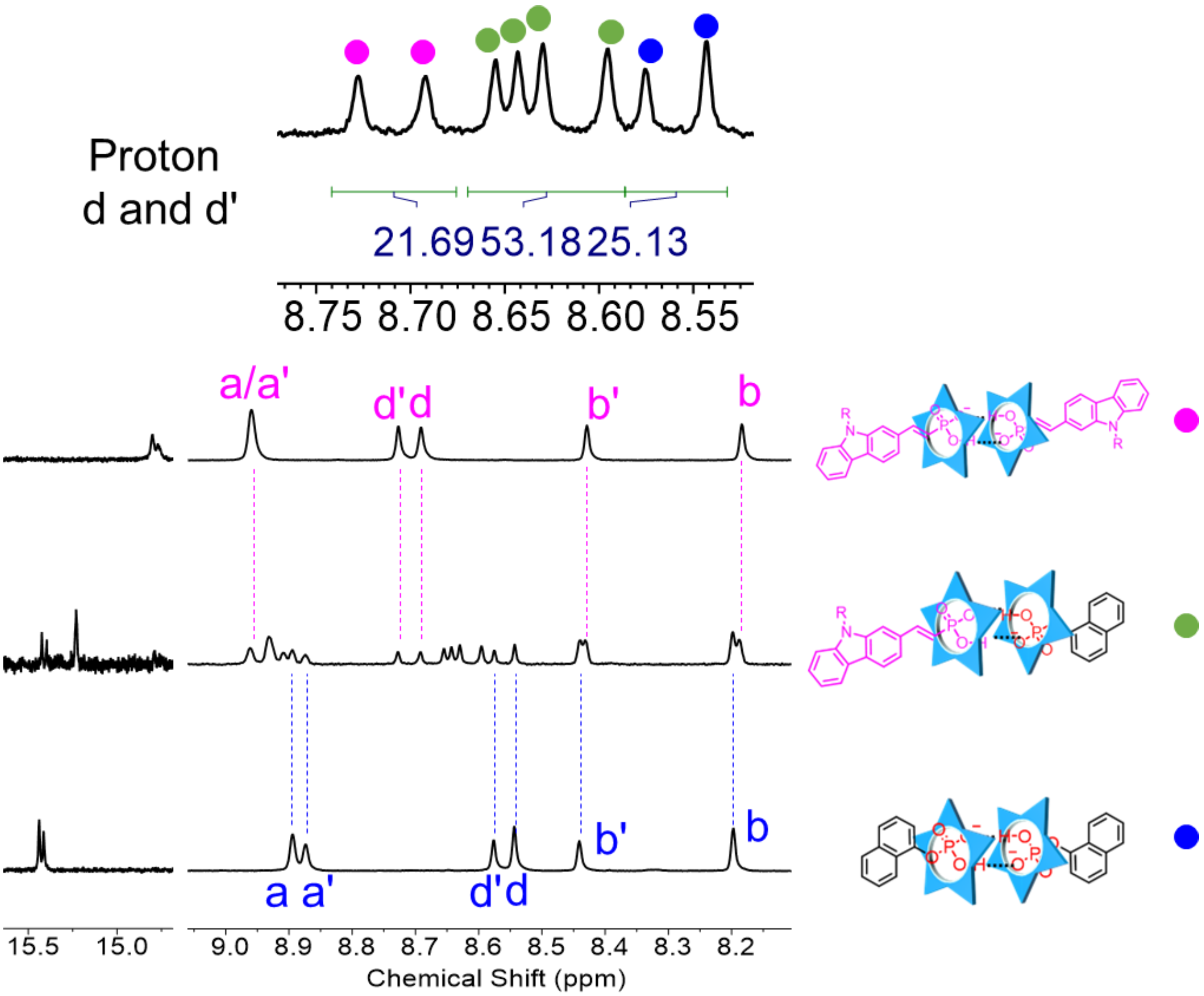

Figure 128. Self-sorting between anion dimers of **Carbazole-VP** (**8,** magenta) and naphthyl phosphate (**P**, blue), heterodimer (**P•8**) is produced (1 mM, 298 K, 600 MHz, CD_2Cl_2). NMR spectra of PP and BB dimers are presented at the very bottom and the very top. The proton pattern of proton d is used to quantitatively analyze the distribution of homodimers and the heterodimer.

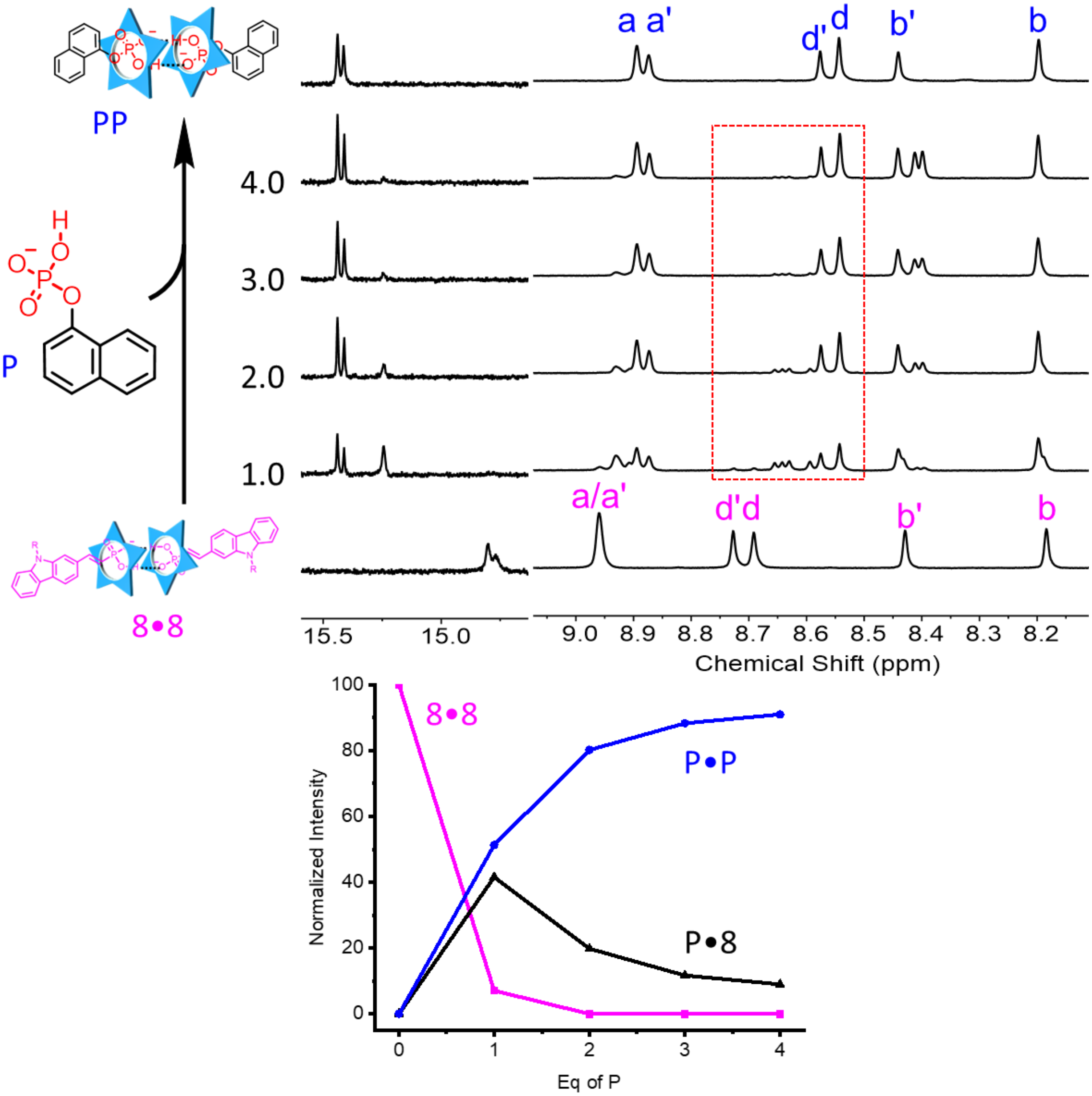

Figure 129. Competition between anion dimers of **8•8** and tetrabutylammonium salt of anion **P** (1 mM, 298 K, 600 MHz, CD_2Cl_2). The proton pattern of proton d (red box) is used to quantitatively analyze the distribution of homodimers and the heterodimer.

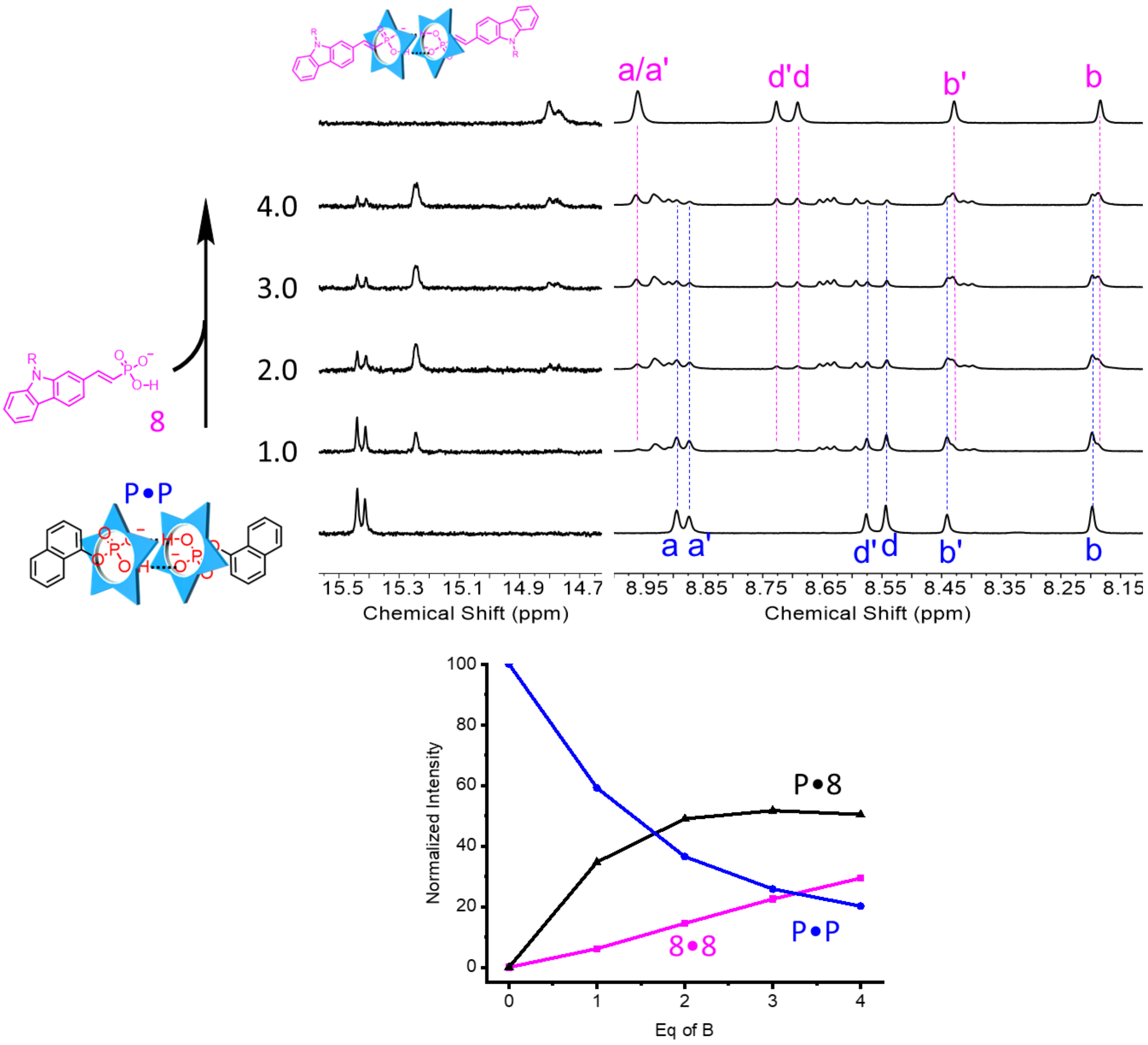

Figure 130. Competition between anion dimers of **P•P** and tetrabutylammonium salt of anion **8** (1 mM, 298 K, 600 MHz, CD_2Cl_2). The proton pattern of proton d is used to quantitatively analyze the distribution of homodimers and the heterodimer.

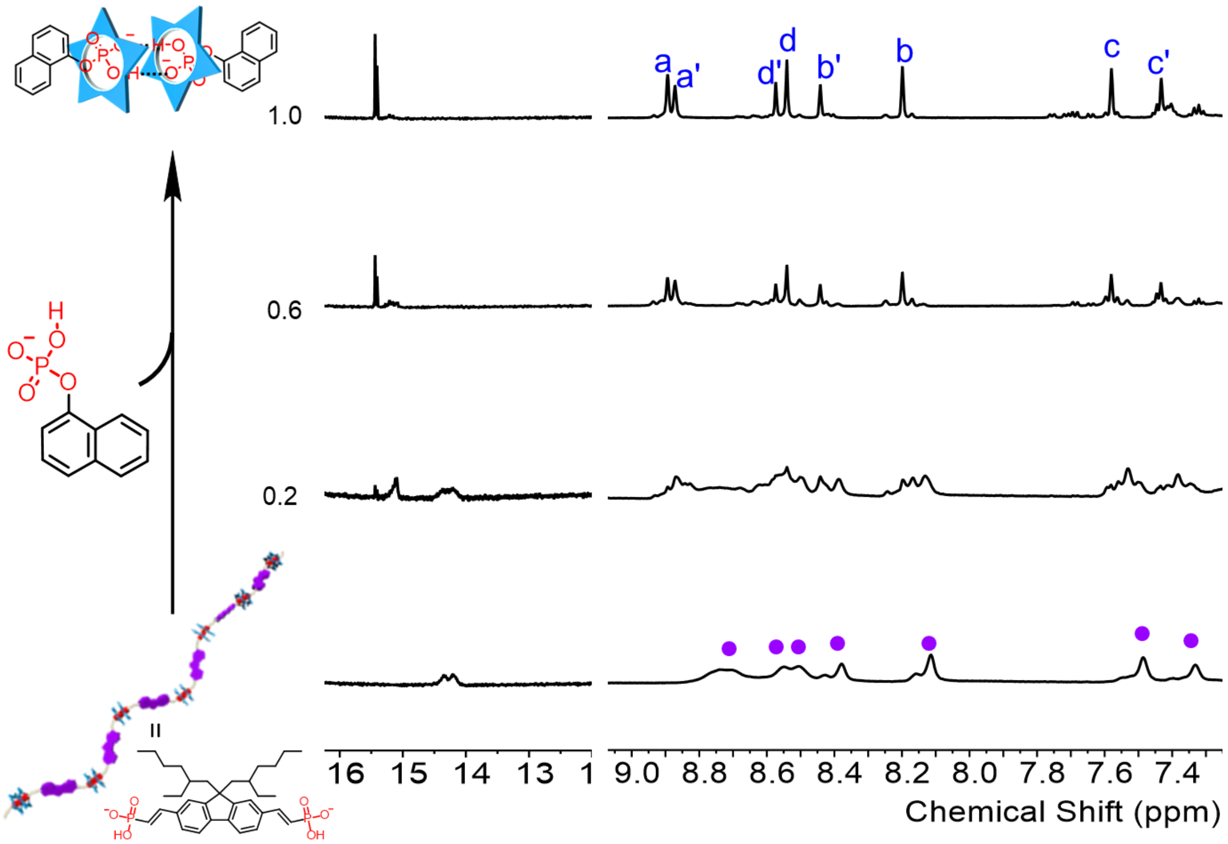

Figure 131. Depolymerization of supramolecular polymers using tetrabutylammonium salt of naphthyl phosphate **P** (5 mM, 298 K, 600 MHz, CD_2Cl_2). Purple dots indicate polymer's proton signal.

Table 5. Diffusion data comparison of supramolecular homopolymer and copolymer at the same cyanostar concentration.

5 mM Cyanostar	Proton Signal (ppm)	Diffusion Coefficient (10^{-11} m^2/s)	Average (10^{-11} m^2/s)
50:50% 11:12 (copolymer)	8.35	6.22106	6.4 ± 0.1
	8.11	6.42907	
	7.46	6.31968	
	7.31	6.48005	
100% 12 (homopolymer)	8.39	8.96395	9.3 ± 0.2
	8.13	9.50585	
	7.50	9.31195	
	7.34	9.30019	

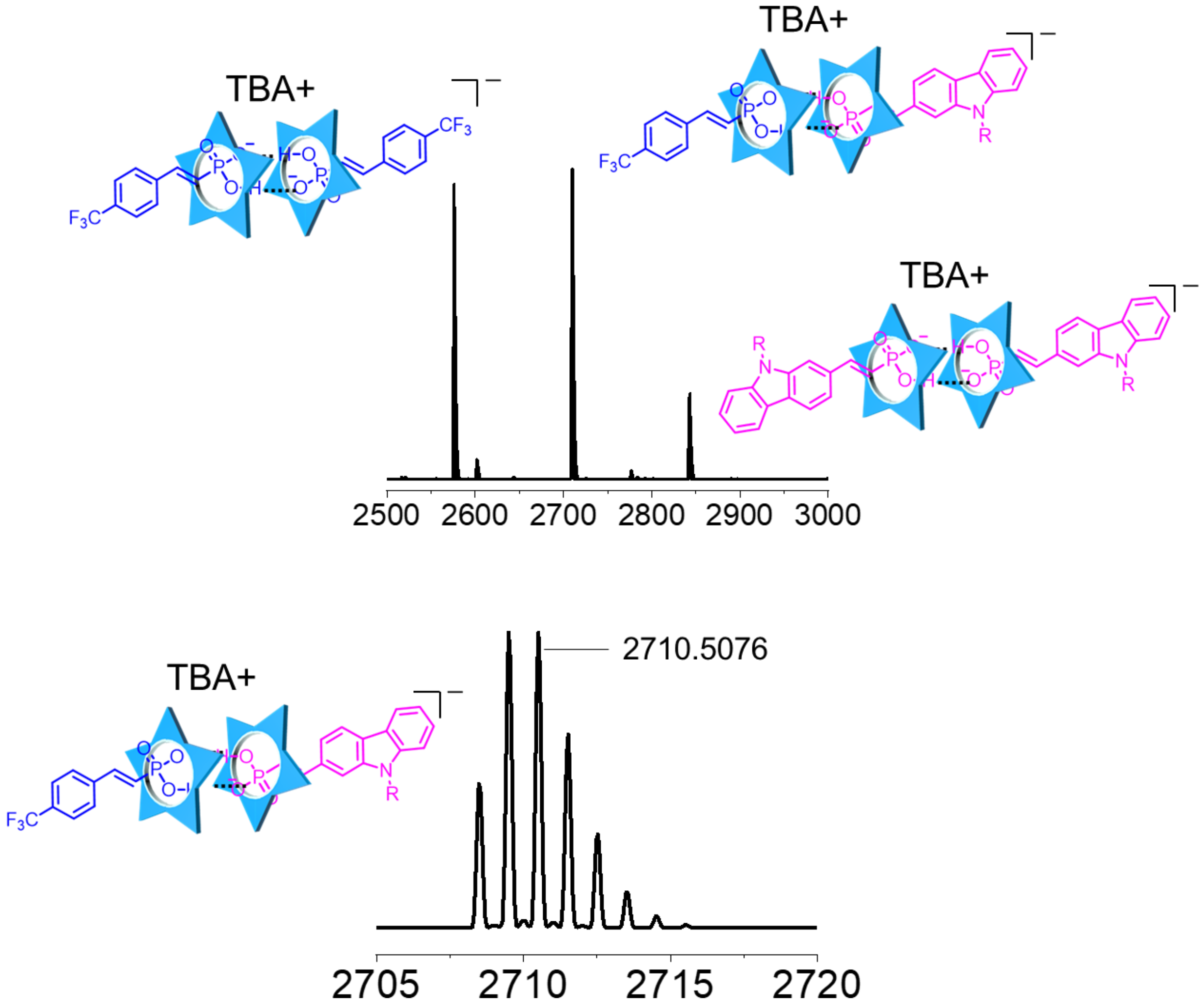

Figure 132. HRMS of heterodimer **2•8**.

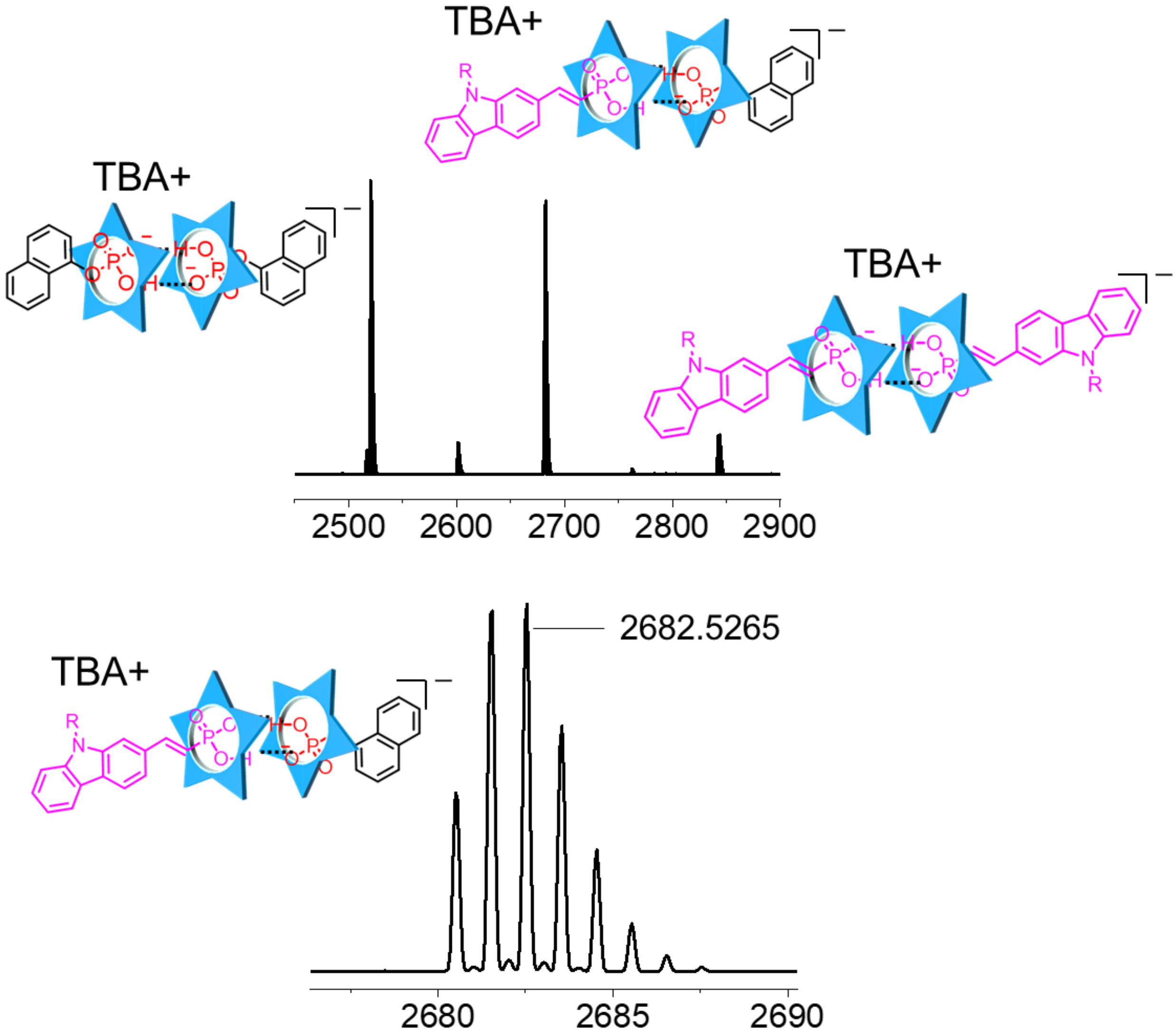

Figure 133. HRMS of heterodimer **P•8**.

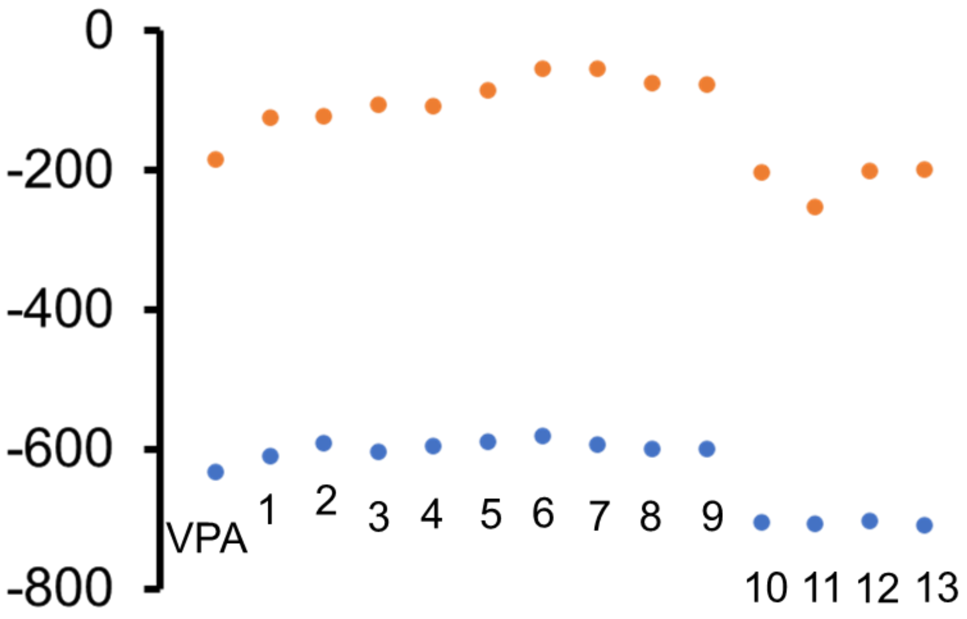

Figure 134. Summary of electrostatic surface potential of 14 vinyl phosphonates (method: RB3LYP, basis set: 6-31G(D)).

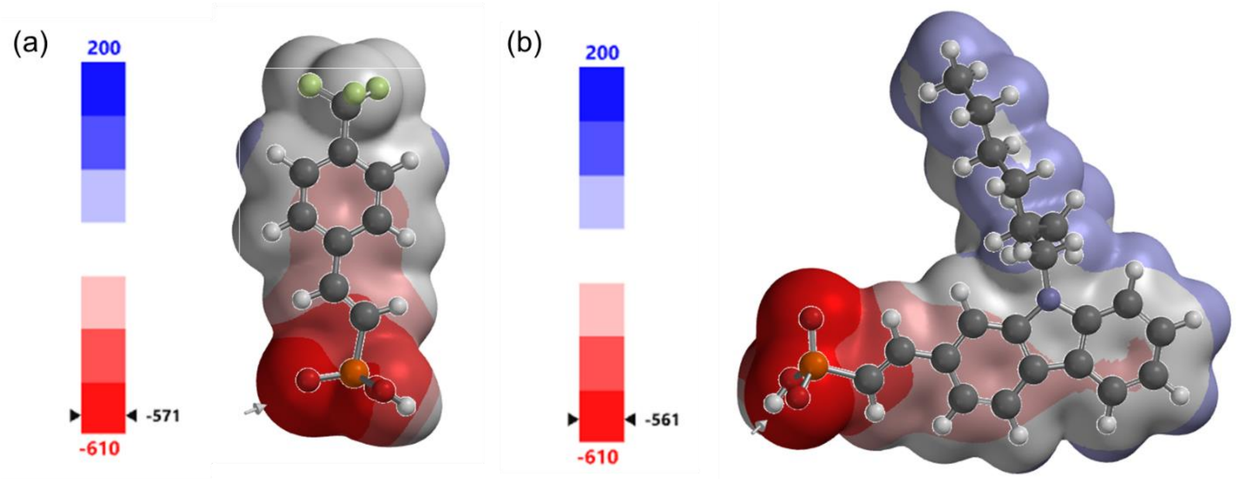

Figure 135. Electrostatic surface potential comparison of (a) **CF_3Phenyl-VP, 2** and (b) **Carbazole-VP, 8** (method: RB3LYP, basis set: 6-31G(D)). The value close to the molecule indicates the electrostatic surface potential of the oxygen atom from the phosphonate anion, unit is kJ.

S9. Supramolecular Polymer Literature Including Multiple Monomers

Table 6. Summary of literature including multiple monomers.

Title and Lead Authors	#	Interaction Type			# of Citations	# of Building Blocks
		HB	H•G	M-L		
Strong dimerization of ureidopyrimidones via quadruple hydrogen bonding. **Meijer, E. W.** *et al.*	1	√			865	**11**
Controlling the structure and photophysics of fluorophore dimers using multiple cucurbit [8] uril clampings. **Scherman, O. A.** *et al.*	2		√		40	**10**
Bis [4′-(4-anilino)-2, 2′: 6′, 2 ″-terpyridine] transition-metal complexes: electrochemically active monomers with a range of magnetic and optical properties for assembly of metallo oligomers and macromolecules. **Colbran, S. B.** *et al.*	3			√	68	**9**
Monosaccharides as versatile units for water-soluble supramolecular polymers. **Meijer, E. W.** *et al.*	4	√			26	**8**
Highly efficient and tunable filtering of electrons' spin by supramolecular chirality of nanofiber-based materials. **Meijer, E. W.** *et al.*	5	√			130	**5**
Living supramolecular polymerization of fluorinated cyclohexanes. **Von Delius, M.** *et al.*	6	√			36	**5**
Toward a single-layer two-dimensional honeycomb supramolecular organic framework in water. **Li, Z. T.** *et al.*	7		√		328	**4**
Supramolecular block copolymers with cucurbit[8]uril in water. **Scherman, O. A.** *et al.*	8		√		321	**4**
Elucidating the ordering in self-assembled glycocalyx mimicking supramolecular copolymers in water. **Meijer, E. W.** *et al.*	9	√			43	**4**
Thioamides: versatile bonds to induce directional and cooperative hydrogen bonding in supramolecular polymers. **Meijer, E. W.** *et al.*	10	√			46	**3**
Modular supramolecular dimerization of optically tunable extended aryl viologens**. Scherman, O. A.** *et al.*	11		√		35	**3**
Transition-metal complexes of 4′(4-anilino)-2, 2′: 6′, 2 ″-terpyridine (and derivatives): versatile building blocks for construction of metallooligomers and macromolecules. **Colbran, S. B.** *et al.*	12			√	26	**3**
Total		6	4	2		

Notes:

(1) HB refers to hydrogen bonding arrays

(2) H•G refers to π-stacked dimers encapsulated by cucurbit[n]uril hosts

(3) M-L refers to terpyridine ligands driven by metal-coordination

S10. References

1. R. E. Fadler, A. Al Ouahabi, B. Qiao, V. Carta, N. F. Konig, X. Gao, W. Zhao, Y. Zhang, J. F. Lutz and A. H. Flood, *J. Org. Chem.*, 2021, **86**, 4532-4546.
2. W. Zhao, B. Qiao, J. Tropp, M. Pink, J. D. Azoulay and A. H. Flood, *J. Am. Chem. Soc.*, 2019, **141**, 4980-4989.
3. W. Zhao, J. Tropp, B. Qiao, M. Pink, J. D. Azoulay and A. H. Flood, *J. Am. Chem. Soc.*, 2020, **142**, 2579-2591.
4. B. A. SAINT V8.40A (2020), Madison, WI.
5. L. H.-I. Krause, R;Sheldrick, GM; Stalke, D. , *J. Appl. Cryst.*, 2015, 3.
6. G. Sheldrick, M., *Acta Crystallogr. Sect. A*, 2015, **71**, 3.
7. G. Sheldrick, M., *Acta Crystallogr. Sect. C*, 2015, **71**, 3.
8. G. A. Jeffrey, *An introduction to hydrogen bonding*, Oxford university press New York, 1997.
9. W. Zhao, A. H. Flood and N. G. White, *Chem. Soc. Rev.*, 2020, **49**, 7893-7906.
10. N. G. White, *CrystEngComm*, 2019, **21**, 4855-4858.
